# Octreotide-LAR in later-stage autosomal dominant polycystic kidney disease (ALADIN 2): A randomized, double-blind, placebo-controlled, multicenter trial

**DOI:** 10.1371/journal.pmed.1002777

**Published:** 2019-04-05

**Authors:** Norberto Perico, Piero Ruggenenti, Annalisa Perna, Anna Caroli, Matias Trillini, Sandro Sironi, Antonio Pisani, Eleonora Riccio, Massimo Imbriaco, Mauro Dugo, Giovanni Morana, Antonio Granata, Michele Figuera, Flavio Gaspari, Fabiola Carrara, Nadia Rubis, Alessandro Villa, Sara Gamba, Silvia Prandini, Monica Cortinovis, Andrea Remuzzi, Giuseppe Remuzzi, for the ALADIN 2 Study Group

**Affiliations:** 1 Istituto di Ricerche Farmacologiche Mario Negri IRCCS, Bergamo, Italy; 2 Unit of Nephrology and Dialysis, Azienda Socio Sanitaria Territoriale Papa Giovanni XXIII, Bergamo, Italy; 3 Department of Diagnostic Radiology, Azienda Socio Sanitaria Territoriale Papa Giovanni XXIII, Bergamo, Italy; 4 Department of Medicine and Surgery, University of Milano–Bicocca, Milan, Italy; 5 Chair of Nephrology, Department of Public Health, University of Naples Federico II, Naples, Italy; 6 Department of Advanced Biomedical Sciences, University of Naples Federico II, Naples, Italy; 7 Nephrology and Dialysis Department, Ca’ Foncello Hospital, Treviso, Italy; 8 Department of Radiology, Ca’ Foncello Hospital, Treviso, Italy; 9 Unit of Nephrology and Dialysis, San Giovanni di Dio Hospital, Agrigento, Italy; 10 Radiology Unit, Vittorio Emanuele Policlinico Hospital, Catania, Italy; 11 Department of Management, Information and Production Engineering, University of Bergamo, Bergamo, Italy; 12 L. Sacco Department of Biomedical and Clinical Sciences, University of Milan, Milan, Italy; Royal Derby Hospital, UNITED KINGDOM

## Abstract

**Background:**

Autosomal dominant polycystic kidney disease (ADPKD) is the most frequent genetically determined renal disease. In affected patients, renal function may progressively decline up to end-stage renal disease (ESRD), and approximately 10% of those with ESRD are affected by ADPKD. The somatostatin analog octreotide long-acting release (octreotide-LAR) slows renal function deterioration in patients in early stages of the disease. We evaluated the renoprotective effect of octreotide-LAR in ADPKD patients at high risk of ESRD because of later-stage ADPKD.

**Methods and findings:**

We did an internally funded, parallel-group, double-blind, placebo-controlled phase III trial to assess octreotide-LAR in adults with ADPKD with glomerular filtration rate (GFR) 15–40 ml/min/1.73 m^2^. Participants were randomized to receive 2 intramuscular injections of 20 mg octreotide-LAR (*n =* 51) or 0.9% sodium chloride solution (placebo; *n =* 49) every 28 days for 3 years. Central randomization was 1:1 using a computerized list stratified by center and presence or absence of diabetes or proteinuria. Co-primary short- and long-term outcomes were 1-year total kidney volume (TKV) (computed tomography scan) growth and 3-year GFR (iohexol plasma clearance) decline. Analyses were by modified intention-to-treat. Patients were recruited from 4 Italian nephrology units between October 11, 2011, and March 20, 2014, and followed up to April 14, 2017. Baseline characteristics were similar between groups. Compared to placebo, octreotide-LAR reduced median (95% CI) TKV growth from baseline by 96.8 (10.8 to 182.7) ml at 1 year (*p =* 0.027) and 422.6 (150.3 to 695.0) ml at 3 years (*p =* 0.002). Reduction in the median (95% CI) rate of GFR decline (0.56 [−0.63 to 1.75] ml/min/1.73 m^2^ per year) was not significant (*p =* 0.295). TKV analyses were adjusted for age, sex, and baseline TKV. Over a median (IQR) 36 (24 to 37) months of follow-up, 9 patients on octreotide-LAR and 21 patients on placebo progressed to a doubling of serum creatinine or ESRD (composite endpoint) (hazard ratio [HR] [95% CI] adjusted for age, sex, baseline serum creatinine, and baseline TKV: 0.307 [0.127 to 0.742], *p =* 0.009). One composite endpoint was prevented for every 4 treated patients. Among 63 patients with chronic kidney disease (CKD) stage 4, 3 on octreotide-LAR and 8 on placebo progressed to ESRD (adjusted HR [95% CI]: 0.121 [0.017 to 0.866], *p =* 0.036). Three patients on placebo had a serious renal cyst rupture/infection and 1 patient had a serious urinary tract infection/obstruction, versus 1 patient on octreotide-LAR with a serious renal cyst infection. The main study limitation was the small sample size.

**Conclusions:**

In this study we observed that in later-stage ADPKD, octreotide-LAR slowed kidney growth and delayed progression to ESRD, in particular in CKD stage 4.

**Trial registration:**

ClinicalTrials.gov NCT01377246; EudraCT: 2011-000138-12.

## Abstract 2

In this double-blind, placebo-controlled, phase 3 randomised trial, Piero Ruggenenti and co-workers assessed the renoprotective effects of Octreotide-LAR in adults with ADPKD at high risk of ESRD

## Author summary

**Why was this study done?:**

- Autosomal dominant polycystic kidney disease is the most frequent genetically determined renal disease and affects approximately 10% of patients on chronic dialysis therapy because of end-stage kidney disease.
- The disease is characterized by relentless growth of renal cysts, with consequent disruption of normal parenchyma and progressive reduction of kidney function up to terminal kidney failure.
- Previous studies found that octreotide long-acting release (octreotide-LAR), an analog of somatostatin, slowed cyst growth and progressive renal function loss in patients with normal or slightly impaired kidney function.

**What did the researchers do and find?:**

- In this randomized and placebo-controlled trial, we evaluated whether the renoprotective effect of octreotide-LAR could be extended to patients with severe renal disease, that is, with chronic kidney disease stage 3b or 4.
- One-hundred patients with estimated glomerular filtration rate 15–40 ml/min/1.73 m^2^ were randomized to receive two 20-mg intramuscular injections of octreotide-LAR (*n =* 51) or sodium chloride (placebo; *n =* 49) every 28 days for 3 years.
- We found that 3-year treatment with octreotide-LAR did not appreciably affect glomerular filtration rate decline compared to placebo, but significantly slowed cyst growth and progression to end-stage kidney failure, in particular in patients with more severe renal insufficiency (stage 4) to start with, and was safe and tolerated well.

**What do these findings mean?:**

- Our present data—combined with previous evidence of a protective effect against kidney and liver volume growth and renal function loss in patients with normal or moderately reduced kidney function—indicate that octreotide-LAR could be a novel disease-modifying therapy that benefits patients with autosomal dominant polycystic kidney disease, including those with later-stage disease.
- Octreotide-LAR is an expensive medication. The identification of a subgroup of patients with CKD stage 4, accounting for approximately 10% to 15% of patients with autosomal dominant polycystic kidney disease, who are at high risk of kidney failure and at the same time may benefit the most from treatment may help increase the cost-effectiveness of octreotide-LAR for the prevention of end-stage renal disease (and related costs of renal replacement therapy and its complications) in this population.

## Introduction

Every year worldwide, 4.8 to 15.3 per million persons with autosomal dominant polycystic kidney disease (ADPKD) progress to end-stage renal disease (ESRD) [1]. In Europe approximately 10% of all patients undergoing renal replacement therapy have ADPKD [2]. In ADPKD patients, mutations in the genes encoding for either polycystin 1 or polycystin 2 result in polycystin complex dysfunction. This dysfunction results in reduced intracellular calcium concentration, leading to high activity of adenylyl cyclase enzyme and up-regulation of 3′,5′-cyclic adenosine monophosphate (cAMP) levels [3]. In the kidneys, the sustained high intracellular cAMP levels in the proximal and distal nephrons as well as collecting ducts lead to aberrant tubular epithelial cell proliferation and chloride-driven fluid secretion, the 2 key components of the process of cyst formation and growth in ADPKD [4]. Uncontrolled cyst growth results in crowding of adjacent nephrons, destruction of normal renal parenchyma, and, eventually, substantial enlargement of the kidneys and progressive renal failure [5].

Somatostatin, an endogenous cyclic peptide with pleiotropic endocrine, paracrine, and autocrine actions [6], inhibits in vitro adenylyl cyclase and post-cAMP events in shark rectal gland [7]. High-affinity, specific SST2 receptors for somatostatin are expressed in human kidney [8] and co-localize with adenylyl cyclase in the basolateral membrane of renal tubular epithelial cells [9]. This evidence suggested the possibility of targeting SST2 receptors with a somatostatin analog in order to limit cell proliferation and fluid secretion by inhibiting cAMP production in renal cells. In a pilot study, we found that octreotide long-acting release (octreotide-LAR), a synthetic somatostatin analog with longer half-life and higher SST2 affinity than the naïve polypeptide [10], slowed the increase in total kidney, and even liver, volume compared with placebo in 12 patients with ADPKD [11,12]. A subsequent study in a rodent polycystic kidney disease model found that the protective effect of somatostatin analogs against hepatorenal cystogenesis was associated with decreased cAMP production [13].

In the “A Long-Acting somatostatin on DIsease progression in Nephropathy due to autosomal dominant polycystic kidney disease” (ALADIN) trial, we compared octreotide-LAR for 3 years versus placebo in adults with ADPKD with normal kidney function or mild-to-moderate renal insufficiency (estimated glomerular filtration rate [eGFR] ≥ 40 ml/min/1.73 m^2^). Results from ALADIN showed a significant reduction in kidney growth and cyst growth, and stabilization of glomerular filtration rate (GFR) at 1 year compared with progressive decline in GFR in the placebo group [14]. The ALADIN 2 trial was designed to assess the effect of octreotide-LAR on kidney growth at 1 year and GFR decline at 3 years in patients with ADPKD with more severe renal insufficiency (chronic kidney disease [CKD] stage 3b to 4).

## Methods

### Prospective protocol and analysis plan

This was an internally funded, prospective, parallel-group, double-blind, placebo-controlled phase III trial aimed at assessing the renal effects of the somatostatin analog octreotide-LAR in adults with later-stage ADPKD. GFR, estimated by the Modification of Diet in Renal Disease Study 4-variable equation (eGFR), at inclusion was 15 to 40 ml/min/1.73 m^2^. Participants were randomized to receive 2 intramuscular injections of 20 mg octreotide-LAR (*n =* 51) or 0.9% sodium chloride solution (placebo; *n =* 49) every 28 days for 3 years. Central randomization was 1:1 using a computerized list stratified by center and presence or absence of diabetes or proteinuria. Co-primary short- and long-term outcomes were 1-year total kidney volume (TKV) growth assessed by computed tomography (CT) scans and 3-year decline of GFR directly measured with the iohexol plasma clearance technique (for further details see S2 Text).

### Participants, setting, and ethics

Participants were identified among patients with ADPKD referred to the outpatient clinics of 4 hospitals in Italy coordinated by the Istituto di Ricerche Farmacologiche Mario Negri IRCCS (see S1 Appendix).

Adult (>18 years) men and women with ADPKD according to Ravine criteria [15] and eGFR between 15 and 40 ml/min/1.73 m^2^ were eligible. We excluded patients with confounding factors that could affect renal function loss independently of kidney growth and treatment allocation (HbA1c > 8%, systolic/diastolic blood pressure > 180/110 mm Hg, urinary protein excretion > 3 g/24 h); patients with abnormal urinalysis suggestive of concomitant, clinically significant glomerular disease; patients with urinary tract lithiasis or infection; patients with symptomatic gallstones, cancer, or major systemic disease; those who were unable to provide informed consent; and pregnant, lactating, or potentially childbearing women without adequate contraception (for further details, please see S1 Protocol and https://clinicaltrials.gov/ct2/show/NCT01377246).

The study protocol was approved by each site’s institutional review board: the Comitato di Bioetica of the Local Health Authority of the Province of Bergamo, the Comitato Bioetico of the Local Health Authority of the Province of Agrigento, the Comitato Etico of the University of Naples Federico II, and the Comitato Etico per la Sperimentazione of the Province of Treviso. The Comitato Etico of the Local Health Authority of Lecce and the Comitato Etico of the Fondazione IRCCS Cà Granda Ospedale Maggiore di Milano also approved the protocol, but the centers of Lecce and Milan did not include patients. Written informed consent was obtained from all participants in compliance with the Declaration of Helsinki. Data were locally recorded in dedicated electronic case report forms and centralized into the database at the coordinating center. This study is reported as per the Consolidated Standards of Reporting Trials (CONSORT) guideline (S1 Checklist).

### Randomization

Eligible participants were stratified for the presence or absence of risk factors that might affect renal function loss (diabetes mellitus and/or 24-hour proteinuria > 1 g). Participants were randomly assigned to treatment groups 1:1 by an independent investigator (G. A. Giuliano see: ALADIN 2 Study Organization in S1 Appendix), using a web-based, computer-generated randomization list created using SAS (version 9.2), stratified by center and the presence or absence of risk factors with a random block size of 4 or 8.

### Baseline assessment and procedures

At the baseline evaluation, blood pressure was measured in the dominant arm after a 10-minute rest in the sitting position. The mean of 3 measurements, taken 2 minutes apart, was recorded for statistical analyses. Blood samples were collected in the morning after overnight fasting for routine blood tests including renal and liver function tests, and peripheral blood cell counts. Twenty-four-hour urine collections were sampled for protein, albumin, sodium, creatinine, urea, glucose, phosphorus excretion, and osmolality assessment. Additionally, albumin-to-creatinine ratio was assessed in spot morning urine samples. GFR was centrally measured by iohexol plasma clearance technique [16]. TKV was quantified on CT scans. After baseline evaluation and every 28 days thereafter for 3 years, participants allocated to active treatment received two 20-mg intramuscular injections of octreotide-LAR, whereas those assigned to placebo were given 2 intramuscular injections of 0.9% sodium chloride solution [14]. All injections were administered at the clinic. Any drug administration was registered in patient case record forms for treatment adherence recording.

Vital sign, physical examination, and laboratory variables were assessed every 3 months, together with gallbladder biliary tract and kidney ultrasound assessment. GFR was measured by the iohexol plasma clearance technique every 6 months during the 3-year follow-up. Blood samples for the measurement of iohexol plasma concentration were collected at 120, 180, 240, 300, 360, 420, and 480 minutes after the injection.

CT images were obtained as previously described [17,18] at baseline and the 1-year and 3-year visits the day after GFR measurement, and collected in Digital Imaging and Communications in Medicine (DICOM) format by the coordinating center for central quality control and subsequent analysis. Kidneys were manually outlined by trained operators using the ImageJ polyline method [19], and double-checked by a single operator (AC). All of the operators were blinded to patient treatment allocation (see S1 Text). TKV was obtained as volume of kidney outlines and finally corrected for height (height-adjusted TKV [htTKV], ml/m) [20] to adjust for sex-related volume differences. The use and choice of a contrast agent was left to the radiologist performing the test according to the center’s procedures.

All participants were encouraged to comply with dietary recommendations as per center practice. Adjustments of existing antihypertensive therapy were allowed to optimize blood pressure to a target of ≤130/80 mm Hg throughout the study. Concomitant changes in blood glucose and HbA1c levels and need for hypoglycemic therapy were carefully monitored during the follow-up. Appropriate treatments were allowed to maintain markers of mineral-bone metabolism and acid/base balance in recommended targets. If gallbladder sand or stones were documented during the scheduled serial ultrasound evaluations, treatment with ursodeoxycholic acid was prescribed. No patient received antidiuretic hormone antagonists.

### Outcome measures

The primary short-term outcome was absolute change in TKV, as measured by CT scan, from baseline to 1-year follow-up. The primary long-term outcome was the chronic rate of GFR decline from 6 months to study end as assessed by serial measurements of iohexol plasma clearance.

Secondary endpoints were the measurement of total liver and liver cyst volumes and a composite endpoint of progression to doubling of serum creatinine (versus baseline) or ESRD at 3-year follow-up. Sensitivity analyses considered 1- and 3-year changes in htTKV. Safety variables included vital signs, clinical laboratory tests, and adverse events.

Statistical analyses of endothelin and MCP-1 urinary excretion mentioned in the protocol were explorative in nature and were not performed because of fund constraints. Also, exploratory analyses of quality of life and societal costs were performed in a subgroup of consenting patients by using the Quality of Life Questionnaire–Version 1 of SF-36, validated in Italy, and the Short-Form Health and Labour Questionnaire. We preferred not to report the above results since in our opinion they were poorly informative. Consistent with the strategy previously adopted for the ALADIN trial [14,21], we decided to report data on liver volumes separately from those on renal outcomes.

### Sample size and statistical analysis

Sample size was estimated for the main prespecified outcome, absolute TKV change at 1 year, assuming use of a 2-group *t* test (2-sided) of the difference between octreotide-LAR and placebo. On the basis of data from the interim results of the ALADIN trial [14], a mean increase of 103.4 ml (SD 149.5) was expected in the placebo group at 1-year follow-up, and octreotide-LAR treatment was predicted to reduce such an increase from 103 ml to 0 ml. Based on these assumptions, and assuming 30% dropout, a sample size of 49 patients per group (total sample size 98) would give the trial 80% power to detect as statistically significant (α = 0.05, 2-tailed test) the expected difference in TKV change between the 2 treatment groups over 1 year.

As for the long-term primary endpoint at 3-year follow-up, assuming a yearly GFR decline (mean ± SD) of 6.31 ± 4.47 ml/min/1.73 m^2^ in the placebo group (data from ADPKD patients with severe renal insufficiency included in the REIN study [22]), the sample size of 49 patients per group was expected to provide the trial 81% power to detect as statistically significant (α = 0.05, 2-tailed test) a 50% (or larger) reduction in the rate of GFR decline (as observed for chronic GFR slopes in ALADIN [14]) in the octreotide-LAR treatment arm (i.e., from 6.31 to 3.16 ml/min/1.73 m^2^) compared to the placebo group.

All statistical analyses were done by modified intention-to-treat, using SAS (version 9.4) and Stata (version 12). All adjusted models included age and sex as covariates and 1, or maximum 2, additional baseline covariates [23]. Changes in TKV and htTKV at 1 and 3 years and all other between-group effects were assessed by nonparametric (because of non-normal data distribution) ANCOVA also adjusted for age, sex, and baseline TKV (or htTKV) using the SAS/IML “NParCov3” Macro [24]. GFR decline was assessed with a linear regression analysis and compared between groups with the Wilcoxon rank-sum test. Exploratory linear mixed models using SAS PROC MIXED were also used for TKV, htTKV, and GFR repeated measures, with age, sex, and baseline value as covariates. For the composite endpoint doubling of serum creatinine or dialysis, a Cox regression model was used, also adjusted for age, sex, and baseline serum creatinine and TKV. Between-group differences and their 95% confidence intervals (CIs) for TKV and htTKV were calculated using the SAS/IML “NParCov3” Macro [24]. The between-group difference in median GFR slope and its 95% CI were determined by means of Hodges–Lehmann estimation using the SAS PROC NPAR1WAY.

To assess whether and to what extent the treatment effect was affected by the severity of renal insufficiency, we evaluated in a post hoc, unplanned analysis, not mentioned in the protocol, all study outcomes in the 2 subgroups of patients with eGFR > 29 (range 30–44) or ≤29 (range 15–29) ml/min/1.73 m^2^ who, according to KDIGO recommendation statements [25], could be classified as patients with moderately to severely decreased eGFR (CKD stage 3b) or severely decreased eGFR (CKD stage 4), respectively.

Data are expressed as mean (SD), median (IQR), or number (%) unless otherwise specified. Percent changes were determined for each participant before calculating descriptive statistics. The normality assumption was assessed by means of the Shapiro–Wilk test. Adjustment for multiplicity was considered for the interim analysis (see S1 Protocol): no further adjustment for multiple testing was performed. All *p-*values are 2-sided. As planned in the study protocol, an interim analysis was performed by the independent data and safety monitoring board on March 3, 2014, and the board decided to continue the study as per protocol guidelines. This trial is registered with ClinicalTrials.gov (NCT01377246) and EudraCT (2011-000138-12).

## Results

**Fig 1:**
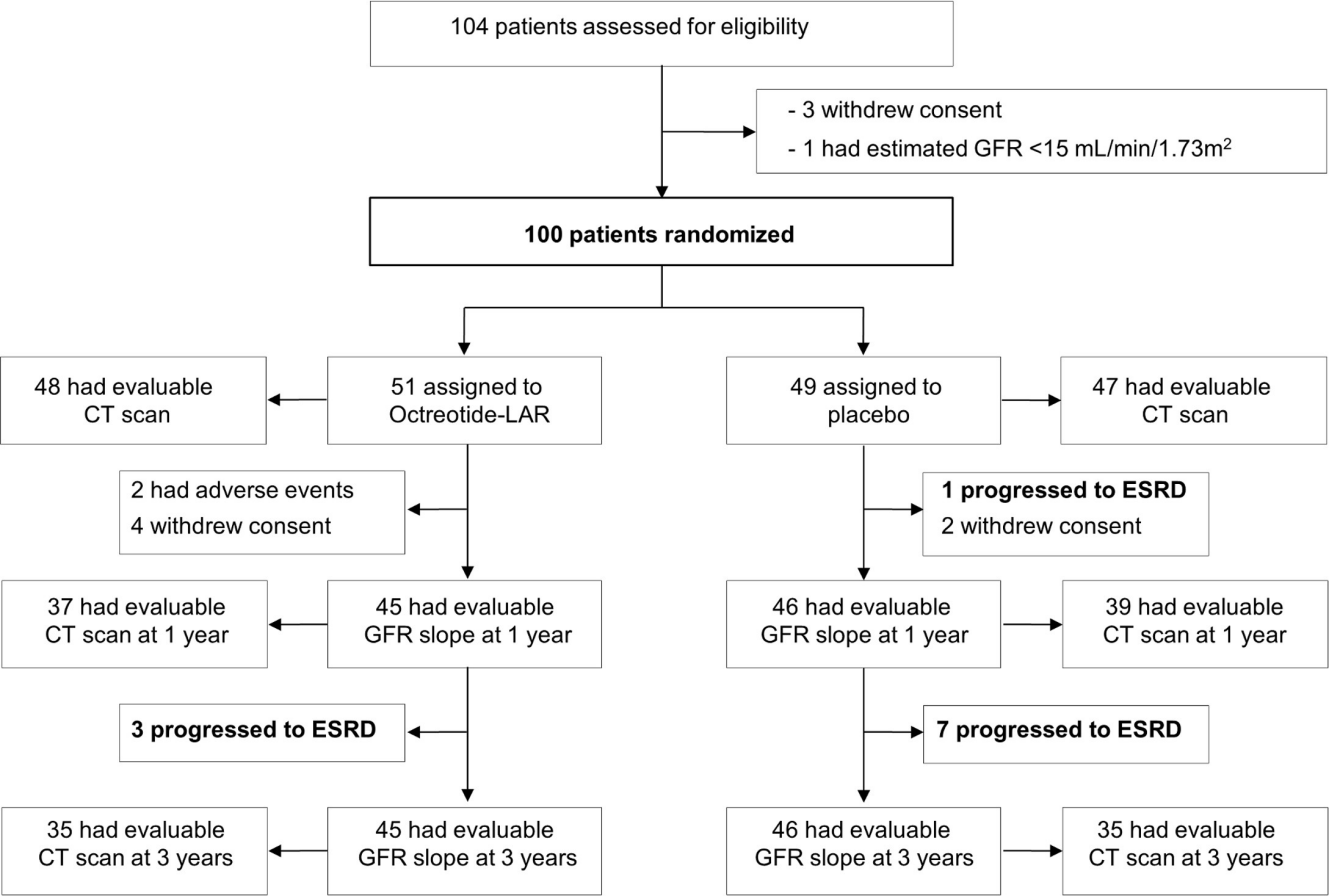
Trial profile. CT, computed tomography; ESRD, end-stage renal disease; GFR, glomerular filtration rate; octreotide-LAR, octreotide long-acting release; TKV, total kidney volume.

Of 104 assessed patients, 3 withdrew consent and 1 had eGFR < 15 ml/min/1.73 m^2^. Thus, 100 patients were randomized from October 11, 2011, to March 20, 2014 (51 to octreotide-LAR and 49 to placebo), and followed for a median (IQR) of 36 (24 to 37) months (Fig 1). Forty-eight patients allocated to octreotide-LAR and 47 allocated to placebo had evaluable TKV at baseline. After randomization, 4 patients on octreotide-LAR and 2 on placebo withdrew consent, 2 on octreotide-LAR left the study because of adverse events, and 1 on placebo progressed to ESRD. At 1 year, 45 patients on octreotide-LAR and 46 on placebo were available for GFR slope analyses; 37 on octreotide-LAR and 39 on placebo also had CT scan data evaluable for TKV analyses. After the first year, 3 patients on octreotide-LAR and 7 on placebo progressed to ESRD. All patients in the study at 1 year also had GFR slope data evaluable for analyses at the 3-year evaluation. In each group, 35 patients also had CT scan data for TKV analyses (Fig 1). All patients received all planned doses of octreotide-LAR or placebo from randomization to final visit. Thus, compliance to treatment was 100%.

### Baseline characteristics

Baseline characteristics—including distribution of imaging classes [26]—of patients randomized to the 2 treatment arms were similar in the study group considered as a whole and in the 37 patients with CKD stage 3b and 63 patients with CKD stage 4 considered separately (Tables 1 and S1; S1 Fig). Independent of treatment allocation, median (IQR) urinary protein excretion was significantly higher in the CKD stage 4 group than in the CKD stage 3b group (355.0 [170.0 to 714.5] versus 180.0 [110.0 to 320.0] mg/24 h, *p =* 0.008; S2 Table). Concomitant medications were distributed similarly between treatment groups (S3 Table).

**Table 1:** Demography and baseline clinical and laboratory characteristics and imaging classification of participants randomized to octreotide-LAR or placebo in the study group considered as a whole (overall) and according to CKD stage 3b and 4.

Characteristic	Overall		CKD stage 3b		CKD stage 4	
	Octreotide-LAR (*n =* 51)	Placebo (*n =* 49)	Octreotide-LAR (*n =* 20)	Placebo (*n =* 17)	Octreotide-LAR (*n =* 31)	Placebo (*n =* 32)
Sex (male/female)	31/20	26/23	11/9	9/8	20/11	17/15
Age (y)	48.7 ± 8.9	50.0 ± 9.3	50.3 ± 9.0	48.9 ± 9.1	47.7 ± 9.1	49.4 ± 8.5
Height (cm)	172.3 ± 9.8	170.7 ± 10.8	172.1 ± 11.1	174.3 ± 9.7	172.1 ± 9.0	169.0 ± 11.3
Weight (kg)	77.2 ± 14.6	76.4 ± 14.1	74.6 ± 14.6	77.7 ± 14.7	78.6 ± 14.8	75.8 ± 14.0
Blood pressure (mm Hg)						
Systolic	134.9 ± 15.4	132.3 ± 13.2	137.8 ± 16.8	132.2 ± 13.2	133.7 ± 14.0	132.7 ± 13.4
Diastolic	81.8 ± 9.3	83.1 ± 8.4	84.5 ± 8.4	84.5 ± 8.7	80.9 ± 9.1	83.1 ± 7.2
Mean	99.5 ± 10.3	99.5 ± 8.9	102.3 ± 11.0	100.4 ± 9.7	98.5 ± 9.1	99.6 ± 7.8
Total cholesterol (mmol/l)	5.0 ± 1.0	4.8 ± 1.0	5.3 ± 1.0	5.2 ± 1.1	4.8 ± 0.9	4.7 ± 0.9
LDL cholesterol (mmol/l)	3.0 ± 0.8	2.9 ± 0.9	3.2 ± 0.8	3.1 ± 0.9	2.9 ± 0.8	2.8 ± 0.9
Triglycerides (mmol/l)	1.3 ± 0.7	1.3 ± 0.6	1.1 ± 0.5	1.3 ± 0.8	1.5 ± 0.7	1.3 ± 0.5
Serum glucose (mmol/l)	5.0 ± 0.6	4.9 ± 0.7	4.9 ± 0.6	5.0 ± 0.7	5.0 ± 0.6	4.8 ± 0.7
Serum phosphorus (mmol/l)	1.2 ± 0.2	1.2 ± 0.2	1.1 ± 0.2	1.2 ± 0.1	1.2 ± 0.2	1.3 ± 0.2
Serum calcium (mmol/l)	2.3 ± 0.1	2.3 ± 0.2	2.3 ± 0.1	2.4 ± 0.2	2.3 ± 0.1	2.3 ± 0.2
Hemoglobin (g/l)	124 ± 15	121 ± 12	131 ± 17	125 ± 12	119 ± 12	119 ± 12
Serum albumin (g/l)	41 ± 4	41 ± 4	40 ± 4	40 ± 5	41 ± 5	41 ± 4
Serum creatinine (μmol/l)	229.8 ± 79.6	238.7 ± 79.6	168.0 ± 26.5	168.0 ± 26.5	265.2 ± 79.6	274.0 ± 70.7
GFR (ml/min/1.73 m^2^)*	31.5 [25.6 to 36.6]	30.9 [21.6 to 37.4]	36.4 [31.2 to 37.9]	37.9 [34.2 to 45.6]	27.8 [24.6 to 34.1]	26.3 [19.9 to 31.7]
eGFR (ml/min/1.73 m^2^)^†^	27.9 [23.5 to 32.2]	25.8 [19.5 to 33.2]	33.6 [31.3 to 38.9]	35.6 [32.3 to 38.4]	24.6 [20.6 to 27.3]	21.8 [18.0 to 25.8]
Urinary proteins (mg/24 h)	268 [135 to 805]	260 [130 to 460]	180 [130 to 330]	160 [90 to 300]	390 [150 to 880]	320 [180 to 570]
Urinary albumin (μg/ml)	50.7 [21.0 to 118.1]	28.3[12.8 to 96.2]	40.8 [31.7 to 129.3]	21.4 [11.1 to 106.9]	52.9 [20.9 to 118.1]	28.3 [12.9 to 61.3]
Urinary albumin-to-creatinine ratio (mg/g)	77.3 [35.9 to 225.9]	45.4 [25.5 to 181.9]	66.8 [40.6 to 272.7]	32.7 [15.7 to 170.6]	97.2 [35.9 to 192.0]	51.1 [30.4 to 194.8]
TKV (ml)	2,338 [1,967 to 3,807]	2,591 [1,959 to 3,835]	2,006 [1,788 to 2,643]	2,809 [2,059 to 3,587]	2,667 [2,026 to 4,060]	2,567 [1,657 to 4,078]
htTKV (ml/m)	1,344 [1,129 to 2,098]	1,528 [1,155 to 2,291]	1,212 [1,088 to 1,497]	1,614 [1,218 to 1,918]	1,623 [1,198 to 2,264]	1,528 [1,082 to 2,534]
Imaging classification						
1A	2 (3.9)	1 (2.0)	1 (5.0)	0 (0.0)	1 (3.2)	1 (3.1)
1B	2 (3.9)	6 (12.2)	1 (5.0)	2 (11.8)	1 (3.2)	4 (12.5)
1C	16 (31.4)	13 (26.5)	9 (45.0)	5 (29.4)	7 (22.6)	8 (25.0)
1D	13 (25.5)	13 (26.5)	5 (25.0)	3 (17.7)	8 (25.8)	10 (31.3)
1E	15 (29.4)	14 (28.6)	3 (15.0)	5 (29.4)	12 (38.7)	9 (28.1)
Not evaluable	3 (5.9)	2 (4.1)	1 (5.0)	2 (11.8)	2 (6.5)	0 (0.0)

Data are mean ± SD, median [IQR], or *n* (percent).

*Measured by iohexol plasma clearance.

^†^Estimated by the 4-variable equation from Modification of Diet in Renal Disease Study. No difference between treatment groups was significant within the study group considered as a whole, nor in the 2 subgroups of patients with CKD stage 3b or 4 considered separately.

CKD, chronic kidney disease; eGFR, estimated glomerular filtration rate; GFR, glomerular filtration rate; htTKV, height-adjusted total kidney volume; octreotide-LAR, octreotide long-acting release; TKV, total kidney volume.

### Primary outcomes

**Fig 2:**
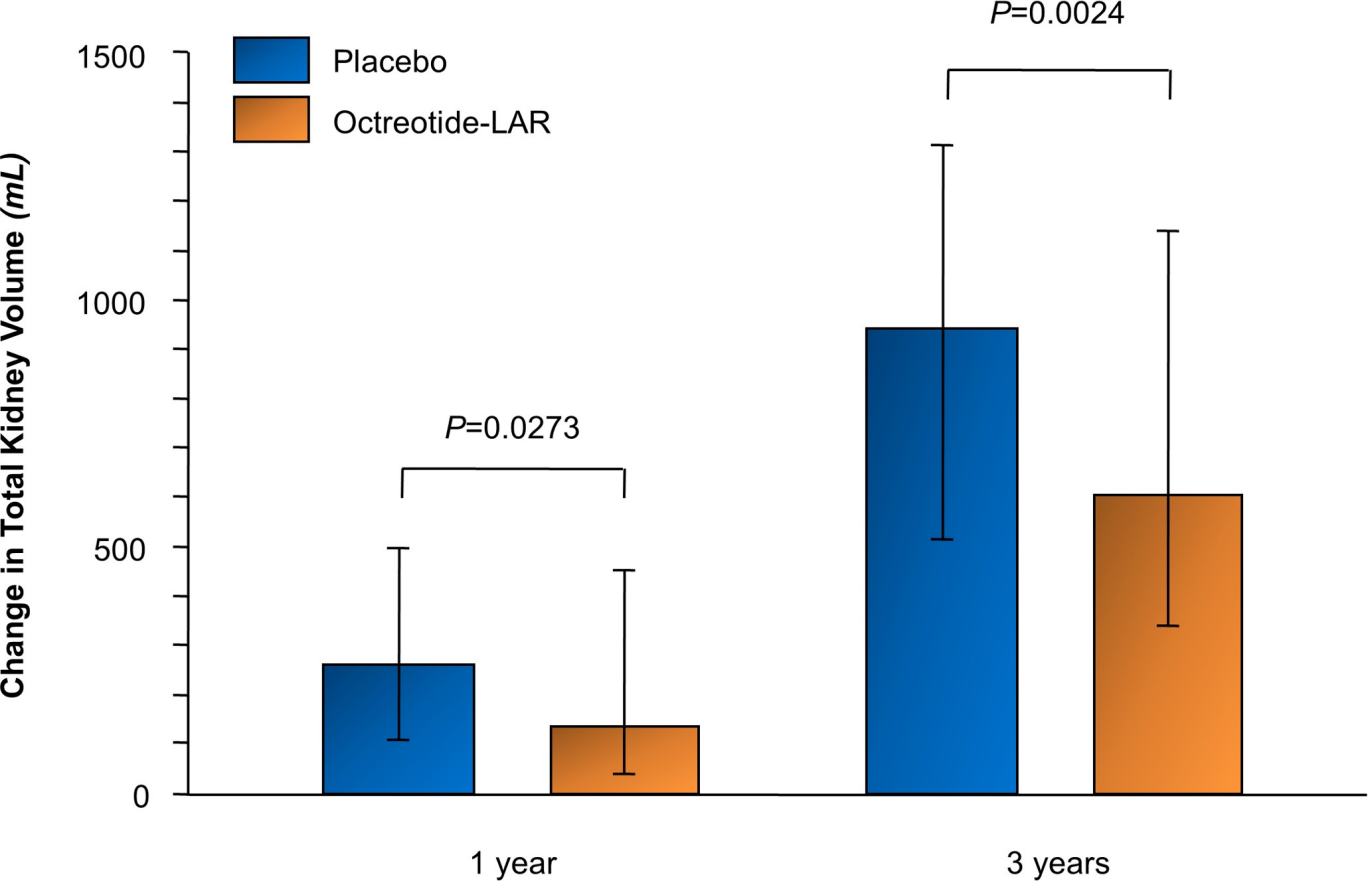
Absolute changes in total kidney volume from baseline to 1-year and 3-year follow-up. Absolute changes in total kidney volume from baseline to 1-year (primary short-term outcome) and 3-year follow-up in patients randomized to either placebo or octreotide-LAR. Data are reported as median and interquartile range. *P* values from non-parametric ANCOVA adjusted for age, sex and baseline total kidney volume. Analysis performed including all non-missing data. octreotide-LAR, octreotide long-acting release.

Median (IQR) TKV increased less with octreotide-LAR than with placebo at 1 year (135.5 [40.4 to 453.1] versus 257.7 [112.6 to 497.7] ml) and 3 years (604.2 [339.1 to 1,145.1] versus 939.1 [515.5 to 1,318.0] ml). Compared to placebo, octreotide-LAR reduced median (95% CI) TKV growth from baseline by 96.8 (10.8 to 182.7) ml at 1 year (*p =* 0.027) and 422.6 (150.3 to 695.0) ml at 3 years (*p =* 0.002) (Fig 2; Table 2). Similar results were obtained for absolute increases in htTKV (Table 2). Median (IQR) percentage increase in TKV was significantly less with octreotide-LAR than placebo at 1 year (5.2% [1.6% to 10.2%] versus 8.8% [5.2% to 13.7%], *p =* 0.036) and numerically lower at 3 years (29.9% [13.0% to 41.8%] versus 37.1% [23.2% to 54.6%], *p =* 0.091).

**Table 2:** TKV and htTKV at baseline, 1-year follow-up (primary short-term outcome), and 3-year follow-up in the study group as a whole (overall) and in the 2 subgroups with CKD stage 3b and 4 considered separately, according to treatment with octreotide-LAR or placebo.

Outcome	Measure	Octreotide-LAR			Placebo			*p-*Value
		Baseline	1 year	3 years	Baseline	1 year	3 years	
**Overall**								
TKV	Median [IQR] (ml)	2,338.9 [1,967.6–3,807.4]	2,513.3 [2,023.6–3,923.5]	3,043.9 [2,337.3–5,470.6]	2,591.0 [1,959.3–3,835.7]	2,935.1 [2,197.1–4,094.4]	3,613.8 [2,584.1–4,866.8]	
	Absolute change (ml)	—	135.5 [40.4–453.1]	604.2 [339.1–1,145.1]	—	257.7 [112.6–497.7]	939.1 [515.5–1,318.0]	0.027* 0.002^†^
htTKV	Median [IQR] (ml/m)	1,344.3 [1,129.0–2,097.7]	1,528.1 [1,237.1–2,281.1]	1,744.9 [1,397.5–3,180.6]	1,527.7 [1,154.6–2,290.9]	1,769.5 [1,258.3–2,488.3]	2,155.0 [1,429.5–2,791.4]	
	Absolute change (ml/m)	—	80.7 [24.8–258.9]	377.6 [201.8–642.4]	—	155.3 [66.1–286.0]	551.3 [310.7–795.7]	0.020* 0.025^†^
**CKD stage 3b**								
TKV	Median [IQR] (ml)	2,005.6 [1,787.7–2,642.8]	2,023.6 [1,754.3–2,697.0]	2,575.2 [1,962.3–3,574.3]	2,808.0 [2,059.0–3,586.5]	2,887.7 [2,157.9–3,767.5]	3,500.9 [2,796.3–4,705.3]	
	Absolute change (ml)	—	49.5 [−4.4 to 103.2]	481.5 [215.6–916.4]	—	194.9 [94.4–309.7]	937.5 [427.4–1,313.3]	0.501* 0.297^†^
htTKV	Median [IQR] (ml/m)	1,212.3 [1,088.0–1,497.3]	1,237.1 [1,076.6–1,528.1]	1,433.1 [1,226.2–1,861.6]	1,614.2 [1,218.3–1,917.9]	1,673.5 [1,221.0–2,050.5]	2,120.2 [1,590.6–2,645.6]	
	Absolute change (ml/m)	—	27.5 [−2.6 to 63.7]	277.7 [136.6–503.5]	—	115.2 [54.4–171.8]	546.9 [252.9–702.3]	0.348* 0.272^†^
**CKD stage 4**								
TKV	Median [IQR] (ml)	2,667.0 [2,026.4–4,059.6]	3,294.0 [2,467.5–4,939.8]	4,156.1 [2,828.2–6,074.2]	2,566.5 [1,657.3–4,077.8]	2,953.9 [2,187.1–4,404.3]	3,953.0 [2,213.7–4,866.8]	
	Absolute change (ml)	—	335.9 [87.4–487.7]	992.1 [544.4–1,914.2]	—	324.4 [118.6–568.6]	1,083.9 [515.5–2,331.0]	0.060* 0.079^†^
htTKV	Median [IQR] (ml/m)	1,622.7 [1,197.9–2,263.9]	1,904.1 [1,410.0–2,729.2]	2,353.7 [1,684.6–3,391.0]	1,527.7 [1,081.7–1,328.0]	1,779.5 [1,328.0–2,575.1]	2,401.9 [1,429.3–2,932.6]	
	Absolute change (ml/m)	—	195.3 [49.7–275.8]	561.0 [320.8–1,073.2]	—	187.7 [71.0–321.2]	595.7 [339.1–1,363.2]	0.057* 0.079^†^

Data are median [IQR]. Comparisons performed by non-parametric ANCOVA adjusted for baseline total kidney volume, age, and sex.

*Octreotide-LAR versus placebo at 1 year.

^†^Octreotide-LAR versus placebo at 3 years.

CKD, chronic kidney disease; htTKV, height-adjusted total kidney volume; octreotide-LAR, octreotide long-acting release; TKV, total kidney volume.

Compared with baseline, measured GFR [16] decreased by 11.3% in the octreotide-LAR group and by 7.0% in the placebo group after 6 months of treatment (Table 3). Thereafter, the reduction in the median (95% CI) rate of GFR decline (0.56 [−0.63 to 1.75] ml/min/1.73 m^2^ per year) with octreotide-LAR compared to placebo was not significant (*p =* 0.295) (Table 3 and S2 Fig show individual values for GFR decline over 3 years, GFR reduction from baseline to 6 months, and chronic GFR decline from 6 months to study end).

Sensitivity analyses restricted to the 70 patients without concomitant diabetes and without proteinuria showed that, compared to placebo, octreotide-LAR reduced median (95% CI) TKV growth from baseline by 90.8 (−4.7 to 186.4) ml at 1 year (*p =* 0.062) and 410.1 (105.8 to 714.3) ml at 3 years (*p =* 0.008). The difference in the median (95% CI) GFR slope (0.88 [−0.52 to 2.27] ml/min/1.73 m^2^ per year) between the octreotide-LAR and placebo groups was not significant (*p =* 0.181) (S4 Table).

**Table 3:** Measured GFR at baseline, 6 months, 1 year, 2 years, and 3 years (primary long-term outcome) in the study group as a whole (overall), and in the 2 subgroups with CKD stage 3b and 4 considered separately, according to treatment with octreotide-LAR or placebo.

Outcome	Octreotide-LAR					Placebo				
	Baseline (*n =* 50)	6 months (*n =* 46)	1 year (*n =* 44)	2 years (*n =* 40)	3 years (*n =* 35)	Baseline (*n =* 47)	6 months (*n =* 47)	1 year (*n =* 46)	2 years (*n =* 40)	3 years (*n =* 35)
**Overall**										
Actual value*	31.5 [25.6 to 36.6]	27.0 [22.2 to 32.3]	25.3 [19.4 to 29.9]	22.5 [17.2 to 26.9]	19.8 [15.5 to 23.7]	30.9 [21.6 to 37.4]	26.3 [20.9 to 34.8]	24.4 [20.9 to 34.8]	22.2 [17.9 to 28.5]	18.1 [14.7 to 26.7]
Total slope^†^ 0–3 years					−4.26 [−6.2 to −3.0]					−4.19 [−5.5 to −1.7]
Chronic slope^†^ 6 months–3 years					−3.76 [−5.1 to −2.4]					−3.97 [−5.9 to −2.0]
**CKD stage 3b**										
Actual value*	36.4 [31.2 to 37.9]	32.0 [27.0 to 35.6]	29.2 [26.1 to 34.8]	26.6 [22.0 to 29.0]	22.5 [18.3 to 26.7]	37.9 [34.2 to 45.6]	35.6 [26.0 to 38.9]	33.4 [29.9 to 38.0]	28.7 [23.7 to 34.9]	26.5 [18.9 to 32.9]
Total slope^†^ 0–3 years					−5.11 [−6.2 to −3.2]					−3.18 [−6.3 to −1.5]
Chronic slope^†^ 6 months–3 years					−4.24 [−5.9 to −2.5]					−3.49 [−6.2 to −1.1]
**CKD stage 4**										
Actual value*	27.8 [24.6 to 34.1]	23.1 [18.8 to 28.2]	21.3 [17.7 to 25.7]	18.8 [14.9 to 25.0]	16.8 [13.6 to 20.3]	26.3 [19.9 to 31.7]	24.5 [19.6 to 27.4]	21.8 [19.2 to 25.4]	18.7 [15.3 to 23.6]	15.1 [12.4 to 17.8]
Total slope^†^ 0–3 years					−4.16 [−6.3 to −3.0]					−4.37 [−5.2 to −2.3]
Chronic slope^†^ 6 months–3 years					−3.66 [−4.8 to −2.1]					−4.77 [−5.7 to −2.4]

Data are median [IQR].

*ml/min/1.73 m^2^.

^†^ml/min/1.73 m^2^ per year.

CKD, chronic kidney disease; GFR, glomerular filtration rate; octreotide-LAR, octreotide long-acting release.

### Secondary outcomes

**Fig 3:**
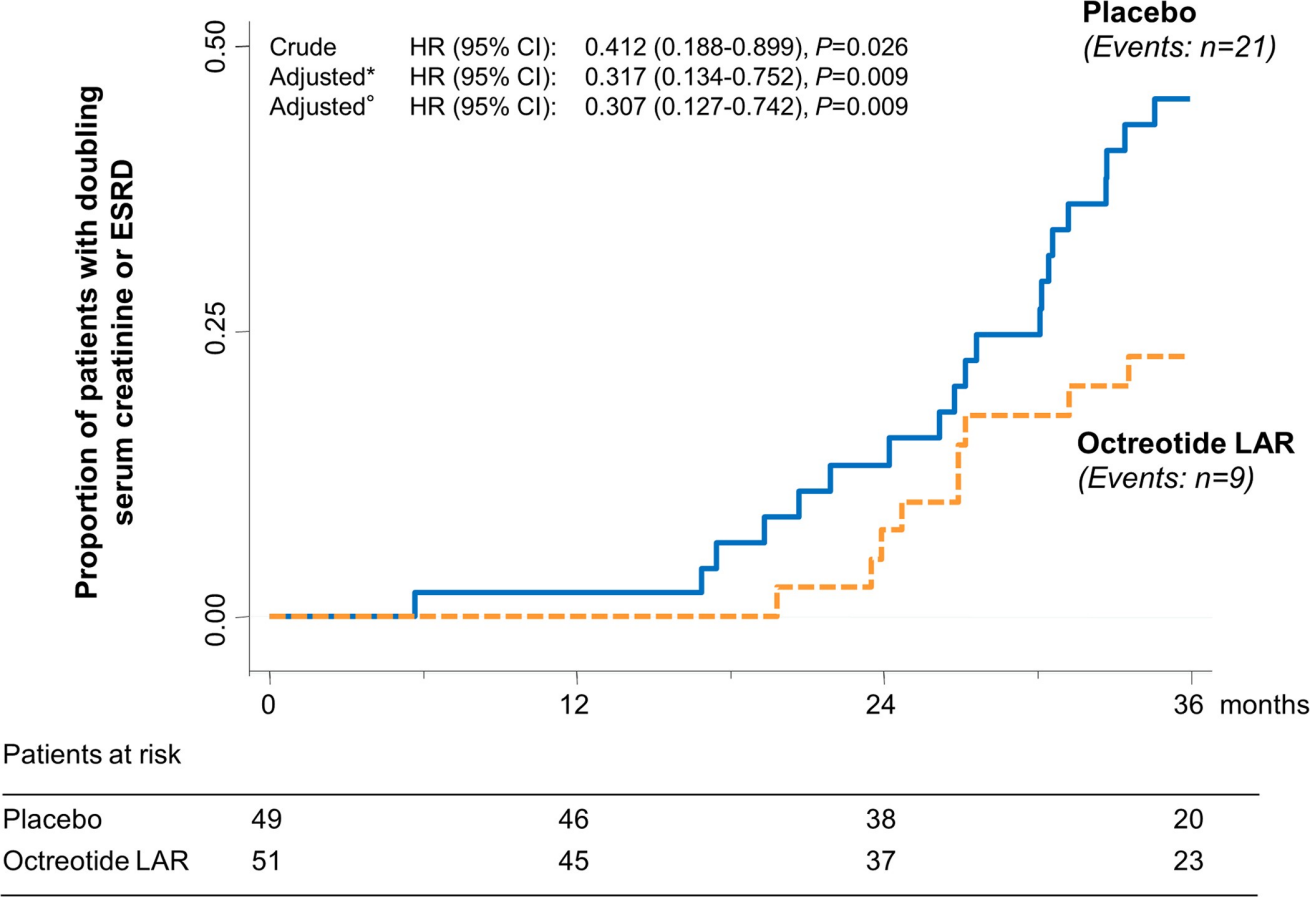
Kaplan–Meier curves for the secondary composite endpoint of doubling of serum creatinine or ESRD. Kaplan–Meier curves show the proportion of patients who reached the composite endpoint of doubling of serum creatinine or ESRD in the placebo and octreotide-LAR groups during the 3-year study period. *Adjusted by age, sex, and baseline serum creatinine.°Adjusted by age, sex, and baseline serum creatinine and total kidney volume. ESRD, end-stage renal disease; HR, hazard ratio; octreotide-LAR, octreotide long-acting release.

**Fig 4:**
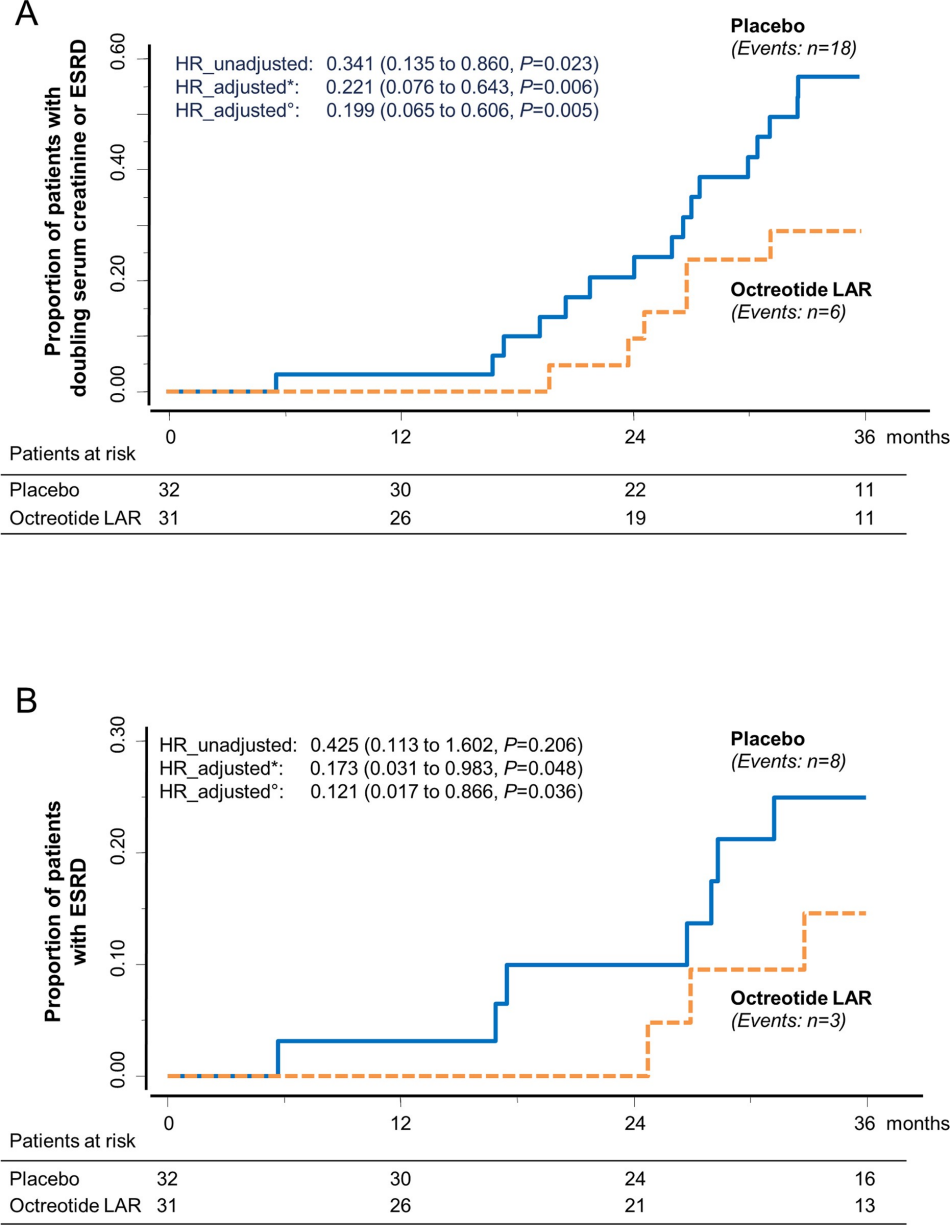
Kaplan–Meier curves for the secondary composite endpoint of doubling of serum creatinine or ESRD and for the single endpoint of ESRD in patients with CKD stage 4. Kaplan–Meier curves show the proportion of patients with CKD stage 4 who reached (A) the composite endpoint of doubling of serum creatinine or ESRD or (B) ESRD considered as a single endpoint (secondary outcomes) in the placebo and octreotide-LAR groups during the 3-year study period. *Adjusted by age, sex, and baseline serum creatinine.°Adjusted by age, sex, and baseline serum creatinine and total kidney volume. CKD, chronic kidney disease; ESRD, end-stage renal disease; HR, hazard ratio; octreotide LAR, octreotide long-acting release.

During the study, 9 of 51 patients (17.6%) on octreotide-LAR progressed to the composite endpoint of doubling of serum creatinine or ESRD compared to 21 of 49 (42.9%) on placebo (crude hazard ratio [HR] 0.412 [95% CI 0.188 to 0.899], *p =* 0.026) (Fig 3). Treatment effect was significant even when the analyses were adjusted for age, sex, and baseline serum creatinine and TKV (adjusted HR 0.307 [95% CI 0.127 to 0.742], *p =* 0.009). Three patients of 51 (5.9%) on octreotide-LAR progressed to ESRD considered as a single endpoint compared to 8 of 49 patients (16.3%) on placebo (crude HR 0.376 [95% CI 0.100 to 1.418], *p =* 0.149). Four patients (95% CI 1 to 7) needed to be treated to prevent 1 composite endpoint, and 10 (95% CI −2 to 21) to prevent 1 ESRD event considered as single endpoint, within the 3-year analysis period. Analyses that were not prespecified showed that all ESRD events were observed in the subgroup of 63 patients with CKD stage 4. In this subgroup, 6 of the 31 patients on octreotide-LAR (19.4%) progressed to the combined endpoint, compared to 18 of the 32 on placebo (56.3%). The difference was significant (crude HR [95% CI]: 0.341 [0.135 to 0.860], *p =* 0.023) even after adjusting for age, sex, and baseline serum creatinine and TKV (adjusted HR [95% CI]: 0.199 [0.065 to 0.606], *p =* 0.005) (Fig 4A). Three patients of 31 on octreotide-LAR (9.7%) progressed to ESRD compared to 8 of 32 (25.0%) on placebo, an effect that was significant after adjusting for age, sex, and baseline serum creatinine and TKV (adjusted HR [95% CI]: 0.121 [0.017 to 0.866], *p =* 0.036) (Fig 4B).

### Other outcomes

At baseline and at each study visit up to study end, systolic and diastolic blood pressure (S3 Fig) and HbA1c serum level were similar between groups. The median (IQR) urinary protein excretion rate increased significantly, from 260 (130 to 460) mg/24 h at baseline to 420 (160 to 710) mg/24 h over the whole follow-up period (*p* < 0.001), in the placebo group, but did not change appreciably in the octreotide-LAR group (S2 Table). Data on eGFR and other clinical and laboratory variables at baseline and follow-up are shown in S5 and S6 Tables. Notably, throughout the whole observation period, urinary osmolality was slightly lower in patients randomized to octreotide-LAR compared to those allocated to placebo (S6 Table). The distribution of blood-pressure-lowering medications and all other considered treatments was similar between groups at baseline and follow-up, with the exception of calcitriol treatment and sodium bicarbonate supplementation, which over the whole follow-up period were more frequent in patients on octreotide-LAR than in those on placebo (S3 Table).

In patients with CKD stage 4, at 3 years of treatment, both TKV and htTKV absolute change from baseline were numerically, though not significantly, lower in the octreotide-LAR than the placebo group. Between baseline and 6 months, median (IQR) measured GFR decreased more with octreotide-LAR than with placebo (−5.6 [−7.7 to −1.7] versus −1.2 [−4.3 to +1.7] ml/min/1.73 m^2^, *p =* 0.03), whereas between 6 months and study end, median (IQR) chronic GFR decline tended to be slower with octreotide-LAR than with placebo (Table 3). Individual data are shown in S2 Fig. In the same cohort, urinary protein excretion increased significantly, from 320 (180 to 570) mg/24 h at baseline to 508 (325 to 750) mg/24 h over the whole follow-up period (*p =* 0.002), in the placebo group, but it did not change appreciably in the octreotide-LAR group (S2 Table).

Overall, 6 of the 37 patients with CKD stage 3b progressed to doubling of serum creatinine: 3 on octreotide-LAR and 3 on placebo. Data on TKV changes at 1 and 3 years and GFR slopes in this subgroup are shown in Tables 2 and 3, respectively.

### Safety

Twelve of 51 (23.5%) participants in the octreotide-LAR group and 11 of 49 (22.4%) in the placebo group had at least 1 serious adverse event (*p =* 0.898). Overall, distribution of serious (Table 4) and non-serious (S7 Table) adverse events was similar between groups. However, 2 of 51 patients (3.9%) on octreotide-LAR compared to 9 of 49 (18.4%) on placebo (*p =* 0.021) had a serious (1 versus 3; Table 4) or non-serious (1 versus 6; S7 Table) renal cyst rupture or infection. These events were considered serious or non-serious according to standard criteria detailed in the study protocol.

**Table 4:** Number of patients with serious adverse events.

Serious adverse event	Octreotide-LAR (*n =* 51)	Placebo (*n =* 49)
Overall	12 (23.5%)	11 (22.4%)
Pulmonary embolism	1 (2.0%)	0
Myocardial infarction	0	1 (2.0%)
Acute renal failure	2 (3.9%)	2 (4.1%)
Renal cyst rupture or infection	1 (2.0%)	3 (6.1%)
Urinary tract infection	0	1 (2.0%)
Acute pyelonephritis	0	1 (2.0%)
Ureteral obstruction due to lithiasis	0	1 (2.0%)
Sepsis due to *Klebsiella pneumoniae*	1 (2.0%)	0
Varicella	1 (2.0%)	0
Umbilical hernia	1 (2.0%)	0
Acute pancreatitis	1 (2.0%)	0
Biliary vomiting	1 (2.0%)	0
Abdominal pain	0	1 (2.0%)
Anemia	1 (2.0%)	1 (2.0%)
Fever	1 (2.0%)	0
Pancreatic enzyme elevation	0	1 (2.0%)
Hyperammonemia	1 (2.0%)	0
Acute retinal detachment	1 (2.0%)	0
Genitourinary prolapse	1 (2.0%)	0
Cystocele	1 (2.0%)	0
Menometrorrhagia	1 (2.0%)	0

Data are *n* (%).

octreotide-LAR, octreotide long-acting release.

Diarrhea, biliary sand, and cholelithiasis were more frequent in the octreotide-LAR group. In this group, diarrhea and other gastrointestinal symptoms recovered spontaneously within the first month of treatment. Biliary sand and cholelithiasis recovered with ursodeoxycholic acid treatment. In addition to renal cyst rupture or infection, other possibly disease-related events including back pain and hepatic cyst rupture appeared to be more frequent in the placebo group (S7 Table).

At 1 and 3 years, body weight and all blood variables were comparable between groups (S6 Table), with the exception of blood glucose concentration, which was higher in the octreotide-LAR group than in the placebo group at both time points. However, new-onset diabetes was not reported in a single patient. Twenty-four-hour urine output and urea, phosphate, and sodium excretion were similar between treatment groups (S6 Table).

No participant required treatment interruption or dose down-titration during the study.

## Discussion

In this study we found that 3-year treatment with octreotide-LAR did not appreciably affect GFR decline compared to placebo in 100 patients with later-stage (CKD stage 3b or 4) ADPKD. Active treatment, however, slowed kidney volume growth and progression to the combined endpoint of doubling of serum creatinine or ESRD, and prevented the urinary protein increase observed in controls randomized to placebo. Octreotide-LAR was well tolerated, and no patient required treatment interruption or even transient dose down-titration during the study. The overall incidence of serious and non-serious adverse events was similar between groups. Our present findings confirm and extend evidence from the ALADIN trial [14] that octreotide-LAR may slow kidney volume growth and renal function loss in ADPKD patients with normal or moderately reduced kidney function. Moreover, our study provides the novel information that a somatostatin analog may slow the progression to a hard clinical endpoint such as ESRD in patients affected by ADPKD. Only one-sixth of patients on octreotide-LAR progressed to the combined endpoint of ESRD or doubling of serum creatinine compared to two-fifths of those on placebo. This finding may have implications for healthcare providers since postponing or even preventing ESRD, in addition to preserving patient quality of life and physical function, also reduces the direct and indirect costs for chronic renal replacement therapy. Notably, only 4 patients needed to be treated to prevent 1 composite endpoint, and 10 to prevent 1 ESRD event considered as a single endpoint, during the 3-year follow-up.

Notably, all ESRD events were observed in patients with CKD stage 4, and the protective effect of octreotide-LAR against progression to the combined endpoint, or to ESRD considered as a single endpoint, was fully driven by treatment effect in this subgroup. In these patients, the reduction in event rates was associated with an acute GFR reduction at 6 months that conceivably reflected amelioration of compensatory glomerular hyperfiltration [11,27–29], a tendency (admittedly non-significant) toward slower chronic GFR decline, and a protective effect against the increase in proteinuria observed on placebo. Thus, in ALADIN 2 patients with CKD stage 4, octreotide-LAR reduced the incidence of ESRD with only marginal effects on chronic GFR decline, an effect that conceivably could be explained by the extremely high number of ESRD events, which increased the power of event-based analyses compared to the power of slope-based analyses. Altogether, these data converge to indicate that even in later pre-terminal stages of ADPKD, when kidney architecture is largely disrupted, octreotide-LAR may still exert a specific and clinically relevant protective effect against progression of the disease.

Another finding that merits further investigation is that nephroprotection appeared to be partially explained by mechanisms—additional to those related to slowed kidney volume growth—similar to those of renin angiotensin system inhibitors, such as amelioration of hyperfiltration [14] and reduction of proteinuria, effects that in this specific context could be mediated by inhibited growth hormone secretion and action [30] and, notably, are not associated with hyperkalemia. As observed in other proteinuric chronic nephropathies [31], these effects may protect residual functioning units from accelerated dysfunction and sclerosis. Thus, based on the above considerations, it is conceivable that proteinuria might be an additional risk factor for disease progression and a specific treatment target for octreotide-LAR in patients with ADPKD and CKD stage 4 [32].

Throughout the whole observation period, urinary osmolality was slightly lower in patients randomized to octreotide-LAR compared to those randomized to placebo. This finding is of potential interest because a retrospective analysis of the Modification of Diet in Renal Disease Study, including 139 patients with ADPKD and chronic kidney disease [33], and of the TEMPO 3:4 trial [34] identified low urinary osmolality as a risk factor for faster renal function loss independent of treatment allocation. Consistently, defective urinary concentration is more evident in patients with larger kidneys [34,35] and appears to worsen in parallel with the progression of cystic lesions and consequent reduction in the interstitial osmotic gradient. This process is associated with peripheral resistance to vasopressin and decreased V2R expression/function in the distal nephron [34,36]. Thus, evidence that ADPKD patients randomized to octreotide-LAR experienced slower kidney growth and delayed progression to ESRD compared to controls, in spite of lower urinary osmolality, further corroborates the working hypothesis that octreotide-LAR may have a renoprotective effect in patients with ADPKD.

Our present findings differ from those of the DIPAK 1 study [37], an open-label randomized clinical trial with blinded endpoint assessment that tested the renal effects of 2.5-year treatment with lanreotide, another somatostatin analog, in 309 patients with ADPKD who had an eGFR of 30 to 60 ml/min/1.73 m^2^. Unlike ALADIN 2, DIPAK 1 failed to detect any treatment effect on worsening of kidney function, defined as a 30% decrease of eGFR compared to baseline or start of dialysis. However, in ALADIN 2 all ESRD events were observed in patients with CKD stage 4, and the protective effect of octreotide-LAR against progression to ESRD considered as a single endpoint or in combination with doubling of serum creatinine from baseline was fully driven by the treatment effect in this subgroup. Exclusion of patients with CKD stage 4 may explain why only 5 (3 on lanreotide) of the 309 randomized patients (1.6%) progressed to ESRD during the DIPAK 1 study, compared to 11 of 63 patients with CKD stage 4 (17.5%) progressing to ESRD during the ALADIN 2 trial [37]. Thus, unlike ALADIN 2, DIPAK 1 was underpowered to detect a treatment effect on ESRD because of a markedly lower incidence of events in the study population. An additional and plausible, but fully speculative, explanation for the differing results of these two studies could be that, because of amelioration of glomerular hyperfiltration and proteinuria, somatostatin analogs are more renoprotective in patients with later-stage ADPKD than in those with less severe renal dysfunction, who may have less or no hyperfiltration or proteinuria. Alternatively, lanreotide could be just less effective than octreotide-LAR in preventing ADPKD progression to ESRD.

The hypothesis of different drug-specific effects is corroborated by the fact that we did not observe any episodes of hepatic cyst infection in our participants given octreotide-LAR. This is at variance with the increased risk for hepatic cyst infection reported during treatment with lanreotide in the DIPAK 1 study, especially in those with a previous history of hepatic cyst infection [38]. Notably, despite the more advanced stage of disease in ALADIN 2 compared to the ALADIN trial, the safety profile of octreotide-LAR did not differ [14]. Morning fasting blood glucose was significantly higher in the octreotide-LAR than placebo group, but serum HbA1c values were similar between groups throughout the whole study period. Thus, it is conceivable that treatment impaired fasting blood glucose without appreciably affecting average blood glucose levels throughout the day. Consistently, no case of new-onset diabetes was observed in the octreotide-LAR group. As expected, diarrhea was more frequent in the octreotide-LAR group. However, in the affected 15 patients, it recovered spontaneously within 1 month from randomization. Biliary sand or stones were detected by routine ultrasound evaluation in 8 otherwise asymptomatic patients on octreotide-LAR, versus none on placebo, and dissolved in all cases with ursodeoxycholic acid supplementation. Adverse events that were most likely related to the disease, including renal cyst rupture or infection (which was serious in 4 cases), were more frequent in the placebo arm.

Our present data confirm the good safety profile of octreotide-LAR reported in the ALADIN trial [14], in a pilot safety study [11], and in a small pilot trial [39]. However, these findings must be taken with caution since they were obtained by relatively small studies that, combined with the ALADIN 2 trial, included a total of only 131 patients with ADPKD who were exposed to octreotide-LAR for a relative short period, ranging from a minimum of 6 to a maximum of 36 months.

On the other hand, octreotide-LAR has been used for years in thousands of patients for the treatment of acromegaly [40] and neuroendocrine tumors [41], and no major worrisome signal has emerged so far. Independent of the above considerations, however, data from larger series of patients with longer exposure to treatment are needed to better establish the risk/benefit profile of octreotide-LAR in the specific context of ADPKD.

Major strengths of this technically challenging study were the measurement of TKV and GFR by gold standard techniques and the centralized assessment of data by investigators with specific expertise. In particular, the use of CT scans with manual contouring to evaluate TKV has been validated in several studies [11,17,42], and comparative analyses between CT and magnetic resonance (MR) images in ADPKD patients [19] found that kidney volume reproducibility was higher for CT scans than for MR images for all considered methods, likely due to lower image quality on MR images, making kidney identification more operator-dependent. Study findings are unlikely to be explained by unbalanced distribution of risk factors for more severe outcome since baseline characteristics were much the same between groups. Moreover, sensitivity analyses restricted to the 70 patients without potentially confounding factors such as diabetes or proteinuria found that the treatment effect on the primary outcomes was very much the same in this subgroup as in the study group considered as a whole. Consistently, the treatment effect on kidney volume was significant even after adjusting for age, sex, and baseline kidney volume, and that on renal events after adjusting for age, sex, and baseline kidney volume and serum creatinine. Similar findings were observed when kidney volume data were corrected by patient height in order to adjust for the potential confounding effect of sex-related differences in kidney volume. Throughout the whole study period, blood pressure control and the distribution of antihypertensive drugs, including renin angiotensin system inhibitors, diuretics, and lipid-lowering agents, were similar between groups. Moreover, evidence that urinary output and 24-hour urea, phosphate, and sodium urinary excretion were almost the same in the 2 treatment groups reasonably excluded any appreciable role of potential confounding factors such as water, protein, and salt intake. Parametric multiple imputations by chain equations confirmed that study results were robust to missing data. The double-blind design was an additional strength. In particular, the decision to initiate chronic renal replacement therapy was made on the basis of standard clinical criteria by physicians who were blinded to both treatment assignment and GFR measurements [43], which enhanced the robustness of the results and their generalizability to everyday clinical practice. Finally, despite the highly labor-intensive design, and the relatively invasive treatment that required 2 intramuscular injections every 28 days, the study had a high retention rate of enrolled participants and full (100%) adherence to the study interventions.

Our study has a number of limitations. At randomization, GFR, eGFR, osmolality, and urinary protein excretion were slightly different between treatment groups. However, randomization (and even stratification in our study) in a clinical trial does not guarantee that patients allocated to the different treatment groups will be similar with respect to all characteristics evaluated at baseline, with potential differences among groups being attributable to chance [44]. Data on progression to doubling of serum creatinine or ESRD were obtained by analyses of a secondary efficacy outcome and need to be confirmed in larger trials. Furthermore, the data from patients with CKD stage 4 must be interpreted with caution, since they were generated by analyses in 63 patients that were not prespecified. Indeed, the sample size of our study was relatively small, and the possibility of a type I error cannot be definitely excluded. As prespecified in the study protocol, the use of contrast agents during CT scan acquisition to discriminate cyst volumes from intermediate or parenchyma volumes could be avoided when radiologists were concerned by the risk of nephrotoxicity in patients with renal insufficiency. Because of this cautious approach, data were obtained from a too-small number of patients, which did not allow us to perform informative analyses of treatment effect on different kidney compartments. Explorative analyses of endothelin and MCP-1 urinary excretion were not performed because of fund restriction.

Study findings may have implications for healthcare providers. Indeed, octreotide-LAR is an expensive medication. The identification of a subgroup—accounting for approximately 10% to 15% of patients with ADPKD [45]—who are at high risk of ESRD and at the same time may benefit the most from treatment may help increase the cost-effectiveness of octreotide-LAR for the prevention of ESRD (and related treatment costs) in this population. Our data may pave the way to large-scale randomized trials with progression to ESRD as the primary outcome, to definitively demonstrate the nephroprotective effect of octreotide-LAR even in patients with less advanced (CKD stage 3a and 3b) disease. This trial could also secondarily test the treatment effect on concomitant polycystic liver disease [21], cardiac function and morphology [46], and fatal and nonfatal major cardiovascular events.

In conclusion, in this internally funded, parallel-group, double-blind, placebo-controlled phase III trial, we assessed whether the renoprotective effect of the somatostatin analog octreotide-LAR shown in patients with early-stage ADPKD could be extended to patients with CKD stage 3b or 4. We found that 3-year treatment with octreotide-LAR did not appreciably affect GFR decline compared to placebo, although secondary analyses suggest that octreotide-LAR may help to postpone ESRD, particularly in patients with CKD stage 4. Further research, involving larger series of patients with longer exposure to treatment, is needed to investigate these signals further. The results of ALADIN 2 confirm and extend previous evidence showing that for adults with ADPKD octreotide-LAR is safe and may have a protective effect against kidney growth and GFR decline, and could be a novel disease-modifying therapy for patients with later-stage disease.

## Supporting information

S1 Appendix ALADIN 2 study organization. (DOCX)

S1 Checklist CONSORT checklist. (DOC)

S1 Fig Measured GFR, eGFR, and TKV at baseline in individual patients considered according to treatment with octreotide-LAR or placebo. (A) Measured GFR, (B) GFR estimated through the Modification of Diet in Renal Disease equation, and (C) TKV at baseline. Circles denote individual values, long lines are median values, and short lines are interquartile ranges. GFR, glomerular filtration rate; TKV, total kidney volume.
(TIF)

S2 Fig Total measured GFR slope throughout the observation period, short-term GFR change, and chronic measured GFR slope in individual patients according to treatment with octreotide-LAR or placebo in the study group as a whole and in the 2 subgroups with CKD stage 3b and 4 considered separately. Total measured GFR slope throughout the study period in the patient population as a whole (A), in patients with CKD stage 3b (B), and in patients with CKD stage 4 (C). Short-term measured GFR change from baseline to 6 months in the overall patient population (D), in patients with CKD stage 3b (E), and in patients with CKD stage 4 (F). Chronic measured GFR slope from 6 months to study end in the overall patient population (G), in patients with CKD stage 3b (H), and in patients with CKD stage 4 (I). Circles denote individual values, long lines are median values, and short lines are interquartile ranges. GFR, glomerular filtration rate; TKV, total kidney volume.
(TIF)

S3 Fig Systolic and diastolic blood pressure throughout the study period. Differences between treatment groups were never significant at any time point of the study. Values are mean ± SD.
(TIF)

S1 Protocol ALADIN 2 protocol. (PDF)

S1 Table Demography and baseline clinical and laboratory characteristics of participants with and without concomitant risk factors (diabetes mellitus or proteinuria > 1 g/24 h) according to randomization to octreotide-LAR or placebo. (DOCX)

S2 Table Twenty-four-hour urinary protein excretion at baseline and over the whole follow-up period according to randomization to octreotide-LAR or placebo in the study group as a whole (overall) and in the 2 subgroups with CKD stage 3b and 4 considered separately. (DOCX)

S3 Table Concomitant medications at baseline and during follow-up according to randomization to octreotide-LAR or placebo. (DOCX)

S4 Table TKV at baseline, 1-year follow-up, and 3-year follow-up, as well as total and chronic measured GFR slopes in patients without diabetes mellitus and without proteinuria > 1 g/24 h at baseline according to treatment with octreotide-LAR or placebo. (DOCX)

S5 Table eGFR at baseline, 6 months, 1 year, 2 years, and 3 years in the study group as a whole (overall), and in the 2 subgroups with CKD stage 3b and 4 considered separately, according to treatment with octreotide-LAR or placebo. (DOCX)

S6 Table Patients’ characteristics at baseline, 1 year, and 3 years according to randomization to octreotide-LAR or placebo treatment. (DOCX)

S7 Table Number (%) of patients with at least 1 non-serious adverse event in the study group as a whole (overall) and according to randomization to octreotide-LAR or placebo treatment. (DOCX)

S1 Text Supplementary methods. (DOCX)

S2 Text Statistical analysis plan. (PDF)

## Abbreviations

ADPKD: autosomal dominant polycystic kidney disease
cAMP: 3′,5′-cyclic adenosine monophosphate
CKD: chronic kidney disease
CT: computed tomography
eGFR: estimated glomerular filtration rate
ESRD: end-stage renal disease
GFR: glomerular filtration rate
HR: hazard ratio
htTKV: height-adjusted total kidney volume
MR: magnetic resonance
octreotide-LAR: octreotide long-acting release
TKV: total kidney volume
