# Supplementary material for: Octreotide-LAR in later-stage autosomal dominant polycystic kidney disease (ALADIN 2): A randomized, double-blind, placebo-controlled, multicenter trial
Source: PLoS Med. 2019 Apr 5;16(4):e1002777. doi: 10.1371/journal.pmed.1002777 (PMC6450618; doi:10.1371/journal.pmed.1002777)
Supplement: S1 Appendix — (DOCX) [file pmed.1002777.s001.docx]

**S1. Appendix ALADIN 2 Study Organization**

Members of the ALADIN 2 Study Organization were as follows (all in Italy unless otherwise noted): *Chief Investigator* — G. Remuzzi (Bergamo); *Study coordinator* — P. Ruggenenti (Bergamo); *Coordinating Centre* — Mario Negri Institute for Pharmacological Research, Clinical Research Center for Rare Diseases *Aldo e Cele Daccò*, Ranica (Bergamo); *Participating centres* — N. Perico, P. Ruggenenti, M. Trillini, B. Ruggiero, A. Gennarini, S. Rota, C. Ferrer Siles, S. Prandini, V. Lecchi, S. Gamba, G. Gherardi, A. Barletta, P. Fasolini, F. Sala, S. Sironi (Bergamo); A. Pisani, E. Riccio, M. Amicone, A. Mancini, F. Petrillo, G. Pisani, A. Ponsiglione, M. Imbriaco (Napoli); M. Dugo, C. Tuono, R. Stefanato, L. Cancian, G. Morana (Treviso); A. Granata, S. Spataro, R.V. Scarfia, M. Figuera (Agrigento); *Monitoring, Drug Distribution, and Pharmacovigilance*  (Mario Negri Institute) — N. Rubis, O. Diadei, A. Villa (Ranica); *Database and Data Validation* (Mario Negri Institute) — D. Martinetti, S. Carminati (Ranica); *Randomization* (Mario Negri Institute) - G.A. Giuliano (Ranica);  *Data Analysis* (Mario Negri Institute) — A. Perna, F. Peraro (Ranica); *Medical Imaging* (Mario Negri Institute) — A. Remuzzi, A. Caroli, K. Sharma, C. Aparicio, F. Cazzaniga, V. Finazzi (Ranica); *Laboratory Measurements* (Mario Negri Institute) — F. Gaspari, F. Carrara, S. Ferrari, N. Stucchi, A. Cannata (Ranica); *Regulatory Affairs* (Mario Negri Institute) — P. Boccardo, S. Peracchi (Ranica); *Independent Data Safety Monitoring Board* — E. Porrini, A. Jimenez Soza (Hospital Universitario de Canarias, Santa Cruz de Tenerife, Tenerife, Spain).
