# Supplementary figures and images for: Octreotide-LAR in later-stage autosomal dominant polycystic kidney disease (ALADIN 2): A randomized, double-blind, placebo-controlled, multicenter trial
Source: PLoS Med. 2019 Apr 5;16(4):e1002777. doi: 10.1371/journal.pmed.1002777 (PMC6450618; doi:10.1371/journal.pmed.1002777)

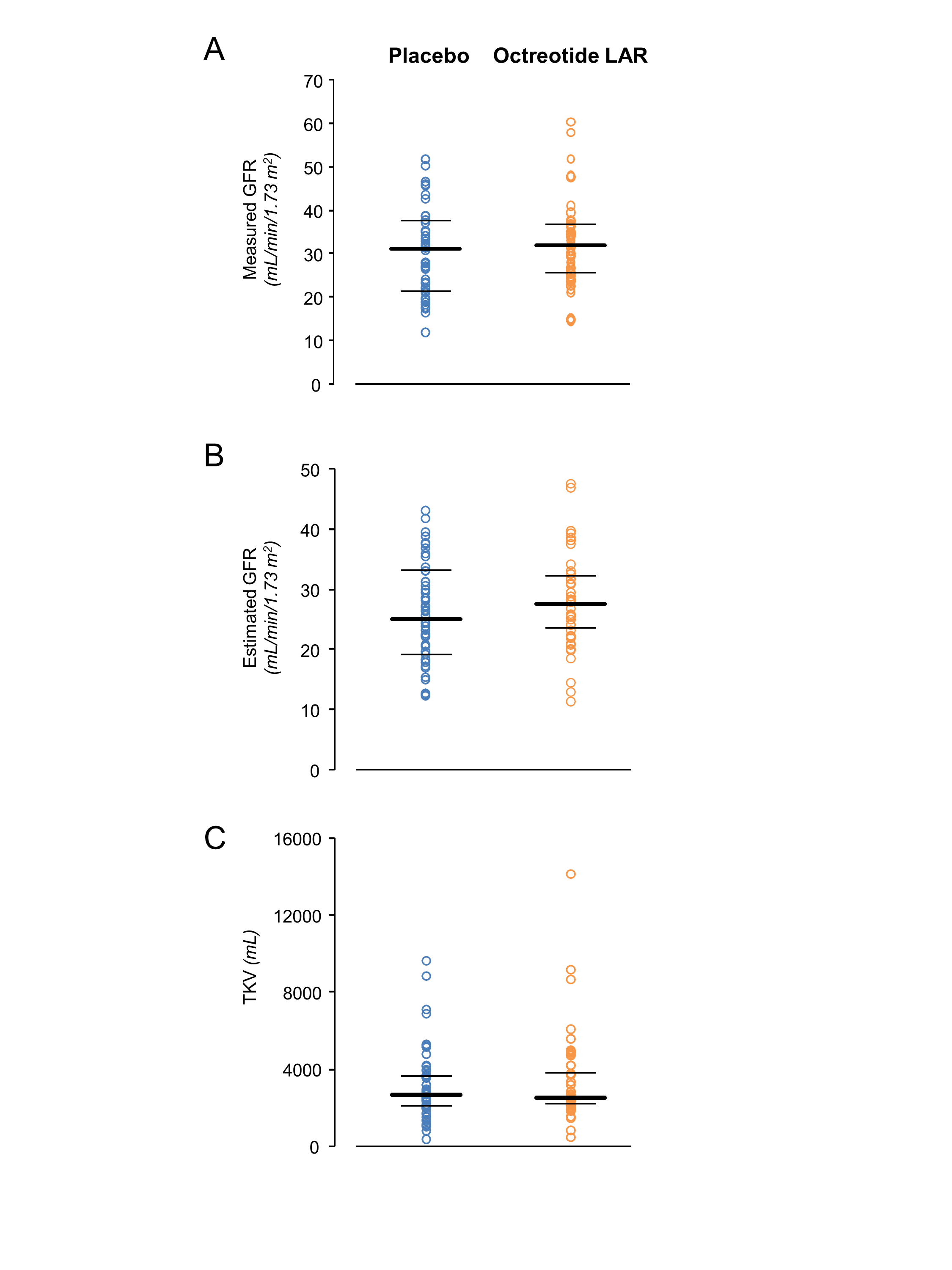

Supplement: S1 Fig — (A) Measured GFR, (B) GFR estimated through the Modification of Diet in Renal Disease equation, and (C) TKV at baseline. Circles denote individual values, long lines are median values, and short lines are interquartile ranges. GFR, glomerular filtration rate; TKV, total kidney volume. (TIF) [file pmed.1002777.s003.tif]

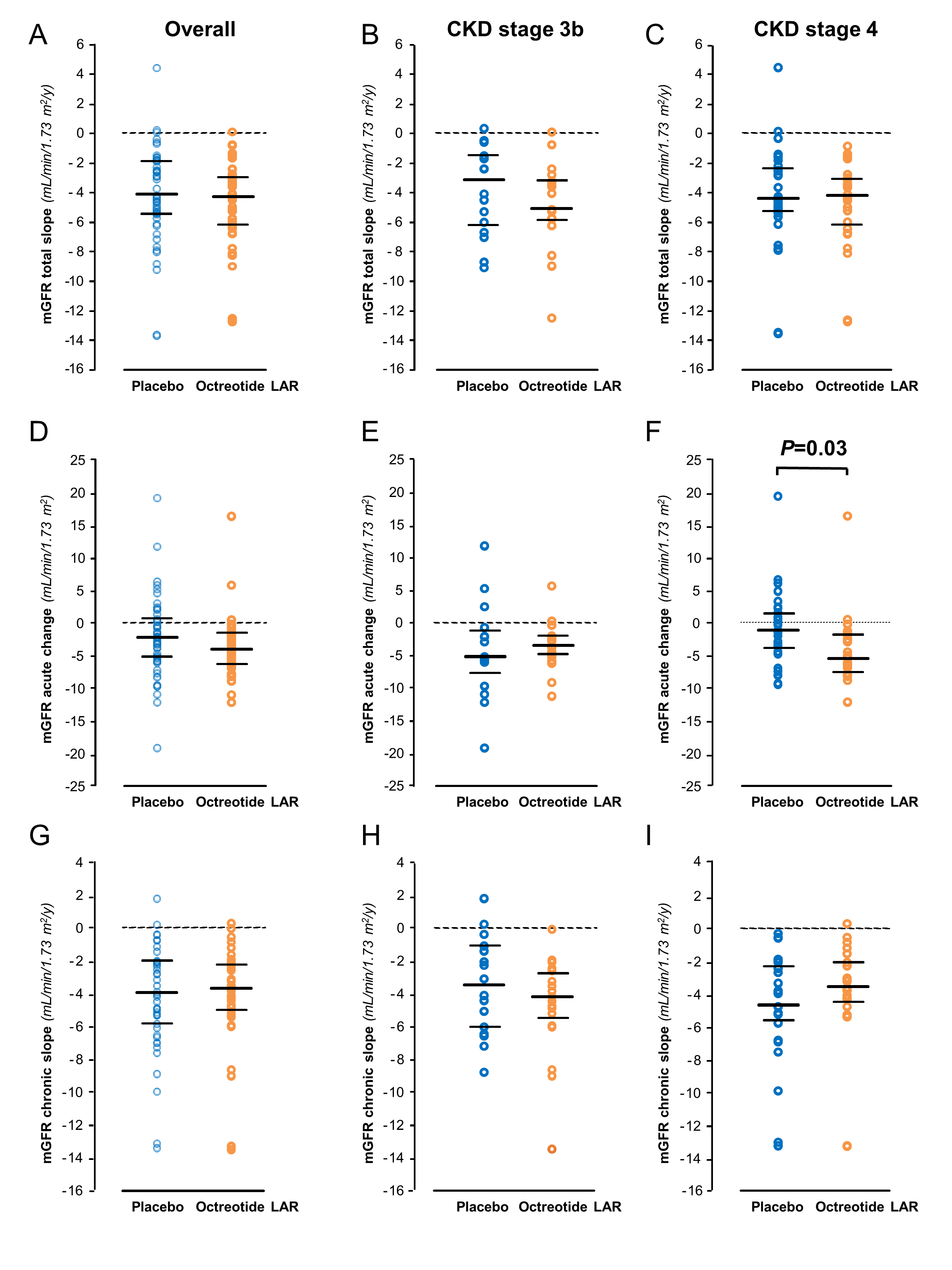

Supplement: S2 Fig — Total measured GFR slope throughout the study period in the patient population as a whole (A), in patients with CKD stage 3b (B), and in patients with CKD stage 4 (C). Short-term measured GFR change from baseline to 6 months in the overall patient population (D), in patients with CKD stage 3b (E), and in patients with CKD stage 4 (F). Chronic measured GFR slope from 6 months to study end in the overall patient population (G), in patients with CKD stage 3b (H), and in patients with CKD stage 4 (I). Circles denote individual values, long lines are median values, and short lines are interquartile ranges. GFR, glomerular filtration rate; TKV, total kidney volume. (TIF) [file pmed.1002777.s004.tif]

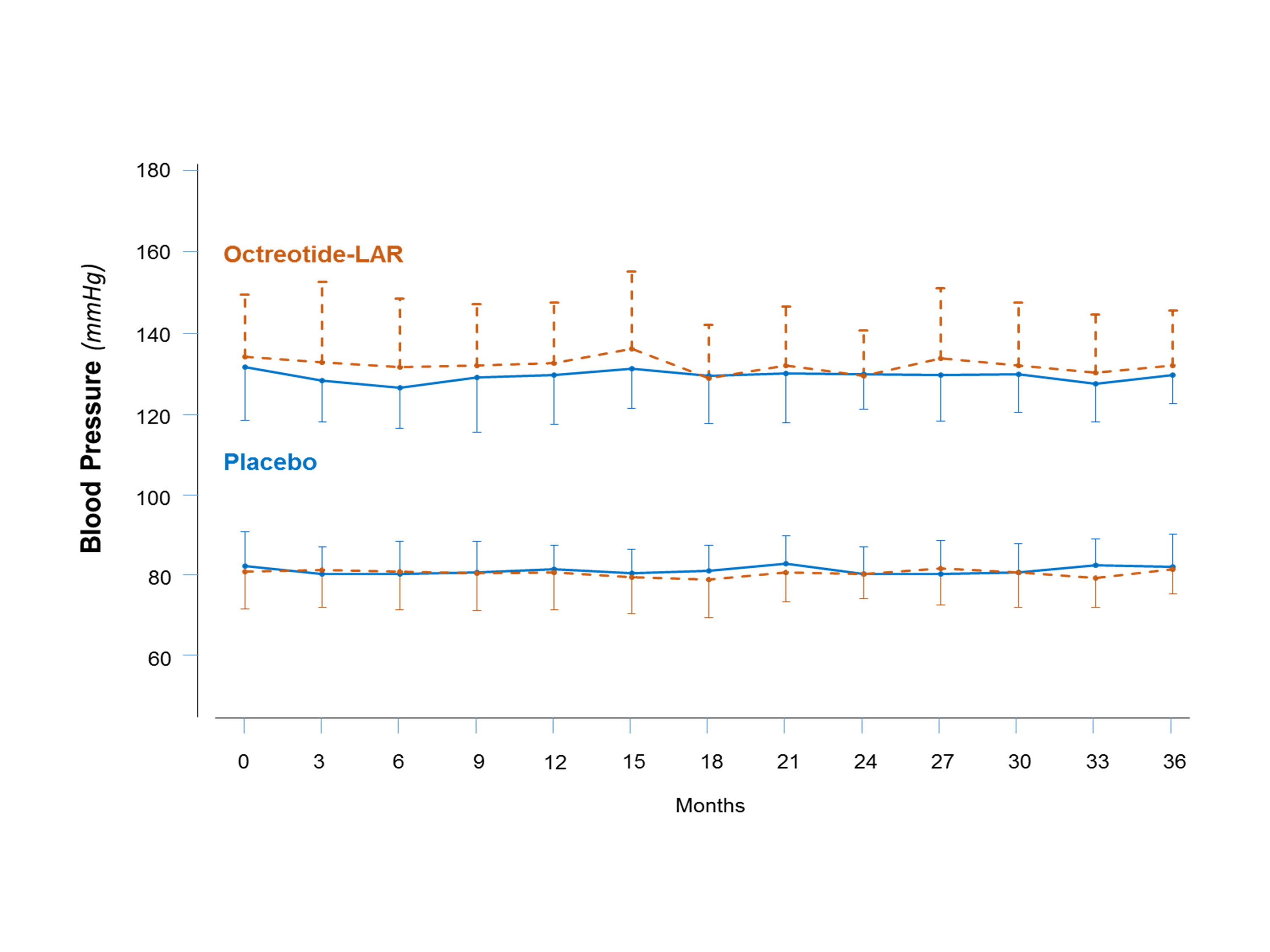

Supplement: S3 Fig — Differences between treatment groups were never significant at any time point of the study. Values are mean ± SD. (TIF) [file pmed.1002777.s005.tif]
