# Supplementary material for: Octreotide-LAR in later-stage autosomal dominant polycystic kidney disease (ALADIN 2): A randomized, double-blind, placebo-controlled, multicenter trial
Source: PLoS Med. 2019 Apr 5;16(4):e1002777. doi: 10.1371/journal.pmed.1002777 (PMC6450618; doi:10.1371/journal.pmed.1002777)
Supplement: S1 Protocol — (PDF) [file pmed.1002777.s006.pdf]

ISTITUTO DI RICERCHE FARMACOLOGICHE MARIO NEGRI

Centro di Ricerche Cliniche per le Malattie Rare "Aldo e Cele Daccò"

Villa Camozzi – 24020 Ranica (BG) – Italy, tel +39.035.45351 – fax +39.035.4535.371
villacamozzi@marionegri.it – http://villacamozzi.marionegri.it

A PROSPECTIVE, RANDOMIZED, DOUBLE-BLIND, PLACEBO CONTROLLED CLINICAL TRIAL TO ASSESS THE EFFECTS OF LONG-ACTING SOMATOSTATIN (OCTREOTIDE LAR) THERAPY ON DISEASE PROGRESSION IN PATIENTS WITH AUTOSOMAL DOMINANT POLYCYSTIC KIDNEY DISEASE AND MODERATE TO SEVERE RENAL INSUFFICIENCY (ALADIN 2 STUDY)

Final version

Bergamo, June 19th, 2013

Prepared by

Norberto Perico, Felipe Rodríguez, Rodolfo Flores Bravo, Luca Antiga, Anna Caroli, Patrizia Ondei, Giorgio Fasolini, Piero Ruggenenti, Flavio Gaspari, Andrea Remuzzi, Giuseppe Remuzzi

Clinical Research Center for Rare Diseases "Aldo & Cele Dacco" Ranica, BG, Italy –
Mario Negri Institute for Pharmacological Research
Unit of Nephrology and Dialysis - Ospedali Riuniti, Bergamo
Unit of Radiology - Ospedali Riuniti, Bergamo

I CONTRIBUTI PER LA RICERCA VERSATI ALL'ISTITUTO SONO FISCALMENTE DEDUCIBILI DAL REDDITO (Gazzetta Uff. N.135 del 13/6/2007)
FONDAZIONE PER RICERCHE ERETTA IN ENTE MORALE, D.P.R. 361 DEL 5/4/1961 - REGISTRO PERSONE GIURIDICHE PREFETTURA MILANO N.227
CONTO CORRENTE POST. N.58337205 - COD. FISC. E PARTITA IVA 03254210150 - ANAGRAFE NAZIONALE RICERCHE COD.G1690099
RECOGNIZED AS A TAX EXEMPT ORGANIZATION UNDER SECTION 501 (c)(3) OF THE USA INTERNAL REVENUE CODE-TAX I.D. No.: 98-6000957
Sistema di gestione qualità certificato da Certiquality UNI EN ISO 9001:2008,
progettazione ed erogazione di corsi di formazione specialistica nell'ambito della biologia e della medicina
SEDE LEGALE: Via Giuseppe La Masa, 19 - 20156 Milano MI - Italy

Confidential Statement

This protocol contains strictly confidential information which is not to be communicated or published unless previously authorized by Mario Negri Institute for Pharmacological Research Clinical Research Center for Rare Diseases

Approved and signed by:

Dr. Piero Ruggenenti ______________________________

Department of Renal Medicine

Mario Negri Institute for Pharmacological Research,

Clinical Research Center for Rare Diseases, Ranica BG

Dr. Norberto Perico ______________________________

Laboratory for Drug Development

Mario Negri Institute for Pharmacological Research,

Clinical Research Center for Rare Diseases, Ranica BG

GENERAL INFORMATION

**Protocol:**	ALADIN 2
**EUDRACT No:**	2011-000138-12
**Title:**	A prospective, randomized, double-blind, placebo controlled clinical trial to assess the effects of long-acting somatostatin (Octreotide LAR) therapy on disease progression in patients with autosomal dominant polycystic kidney disease and moderate to severe renal insufficiency
**Date:**	June 19th, 2013
**Version:**	Final version
**Product:**	Long-acting Somatostatin (Octreotide LAR) 20 mg
**Pharmaceutical form:**	Vial
**Dosage:**	20 mg
**Sponsor:**	Istituto di Ricerche Farmacologiche Mario Negri<br>Centro di Ricerche Cliniche per le Malattie Rare<br>Aldo e Cele Daccò<br>Villa Camozzi – Via G. Camozzi, 3<br>24020 Ranica Bergamo<br>tel. 035.45351
**Sponsor's Medical Expert**	Dr. Piero Ruggenenti<br>Dipartimento di Medicina Renale<br>Centro di Ricerche Cliniche per le Malattie Rare<br>Aldo e Cele Daccò<br>Villa Camozzi – Via G. Camozzi, 3<br>24020 Ranica Bergamo<br>tel. 035.45351

LIST OF ABBREVIATIONS

**ACE**	Angiotensin Converting Enzyme
**ADPKD**	Autosomal Dominant Polycystic Kidney Disease
**ADR**	Adverse Drug Reaction
**AE**	Adverse Event
**ALT**	Alanine aminotransferase
**AST**	Aspartate aminotransferase
**AVH**	Arginine-vasopressin hormone
**cAMP**	Cyclic adenosine monophosphate
**BP**	Blood pressure
**CPK**	Creatine phosphokinase
**CRF**	Case Report Form
**CT**	Computed tomography
**ECG**	ElectroCardioGram
**e-CRF**	Electronic Case Report Form
**ESRD**	End-stage renal disease
**GCP**	Good Clinical Practice
**GFR**	Glomerular filtration rate
**GGT**	Gamma Glutamil Transferase
**HDL**	High Density Lipid
**IEC**	Indipendent Ethics Committee
**IGF-1**	Insulin Growth Factor-1
**LDL**	Low Density Lipid
**MCP-1**	Monocyte chemotactic protein -1
**MNI**	Mario Negri Institute
**QoL**	Quality of Life
**SAE**	Serious Adverse Event
**SD**	Standard Deviation
**SF-HLQ**	Short-Form-Health and Labour Questionnaire
**TKV**	Total kidney volume
**WBC**	White Blood Cells

TABLE OF CONTENTS

GENERAL INFORMATION 4
LIST OF ABBREVIATIONS 5
TABLE OF CONTENTS 6
BACKGROUND 8
AIMS OF THE STUDY 10
  PRIMARY 10
    SHORT-TERM (1-YEAR) 10
    LONG-TERM (3-YEARS) 10
  SECONDARY 11
PATIENTS 13
  INCLUSION CRITERIA 13
  EXCLUSION CRITERIA 13
  STRATIFICATION/RANDOMIZATION 13
STUDY DRUG 14
  LONG-ACTING SOMATOSTATIN 14
  PLACEBO 14
TREATMENT 15
  TREATMENT GROUP 15
  PLACEBO GROUP 15
STUDY DESIGN 16
END POINTS 17
  PRIMARY 17
    SHORT-TERM (1-YEAR) 17
    LONG-TERM (3-YEARS) 17
  SECONDARY 17
SAMPLE SIZE 18
  MORPHOLOGICAL OUTCOMES (ONE YEAR) 18
  GFR DECLINE (THREE YEARS) 18
FOLLOW-UP AND INTERIM ANALYSIS 18
ADPKD – LONG-ACTING SOMATOSTATIN 19
SPIRAL CT SCANNING 20
CENTERS 20
DATA HANDLING AND RECORD KEEPING 21
  ELECTRONIC CASE REPORT FORMS (E-CRF) 21
  STUDY MONITORING 21
  DATA MANAGEMENT 22
  ESSENTIAL DOCUMENT RETENTION 22
ADVERSE EVENT DEFINITION 22
  ADVERSE EVENT 22
  ADVERSE DRUG REACTION 22
  SERIOUS ADVERSE EVENT 23
  COLLECTION/REGISTRATION OF INFORMATION 24
QUALITY OF LIFE AND SOCIETAL COST EVALUATION 25
LEGAL AND ETHICAL ASPECTS 25
  HEALTH AUTHORITIES AND ETHICS COMMITTEE 25
  WRITTEN INFORMED CONSENT 25

INSURANCE POLICY 26
REFERENCES .... ERRORE. IL SEGNALIBRO NON È DEFINITO.
APPENDIX 1: FLOWCHART 28
APPENDIX 2: LIST OF PARTICIPATING CENTERS
APPENDIX 3: SF-36 QUESTIONNAIRE
APPENDIX 4: SF-HLQ QUESTIONNAIRE

BACKGROUND

Autosomal Dominant Polycystic Kidney Disease (ADPKD), the most common hereditary cystic renal disease, has an incidence of 1 in 800 live births and account for 7-10% of patients on dialysis in developed countries (1). ADPKD shows genetic heterogeneity, with two main genes implicated, the PKD gene 1 (85%-90% of the cases) and the PKD gene 2 (10-15% of the cases) which encode for proteins policystin 1 and policystin 2 respectively (1,2). Clinically, ADPKD is characterized by renal and extra renal manifestations (1). In the kidneys, multiple cysts grow from distal and collecting tubular epithelial cells producing progressive renal enlargement with relatively initial stable renal functions (3). Thereafter, both tubular and secondary interstitial damage lead to faster renal loss and end-stage renal disease (ESRD) in approximately half of all patients affected in their fifth or sixth decade of life (2,4). More than 50% of the patients display hepatic cysts derived from cholangiocyte proliferation and fluid secretion (5). Pancreatic and intestinal cysts as well as increased risk of aortic aneurysms, heart-valve defects and sudden death due to rupture of intracerebral aneurysms are extra-renal manifestations, too (6). The precise molecular pathogenic mechanisms of ADPKD are not completely understood, but experimental models in renal tubular cells suggest that defect in policystin 1 or policystin 2 carry out to decrease intracellular calcium and increase cAMP levels changing the cell toward a proliferative and secretor phenotype (7,8,9). Cysts growth and enlargement are mainly consequence of epithelial cells proliferation and fluid chloride secretion through apical channels dependent of cAMP (10,11). Likewise, for hepatic cyst development, the proposed mechanisms are linked to abnormalities in epithelial cell proliferation and secretion in response to high intracellular cAMP concentrations secondary to policystin defects (12).

Patients with ADPKD, at similar levels of proteinuria and blood pressure control, do not seem to benefit to the same extent of ACE inhibitor therapy and have faster decline in glomerular filtration rate (GFR) compared with other chronic kidney diseases (13). Thus, in ADPKD renoprotective interventions - in addition to achieving maximal reduction of arterial blood pressure and proteinuria, and limiting the effects of other potential disease progression promoters (such as dyslipidemia, chronic hyperglycemia, or smoking) (14,15) - should also be specifically aimed to correct the dysregulation of epithelial cell growth, fluid secretion, and extracellular matrix deposition that is characteristic of this disease. Up to now, no specific therapies for ADPKD are available, but drugs like somatostatin, rapamycin, and

tolvaptan targeting to growth and chloride secretions pathways are now being testing worldwide in some clinical trials (16,17).

Somatostatin is an endogenous cycled polypeptide presents in two active forms (14 and 18 amino acids) with endocrine, paracrine and autocrine actions (18). The effects of somatostatin are pleiotropic depending on the tissue and the type of receptors it binds to. Five subtypes of somatostatin receptors have been described (sst1-sst5), but on the renal tubular epithelial cells, sst1 and sst2 receptors predominate. Of interest, it has been reported that somatostatin inhibits the increase in intracellular cAMP and chloride secretion induced by arginine-vasopressin hormone (AVH) in rectal glands of sharks (19,20). Octreotide, a synthetic analog of somatostatin with longer half-life and higher sst2 affinity than naive polypeptides (21), has been used with negligible side effects for long-term treatment of acromegaly (22) and some malignant tumors (23).

Considering the inhibitory effect of somatostatin on cell proliferation and chloride secretion, we have performed some years ago a pilot prospective cross-over controlled study with long-acting somatostatin analog in patients with ADPKD and different degree of renal dysfunction (17). We found that in these patients, 6 month treatment with octreotide was safe, well tolerated, and slowed the time-dependent increase in total kidney volume to a significant extent compared to placebo. The net effect in kidney volume resulted from an action of the drug on cyst volume and on parenchyma volume (17). Moreover, more recent post-hoc analysis of the concomitant liver disease progression in the same ADPKD patients demonstrated a significant reduction in the total liver volume during octreotide treatment, not appreciably observed during placebo. The beneficial effect of octreotide has been then confirmed in rodent models of polycystic liver and kidney disease where the drug significantly reduced liver weight, cyst volume, hepatic fibrosis and mitotic indexes by reducing the cholangiocyte and serum cAMP levels (24). Moreover, in the untreated ADPKD patients enrolled in our study, computed tomography evaluation of disease progression showed that the ratio of faintly contrast-enhanced parenchyma volume – referred as intermediate parenchyma – over total parenchyma volume strongly correlated with basal GFR and GFR changes during the observation period (25).

The good safety profile of octreotide and the slowing of renal growth demonstrated in our short-term clinical study did suggest the feasibility of a randomized trial in larger series of ADPKD patients with normal renal function or mild renal insufficiency to verify whether long-term somatostatin treatment may eventually provide effective renoprotection. This trial - the ALADIN study - is ongoing and the planned ADPKD patients have been enrolled. So far, no particular side effects have been reported. More

important, preliminary interim analysis of data from patients who reached 1 year treatment, confirmed the beneficial effect of octreotide in slowing the growth of total kidney volume compared to placebo. There is urgent need for renoprotection in ADPKD patients, particularly for those with more advanced renal dysfunction, for whom so far no clinical trials have been designed. The findings of the safety and potential benefit of octreotide in few patients with severe renal insufficiency observed in our initial pilot study (17) and the encouraging preliminary long-term effect results of octreotide on kidney growth, make worth investigating the efficacy of a long-acting somatostatin (Octreotide LAR) in slowing or even halting the kidney enlargement and renal function decline in ADPKD patients with moderate/severe renal failure.

AIMS OF THE STUDY

The general aim of the trial is to assess the efficacy of one year treatment with long-acting somatostatin analogue (Octreotide LAR) compared with placebo in slowing kidney and liver growth rate in patients with ADPKD and moderate/severe renal insufficiency (estimated GFR by MDRD 4 variables: 15-40 ml/min/1.73m^2) and to assess whether and to which extent this translates into slower renal function decline over 3-year follow-up.

Specific objectives are the followings:

Primary

Short-term (1-year)

To compare the effect of long acting somatostatin analogue (Octreotide LAR) versus placebo on total kidney volume (TKV) change (delta TKV) as assessed by spiral computed tomography (spiral CT) scan.

Long-term (3-years)

To compare the effect of long-acting somatostatin analogue (Octreotide LAR) vs. placebo on the rate of GFR decline as assessed by serial measurements of the iohexol plasma clearance.

Secondary

To compare, among the two groups, absolute and relative changes in renal and liver volume parameters (at month 0, 12 and 36) as well as in functional and biochemical parameters by quarterly clinical visits during the 36 month follow-up. In particular, the following parameters will be assessed:

- Total kidney volume (at 3-years)
- Total renal cyst volume*
- Total renal parenchyma volume*
- Total renal intermediate volume*
- Total liver and liver cyst volumes*

  **When feasible, by minimized radio contrast agent administration during CT acquisition*
- GFR decline (as assessed by serial measurements of the plasma iohexol clearance at month 0 and, every 6 months thereafter)
- Slope of eGFR (by MDRD 4 variable equation)
- Progression to the combined end point of doubling of serum creatinine and/or ESRD°
- Progression to ESRD considered as a single endpoint°

  ° ESRD is defined as need for chronic renal replacement therapy by dialysis or transplantation established on the basis of standard clinical criteria by physicians who are in charge of the patients and are blind to treatment allocation and patient measured GFR
- Systolic, diastolic and mean blood pressure
- 24 h urinary protein excretion rate (at month 0 and every six months thereafter)
- Urinary osmolality (assessed from urine sodium, urea and glucose concentration on 24 hr urine samples)
- Morning spot urine sample (proteins, albumin, creatinine)
- Serum Insulin Growth Factor-1 (IGF-I)
- 24 h urinary phosphorus, creatinine and creatinine clearance
- Gallbladder sand/stones and renal stones (by ultrasonography).

Exploratory

- To assess treatment effect on endothelin and MCP-1 urinary excretion rate (overnight urine sample), in a subgroup (pending on study findings and fund availability)

Safety

To assess the safety profile of the somatostatin analogue, the following parameters will be monitored:

At months 3-9-15-21-27-33:

- Serum creatinine and urea
- Hepatic profile (ALT, AST, GGT, alkaline phosphatase, total and direct bilirubin, serum total proteins and albumin)
- Fasting blood glucose
- Serum electrolytes (sodium, potassium, calcium, phosphorus)
- Serum uric acid
- Blood cell count (WBC, Ht, Hb, platelet count)
- Morning spot urine sample (proteins, albumin, creatinine)
- Gallbladder sand/stones and renal stones (by ultrasonography)

In addition history of allergy/intolerance to radio-contrast agents (despite pre-medication with antihistamine drugs and/or steroids) will be assessed before the spiral computed tomography

At months 6-18-30: the same scheduled for month 3 plus

- serum creatin phosphokinase (CPK)
- Lipid profile (total cholesterol, LDL- cholesterol, HDL-cholesterol, triglycerides)

At month 24: the same scheduled for month 6 plus

1. Fasting blood glycosylated hemoglobin

At month 0-12-36: the same scheduled for month 24 plus

1. IGF-1

PATIENTS

Inclusion Criteria

- Age > 18 years
- Clinical and ultrasound diagnosis of ADPKD
- Estimated GFR between 15 and 40 ml/min/1.73m^2 (by the MDRD 4 variable equation)
- Written informed consent

Exclusion Criteria

- 24-h Urinary protein excretion rate >3g (suggestive of a concomitant glomerular disease that could benefit of specific therapy)
- Symptomatic urinary tract lithiasis or obstruction
- Uncontrolled diabetes mellitus (HbA1c >8%) or hypertension (systolic/diastolic BP >180/110 mmHg)
- Current urinary tract infection
- Symptomatic biliary tract lithiasis
- Active cancer
- Psychiatric disorders or any condition that might prevent full comprehension of the purposes and risks of the study
- Pregnancy, lactation or child bearing potential and ineffective contraception (estrogen therapy in post menopausal women should not be stopped)

Stratification/Randomization

Eligible patients will be stratified for the presence (YES or NO) of concomitant clinical conditions that could appreciably affect the rate of renal function loss over time including diabetes mellitus, 24 hour urinary protein excretion >1g, or any other evidence of concomitant renal disease without indication for specific therapy.

Within each stratum patients will be randomized on a 1:1 basis to Long-acting Somatostatin (Octreotide LAR) 40 mg (treatment group) or saline solution (placebo group). All study participants including patients, doctors and nurses involved in patient care, and technicians, monitors and statisticians involved in data analyses will be blinded to treatment allocation. The only subject who will not be blinded to treatment allocation will be the research nurse who will prepare the study drug (the active compound or

placebo) and will administer it to the patient. This subject will not be involved in any phase of study conduction. Randomization will be centralized at the Laboratory of Biostatistics of the Clinical Research Center for Rare Diseases "Aldo e Cele Daccò" Villa Camozzi, Ranica, Bergamo of the Mario Negri Institute for Pharmacological Research under the responsibility of an independent investigator.

STUDY DRUG

Long-acting Somatostatin (Octreotide LAR)

Study drug will be manufactured by Novartis Farma S.p.a. and will be packed in a carton box containing one glass vial of Long-acting Somatostatin (Octreotide LAR) 20 mg, prefilled syringe containing vehicle solution and two needles.

Boxes will be labeled with "tear-off" labels. When administering the study medication, then Investigator will peel off the respective part of the label from the each box and will fix these to the provided space in the appropriate form.

All medication supplies must be stored separately from normal hospital/practice stocks in a refrigerator grade temperature between 2°C to 8°C. Keep vial in the outer carton in order to protect it from light. The study medication can remain below 25°C on the day of the injection. However the suspension must only be prepared immediately prior to intra muscular application.

The Investigator or the hospital pharmacist will confirm receipt and disposition of the study drug in writing, including all following supplies. Unused drugs should be returned. The Investigator will administer the study drug only to the patients included in the trial by following the procedures set out in the study protocol. It is prohibited to use the trial drug for any other purpose.

Placebo

Placebo will be saline solution with identical volume, presentation and general aspect as compared to the study drug. All considerations and precautions for storage, preparation, application and disposal mentioned above for the study drugs should be also keeping in count for the placebo.

TREATMENT

Treatment Group

Patients randomized to treatment group will receive intramuscularly *Long-Acting Somatostatin (Octreotide LAR) (Novartis, Basel)* at the dose of 40 mg every 28 days (in two intragluteal 20 mg injections) for three years. This is the regimen that is currently recommended for chronic treatment of pituitary adenomas and acromegaly (26) and that has been shown to slow renal (17) and liver growth (27) in APDK patients. At this dosage treatment with *Long-Acting Somatostatin (Octreotide LAR)* was safe and well tolerated in long-life chronic therapy for pituitary adenomas and acromegaly, and in clinical trials of ADPKD patients. Thus, the definition of the above treatment regimen is based on previous evidence that this is the optimal regimen to test the working hypothesis of the present study without exposing study patients to an excess risk of side effects. In previous studies in ADPKD patients the planned dose was well tolerated and in no case it had to be reduced or the treatment had to be withdrawn. However, in the present study, in the case of treatment related adverse events the dosage will be halved. If the adverse event does not recover, treatment will be stopped and patient will be maintained on active follow up until study end. The adverse event will be monitored until complete resolution.

Placebo Group

Patients randomized to placebo group will receive intramuscularly saline solution at the same volume of study drug every 28 days (in two intragluteal injections) for three years.

In addition, both groups will be given conventional therapy when needed. Conventional treatment relates usually to the administration of antihypertensive drugs for patients with high blood pressure (target =130/80 mm Hg). If there are no contraindications, ACE inhibitors or ARBs will be considering first choice antihypertensive drugs. No major change in antihypertensive treatment should be introduced throughout the whole study period unless deemed clinically necessary (the reason of the changes should be clearly explained in the CRF). This approach is aimed to minimize the confounding effect that drugs acting on renin angiotensin axis can produce on renal hemodynamic and urinary protein excretion rate. Any decrease in the overall dose of antihypertensive drugs needed to maintain the target blood pressure could be taken as an indirect evidence of a beneficial effect of somatostatin on kidney function. Also, co-factors that may contribute to progression of renal disease independently of the cystic disease (such as dyslipidemia, de-novo diabetes, smoking habit) will be approached according to specific medical guidelines. Concomitant changes in blood glucose levels and need for anti-diabetic therapy throughout

the study period should be carefully monitored and reported in the CRF, since they could be taken as an indirect evidence of an untoward effect of somatostatin on insulin sensitivity and glucose metabolism. If gallbladder sand or stones are documented during the scheduled serial ultrasound evaluation, ursodeoxycholic acid will be prescribed.

STUDY DESIGN

This will be a prospective, double-blind, placebo-controlled, randomized three-year study .

After randomization, eligible ADPKD patients will undergo baseline evaluation of biochemical, urinary and renal hemodynamic parameters. Blood samples will be taken in the morning with the patient fasting from the evening before (at least 10 hour since the last meal) for laboratory evaluations including study and safety blood tests. The patients will bring a 24 hour urine collection for measurement of urine volume, protein, sodium, urea, glucose, phosphorus, endothelin and MCP-1 excretion, as well as urinalysis on random urine samples. Urinary osmolality will be calculated by standard formula. GFR ~~F~~, by plasma iohexol clearance, will be measured. In addition, baseline kidney and liver volumes will be evaluated by spiral-CT scanning. Thereafter, patients will start treatment according to randomization.

Blood pressure and pulse rate will be measured at the dominant arm with the patient in sitting position (by a standard sphygmomanometer or other non-invasive device) using the average of 3 measures taken consecutively 2 minutes apart, after 10 rest minutes. Using serum creatinine values and anthropometric parameters, GFR will be estimated at each time points by MDRD equation (4 variables) and the slope determined from baseline to 1 year follow-up. The GFR will be measured every six months by the iohexol plasma clearance technique, kidney/liver volume (by spiral-CT) will be evaluated at 1 and 3 years treatment. Throughout the whole study period, additional laboratory evaluations will be performed whenever deemed clinically appropriate. Safety will be monitored throughout the whole duration of the study. Concomitant and intercurrent diseases will be treated according to the best available treatment. Any adverse event will be recorded and any patients that should or would be withdrawn from the study will be notified. A detailed annotation should be written in the clinical file and in the case report forms (CRF).All patients will be maintained on their treatment arm until study end (3 years) unless results of interim analyses planned at 1 year follow-up will show that the study has to be prematurely stopped for efficacy or safety/futility reasons. In the first case all subjects will be maintained on active treatment with long acting somatostatin (Octreotide LAR), in the second case the study will be stopped.

END POINTS

Primary

Short-term (1-year)

Total kidney volume (TKV) change (delta TKV) as assessed by spiral computed tomography (spiral CT) scan.

Long-term (3-years)

Rate of GFR decline as assessed by serial measurements of the iohexol plasma clearance.

Secondary

Renal and liver volume parameters and functional/biochemical parameters including

- Total renal cyst volume*
- Total renal parenchyma volume*
- Total renal intermediate volume*
- Total liver and liver cyst volumes*

  **When feasible, by minimized radio contrast agent administration during CT acquisition*
- GFR decline (as assessed by serial measurements of the plasma iohexol clearance at month 0 and, every 6 months thereafter)
- Slope of eGFR (by MDRD 4 variable equation)
- Progression to the combined end point of doubling of serum creatinine and/or ESRD°
- Progression to ESRD considered as a single endpoint°

  ° ESRD is defined as need for chronic renal replacement therapy by dialysis of transplantation established on the basis of standard clinical criteria by physicians who are in charge of the patients and are blind to treatment allocation and patient measured GFR
- Systolic, diastolic and mean blood pressure
- 24 h urinary protein excretion rate (at month 0 and every six months thereafter)
- Urinary osmolality (assessed from urine sodium, urea and glucose concentration on 24 hr urine samples)
- Morning spot urine sample (proteins, albumin, creatinine)
- Serum Insulin Growth Factor-1 (IGF-I)
- 24 h urinary phosphorus, creatinine and creatinine clearance

Exploratory

- To assess treatment effect on endothelin and MCP-1 urinary excretion rate (overnight urine sample), in a subgroup (pending on study findings and fund availability)

Safety

SAMPLE SIZE

Morphological outcomes (one year)

The following assumptions have been made for the sample size calculation:

a. The expected mean (SD) change in TKV between baseline and 1 year in the conventional therapy group is +103.4 (149.5) ml. (Data obtained from interim results of the ALADIN trial)
b. No change is expected in the active treatment group (i.e. a mean change equal to 0.0 ml).

It is therefore estimated that the study requires 98 patients considering 30% of drop out, i.e. 49 patients per group (alpha= 0.05, two tailed test, power=0.80, t-test).

GFR decline (three years)

Assuming a yearly GFR decline (mean±SD) of 6.31±4.47 ml/min/1.73m^2 in controls on placebo (data from ADPKD patients with severe renal insufficiency included in the REIN study), the sample size of 49 patients per group will provide the trial an 81% power to detect as statistically significant (alpha=0.05, two tailed test) a 50% (or larger) reduction in the rate of GFR decline in the active treatment arm (i.e from 6.31 to 3.16 ml/min/1.73m^2) as compared to the placebo arm. This difference would be clinically relevant.

FOLLOW-UP AND INTERIM ANALYSIS

All patients will be maintained on their original treatment arm and on active follow-up for one year of treatment. At this stage, the treatment effect on the main outcome variable (absolute change in TKV measured by initial and at one year simple spiral-CT), will be assessed in ad hoc interim analysis.

One interim efficacy comparison will be undertaken at significance levels which ‘preserve’ an overall 5% level of significance. The nominal significance levels used will be (28):

- Analysis 1 a = 0.005
- Analysis 2 a = 0.049

Other details are displayed in Table 1. This interim analysis will be undertaken on the intention-to-treat population using the primary efficacy endpoint and comparing long acting somatostatin and conventional therapy after the 49th patient completed 1 year follow up. It is not intended for this analysis to result in early termination of the trial, although the trial could be stopped for safety reasons. Furthermore, possible adaptation of the sample size or observation period in order to achieve adequate power will take place. It is possible however, that the trial may be terminated on the basis of futility. If the 100 (1-a) % confidence interval for the primary endpoint comparing long-acting somatostatin and conventional therapy excludes clinically important effects then termination of the trial will be considered. The minimum important difference is set to 15% (i.e. from +103.4 to +87.9 ml).

Table 1. ADPKD – Long-Acting Somatostatin (Octreotide LAR)

Interim Analysis

Difference in favor of	Interim Analysis p-value (α´= 0.005)	Stopping rule
1- Long-acting Somatostatin	$p < 0.005$	Stop study early
2- Long-acting Somatostatin	$p \geq 0.005$ (unless $p \geq 0.049$ and Δ excluded from the 99% CI)	Completed study as planned
3- Long-acting Somatostatin	$p \geq 0.049$ and Δ excluded from the 99% CI	Stop study for futility
4- Placebo	$p \geq 0.005$ and Δ included in the 99% CI	Stop study for futility (impossibility to obtain a reversal of the results at this point of the study)
5- Placebo	$p < 0.05$ or $p \geq 0.05$ and Δ excluded from the 99% CI	Stop study for emerging evidence of the placebo superiority

Δ = minimal important difference in favor of Long-acting Somatostatin (i.e. Long-acting Somatostatin - conventional therapy = 103.4 – 87.9 = 15.5 ml

99% CI = 99% Confidence Interval for the difference in the primary endpoint.

The decision to stop or continue the study however will be taken by the Steering Committee not only on the basis of the above statistical criteria, but also on the basis of clinical judgment of the other safety and efficacy outcome variables.

SPIRAL CT SCANNING

Volumetric computed tomography (CT) acquisitions of the complete kidneys and liver will be carried out on a 64-slice scanner (LightSpeed VCT, GE Healthcare, Milwaukee, WI). A first thick-slice, low-dose survey scan will be performed in order to identify the axial extension of the kidneys and liver and adequately prescribe the acquisition volume of the subsequent thin-slice scan. The latter will be performed 80 seconds after the injection of 100 ml non-ionic iodinated contrast agent (Iomeron 350, Bracco, Italy) at an injection rate of 2 ml/s, followed by 20 ml physiologic solution at the same injection rate. The thin-slice contrast-enhanced scan will be performed using 2.5 mm collimation, pitch 0.984, 2.5 mm increment, 120 kV voltage and a maximum of 500 mA intensity. Images will be transferred on digital media immediately after the scanning session and transferred to the Medical Imaging Unit, Biomedical Engineering Department, Mario Negri Institute, for processing and quantification. Quantification of total kidney, cystic, intermediate and parenchymal volumes will be performed automatically on the basis of image intensity statistics following the approach described in (25).

CENTERS

The study will be coordinated and monitored by the Clinical Research Center for Rare Diseases *Aldo and Cele Daccò* of the Mario Negri Institute for Pharmacological Research, Ranica, Bergamo. It will be conducted at Department of Renal Medicine of the Clinical Research Center *Aldo and Cele Daccò* and at Nephrology Units listed in Appendix 2.

DATA HANDLING AND RECORD KEEPING

Electronic case report forms (e-CRF)

Demographic, efficacy and safety data will be collected for the purpose of the study, to be registered by the Investigator (or his/her designated collaborator) on the web based e-CRFs generated by Biomedical Technologies Laboratory of MNI (Ranica – Bg).

Every Investigator and the designated personnel will be provided with the credentials for the use of computerized system.

The Investigator will be responsible for the accuracy of the data entered in the e-CRFs. All entries must be written in English. Source documents should be available to support all the data recorded in the e-CRFs (a reasonable explanation must be given for all missing data). Location of source data will be specified and listed at the Center initiation visit.

The data must be collected for randomized patient and for patients who, after having consented to participate, underwent baseline examinations but were not further randomized.

Study monitoring

The study will be monitored by the staff of the Mario Negri Institute Drug Monitoring Unit. The Investigator agrees to allow monitoring visits on site (clinical area and laboratory) at regular times prior, during and after the completion of the study. These visits are designed to implement Good Clinical Practice requirements. The Investigator will ensure that adequate time will be available for the Clinical Monitor to review case report forms, study source and raw data, and to assure the accuracy and completeness of the recorded data.

The Monitor will normally require time to go through the e-CRFs and associated source documents alone and if required, the Investigator and/or Co-Investigator should be available on the day of the visit, in order to clarify any details and answer any queries raised by the Clinical Monitor.

During the visits, the Clinical Monitor will ensure that there is adherence to the protocol, and that patients included in the study meet the inclusion criteria and that patients and/or caregivers have given their written informed consent. The Clinical Monitor will collect completed and signed case report forms as they become available.

Data Management

A systematic and extensive electronic validation of the quality of the data base will be performed.

Previous and concomitant medication, previous diseases and adverse events will be coded according to standardized dictionaries. Once all data discrepancies will be corrected, the database will be locked and data converted into SAS-transport files.

Essential document retention

The investigator will retain copies of all the essential documents as required by the applicable regulatory requirements. The investigator should take measures to prevent accidental or premature destruction of these documents. The essential documents include at least: the signed protocol, copies of the completed CRFs, signed patient informed consent documents from all patients who consented, hospital records and other source documents. Also, International Ethical Committee (IEC) approved documents and all other documentation included in the investigator site file and pharmacy/dispensing file are considered essentials.

ADVERSE EVENT DEFINITION

Adverse Event

Any untoward medical occurrence in a patient or clinical investigation subject administered a pharmaceutical product and which does not necessarily have to have a causal relationship with this treatment.

An adverse event (AE) can therefore be any unfavourable and unintended sign (including an abnormal laboratory finding, for example), symptom, or disease temporally associated with the use of a medicinal product, whether or not considered related to the medicinal product.

Adverse Drug Reaction

An adverse drug reaction (ADR) is a noxious and unintended response to a medicinal product related to any doses. The phrase “response to a medicinal product” means that a causal relationship between a medicinal product and an adverse event is at least a reasonable possibility, i.e. the relationship cannot be ruled out.

Serious Adverse Event

A serious adverse event is any untoward medical occurrence that at any dose:

- results in death,
- is immediately life-threatening,

  NOTE: the term "life-threatening" in the definition of "serious" refers to an event in which the patient was at risk of death at the time of the event; it does not refer to an event which hypothetically might have caused death if it were more severe.
- requires inpatient hospitalisation or prolongation of existing hospitalisation,

  a) elective surgery or cases where the decision to hospitalize the patient was made before the signed consent or attendance at hospital without an overnight stay, is not hospitalisation

  b) event which would have normally required hospitalisation, but the patient was not hospitalised (for whatever reason) is hospitalisation

  c) if the patient was already in the hospital when the event occurred, and the event required inpatient treatment (i.e. the patient would have been hospitalized unless she/he already was) but did not cause the prolongation of the hospitalization, the event must be reported as serious for hospitalization;
- results in persistent or significant disability/incapacity, or
- is a congenital malformation/birth defect.
- other: it is a category that covers events that are considered as serious, when based upon appropriate medical judgment that they may jeopardise the patient/subject, and may require medical or surgical intervention to prevent one of the other seriousness criteria from occurring;

this also includes:

- patient requiring treatment in order to prevent serious outcome: if a patient required treatment but not hospitalisation in order to prevent permanent and/or severe disablement or fatal outcome, then the event is reported per serious standard. Treatment may involve a physician's visit or presentation to the emergency room.
- cancer: if cancer is the indication for treatment , only cancers of new histology and cases where there is clear evidence of exacerbation of an existing cancer qualify as a serious event. Every new occurrence of cancer must be reported as a serious event regardless of the duration between discontinuation of the drug and the occurrence of the cancer.
- abuse or dependency: it qualifies as serious for other medical reasons

An event which doesn't meet these definitions is considered to be "Not Serious".

Collection/Registration of information

Adverse events will be monitored at each visit and by telephone reports from investigators as needed between outpatient visits.

Any adverse event reported spontaneously or upon questioning will be evaluated by the Investigator and recorded on the relevant page in the electronic case report form.

However, if the event includes a group of signs and symptoms, which, when considered together, are known or presumed to characterize a syndrome, only one form must be filled in. In this case, all events will be fully described, but any additional information such as intensity, outcome, causality, etc., will refer to the syndrome only. For each adverse event the Investigator is required to evaluate the nature of the event, the date and time of its onset (if known), the date and time the adverse event stopped (if known), the duration of the event and its maximum intensity. The Investigator will record any remedial action taken and the final outcome of the adverse event. All modern facilities for resuscitation must be immediately available in case of emergency. The Investigator is also required to assess the causal relationship between the event and the study treatment.

All the patients experiencing adverse events must be monitored until symptoms and any abnormal laboratory values have returned to baseline, or until there is a satisfactory explanation for the changes observed, or until death, in which case a full pathologist's report should be supplied, if possible. All findings must be recorded on the "adverse event" page in the case report forms and in the patients' medical records.

As soon as possible one or more new follow-up reports until the event resolution must be prepared by the Investigator and sent by fax to the MNI.

The contact addresses for reporting of serious adverse events is:

Nadia Rubis, *Clinical Monitor*

Pharmacovigilance Secretariat

Istituto di Ricerche Farmacologiche Mario Negri

Centro di Ricerche Cliniche per le Malattie Rare

Aldo e Cele Daccò

Via G. B. Camozzi, 3

I-24020 Ranica (Bergamo)

Italy

SAEs Fax: **+39 035 4535371**

Tel: +39 035 45351

e-mail: farmacovigilanza.vc@marionegri.it

EXPLORATIVE QUALITY OF LIFE AND SOCIETAL COST EVALUATION

This study is also aimed to perform an exploratory analysis of the quality of life and the societal costs by submitting to consenting patients a Q*uality of life (QoL) Questionnaire* - Version 1 of SF-36 (QoL SF-36 V1TM Questionnaire) validated in Italy – and a *Short-Form-Health and Labour Questionnaire (SF-HLQ).*

LEGAL AND ETHICAL ASPECTS

Health Authorities and Ethics Committee

The study protocol and all the other appropriate documents will be submitted to the Regulatory Authorities in accordance with local legal requirements. The investigational sites will not commence study procedures until has received the appropriate written approval. All protocol amendments, treatment-related serious adverse events and new versions of the Investigator's Brochure must be submitted to the Principal Investigators and to the Ethics Committee.

Written Informed Consent

Good Clinical Practice guidelines require that a written informed consent be obtained from each patient. Doctor must inform patient and caregiver of the aims of the study and how it will be organized, the type

of study treatments, the anticipated benefits which can be expected from the study, any potential hazards of the study and discomfort it may entail, alternative treatments, the freedom to ask for further information at any time, the patient's right to withdraw from the trial at any time without giving reasons and without jeopardizing the further course of the treatment, the existence of patient's insurance cover and obligations following from this cover. This information should be given in both oral and written form. The patient and the caregiver should have sufficient time to decide whether or not to take part in the study.

Insurance policy

The study is covered by an insurance policy, according to the laws in force, in the event of a patient suffering any significant deterioration in health or well-being, which is proven as being as a direct result of study participation.

REFERENCES

1. P Wilson. Mechanisms of disease: Polycystic Kidney Disease. N Engl J Med 2004; 350:151-64.
2. M Simons, G Walz. Polycystic kidney disease: Cell division without a c(l)ue?. Kidney Int 2006; 70: 854–864.
3. Grantham JJ, Chapman AB, Torres VE. Volume progression in autosomal dominant polycystic kidney disease: the major factor determining clinical outcomes. Clin J Am Soc Nephrol 2006; 1: 148-157.
4. E Meijer, P Jong, D Peters, R Gansevoort. Better understanding of ADPKD results in potential new treatment options: ready for the cure. J Nephrol 2008; 21: 133-138.
5. Alvaro D, Onori P, Alpini G, Franchitto A, Jefferson D, Torrice A et al. Morphological and functional features of hepatic cyst epithelium in autosomal dominant polycystic kidney disease. Am J Pathol. 2008 Feb;172(2):321-32.
6. Ul Haque, Moatasim A. Adult polycystic kidney disease: a disorder of connective tissue? Int J Clin Exp Pathol 2008; 1(1):84-90.
7. E Meijer, P Jong, D Peters, R Gansevoort. Better understanding of ADPKD results in potential new treatment options: ready for the cure. J Nephrol 2008; 21: 133-138.
8. T Yamaguchi, D Wallace, B Magenheimer, S Hempson, J Grantham, J Calvet. Calcium restriction allows cAMP activation of the B-Raf/ERK pathway, switching cells to a cAMP-dependent growth-stimulated phenotype. J Biol Chem 2004; 279: 40419–40430.
9. T Yamaguchi, S Hempson, G Reif, A Hedge, D Wallace. Calcium restores a normal proliferation phenotype in human polycystic kidney disease epithelial cells. J Am Soc Nephrol 2006; 17: 178–187.
10. M Albaqumi, S Srivastava, Z Li, O Zhdnova, H Wulff. KCa3.1 potassium channels are critical for cAMP-dependent chloride secretion and cyst growth in ADPKD. Kidney Int (advance online publication, June 11, 2008).
11. B Yang, N Sonawane, D Zhao, S Somlo, A. Verkman. Small-molecule CFTR inhibitors slow cyst growth in polycystic kidney disease. J Am Soc Nephrol 2008; 19: 1300–1310.
12. Francis H, Glaser S, Ueno Y, Lesage G, Marucci L, Benedetti A et al. cAMP stimulates the secretory and proliferative capacity of the rat intrahepatic biliary epithelium through changes in the PKA/Src/MEK/ERK1/2 pathway. J Hepatol. 2004 Oct;41(4):528-37.

13. Van Dijk MA, Breuning MH, Duiser R, van Es LA, Westendorp RG. No effect of enalapril on progression in autosomal dominant polycystic kidney disease. Nephrol Dial Transplant 2003; 18: 2314-2320.
14. Ecder T et al: Effect of antihypertensive therapy on renal function and urinary albumin excretion in hypertensive patients with autosomal dominant polycystic kidney disease. Am J Kidney Dis 2000; 35: 427-432.
15. Torres VE. Hypertension, proteinuria, and progression of autosomal dominant polycystic kidney disease. Am J Kidney Dis 2000; 35: 547-550.
16. A. Chapman. Autosomal Dominant Polycystic Kidney Disease: Time for a Change? J Am Soc Nephrol 2007; 18: 1399-1407
17. Ruggenenti P, Remuzzi A, Ondei P, Fasolini G, Antiga L, Ene-Iordache B, Remuzzi G, Epstein F. Safety and efficacy of long-acting somatostatin treatment on autosomal dominant polycystic kidney disease. Kidney Int 2005; 68: 206–216.
18. M. Pawlikowski, G. Melen-Mucha. Perspective of new potential therapeutic applications of somatostatin analogs. Neuroendocrinology Letters 2003; 24: 21-27.
19. P Silva et al: Mode of action of somatostatin to inhibit secretion by shark rectal gland. Am J Physiol 1985 249:R329–R334.
20. P Silva et al: Somatostatin inhibits CNP-induced stimulation of shark rectal gland. Bull Mt Desert Island Biol Lab 1991; 40: 25–29.
21. G Tulipano and S Schulz. Novel insights in somatostatin receptor physiology. Eur J Endocrinol 2007; 156: S3–S11.
22. J Ayuk, SE Stewart, PM Stewart, MC Sheppard. Long-term safety and efficacy of depot long-acting somatostatin analogs for the treatment of acromegaly. J Clin Endocrinol Metab 2002; 87: 4142–4146.
23. C Susini, L Buscail. Rationale for the use of somatostatin analogs as antitumor agents. Ann Oncol 2006; 17: 1733–1742.
24. Masyuk T, Masyuk A, Torres V, Harris P, Larusso N. Octreotide inhibits hepatic cystogenesis in a rodent model of polycystic liver disease by reducing cholangiocyte adenosine 3',5'- cyclic monophosphate. Gastroenterolgy 2006; 123: 1104-1116.

25. Antiga L, Piccinelli M, Fasolini G, Ene-Iordache B, Ondei P, Bruno S, Remuzzi G, Remuzzi Computed tomography evaluation of ADPKD progression: a progress report. Clin J Am Soc Nephrol 2006; 1: 754-760.
26. Feelders R, Hofland L, van Aken M, Neggers S, Lamberts S, de Herder W and van der Lely AJ. Drugs 2009, 69 (16):2207-26.
27. Caroli A, Antiga L, Cafaro M, Fasolini G, Remuzzi A, Remuzzi G and Ruggenenti P .Clin J Am Soc Nephrol. 2010, 5:783-9.
28. O'Brien P, Fleming J. A multiple testing procedure for clinical trials. Biometrics 1979; 35:549-556.

APPENDIX 1 - FLOWCHART

		TREATMENT PHASE												
**Visit number**	**1**	**2**	**3**	**4**	**5**	**6**	**7**	**8**	**9**	**10**	**11**	**12**	**13**	**14**
Time[1]	-30/-1 d	0	3 m	6 m	9 m	12 m	15 m	18 m	21 m	24 m	27 m	30 m	33 m	36 m
Informed consent	x													
Baseline Data[2]	x													
Physical Examination		x	x	x	x	x	x	x	x	x	x	x	x	x
Vital Signs[3]		x	x	x	x	x	x	x	x	x	x	x	x	x
Blood Laboratory Examinations[4]		x	x	x	x	x	x	x	x	x	x	x	x	x
Spiral Computed Tomography[5]		x				x								x
Gallbladder Ultrasonography[6]		x	x	x	x	x	x	x	x	x	x	x	x	x
Kidney Ultrasonography[7]		x	x	x	x	x	x	x	x	x	x	x	x	x
24 Hours Urine Collection[8]		x		x		x		x		x		x		x
Urinary Analysis[9]		x	x	x	x	x	x	x	x	x	x	x	x	x
Iohexol Plasma Clearance[10]		x		x		x		x		x		x		x
Quality life (SF36) and SF HLQ		x				x				x				x
Eligibility Tests[11]	x													
Inclusion Criteria		x												
Exclusion Criteria		x												
Randomization		x												
Investigational Medicinal Product[12]		x	**Every 28 days**											
Concomitant Treatments	x	x	x	x	x	x	x	x	x	x	x	x	x	x
Adverse Events	x	x	x	x	x	x	x	x	x	x	x	x	x	x
IMP Permanently Discontinuation		(x)	(x)	(x)	(x)	(x)	(x)	(x)	(x)	(x)	(x)	(x)	(x)	x
Trial End Summary		(x)	(x)	(x)	(x)	(x)	(x)	(x)	(x)	(x)	(x)	(x)	(x)	x
Final Declaration		(x)	(x)	(x)	(x)	(x)	(x)	(x)	(x)	(x)	(x)	(x)	(x)	x

[1] **Time**: "d" means days and "m" means months.

[2] **Baseline data**: baseline data include written informed consent, patient data, life style, marital status, education, professional status, chilbearing potential, familiar history, previous diseases, and previous treatments.

[3] **Vital signs**: blood pressure and pulse rate will be measured at the dominant arm with the patient in sitting position (by a standard sphygmomanometer or other non-invasive device) using the average of 3 measures taken consecutively 2 minutes apart, after 10 rest minutes.

[4] **Blood laboratory examinations**:
- visits 3-5-7-9-11-13: creatinine, urea, sodium, potassium, calcium, phosphorus, AST, ALT, alkaline phosphatase, γ-GT, total bilirubin, direct bilirubin, glucose, uric acid, leukocytes, platelets, erythrocytes, hemoglobin, and hematocrit, serum total proteins, albumin.
- visits 4-8-12: the same scheduled for the visit 3 plus CPK, total cholesterol, LDL-cholesterol, HDL-cholesterol, triglycerides.
- visit 10: the same scheduled for the visit 4 plus HbA1c.
- visits 2-6-14: the same scheduled for the visit 10 plus IGF-1.

[5] **Spiral computed tomography**: total kidney volume, total renal and renal cyst volume, total renal parenchyma volume, total renal intermediate volume, and total liver and liver cyst volume.

[6] **Gallbladder ultrasonography**: gallbladder sand and stones.

[7] **Kidney ultrasonography**: renal stones.

[8] **24 hours urine collection**: volume, duration, proteins, sodium, urea, glucose, phosphorus, creatinine and creatinine clearance; endothelin excretion, and MCP-1 excretion (overnight) in a subgroup only.

[9] **Urinary analysis:** proteins, albumin, creatinine.

[10] **Iohexol plasma clearance**: glomerular filtration rate (GFR).

[11] **Eligibility tests**: blood laboratory examinations (creatinine, glucose and HbA1c), urinary examination (24-hours proteinuria), and kidney and liver ultrasonography. Blood and urinary examinations are still valid if performed within 3 months prior to visit 2. Kidney and liver ultrasonography is still valid if performed within 1 year prior to visit 2.

[12] **Investigational medicinal product (IMP)**: patients will receive intramuscular IMP every 28 days.
