# Supplementary material for: Octreotide-LAR in later-stage autosomal dominant polycystic kidney disease (ALADIN 2): A randomized, double-blind, placebo-controlled, multicenter trial
Source: PLoS Med. 2019 Apr 5;16(4):e1002777. doi: 10.1371/journal.pmed.1002777 (PMC6450618; doi:10.1371/journal.pmed.1002777)
Supplement: S1 Table — (DOCX) [file pmed.1002777.s007.docx]

**S1. Table** Demography and baseline clinical and laboratory characteristics of participants with and without concomitant risk factors (diabetes mellitus or proteinuria > 1 g/24 h) according to randomization to octreotide-LAR or placebo.

|  | **Concomitant Risk Factors YES** | | **Concomitant Risk Factors NO** | |
| --- | --- | --- | --- | --- |
|  | ***Octreotide-LAR (n=16)*** | ***Placebo***  ***(n=14)*** | ***Octreotide-LAR (n=35)*** | ***Placebo***  ***(n=35)*** |
| Gender *(M/F)* | 11/5 | 8/6 | 20/15 | 18/17 |
| Age *(y)* | 44.0 ± 12.0 | 51.6 ± 6.2 | 49.6 ± 9.0 | 49.3 ± 10.3 |
| Weight *(kg)* | 86.8 ± 19.0 | 76.9 ± 15.5 | 75.1 ± 13.5 | 76.3 ± 13.8 |
| Height (*cm*) | 173.9 ± 9.1 | 170.0 ± 9.6 | 172.9 ± 10.5 | 171.0 ± 11.4 |
| Blood Pressure (*mmHg*) |  |  |  |  |
| Systolic | 147.1 ± 11.8 | 135.8± 15.3 | 132.0 ± 13.9 | 131.0 ± 12.3 |
| Diastolic | 84.1 ± 6.4 | 82.0± 6.6 | 81.3 ± 9.9 | 83.5 ± 9.0 |
| MAP | 105.1 ± 5.4 | 99.9 ± 7.9 | 98.2 ± 10.5 | 99.3 ± 9.2 |
| Total Cholesterol (*mg/dL*) | 183.2 ± 38.9 | 166.6 ± 41.0 | 196.5 ± 36.3 | 193.8 ± 35.1 |
| LDL Cholesterol (*mg/dL*) | 114.5 ± 40.6 | 93.7 ± 39.2 | 117.7 ± 26.2 | 116.8 ± 30.5 |
| Triglycerides (*mg/dL*) | 142.8 ± 60.6 | 111.5 ± 44.4 | 110.5 ± 61.1 | 122.6 ± 61.9 |
| S. Glucose (*mg/dL*) | 86.0 ± 10.4 | 81.8 ± 11.5 | 89.6 ± 10.8 | 90.0 ± 11.6 |
| S. Phosphorus (*mg/dL*) | 3.8 ± 0.5 | 4.0 ± 0.8 | 3.6 ± 0.6 | 3.8 ± 0.4 |
| S. Calcium (*mg/dL*) | 8.8 ± 0.5 | 8.9 ± 0.6 | 9.3 ± 0.5 | 9.4 ± 0.5 |
| Hemoglobin (*g/dL*) | 12.1 ± 1.4 | 11.9 ± 1.2 | 12.5 ± 1.5 | 12.2 ± 1.2 |
| S. Albumin (*g/dL*) | 4.3 ± 0.4 | 4.4 ± 0.3 | 4.0 ± 0.4 | 4.0 ± 0.4 |
| S. Creatinine (*mg/dL*) | 3.1 ± 1.2 | 3.2 ± 1.2 | 2.3 ± 0.5 | 2.5 ± 0.7 |
| GFR (*mL/min/1.73 m^2^*)* | 26.8 [23.0 to 36.6] | 20.5 [18.5 to 32.9] | 33.5 [27.7 to 36.7] | 31.7 [23.7 to 38.4] |
| eGFR (*mL/min/1.73 m^2^*)† | 23.6 [17.9 to 28.9] | 20.9 [17.0 to 28.3] | 28.7 [25.2 to 32.2] | 26.6 [21.8 to 33.2] |
| Ur. Proteins (*mg/24h*) | 850 [252 to 1270] | 253 [141 to 1625] | 210 [130 to 390] | 265 [130 to 430] |
| Ur. Albumin (*µg/mL*) | 155.0 [52.9 to 392.0] | 18.9 [12.9 to 215.0] | 40.8 [21.0 to 68.7] | 33.5 [12.7 to 85.4] |
| Ur. Alb-to-Cre ratio (*mg/g*) | 326.5 [107.9 to 583.0] | 51.1 [30.4 to 387.3] | 66.8 [34.5 to 123.3] | 42.6 [25.5 to 170.6] |
| Osmolality *(mmol/kg)* | 365.5 [269.9 to 507.3] | 356.5 [275.2 to 416.4] | 261.0 [217.2 to 340.2] | 298.0 [253.8 to 406.6] |
| TKV (*mL*) | 2368 [1697 to 3588] | 2631 [2248 to 3858] | 2339 [2006 to 4111] | 2441 [1873 to 3836] |
| htTKV (m*L/m*) | 1429 [999 to 2038] | 1498 [1310 to 2185] | 1344 [1158 to 2418] | 1597 [1082 to 2380] |

Data are mean ± SD, median [IQR] or counts. eGFR=estimated glomerular filtration rate. GFR=glomerular filtration rate. LAR=long-acting release. MAP=mean arterial pressure. TKV=total kidney volume. htTKV=height adjusted TKV. *Measured by iohexol plasma clearance. †Estimated by the four-variable equation from Modification of Diet in Renal Disease study.
