# Supplementary material for: Octreotide-LAR in later-stage autosomal dominant polycystic kidney disease (ALADIN 2): A randomized, double-blind, placebo-controlled, multicenter trial
Source: PLoS Med. 2019 Apr 5;16(4):e1002777. doi: 10.1371/journal.pmed.1002777 (PMC6450618; doi:10.1371/journal.pmed.1002777)
Supplement: S2 Table — (DOCX) [file pmed.1002777.s008.docx]

**S2**. **Table** Twenty-four-hour urinary protein excretion at baseline and over the whole follow-up period according to randomization to octreotide-LAR or placebo in the study group as a whole (overall) and in the 2 subgroups with CKD stage 3b and 4 considered separately.

|  | **Overall** | | | | **CKD stage 3b** | | | | **CKD stage 4** | | | |
| --- | --- | --- | --- | --- | --- | --- | --- | --- | --- | --- | --- | --- |
|  | **Octreotide-LAR** *(n=51)* | | **Placebo**  *(n=49)* | | **Octreotide-LAR**  *(n=20)* | | **Placebo**  *(n=17)* | | **Octreotide-LAR**  *(n=31)* | | **Placebo**  *(n=32)* | |
| Ur. Proteins (*mg/24h*) |  |  | |  | |  | |  | |  | |  |
| Baseline | 268 [135 to 805] | 260 [130 to 460] | | 180 [130 to 330] | | 160 [90 to 300] | | 390 [150 to 880] | | 320 [180 to 570] | |  |
| Follow-up | 350 [173 to 835] | 420 [160 to 710] | | 225 [160 to 465] | | 197 [135 to 430] | | 490 [235 to 916] | | 508 [325 to 750] | |  |
| Follow-up vs baseline | 15.0 [-87.0 to 205.0] | 90.0 [-25.0 to 255.0]***** | | 15.0 [-87.0 to 160.0] | | 40.0 [-20.0 to 117.5] | | 32.3 [-100.0 to 328.0] | | 150.0 [-25.0 to 290.0] **^¥^** | |  |

Data are median [IQR]. Follow-up vs baseline by Wilcoxon signed test. **^*^***P=0.0003* vs baseline. **^¥^***P=0.0016* vs baseline. All other comparisons performed by non-parametric ANCOVA.
