# Supplementary material for: Octreotide-LAR in later-stage autosomal dominant polycystic kidney disease (ALADIN 2): A randomized, double-blind, placebo-controlled, multicenter trial
Source: PLoS Med. 2019 Apr 5;16(4):e1002777. doi: 10.1371/journal.pmed.1002777 (PMC6450618; doi:10.1371/journal.pmed.1002777)
Supplement: S3 Table — (DOCX) [file pmed.1002777.s009.docx]

| **S3. Table** Concomitant medications at baseline and during follow-up according to randomization to octreotide-LAR or placebo. | | | | | |
| --- | --- | --- | --- | --- | --- |
|  | | **Baseline** | | **Follow-up** | |
| ***Patients on Pharmacological Medications – n (%)*** | | **Octreotide-LAR (N=51)** | **Placebo (N=49)** | **Octreotide-LAR (N=51)** | **Placebo (N=49)** |
| *Antihypertensive agents* | |  |  |  |  |
| - Any | | 50 (98%) | 48 (98%) | 50 (98%) | 49 (100%) |
| - Diuretics | | 8 (16%) | 9 (18%) | 11 (22%) | 16 (33%) |
| - Angiotensin-converting-enzyme inhibitors | | 27 (53%) | 31 (63%) | 32 (63%) | 35 (71%) |
| - Angiotensin receptor blockers | | 19 (37%) | 19 (39%) | 22 (43%) | 21 (43%) |
| - Calcium-channel blockers | | 18 (35%) | 12 (24%) | 30 (59%) | 30 (61%) |
| - Beta blockers | | 14 (27%) | 16 (33%) | 16 (31%) | 19 (39%) |
| - Sympatholytic agents | | 15 (29%) | 5 (10%) | 26 (51%) | 17 (35%) |
| *Lipid-lowering agents* | |  |  |  |  |
| - Any | | 12 (24%) | 17 (35%) | 25 (49%) | 27 (55%) |
| - Statin monotherapy | | 9 (18%) | 12 (24%) | 21 (41%) | 22 (45%) |
| - Omega-3 fatty acid monotherapy | | 3 (6%) | 3 (6%) | 2 (4%) | 2 (4%) |
| - Statin and Omega-3 fatty acid | | 0 | 2 (4%) | 2 (4%) | 3 (6%) |
| *Metabolic treatments* | |  |  |  |  |
| - Allopurinol | | 19 (37%) | 24 (49%) | 36 (71%) | 36 (73%) |
| - Febuxostat | | 2 (4%) | 1 (2%) | 7 (14%) | 7 (14%) |
| - Calcitriol (1,25-dihydroxyvitamin D3) | | 6 (12%) | 12 (24%) | 29 (57%) | 16 (33%) * |
| - Paricalcitol (19-nor-1,25-OH 2-vitamin D2) | | 0 | 1 (2%) | 2 (4%) | 1 (2%) |
| - Vitamin D_3_ (cholecalciferol), 25-OH Vitamin D (calcifediol) | | 4 (8%) | 4 (8%) | 9 (18%) | 6 (12%) |
| - Calcium (carbonate, acetate, gluconate) | | 3 (6%) | 3 (6%) | 28 (55%) | 21 (43%) |
| - Sevelamer | | 0 | 0 | 7 (14%) | 11 (22%) |
| - Sodium bicarbonate | | 14 (27%) | 12 (24%) | 39 (76%) | 12 (24%) ° |
| - Erythropoietin | | 2 (4%) | 1 (2%) | 13 (25%) | 17 (35%) |
| *Antiplatelet agents* | | 2 (4%) | 4 (8%) | 6 (12%) | 6 (12%) |
|  |  | |  | | |

Chi square o Fisher’s exact test: **P*<0.05, °*P*<0.0001 vs. Octeotride-LAR.
