# Supplementary material for: Octreotide-LAR in later-stage autosomal dominant polycystic kidney disease (ALADIN 2): A randomized, double-blind, placebo-controlled, multicenter trial
Source: PLoS Med. 2019 Apr 5;16(4):e1002777. doi: 10.1371/journal.pmed.1002777 (PMC6450618; doi:10.1371/journal.pmed.1002777)
Supplement: S4 Table — (DOCX) [file pmed.1002777.s010.docx]

**S4. Table** TKV at baseline, 1-year follow-up, and 3-year follow-up, as well as total and chronic measured GFR slopes in patients without diabetes mellitus and without proteinuria > 1 g/24 h at baseline according to treatment with octreotide-LAR or placebo.

|  |  | **Octreotide-LAR** | | | **Placebo** | | | **P value** |
| --- | --- | --- | --- | --- | --- | --- | --- | --- |
|  |  | **Baseline** | **1 year** | **3 year** | **Baseline** | **1 year** | **3 year** |  |
| **TKV** | Median [IQR] (mL) | 2338.9  [2005.6-4110.9] | 2504.1  [2047.0-4567.9] | 2950.7  [2337.3-5548.7] | 2441.0  [1872.6-3835.7] | 2732.8  [2197.1-3896.2] | 3613.8  [2773.9-4899.8] |  |
|  | Absolute change (mL) | -- | 157.5  [36.3-484.0] | 602.0  [339.1-1145.1] | -- | 277.7  [118.6-458.0] | 940.6  [614.7-1313.3] |  |
|  | Octreotide-LAR vs placebo difference (mL) |  | -90.8  [-186.4 to 4.7] | -410.1  [-714.3 to -105.8] |  |  |  | 0.062^*^;  0.0082^¥^ |
|  |  |  |  |  |  |  |  |  |
| **Total Slope**  **0-3 yrs** | Median [IQR], mL/min/1.73m^2^ per year |  |  | -3.94  [-5.19 to -2.89] |  |  | -4.40  [-6.03 to -2.03] |  |
|  | Octreotide-LAR vs placebo difference, mL/min/1.73m^2^ per year |  |  | 0.23  [-1.10 to 1.55] |  |  |  | 0.655 |
|  |  |  |  |  |  |  |  |  |
| **Chronic slope**  **6 mo-3 yrs** | Median [IQR],  mL/min/1.73m^2^ per year |  |  | -3.58  [-4.40 to -2.38] |  |  | -4.77  [-6.53 to -2.40] |  |
|  | Octreotide-LAR vs placebo difference, mL/min/1.73m^2^ per year |  |  | 0.88  [-0.52 to 2.27] |  |  |  | 0.181 |

Data are Median [IQR]. ^*^ Octreotide-LAR versus placebo at 1 year. ^¥^ Octreotide-LAR versus placebo at 3 years.
