# Supplementary material for: Octreotide-LAR in later-stage autosomal dominant polycystic kidney disease (ALADIN 2): A randomized, double-blind, placebo-controlled, multicenter trial
Source: PLoS Med. 2019 Apr 5;16(4):e1002777. doi: 10.1371/journal.pmed.1002777 (PMC6450618; doi:10.1371/journal.pmed.1002777)
Supplement: S5 Table — (DOCX) [file pmed.1002777.s011.docx]

**S5. Table** eGFR at baseline, 6 months, 1 year, 2 years, and 3 years in the study group as a whole (overall), and in the 2 subgroups with CKD stage 3 and 4 considered separately, according to treatment with octreotide-LAR or placebo*.*

|  | |  | **Octreotide-LAR** | | | | | **Placebo** | | | | | |
| --- | --- | --- | --- | --- | --- | --- | --- | --- | --- | --- | --- | --- | --- |
|  | |  | Baseline  (n=51) | 6 mo  (n=44) | 1 yr  (n=45) | 2 yrs  (n=37) | 3 yrs  (n=35) | Baseline  (n=49) | 6 mo  (n=46) | 1 yr  (n=46) | 2 yrs  (n=39) | 3 yrs  (n=35) |  |
| **Overall** | *Actual value** | | 27.9  [23.5 to 32.1] | 25.5  [19.5 to 30.1] | 22.5  [17.3 to 27.7] | 17.7  [13.2 to 22.8] | 14.9  [11.3 to 20.2] | 25.8  [19.5 to 33.2] | 22.2  [17.3 to 31.7] | 20.2  [14.7 to 28.1] | 16.8  [11.4 to 25.6] | 15.0  [7.5 to 24.4] | |
|  | Total Slope°  0-3 yrs | |  |  |  |  | -5.2  [-6.2 to -3.5] |  |  |  |  | -4.7  [-6.6 to -3.2] | |
|  | Chronic slope°  6 mo-3 yrs | |  |  |  |  | -4.4  [-5.9 to -3.6] |  |  |  |  | -4.1  [-5.9 to -2.8] | |
| **CKD**  **stage 3b** | *Actual value** | | 33.6  [31.3 to 38.9] | 31.2  [29.2 to 34.5] | 29.9  [25.7 to 33.8] | 26.2  [19.5 to 29.9] | 19.6  [14.9 to 22.8] | 35.6  [32.3 to 38.4] | 33.4  [30.5 to 37.4] | 29.3  [27.6 to 37.0] | 25.9  [22.1 to 30.3] | 21.5  [16.7 to 32.6] | |
|  | Total Slope°  0-3 yrs | |  |  |  |  | -5.7  [-6.2 to -4.7] |  |  |  |  | -4.1  [-5.2 to -2.6] | |
|  | Chronic slope°  6 mo-3 yrs | |  |  |  |  | -4.6  [-5.9 to -4.1] |  |  |  |  | -3.8  [-5.1 to -2.4] | |
| **CKD**  **stage 4** | *Actual value** | | 24.6  [20.6 to 27.3] | 20.8  [17.7 to 25.7] | 17.7  [14.3 to 21.6] | 14.8  [11.9 to 18.5] | 12.3  [8.8 to 16.0] | 21.8  [18.0 to 25.8] | 18.3  [15.7 to 21.7] | 16.2  [13.9 to 20.1] | 12.5  [10.2 to 16.8] | 7.8  [7.2 to 15.0] | |
|  | Total Slope°  0-3 yrs | |  |  |  |  | -4.7  [-6.6 to -3.3] |  |  |  |  | -5.1  [-6.8 to -3.2] | |
|  | Chronic slope°  6 mo-3 yrs | |  |  |  |  | -4.0  [-6.1 to -2.9] |  |  |  |  | -4.2  [-5.9 to -3.1] | |

Data are median [IQR]. eGFR=estimated glomerular filtration rate, * mL/min/1.73m^2^; ° mL/min/1.73m^2^ per year.
