# Supplementary material for: Octreotide-LAR in later-stage autosomal dominant polycystic kidney disease (ALADIN 2): A randomized, double-blind, placebo-controlled, multicenter trial
Source: PLoS Med. 2019 Apr 5;16(4):e1002777. doi: 10.1371/journal.pmed.1002777 (PMC6450618; doi:10.1371/journal.pmed.1002777)
Supplement: S6 Table — (DOCX) [file pmed.1002777.s012.docx]

**S6. Table** Patients’ characteristics at baseline, 1 year, and 3 years according to randomization to octreotide-LAR or placebo treatment.

|  | **Octreotide-LAR (n=51)** | | | **Placebo (n=49)** | | | |
| --- | --- | --- | --- | --- | --- | --- | --- |
|  | ***Baseline*** | ***1 Year*** | ***3 Years*** |  | ***Baseline*** | ***1 Year*** | ***3 Years*** |
| *Clinical parameters* |  |  |  |  |  |  |  |
| Weight (Kg) | 77.2 (14.6) | 77.1 (13.7) | 74.5 (15.0)* |  | 76.4 (14.1) | 77.2 (13.8) | 73.6 (12.8) |
| SBP *(mmHg)* | 134.9 (15.4) | 133.3 (15.1) | 132.6 (13.6) |  | 132.3 (13.2) | 130.3 (12.2) | 130.3 (6.9) |
| DBP *(mmHg)* | 81.8 (9.3) | 81.5 (9.2) | 82.4 (6.1) |  | 83.1 (8.4) | 82.3 (6.0) | 82.9 (8.0) |
| MAP *(mmHg)* | 99.5 (10.3) | 98.7 (10.1) | 99.1 (7.8) |  | 99.5 (8.9) | 98.3 (6.5) | 98.7 (6.7) |
| *Hematochemistry* |  |  |  |  |  |  |  |
| Serum Creatinine *(mg/dL)* | 2.6 (0.9) | 3.4 (1.7)*** | 4.9 (2.8)*** |  | 2.7 (0.9) | 3.3 (1.4)*** | 4.7 (2.4)*** |
| Urea *(mg/dL)* | 87.4 (23.8) | 110.4 (38.0)*** | 145.0 (54.1)*** |  | 92.2 (31.8) | 110.0 (43.3)** | 128.6 (54.6)** |
| Sodium *(mEq/L)* | 140.5 (2.7) | 140.8 (2.9) | 140.5 (2.6) |  | 140.8 (8.2) | 140.3 (2.4) | 140.3 (2.5) |
| Potassium *(mEq/L)* | 4.4 (0.6) | 4.7 (0.6)* | 4.8 (0.6)** |  | 4.5 (0.6) | 4.6 (0.6)* | 4.7 (0.4)** |
| Calcium *(mg/dL)* | 9.1 (0.5) | 9.1 (0.5) | 9.0 (0.7)** |  | 9.2 (0.6) | 9.4 (0.4)° | 9.0 (1.3) |
| Phosphorus *(mg/dL)* | 3.7 (0.5) | 4.0 (0.8)** | 4.4 (1.0)*** |  | 3.8 (0.6) | 4.1 (0.8) | 4.5 (1.1)** |
| GOT (*IU/L)* | 18.4 (4.9) | 17.5 (5.1) | 16.3 (4.4)* |  | 19.1 (6.0) | 17.7 (4.0) | 16.8 (5.1)* |
| GPT *(IU/L)* | 18.2 (7.2) | 17.6 (16.3) | 14.6 (6.2)** |  | 19.1 (8.0) | 17.0 (8.2) | 14.2 (5.2**) |
| GGT *(IU/L)* | 27.0 (21.0) | 29.3 (25.9) | 24.5 (17.8) |  | 25.2 (19.4) | 25.2 (22.7) | 23.8 (14.2) |
| Alkaline phosphatase *(IU/L)* | 60.7 (20.0) | 78.9 (52.4) | 71.6 (17.2)** |  | 67.9 (37.4) | 73.2 (44.6)* | 73.1 (41.3) |
| **S6 Table (cont.)** | |  |  |  |  |  |  |
| Total bilirubin *(mg/dL)* | 0.62 (0.29) | 0.57 (0.22)* | 0.57 (0.23) |  | 0.61 (0.41) | 0.60 (0.36) | 0.59 (0.35) |
| Direct bilirubin *(mg/dL)* | 0.13 (0.08) | 0.11 (0.05) | 0.11 (0.06) |  | 0.12 (0.07) | 0.12 (0.07) | 0.13 (0.08) |
| CPK *(IU/L)* | 136.3 (100.1) | 142.5 (116.9) | 132.3 (96.0) |  | 121.7 (67.5) | 123.3 (68.0) | 130.2 (72.7) |
| Glucose *(mg/dL)* | 88.5 (10.7) | 95.7 (12.0)*** | 96.0 (13.7)* |  | 87.7 (12.0) | 88.6 (10.5)°° | 87.4 (8.4)°° |
| Hba1c (*%)* | 4.2 (1.0) | 6.2 (1.5)** | 5.5 (0.4)*** |  | 4.2 (1.0) | 6.0 (1.4) | 5.1 (0.5)*** |
| Uric acid *(mg/dL)* | 6.8 (1.5) | 6.2 (1.5)** | 5.8 (1.2)** |  | 6.5 (1.1) | 6.0 (1.4) | 5.5 (1.3)** |
| Total cholesterol *(mg/dL)* | 192.4 (37.2) | 169.4 (29.9)** | 161.8 (24.0)*** |  | 186.3 (38.4) | 180.3 (46.9) | 167.8 (35.2)** |
| LDL cholesterol *(mg/dL)* | 116.8 (30.4) | 93.5 (22.2)** | 84.7 (19.8)*** |  | 110.7 (34.1) | 110.1 (45.5)° | 94.9 (32.2)** |
| HDL cholesterol *(mg/dL)* | 49.7 (13.2) | 50.0 (15.1) | 50.3 (16.9) |  | 49.7 (15.9) | 47.3 (11.8) | 48.8 (12.8) |
| Triglycerides *(mg/dL)* | 120.4 (62.1) | 102.7 (37.5)* | 103.8 (47.1) |  | 119.5 (57.4) | 120.0 (59.6) | 112.8 (56.7) |
| Total proteins *(g/dL)* | 6.8 (0.5) | 7.0 (0.5) | 6.9 (0.6) |  | 6.9 (0.6) | 7.0 (0.5) | 6.9 (0.4) |
| Albumin *(g/dL)* | 4.1 (0.4) | 4.1 (0.4) | 4.1 (0.4) |  | 4.1 (0.4) | 4.1 (0.5) | 4.1 (0.4) |
| IGF1 *(ng/mL)* | 153.0 (56.0) | 111.4 (44.1)** | 136.3 (59.7) |  | 163.1 (77.4) | 138.3 (61.1)° | 139.8 (61.3) |
| *Hemochrome* |  |  |  |  |  |  |  |
| Leukocytes *(*10^3^/µL)* | 5.9 (1.5) | 6.6 (2.8) | 5.9 (1.3) |  | 5.8 (1.7) | 5.8 (1.5) | 5.4 (1.0) |
| Platelets *(*10^3^/µL)* | 196.9 (58.0) | 188.8 (60.9) | 178.7 (51.2) |  | 195.6 (50.1) | 190.6 (46.9) | 182.2 (44.2)* |
| Erythrocytes *(*10^3^/µL)* | 4.2 (0.5) | 4.1 (0.5)** | 3.9 (0.5)*** |  | 4.1 (0.4) | 4.0 (0.4)* | 3.9 (0.5)* |
| Hemoglobin *(g/dL)* | 12.4 (1.5) | 12.0 (1.2)** | 11.4 (1.4)*** |  | 12.1 (1.2) | 11.8 (1.2)* | 11.2 (1.5)** |
| Hematocrit *(%)* | 37.2 (4.0) | 36.9 (3.7) | 35.3 (4.5)** |  | 36.4 (3.5) | 35.9 (3.3) | 34.8 (4.6)* |
| *24 Hours urine collection* |  |  |  |  |  |  |  |
| Volume (*mL)* | 2403 [1827 to 2815] | 2285 [1866 to 2598] | 2000 [1750 to 2558]* |  | 2500 [1900 to 3000] | 2500 [1800 to 2961] | 2232 [1800 to 2970] |
| Proteins (*g/24h)* | 268 [ 135 to 804] | 340 [170 to 920] | 480 [ 190 to 820] |  | 260 [ 130 to 460] | 340 [130 to 580]* | 580 [180 to 968]*** |
|  |  |  |  |  |  |  |  |
|  |  |  |  |  |  |  |  |
| **S6 Table (cont.)** | |  |  |  |  |  |  |
| Creatinine (*mg/24h)* | 1293 [1054 to 1600] | 1279 [956 to 1452] | 1136 [817 to 1439]** |  | 1271 [1085 to 1601] | 1187 [911 to 1601]* | 1125 [850 to 1353]*** |
| Creatinine clearance (mL/min) | 37.99 [29.87 to 43.42] | 26.71 [17.05 to 33.0] | 18.67 [14.95 to 26.94] |  | 35.00 [25.40 to 43.54] | 27.16 [17.74 to 40.27] | 19.43 [15.59 to 27.12] |
| Sodium (*mEq/24h)* | 141.7 [111.3 to 190.0] | 146.8 [114.2 to 185.0] | 133.8 [114.0 to 155.6]* |  | 154.9 [125.2 to 205.0] | 166.0 [116.5 to 198.3] | 157.0 [127.8 to 181.4] |
| Urea (*g/24h)* | 18.9 [15.9 to 24.1] | 19.5 [14.0 to 23.6] | 16.2 [12.6 to 20.3]** |  | 19.5 [16.0 to 26.0] | 18.5 [14.2 to 23.9]* | 16.5 [11.7 to 21.0]** |
| Glucose (*mg/24h)* | 84.1 [31.8 to 193.0] | 75.3 [30.0 to 105.2] | 129.8 [63.3 to 300]* |  | 90.5 [58.3 to 236.5] | 102.0 [50.0 to 212.3] | 154.0 [64.7 to 420.0]* |
| Phosphate (*mg/24h)* | 603.7 [443.9 to 752.4] | 591.6 [494.5 to 771.1] | 508.2 [420.0 to 612.5]** |  | 606.4 [515.4 to 728.5] | 563.1 [442.2 to 715.2]** | 506.0 [372.2 to 643.8]** |
| Osmolality (*mOsm/L)* | 279.2 [217.8 to365.5] | 289.4 [230.8 to 381.2] | 266.5 [221.3 to 306.4] |  | 313.7 [253.8 to 407.6] | 341.0 [245.1 to 396.4] | 317.3 [252.2 to 384.5]° |
| *Spot morning urine* |  |  |  |  |  |  |  |
| Proteins (*mg/dL)* | 11.4 [7.0 to 23.6] | 14.9 [8.4 to 39.3]* | 18.1 [9.0 to 28.7]** |  | 9.1 [6.0 to 18.6] | 16.5 [7.2 to 24.0] | 25.2 [8.7 to 43.8]** |
| Albumin (*μg/mL)* | 50.7 [21.0 to 118.1] | 42.5 [33.3 to 210.5]* | 74.3 [33.5 to 111.0]* |  | 28.3 [12.8 to 96.2] | 57.7 [18.4 to 130.0] | 90.0 [53.5 to 150.0]** |
| Creatinine (*mg/dL)* | 60.2 [45.0 to 70.4] | 58.3 [48.4 to 70.4] | 49.3 [41.8 to 59.6]* |  | 59.0 [50.1 to 73.1] | 55.6 [46.7 to 69.3]* | 52.7 [44.0 to 60.0]** |
| Protein-to-creatinine ratio *(mg/g)* | 0.24 [0.12 to 0.55] | 0.26 [0.13 to 0.73]* | 0.40 [0.15 to 0.62]** |  | 0.15 [0.10 to 0.37] | 0.30 [0.15 to 0.44]** | 0.39 [0.24 to 0.81]** |
| Albumin-to-creatinine ratio *(mg/g)* | 77.3 [35.9 to 225.9] | 80.0 [51.9 to 364.4]* | 130.6 [54.2 to 286.0]* |  | 45.4 [25.5 to 181.9] | 108.1 [35.0 to 211.0]** | 162.2 [102.8 to 260.3]** |

Data are mean ± SD or median [IQR]; ° *P*<0.05, °° *P*<0.01 vs octreotide-LAR, adjusted for baseline value (ANCOVA); ^*^ *P*<0.05, ^**^ *P*<0.01, *** *P*<0.001 vs baseline (paired t-test); SBP=systolic blood pressure. DBP=diastolic blood pressure. MAP=mean arterial pressure. AST=aspartate aminotransferase. ALT=alanine aminotransferase. GGT=γ-glutamyl-transpeptidase. LDL=low-density lipoprotein. HDL=high-density lipoprotein.
