# Supplementary material for: Octreotide-LAR in later-stage autosomal dominant polycystic kidney disease (ALADIN 2): A randomized, double-blind, placebo-controlled, multicenter trial
Source: PLoS Med. 2019 Apr 5;16(4):e1002777. doi: 10.1371/journal.pmed.1002777 (PMC6450618; doi:10.1371/journal.pmed.1002777)
Supplement: S7 Table — (DOCX) [file pmed.1002777.s013.docx]

**S7. Table** Number (%) of patients with at least 1 non-serious adverse event in the study group as a whole (overall) and according to randomization to octreotide-LAR or placebo treatment.

| **Adverse Events** | **Overall**  (n=100) | | **Octreotide-LAR**  (n=51) | | **Placebo**  (n=49) | |
| --- | --- | --- | --- | --- | --- | --- |
| ***Cardiovascular*** |  | |  | |  | |
| Calf swelling | 15 | 15.0% | 9 | 17.6% | 6 | 12.2% |
| Hydrosaline retention | 1 | 1.0% | 0 | - | 1 | 2.0% |
| Pericardial effusion | 3 | 3.0% | 1 | 2.0% | 2 | 4.1% |
| Bilateral pleural effusion | 2 | 2.0% | 1 | 2.0% | 1 | 2.0% |
| Aortic calcification | 1 | 1.0% | 1 | 2.0% | 0 | 0.0% |
| Claudicatio intermittents | 1 | 1.0% | 1 | 2.0% | 0 | - |
| Calf vein thrombosis | 1 | 1.0% | 1 | 2.0% | 0 | - |
| Left ventricular hypertrophy | 2 | 2.0% | 0 | - | 2 | 4.1% |
| Left atrial enlargement | 1 | 1.0% | 0 | - | 1 | 2.0% |
| Atrial fibrillation | 2 | 2.0% | 0 | - | 2 | 4.1% |
| Palpitations | 5 | 5.0% | 3 | 5.9% | 2 | 4.1% |
| Hypotension | 2 | 2.0% | 1 | 2.0% | 1 | 2.0% |
| Precordial pain | 2 | 2.0% | 0 | - | 2 | 4.1% |
| ***Renal*** |  |  |  |  |  |  |
| Acute renal failure | 9 | 9.0% | 4 | 7.8% | 5 | 10.2% |
| Renal colic, lithiasis | 10 | 10.0% | 7 | 13.7% | 3 | 6.1% |
| Hematuria | 15 | 15.0% | 6 | 11.8% | 9 | 18.4% |
| Urinary tract infection | 11 | 11.0% | 6 | 11.8% | 5 | 10.2% |
| Renal cyst infection or rupture | 7 | 7.0% | 1 | 2.0% | 6* | 12.2% |
| Asymptomatic bacteriuria, dysuria | 4 | 4.0% | 2 | 3.9% | 2 | 4.1% |
| **Gastrointestinal** |  |  |  |  |  |  |
| Diarrhea | 20 | 20.0% | 15 | 29.4% | 5* | 10.2% |
| Abdominal pain, bloating | 20 | 20.0% | 12 | 23.5% | 10 | 20.4% |
| Gastritis, nausea, dyspepsia, vomiting | 13 | 13.0% | 4 | 7.8% | 9 | 18.4% |
| Cholelithiasis | 4 | 4.0% | 3 | 5.9% | 1 | 2.0% |
|  |  |  |  |  |  |  |
| **S7. Table (cont.)** |  |  |  |  |  |  |
| Biliary sand | 8 | 8.0% | 8 | 15.7% | 0** | - |
| Gallbladder polyp | 3 | 3.0% | 2 | 3.9% | 1 | 2.0% |
| Liver steatosis | 3 | 3.0% | 1 | 2.0% | 2 | 4.1% |
| Hepatic cyst rupture, hemorrhagic hepatic cyst | 2 | 2.0% | 0 | - | 2 | 4.1% |
| Constipation | 2 | 2.0% | 0 | - | 2 | 4.1% |
| Flatulence | 1 | 1.0% | 1 | 2.0% | 0 | - |
| Colon diverticulosis | 2 | 2.0% | 1 | 2.0% | 1 | 2.0% |
| Other gastrointestinal events | 9 | 9.0% | 6 | 11.8% | 3 | 6.1% |
| **Metabolic, endocrine, hematological** |  |  |  |  |  |  |
| Chronic kidney disease-mineral and bone disorder (CKD-MBD) | 36 | 36.0% | 19 | 37.3% | 17 | 34.7% |
| Secondary hyperparathyroidism | 43 | 43.0% | 25 | 49.0% | 18 | 36.7% |
| Metabolic acidosis | 40 | 40.0% | 23 | 45.1% | 17 | 34.7% |
| Anaemia | 34 | 34.0% | 11 | 21.6% | 23** | 46.9% |
| Hyperuricemia | 25 | 25.0% | 16 | 31.4% | 9 | 18.4% |
| Dyslipidemia | 15 | 15.0% | 10 | 19.6% | 5 | 10.2% |
| Hyperkalemia | 4 | 4.0% | 3 | 5.9% | 1 | 2.0% |
| Hypokalemia | 1 | 1.0% | 0 | - | 1 | 2.0% |
| Hyponatremia | 1 | 1.0% | 1 | 2.0% | 0 | - |
| Hyperhomocysteinemia | 7 | 7.0% | 3 | 5.9% | 4 | 8.2% |
| Pancytopenia | 2 | 2.0% | 2 | 3.9% | 0 | 0.0% |
| High CPK levels | 2 | 2.0% | 1 | 2.0% | 1 | 2.0% |
| Benign cutaneous lymphoid infiltrate | 1 | 1.0% | 0 | - | 1 | 2.0% |
| Other metabolic, endocrine, hematological events | 6 | 6.0% | 4 | 7.8% | 2 | 4.1% |
| **Musculoskeletal** |  |  |  |  |  |  |
| Back pain, flank pain | 18 | 18.0% | 8 | 15.7% | 10 | 20.4% |
| Muscular pain, cramps | 18 | 18.0% | 9 | 17.6% | 9 | 18.4% |
| Fatigue | 18 | 18.0% | 10 | 19.6% | 8 | 16.3% |
| Site injection pain, site injection nodule | 10 | 10.0% | 7 | 13.7% | 3 | 6.1% |
| Osteoarthritis | 5 | 5.0% | 2 | 3.9% | 3 | 6.1% |
| Fracture, trauma, tendonitis, tendon tear | 8 | 8.0% | 2 | 3.9% | 6 | 12.2% |
| **S7. Table (cont.)** |  |  |  |  |  |  |
| Septic Arthritis | 1 | 1.0% | 1 | 2.0% | 0 | 0.0% |
| Discal hernia | 1 | 1.0% | 0 | - | 1 | 2.0% |
| Other musculoskeletal events | 2 | 2.0% | 1 | 2.0% | 2 | 4.1% |
| **Nervous system, psychiatric** |  |  |  |  |  |  |
| Depression, anxiety | 2 | 2.0% | 0 | - | 2 | 4.1% |
| Headache | 5 | 5.0% | 1 | 2.0% | 4 | 8.2% |
| Dizziness | 4 | 4.0% | 2 | 3.9% | 2 | 4.1% |
| Neuropathic pain | 2 | 2.0% | 0 | - | 2 | 4.1% |
| Tremor | 1 | 1.0% | 1 | 2.0% | 0 | - |
| Other nervous system events | 2 | 2.0% | 1 | 2.0% | 1 | 2.0% |
| **Respiratory** |  |  |  |  |  |  |
| Pharyngitis, rhinitis, common cold, cough | 11 | 11.0% | 4 | 7.8% | 7 | 14.3% |
| Acute Bronchitis | 9 | 9.0% | 4 | 7.8% | 5 | 10.2% |
| Pneumonia | 1 | 1.0% | 1 | 2.0% | 0 | - |
| **Gynecologic, urologic** |  |  |  |  |  |  |
| Ovarian cyst, follicle rupture | 1 | 1.0% | 0 | - | 1 | 2.0% |
| Vaginitis | 2 | 2.0% | 0 | - | 2 | 4.1% |
| Endometrial hyperplasia, endometrial polyps | 2 | 2.0% | 1 | 2.0% | 1 | 2.0% |
| Benign prostatic hyperplasia | 6 | 6.0% | 1 | 2.0% | 5 | 10.2% |
| Prostatitis | 1 | 1.0% | 0 | - | 1 | 2.0% |
| Impotence | 2 | 2.0% | 0 | - | 2 | 4.1% |
| Other urologic events | 3 | 3.0% | 2 | 3.9% | 1 | 2.0% |
| **Ocular, ear, oral, nasal** |  |  |  |  |  |  |
| Otitis | 1 | 1.0% | 0 | - | 1 | 2.0% |
| Tinnitus | 1 | 1.0% | 0 | - | 1 | 2.0% |
| Hyposmia | 1 | 1.0% | 0 | - | 1 | 2.0% |
| Degenerative maculopathy | 1 | 1.0% | 1 | 2.0% | 0 | - |
| Amblyopia | 1 | 1.0% | 0 | - | 1 | 2.0% |
| Other ocular, ear, labyrinth, oral, nasal events | 5 | 5.0% | 4 | 7.8% | 1 | 2.0% |
|  |  |  |  |  |  |  |
| **S7. Table (cont.)** |  |  |  |  |  |  |
| **Dermatologic** |  |  |  |  |  |  |
| Pruritus | 6 | 6.0% | 1 | 2.0% | 5 | 10.2% |
| Rash | 4 | 4.0% | 2 | 3.9% | 2 | 4.1% |
| Dermatitis | 3 | 3.0% | 1 | 2.0% | 2 | 4.1% |
| Onychomycosis | 2 | 2.0% | 0 | - | 2 | 4.1% |
| Folliculitis, forunculosis | 2 | 2.0% | 1 | 2.0% | 1 | 2.0% |
| Subcutaneous hematoma of the limbs | 2 | 2.0% | 0 | - | 2 | 4.1% |
| Alopecia | 1 | 1.0% | 1 | 2.0% | 0 | - |
| **Allergic reactions** |  |  |  |  |  |  |
| Allergic reaction to study drug | 2 | 2.0% | 1 | 2.0% | 1 | 2.0% |
| Non treatment-related allergic reactions | 5 | 5.0% | 3 | 5.9% | 2 | 4.1% |
| **Other infections** |  |  |  |  |  |  |
| Flu like syndrome | 15 | 15.0% | 6 | 11.8% | 9 | 18.4% |
| Fever (unspecified) | 9 | 9.0% | 5 | 9.8% | 4 | 8.2% |
| Herpes zoster | 1 | 1.0% | 1 | 2.0% | 0 | - |
| Herpes simplex | 1 | 1.0% | 1 | 2.0% | 0 | - |

Chi-square test or Fisher’s exact test; * *P*<0.05, ***P*<0.01 vs Placebo.
