# Supplementary material for: Octreotide-LAR in later-stage autosomal dominant polycystic kidney disease (ALADIN 2): A randomized, double-blind, placebo-controlled, multicenter trial
Source: PLoS Med. 2019 Apr 5;16(4):e1002777. doi: 10.1371/journal.pmed.1002777 (PMC6450618; doi:10.1371/journal.pmed.1002777)
Supplement: S1 Text — (DOCX) [file pmed.1002777.s014.docx]

**S1. Text Supplementary Methods**

**Study participants, monitoring and masking**

This phase 3, academic, randomized, parallel-group, double-blind, placebo-controlled trial enrolled patients with ADPKD referred to outpatient clinics of four hospitals in Italy (Azienda Socio-Sanitaria Territoriale Papa Giovanni XXIII, Bergamo; Università Federico II, Naples; Ospedale Ca' Foncello, Treviso; and Ospedale San Giovanni di Dio, Agrigento).

The study was co-ordinated and monitored by the Department of Renal Medicine of the Aldo e Cele Daccò Clinical Research Center for Rare Diseases (Istituto di Ricerche Farmacologiche Mario Negri IRCCS, Bergamo, Italy) according to Good Clinical Practice guidelines.

All study participants, including investigators and outcome assessors, were blinded to treatment allocation, with the only exception of the research nurses, not otherwise involved in the trial, who prepared the study drug (active compound or placebo) and administered it to the patients.

**Additional statistical analysis information**

One interim efficacy comparison was done by the Independent Data Safety Monitoring Board after the 49^th^ patient completed 1-year follow-up to assess whether the study was to continue as planned, or whether the sample size and/or follow-up duration needed to be revised or the study stopped for futility or safety reasons (for detailed stopping rules, see study protocol at <http://clintrials.marionegri.it/index.php/electronictrials/completed-electronic-trials.html>). This interim analysis was performed by the Independent Data Safety Monitoring Board (see Study Organization) on March 3, 2014, whose decision was to continue the trial as per protocol.

In order to provide analytic reassurance that our findings on mGFR and total kidney volume were robust to missing data, we first carried out a non-parametric simple mean imputation, replacing the missing values with the arithmetic average of the observed data for that variable. We then used a parametric, multiple imputation by chained equations (‘ice’ STATA 12 command) [1,2]. The process was iterated through ten cycles and the procedure repeated n=5 times to produce five imputed datasets. This approach provided parameter estimates under the Missing At Random (MAR) mechanism [1]. Although we cannot assess it for certainty, the above classification is the most plausible. The results of multiple imputations were obtained by the two datasets with the lowest and the highest mean of the five imputed datasets. Results of multiple imputation analyses confirmed that study findings are robust to missing data. Altogether, the above data suggest that it is very unlikely that missing data introduced appreciable attrition bias into the study findings.

**Adverse event definition for safety analysis**

The adverse events reported during the ALADIN 2 study were defined according to seriousness and intensity. In particular, a Serious Adverse Event (SAE) was any untoward medical occurrence that met any of the following criteria: results in death, is immediately life-threatening, requires in-patient hospitalization or prolongation of existing hospitalization, results in persistent or significant disability/incapacity. An event which did not meet these definitions was considered to be 'Not Serious'. The intensity of an adverse event was classified according to the following categories: Mild: usually transient in nature and generally not interfering with normal activities; Moderate: sufficiently discomforting to interfere with normal activities; Severe: prevents normal activities [3].

**Computed tomography processing and volume quantification**

Once acquired, computed tomography (CT) scans were transferred to a DICOM 16-bit format from the clinical scanner to digital media and sent to the coordinating center (Medical Imaging Unit, Bioengineering Department, Mario Negri Institute for Pharmacological Research, Bergamo, Italy) for subsequent central processing. Upon receipt, each CT acquisition underwent careful quality control, to ensure compliance with the acquisition protocol and image quality adequacy, and was re-sampled to 5 mm slice thickness for volume quantification. To quantify total kidney volume (TKV), kidneys were first manually outlined on all acquired digital images by trained operators (two bioengineers and one nephrologist), blind to treatment. Manual tracing was performed by the ImageJ interactive image editing software (ImageJ, Image processing and Analysis in Java, National Institutes of Health, http://rsbweb.nih.gov/ij) using a planimetry polyline method denoted by high intra- and inter-rater reliability (coefficient of variation of repeated measures = 1.14% and 1.38%, respectively) [4]. The main renal blood vessels and hilum were carefully excluded from the kidney outlines. Special attention was given to regions where the kidneys and liver were adjacent. Tracing accuracy was double-checked and, whenever needed, manual outlines were corrected by a single, expert, blind operator (A.C., with 10 years of experience in ADPKD imaging), in order to limit potential inter-operator variability. Renal masks were created from manual outlining, and TKV was computed by multiplying the voxel count of the masks by voxel volume. Volume computation was performed with in-house software based on the Insight Toolkit version 4.5 [5] and developed in the C++ programming language. TKV was finally adjusted for height (htTKV, mL/m) [6] to take into account differences between male and female patients.

**References**

1. Little RJA, Rubin DB. Statistical Analysis with Missing data. 2nd Edition. NY: Wiley Series in Probability & Statistics; 2002.

2. White IR, Royston P, Wood AM. Multiple imputation using chained equations: Issues and guidance for practice. Stat Med. 2011; 30: 377-99.

3. (EUROPEAN COMMISSION. Communication from the Commission — Detailed guidance on the collection, verification and presentation of adverse event/reaction reports arising from clinical trials on medicinal products for human use (‘CT-3’), 2011,C172:01-13) accessed on March 20, 2018 at https://ec.europa.eu/health/sites/health/files/files/eudralex/vol-10/2011_c172_01/2011_c172_01_en.pdf).

4. Sharma K, Caroli A, Quach LV, et al.. Kidney volume measurement methods for clincila studies on autosomal dominant polycystic kidney disease. PLoS One 2017; 12: e0178488.

5. Ibanez L, Schroeder W, Ng L, Cates J. The ITK Software Guide, 2nd Ed., Albany, Kitware Inc.; 2005.

6. Chapman AB, Bost JE, Torres VE, et al. Kidney volume and functional outcomes in autosomal dominant polycystic kidney disease. Clin J Am Soc Nephrol. 2012; 7: 479-86.
