# Supplementary material for: Octreotide-LAR in later-stage autosomal dominant polycystic kidney disease (ALADIN 2): A randomized, double-blind, placebo-controlled, multicenter trial
Source: PLoS Med. 2019 Apr 5;16(4):e1002777. doi: 10.1371/journal.pmed.1002777 (PMC6450618; doi:10.1371/journal.pmed.1002777)
Supplement: S2 Text — (PDF) [file pmed.1002777.s015.pdf]

A PROSPECTIVE, RANDOMIZED, DOUBLE-BLIND, PLACEBO CONTROLLED CLINICAL TRIAL TO ASSESS THE EFFECTS OF LONG-ACTING SOMATOSTATIN (OCTREOTIDE LAR) THERAPY ON DISEASE PROGRESSION IN PATIENTS WITH AUTOSOMAL DOMINANT POLYCYSTIC KIDNEY DISEASE AND MODERATE TO SEVERE RENAL INSUFFICIENCY (ALADIN 2 STUDY)

Sponsor: **IRCCS Mario Negri Institute for Pharmacological research**
Clinical Researh Center for Rare Diseases
Aldo e Cele Daccò **- Ranica, Bergamo (Italy)**

Co-sponsor: **Novartis Farma S.p.A.**
Origgio, Varese (Italy)

Principal Investigators: **Dr. Piero Ruggenenti**
Dr. Norberto Perico

STATISTICAL ANALYSIS PLAN (VERSION 1.0)

Study Number: **EUDRACT 2011-000138-12**

DATE **September 06, 2013**

AUTHOR **Annalisa Perna**

REVIEW

Giuseppe Remuzzi
Piero Ruggenenti

APPROVAL

__September 06, 2013__
(Date)

Piero Ruggenenti
Department of Renal Medicine
IRCCS Mario Negri Institute for Pharmacological Research,
Clinical Research Center for Rare Diseases,
Ranica (Bergamo), Italy

__September 06, 2013__
(Date)

Annalisa Perna
Department of Renal Medicine
Laboratory of Biostatistics
IRCCS Mario Negri Institute for Pharmacological Research,
Clinical Research Center for Rare Diseases,
Ranica (Bergamo), Italy

CONTENTS

1. INTRODUCTION 5
2. STUDY CONDUCT 8
  2.1 Analysis Populations 8
  2.2 Patient Accounting 9
  2.3 Protocol Violations 9
  2.4 Compliance and Adherence to Study Schedule 10
3. STATISTICAL METHODS 11
  3.1 Baseline Summaries 11
  3.2 Analysis Specifications 14
  3.3 Efficacy Analysis 14
    3.3.1.1 One Year Primary Efficacy Parameter 14
    3.3.1.2 Three Years Primary efficacy Parameter 15
    3.3.2 Secondary Efficacy Parameters 15
      3.3.2.1 Blood Pressure, Heart Rate and Weight 15
      3.3.2.2 Serum Creatinine 15
      3.3.2.3 Hematochemistry 15
      3.3.2.4 Creatinine Clearance 16
      3.3.2.5 24 Hours Urine Collection 16
      3.3.2.6 Spot Morning Urine 16
      3.3.2.7 TKV, absolute and percent TKV changes 16
      3.3.2.8 htTKV, absolute and percent htTKV changes 16
      3.3.2.9 Hemochrome 17
      3.3.2.10 Total slope eGFR (MDRD and CKD formula) 17
      3.3.2.11 Chronic slope mGFR and eGFR (MDRD and CKD formula) 17
    3.3.3 Other Efficacy Parameters 18
      3.3.3.1 Time to ESRD and to Composite Endpoint 18
      3.3.3.2 Overall,,Cardiovascular and Non-cardiovascular Mortality 19
      3.3.3.3 Incidence of Major cardiovascular Events 19
  3.4 Safety Analysis 20
    3.4.1 Adverse Events 21
    3.4.2 Deaths 21
    3.4.3 Laboratory Tests 21
    3.4.4 Urine Analysis 22

CONTENTS (continued)

4. PROPOSED TABLES 24

5. PROPOSED FIGURES 26

6. PROPOSED APPENDICES 27

7. TABLE TEMPLATES

SECTION 1

INTRODUCTION

This study is a multi-centre, prospective, randomised double-blind parallel-group comparison of long acting somatostatin analogue (Octreotide LAR) and placebo.
The study is coordinated by the Clinical Research Center for Rare Diseases of the Mario Negri Institute for Pharmacological Research, Ranica, Bergamo and will be conducted with the cooperation of:

- Hospital *'Ospedale San Giovanni di Dio'*, Agrigento, Italy;
- Clinical Research Center for Rare Diseases *'Aldo e Cele Daccò'*, Ranica (Bergamo), Italy
- Hospital *'Ospedale Vito Fazzi'*, Lecce, Italy
- Hospital *'Ospedale Maggiore Policlinico, Mangiagalli e Regina Elena'*, Milan, Italy
- University *'Università Federico II'*, Naples, Italy
- Hospital *'Ospedale Ca' Foncello'*, Treviso, Italy

Eligible patients will be stratified for the presence (YES or NO) of concomitant clinical conditions that could appreciably affect the rate of renal function loss over time including diabetes mellitus, 24 hour urinary protein excretion >1g, or any other evidence of concomitant renal disease without indication for specific therapy. Within each stratum patients will be randomized on a 1:1 basis to Long-acting Somatostatin (Octreotide LAR) 40 mg (treatment group) or saline solution (placebo group). Data will be collected by means of an *ad-hoc* study Case Report Form (see Appendix 1).
The general aim of the trial is to assess the efficacy of one year treatment with long-acting somatostatin analogue (Octreotide LAR) compared with placebo in slowing kidney and liver growth rate in patients with ADPKD and moderate/severe renal insufficiency (estimated GFR by MDRD 4 variables: 15-40 ml/min/1.73m2) and to assess whether and to which extent this translates into slower renal function decline over 3-year follow-up.
Primary objectives are the followings: to compare the effect of long acting somatostatin analogue (Octreotide LAR) versus placebo on total kidney volume (TKV) change (delta TKV) as assessed by spiral computed tomography (spiral CT)

scan (short-term, one year) and to compare the effect of long-acting somatostatin analogue (Octreotide LAR) vs. placebo on the rate of GFR decline as assessed by serial measurements of the iohexol plasma clearance (long-term, three years).

The secondary objectives are to compare, among the two groups, absolute and relative changes in renal and liver volume parameters (at month 0, 12 and 36) as well as in functional and biochemical parameters by quarterly clinical visits during the 36 month follow-up. In particular, the following parameters will be assessed:

- Total kidney volume (at 3-years)
- Total renal cyst volume*
- Total renal parenchyma volume*
- Total renal intermediate volume*
- Total liver and liver cyst volumes*

**When feasible, by minimized radio contrast agent administration during CT acquisition*

- GFR decline (as assessed by serial measurements of the plasma iohexol clearance at month 0 and, every 6 months thereafter)
- Slope of eGFR (by MDRD 4 variable equation)
- Progression to the combined end point of doubling of serum creatinine and/or ESRD (*ESRD: need for chronic renal replacement therapy by dialysis or transplantation established on the basis of standard clinical criteria)*
- Progression to ESRD considered as a single endpoint
- Systolic, diastolic and mean blood pressure
- 24 h urinary protein excretion rate (at month 0 and every six months thereafter)
- Urinary osmolality (assessed from urine sodium, urea and glucose concentration on 24 hr urine samples)
- Morning spot urine sample (proteins, albumin, creatinine)
- Serum Insulin Growth Factor-1 (IGF-I)
- 24 h urinary phosphorus, creatinine and creatinine clearance
- Gallbladder sand/stones and renal stones (by ultrasonography).

In a subgroup of patients referred to the Clinical Research Center for Rare Diseases 'Aldo e Cele Daccò' and/or other institutions where these evaluations are feasible the

following other secondary objectives are to compare, among the two groups the quality of life and the societal costs by submitting to consenting patients a *Quality of life (QoL) Questionnaire* - Version 1 of SF-36 (QoL SF-36 V1^{TM} Questionnaire) validated in Italy – and a *Short-Form-Health and Labour Questionnaire (SFHLQ)*. (See Appendix 2).

Furthermore, for exploratory purposes only, the treatment effect on endothelin and MCP-1 urinary excretion rate (overnight urine sample), in a subgroup (pending on study findings and fund availability).

This statistical analysis plan will be reviewed at the blind review of the ALADIN 2 data and modifications in light of the study conduct considered in line with the recommendations in the International Conference on Harmonization (ICH) guideline, 'Statistical Principles for Clinical Trials E9' (ICH (1998)). The above statistical plan is inspired to the ALADIN trial report (Caroli 2013), due to the analogies with ALADIN 2 study design.

SECTION 2

STUDY CONDUCT

2.1 ANALYSIS POPULATIONS

Several analysis populations will be used:

Full Analysis Set
This set will consist of all patients randomised to receive study medication in the ALADIN 2 study.

Per-Protocol Set
This set will include all patients randomised to receive study medication in the ALADIN 2 study. All patients who have major protocol violations (see Paragraph 2.3) will be excluded.

Safety Set
This set will consist of all patients who received at least one dose of study medication.

A decision regarding the exact composition of the Per-Protocol Set will be made at the 'blind review' stage.

The efficacy analysis will be performed on both the Full Analysis Set and the Per-Protocol Analysis Set. The analysis performed on the Full Analysis Set should be considered the primary one.

The safety analysis will be performed on the Safety Set.

2.2 PATIENT ACCOUNTING

Individual patient randomisation details will be presented in Appendix 21.

Patient accounting details will be presented in Table 1.1. This table will provide numbers of patients in each of the analysis populations and the number of patients per site.
An outline of the treatment phase is described in the flow chart of the study (Appendix 3).
Table 1.2 will show the numbers of patients remaining in the study, in each treatment group and overall, at each of the follow-up time points, for the Full Analysis Set and the Per Protocol Set.

The reasons for withdrawal will be summarised in Table 1.3, by treatment group and overall. In addition, an individual patient data listing of trial-end details will be provided in Appendix 4.

2.3 PROTOCOL VIOLATIONS

The following are defined to be major protocol violations.

- Age <18 years
- Absence of clinical diagnosis of ADPKD
- Absence of ultrasound diagnosis of ADPKD
- Written Informed consent not given
- Presence of symptomatic biliary tract lithiasis
- Presence of active cancer
- Presence of psychiatric disorders or any condition that might prevent full comprehension of the purposes and risks of the study
- Presence of pregnancy, lactation or child bearing potential and ineffective contraception

During follow-up:

- Temporary withdrawal for at least 30 consecutive days or total amount of temporary withdrawal days more or equal to 20% of total duration of treatment phase
- Study duration less than 3 months when the main reason for discontinuation is: unsatisfactory efficacy, unsatisfactory compliance, protocol violation, lost of follow-up, withdrawal of consent. Patients who discontinued due to adverse event or death are *not* considered as major protocol violators.
- Breaking the blind code after randomization.

All remaining violations are to be considered minor protocol violations.

An individual patient data listing of all detected protocol violations (major and minor) will be provided in Appendix 5, identifying major protocol violations which resulted in the exclusion of the patient from the Per Protocol Set. Tables 1.4.1 and 1.4.2 summarize how often different types of violations occurred.

2.4 COMPLIANCE AND ADHERENCE TO STUDY SCHEDULE

Compliance details, in terms of temporary withdrawal, will be listed in Appendix 11.

The differences between actual and scheduled timing of each of the visits will be summarised in Table 1.5. The following schedule in relation to the differencing of dates will be adopted:

One week = 7 days,	One month = 30 days,
3 months = 91 days,	6 months = 183 days,
9 months = 274 days,	12 months = 365 days,

15 months = 456 days,	18 months = 548 days,
21 months = 639 days,	24 months = 730 days,
27 months = 821 days,	30 months = 913 days,
36 months = 1095 days.	

The following acceptable time windows in relation to the differencing of dates will be considered:

One week = 4-10 days,	One month = 20-40 days,
3 months = 61-121 days,	6 months = 153-213 days,
9 months = 244-304 days,	12 months = 335-395 days,
15 months = 426-486 days,	18 months = 518-578 days,
21 months = 609-669 days,	24 months = 700-760 days,
27 months = 791-851 days,	30 months = 883-943 days,
36 months = 1065-1125 days.	

SECTION 3

STATISTICAL METHODS

3.1 BASELINE SUMMARIES

The baseline visit is Visit 2 (Time 0). Summary statistics will be tabulated by treatment group, to assess the comparability of the groups, and overall, to describe the patient population (Full Analysis Set and Per Protocol Set), for the following parameters:

- Age, Gender, Race, Weight, Smoke, Alcohol, Coffee/tea, Marital Status, Education, Professional Status overall (Table 2.1.1) and according to stratum: CCC absent (Table 2.1.2) and to stratum: CCC present (Table 2.1.3)

- Vital signs: Blood Pressure (systolic and diastolic), Pulse Rate, Weight, Mean Arterial Pressure (defined as 2/3 diastolic blood pressure plus 1/3 systolic blood pressure) and ECG results overall (Table 2.2.1) and according to stratum: CCC absent (Table 2.2.2) and to stratum: CCC present (Table 2.2.3)
- TKV, height-adjusted TKV, overall (Table2.3.1) and according to stratum: CCC absent (Table2.3.2) and to stratum: CCC present (Table2.3.3)
- mGlomerular Filtration Rate (m = measured) measured by iohexol plasma clearance (Table 2.4.1) overall and according to stratum: CCC absent (Table 2.4.2) and to stratum: CCC present (Table 2.4.3)
- eGlomerular Filtration Rate (e = estimated) using the 'four variable' abbreviated MDRD formula: $GFR=186x(SCR)^{-1.154}x\ (Age)^{-0.203}x(0.755$ if female)x(1.210 if African-American) (Table 2.5.1) overall and according to stratum: CCC absent (Table 2.5.2) and to stratum: CCC present (Table 2.5.3)
- eGlomerular Filtration Rate (e = estimated) using the CKD-EPI equation (Levey 2009): if female and serum creatinine ≤0.7 mg/dl: $144 \times (0.993)^{Age} \times (Scr/0.7)^{-0.329}$; if female and serum creatinine >0.7 mg/dl: $144 \times (0.993)^{Age} \times (Scr/0.7)^{-1.209}$; if male and serum creatinine ≤0.9 mg/dl: $141 \times (0.993)^{Age} \times (Scr/0.7)^{-0.411}$; if male and serum creatinine >0.9 mg dl: $141 \times (0.993)^{Age} \times (Scr/0.7)^{-1.209}$ (Table 2.6.1) overall and according to stratum: CCC absent (Table 2.6.2) and to stratum: CCC present (Table 2.6.3)
- Serum creatinine (Table 2.7.1) overall and according to stratum: CCC absent (Table 2.7.2) and to stratum: CCC present (Table 2.7.3)
- Hematochemistry: Urea, Sodium, Potassium, Calcium, Phosphorus, GOT, GPT, GGT, Alkaline phosphatase, total bilirubin, Direct Bilirubin, glucose, HbA1c, Uric Acid, Total Proteins, Albumin, IGF1 (Tables 2.8.1-2.24.1) overall and according to stratum: CCC absent (Tables 2.8.2-2.24.2) and to stratum: CCC present (Table 2.8.3-2.24.3)
- Lipid profile: Total cholesterol, HDL cholesterol, LDL cholesterol, Triglycerides (Tables 2.25.1-2.28.1) overall and according to stratum: CCC absent (Tables 2.25.2-2.28.2) and to stratum: CCC present (Table 2.25.3-2.28.3)

- Hemochrome: Leukocytes, Platelets, Erytrhocytes, Hemoglobin, Hematocrit (Tables 2.29.1-2.33.1) overall and according to stratum: CCC absent (Tables 2.29.2-2.33.2) and to stratum: CCC present (Table 2.29.3-2.33.3)
- 24 Hours Urine collection: Proteins, Creatinine, Creatinine Clearance, Sodium, Urea, Glucose, Phosphate, Osmolality (Tables 2.34.1-2.41.1) overall and according to stratum: CCC absent (Tables 2.34.2-2.41.2) and to stratum: CCC present (Table 2.34.3-2.41.3)
- Spot Morning Urine: Proteins, Albumin, Creatinine, Protein-to-creatinine ratio, Albumin-to-creatinine ratio (Tables 2.42.1-2.46.1) overall and according to stratum: CCC absent (Tables 2.42.2-2.46.2) and to stratum: CCC present (Table 2.42.3-2.46.3)
- Previous Diseases (Table 2.47.1) overall and according to stratum: CCC absent (Table 2.47.2) and to stratum: CCC present (Table 2.47.3)
- Previous Medication/Non-Drug Therapy (Table 2.48.1) overall and according to stratum: CCC absent (Table 2.48.2) and to stratum: CCC present (Table 2.48.3)
- Concomitant Diseases (Table 2.49.1) overall and according to stratum: CCC absent (Table 2.49.2) and to stratum: CCC present (Table 2.49.3)
- Concomitant Medication/Non-Drug Therapy (Table 2.50.1) overall and according to stratum: CCC absent (Table 2.50.2) and to stratum: CCC present (Table 2.50.3)

For 'continuous' variables (e.g. age) the statistics presented will be mean(sd), median, Q1, Q3, minimum, and maximum and number of patients. For categorical variables (e.g. gender) frequency counts and percentages will be provided. Diseases will be summarised by MedDRA preferred term and medication/non-drug therapy will be summarised by ATC class (level 2), in terms of frequency counts and percentages.
Individual patient data listings of demographic data, previous diseases, concomitant diseases, previous medication/non-drug therapy and concomitant medication/non-drug therapy will be presented in Appendices 6 to 10 respectively.

3.2 ANALYSIS SPECIFICATIONS

Assumptions underlying all parametric analyses will be checked and transformations to normality (e.g. log-transformation) or non-parametric methods used, where violations of assumptions are seen.

For efficacy analyses based on the Full Analysis Set, no imputation of missing values will be performed. The last available data value will be used to calculate the time to onset of an event.
For both the analyses based on the Full Analysis Set and the Per Protocol Set, visit windows will not be used and data points will be classified according to the named (nominal) visit.

All statistical tests will be two-sided unless otherwise stated.

3.3 EFFICACY ANALYSIS

Primary efficacy analysis at one year is referred to TKV absolute change from time 0 (Visit 2) to 12 months (Visit 7). Primary efficacy analysis at three years is referred to the measured GFR slope from time 0 (Visit 2) onward.

3.3.1.1 One Year Primary Efficacy Parameter

The short-term primary efficacy variable at one year, i.e. absolute change at 12 months from baseline in TKV, will be compared between the treatment groups using analysis of covariance (ANCOVA) model of the log-transformed TKV, including baseline TKV, age and gender as covariates. Details including least square means and standard errors, and covariate coefficients, standard errors, p-values and 95% confidence intervals will be provided respectively. (Senn (1997)). In case of departure from Normality assumptions in the log-transformed TKV distribution a non parametric approach will be used instead. Summary statistics, namely mean (sd), median, q1, q3, minimum, and

maximum for TKV and absolute TKV change from baseline to six months will be tabulated by nominal visit and treatment group (Tables 3.1.1-3.1.3).

3.3.1.2 Three Years Primary Efficacy Parameter

The long-term primary efficacy variable - i.e. measured GFR total slope – will be assessed with a linear regression analysis and compared with the Wilcoxon rank-sum test. Exploratory linear mixed-models using SAS PROC MIXED will be also also used measured GFR repeated measures, with age, gender and baseline measured GFR as covariates. Between-groups differences in median GFR slopes and their 95% CI were determined by means of Hodges-Lehmann Estimation using the SAS PROC NPAR1WAY. Summary statistics, namely mean (sd), median, q1, q3, minimum, and maximum for measured GFR will be tabulated by nominal visit and treatment group. Moreover, relevant summary statistics will be presented for measured GFR total slope (overall and according to stratum CCC absent or stratum CCC present).

3.3.2 Secondary Efficacy Parameters

3.3.2.1 Blood Pressure, Pulse Rate and Weight (Tables 3.3.1 to 3.7.3)

Summary statistics, namely mean (sd), median, q1, q3, minimum, and maximum for the mean systolic and mean diastolic blood pressure, mean arterial pressure, pulse rate and weight, will be tabulated by nominal visit and treatment group.

3.3.2.2 Serum Creatinine (Tables 3.8.1-3.8.3)

Summary statistics, namely mean (sd), median, q1, q3, minimum, and maximum for serum creatinine, will be tabulated by nominal visit and treatment group.

3.3.2.3 Hematochemistry (Tables 3.9.1 3.25.3)

Summary statistics, namely mean (sd), median, q1, q3, minimum, and maximum for, Urea, Sodium, Potassium, Calcium, phosphorus, GOT, GPT, GGT, Alkaline phosphatase,

total bilirubin, Direct Bilirubin, glucose, HbA1c, Uric Acid, Total Proteins, Albumin and IGF1 will be tabulated by nominal visit and treatment group.

3.3.2.4 Creatinine Clearance (Tables 3.26.1 to 3.26.3)

Summary statistics, namely mean (sd), median, q1, q3, minimum, and maximum for Proteins, Creatinine, Creatinine Clearance, Sodium, Urea, Glucose, Phosphate and Osmolality will be tabulated by nominal visit and treatment group.

3.3.2.5 24 Hours Urine Collection (Tables 3.27.1 to 3.33. 3)

Summary statistics, namely mean (sd), median, q1, q3, minimum, and maximum for Proteins, Creatinine, Sodium, Urea, Glucose, Phosphate and Osmolality will be tabulated by nominal visit and treatment group.

3.3.2.6 Spot Morning Urine (Tables 3.34.1 to 3.38.3)

Summary statistics, namely mean (sd), median, q1, q3, minimum, and maximum for Proteins, Albumin, Creatinine, Protein-to-creatinine ratio, and Albumin-to-creatinine ratio will be tabulated by nominal visit and treatment group.

3.3.2.7 TKV and absolute and percent changes in TKV (Tables 3.39.1 to 3.39.3)

Summary statistics, namely mean (sd), median, q1, q3, minimum, and maximum for TKV and TKV absolute and percent changes from baseline onward will be tabulated by nominal visit and treatment group.

3.3.2.8 Height-adjusted TKV, absolute and percent changes in height-adjusted TKV (Tables 3.40.1 to 3.40.3)

Summary statistics, namely mean (sd), median, q1, q3, minimum, and maximum for height-adjusted TKV and absolute

and percent changes in height-adjusted TKV rom baseline onward will be tabulated by nominal visit and treatment group.

3.3.2.9 Lipid profile (Tables 3.41.1 to 3.44.3)

Summary statistics, namely mean (sd), median, q1, q3, minimum, and maximum for Total cholesterol, HDL cholesterol, LDL cholesterol and Triglycerides will be tabulated by nominal visit and treatment group.

3.3.2.10 Hemochrome (Tables 3.45.1 to 3.49.3)

Summary statistics, namely mean (sd), median, q1, q3, minimum, and maximum for Leukocytes, Platelets, Erytrhocytes, Hemoglobin and Hematocrit will be tabulated by nominal visit and treatment group.

3.3.2.11 Total slope of $eGFR_{mdrd}$ and $eGFR_{ckd\text{-}epi}$ (Tables 3.50.1-3.51.3)

The total slope of $eGFR_{mdrd}$ and $eGFR_{ckd\text{-}epi}$ will be described by the slope calculated for each patient using linear regression. Relevant summary statistics and the results of the analyses will be presented in Tables 3.50.1-3.51.3.

3.3.2.1 Chronic Slope of mGFR, $eGFR_{mdrd}$ and $eGFR_{ckd\text{-}epi}$ (Tables 3.2, 3.52, 3.53)

The chronic slope of mGFR, $eGFR_{mdrd}$ and $eGFR_{ckd\text{-}epi}$ will be calculated from six months onward and described by the slope calculated for each patient using linear regression. Relevant summary statistics and the results of the analyses will be presented in **Tables 3.2, 3.52 and 3.53.**

3.3.3 Other Efficacy Parameters

3.3.3.1 Time to ESRD and time to doubling of Serum Creatinine or ESRD (Tables 3.53.1-3.53.2, 3.54.1-3.54.2, Figures 1 and 2)

The time to the ESRD and the time to the composite endpoint 'doubling of serum creatinine or ESRD will give rise to right censored data. Kaplan-Meier curves will be plotted in Figures 1 and 2 and summary statistics, including the Kaplan-Meier estimates of the proportions of patients event-free at monthly intervals, median event times and proportions of patients providing censored values, will be given in Table 3.53.1 and 3.54.1 respectively. Confidence intervals from these proportions and for the median event time calculated using Greenwood's formula will also be provided in Table 3.53.1 and 3.54.1. Cumulative event curves will be constructed using the Kaplan-Meier procedure and the Cox regression model will be used to estimate the hazard ratio for the ESRD endpoint and 'doubling of serum creatinine or ESRD' composite endpoint with the corresponding 95% CI. The model will be adjusted for age, gender and baseline serum creatinine. Participants who didn't reach the outcome of interest will be considered as right censored. Proportionality assumptions were assessed using Schoenfeld residuals. Individual patient data for the time to ESRD and time to doubling of serum creatinine or ESRD will be listed in Appendix 12 and 13

3.3.3.2 Overall, Cardiovascular and Non-cardiovascular Mortality (Tables 3.55.1 to 3.57.2 and Figures 3,4 and 5)

Time to total death, time to cardiovascular death and time to non-cardiovascular death will be analysed as time to ESRD, outlined in Section 3.3.3.1. Kaplan-Meier curves will be plotted in Figures 3,4 and 5 while Tables 3.55.1 to 3.57.2 will contain, respectively, summary statistics associated with the

Kaplan-Meier curves, and Proportional Hazards regression model fitting details. Individual patient data for the time to death, time to cardiovascular mortality and time to non-cardiovascular mortality will be listed in Appendices 14-16.

3.3.3.3 Incidence of Major Cardiovascular Events (Tables 3.58.1-3.58.2 and Figure 6)

Classification of major cardiovascular events will take place at the 'blind review' stage. A "major" CV event is, by definition, a fatal or nonfatal Serious Adverse Event (SAE). Consequently, the final list of all SAEs identified for SAE reconciliation will be considered also for classifying each event as "major CV" or not. The CV events proposed as "major" will be "peripheral revascularization or amputation", including: first event of Myocardial Infarction (MI), stroke, Transitory Ischemic Attack (TIA), first hospitalisation for heart failure, unstable angina, coronary or peripheral revascularization, mesenteric infarction or amputation. "First event" is specified in order to avoid the situation of counting the patient twice for the same event. A composite endpoint of the above mentioned "major" CV events will be considered. The single components (events) will also be evaluated. Carotid stenoses or any other vascular disease that does not result in any of the above events will not be considered as major CV events, but just as vascular changes. Frequency distributions for the number of cardiovascular events per patient will be tabulated by treatment group in Table 3.58.1. The time to the first major cardiovascular event will be analysed as time to ESRD, outlined in Section 3.3.3.1. Kaplan-Meier curves will be plotted in Figure 6, while Table 3.58.2 will contain summary statistics associated with the Kaplan-Meier curves, and Proportional Hazards regression model fitting details. Individual patient data for the time to first major cardiovascular event will be listed in Appendix 17.

3.4 SAFETY ANALYSIS

All safety analyses will be performed using the Safety Set

3.4.1 Adverse Events (Tables 4.1 to 4.5)

Adverse events with an onset after the 1st drug intake will be coded using the MedDRA classification system. Multiple reports of the same event by the same patient will be counted only once for the purposes of summary. This 'composite' event will have the 'worst' aspects of the observed multiple reports attributed to it. In particular, the worst maximum intensity, the most likely relationship to study medication and the most serious of the observed multiple reports will be attributed to the composite report. Also, if any of the multiple reports led to study drug discontinuation, this will be attributed to the composite report. This same approach will be used within a defined time period of the study, when summary of adverse events within sub-periods is undertaken. Missing values for dates will be imputed in the following way: '1' for day missing, 'January' for month missing. If however the year is missing this date will be considered as missing.

All adverse event summaries will be presented by treatment group and overall.

An overall summary of adverse events will be provided in Tables 4.1-4.5. This table will provide information on the numbers and percentages of patients with adverse events, serious adverse events, non-serious adverse events, severe adverse events, adverse events considered by the investigator to be probably related to the study drug and adverse events leading to permanent discontinuation. In addition, this table will show the average number of adverse events per patient, the average number of adverse events per patient experiencing adverse events and the number of deaths. A similar summary will be provided in Table 4.4 for serious adverse events only.

Adverse events (MedDRA preferred term) will be summarised by body system in Table 4.3. Similar summaries will be provided in Tables 4.4 to 4.5 for serious adverse events and deaths. Within the text of the statistical report, a summary of serious adverse events will be presented by treatment group, classifying whether each serious adverse event was:

- life-threatening
- permanently disabling
- required / prolonged hospitalisation
- cancer, congenital anomaly, result of overdose

Individual patient adverse event data will be listed in Appendix 18.

3.4.2 Death (Table 4.5)

The number of patients dying during the study will be presented by treatment group in Table 4.5. The reasons for death will also be summarised in terms of frequency counts and percentages.

3.4.3 Laboratory Tests (Tables 4.6 to 4.23)

Table 4.6 will contain summary statistics by treatment group for the complete collection of laboratory variables collected at Week 0. The statistics used will be mean(sd), median, minimum, and maximum and frequencies of normal or abnormal but not clinically relevant values (N) and abnormal and clinically relevant values (P).

Summary statistics will be provided for the 'selected' laboratory tests and for the change from baseline in the 'selected' laboratory tests at

each visit and also for the final visit (last value available) in Tables 4.6 to 4.23.

Individual patient laboratory data will be presented in Appendix 19.

3.4.4 Urine Analysis (Tables 4.24 to 4.30)

The first set of urine analysis tests at each visit will be used in the summaries.

Shift tables showing movement in terms of NN, NP, PN, PP (N=negative, P=positive) will be provided in Tables 4.24 to 4.30 for the urine analysis tests (leukocytes, nitrates, ketones, pH, glucose, Hb, proteins). pH will be summarised using the same categories as the 'selected' laboratory tests.

Individual patient urine analysis data will be listed in Appendix 20.

REFERENCES

ICH 1998

The ICH Guideline E9 Statistical Principles for Clinical Trials; European Medicines Agency 1998..

Caroli 2013

Caroli A, Perico N, Perna A, Antiga L, Brambilla P, Pisani A, et al. Effect of longacting somatostatin analogue on kidney and cyst growth in autosomal dominant polycystic kidney disease (ALADIN): a randomised, placebo-controlled, multicentre trial. *Lancet* 2013; 382: 1485-95.

Levey 2009

Levey AS et al. A new equation to estimate glomerular filtration rate. *Ann Intern Med* 2009; 150: 604-612

Senn 1997

Senn S. Statistical issues in drug development. Chichester: Wiley; 1997

SECTION 4

PROPOSED TABLES

1.1 Analysis Populations
1.2 Number of Randomised Patients at each Visit
1.3 Reasons for Withdrawal
1.4.1 Major protocol violations
1.4.2 Minor protocol violations
1.5 Adherence to Study Schedule
2.1.1 Demography (overall)
2.1.2 Demography (Stratum CCC Absent)
2.1.3 Demography (Stratum CCC Present)
2.2.1 Vital signs (Blood Pressure and ECG (overall)
2.2.2 Vital signs (Blood Pressure and ECG (Stratum CCC absent)
2.2.3 Vital signs (Blood Pressure and ECG (Stratum CCC Present)
2.3.1 TKV and height-adjusted TKV at Visit 2 (Month 0) – Overall
2.3.2 TKV and height-adjusted TKV at Visit 2 (Month 0) – Stratum CCC Absent
2.3.3 TKV and height-adjusted TKV at Visit 2 (Month 0) – Stratum CCC Present
2.4.1 Measured Glomerular Filtration Rate at Visit 2 (Month 0) – Overall
2.4.2 Measured Glomerular Filtration Rate at Visit 2 (Month 0) – Stratum CCC Absent
2.4.3 Measured Glomerular Filtration Rate at Visit 2 (Month 0) – Stratum CCC Present
2.5.1 Estimated (MDRD) GFR at Visit 2 (Month 0) – Overall
2.5.2 Estimated (MDRD) GFR at Visit 2 (Month 0) – Stratum CCC Absent
2.5.3 Estimated (MDRD) GFR at Visit 2 (Month 0) – Stratum CCC Present
2.6.1 Estimated (CKD-EPI) GFR at Visit 2 (Month 0) – Overall
2.6.2 Estimated (CKD-EPI) GFR at Visit 2 (Month 0) – Stratum CCC Absent
2.6.3 Estimated (CKD_EPI) GFR at Visit 2 (Month 0) – Stratum CCC Present
2.7.1 Serum Creatinine at Visit 2 (Month 0) – Overall
2.7.2 Serum Creatinine at Visit 2 (Month 0) – Stratum CCC Absent
2.7.3 Serum Creatinine at Visit 2 (Month 0) – Stratum CCC Present
2.8.1 Hematochemistry at Visit 2 (Month 0)(up to Table 2.24.3)

2.25.1 Lipid Profile at Visit 2 (Month 0) (up to Table 2.28.3)

2.29.1 Hemochrome at Visit 2 (Month 0) (up to Table 2.33.3)

2.34.1 24 Hours Urine Collection at Visit 2 (Month 0) (up to Table 2.41.3)

2.42.1 Spot Morning Urine at Visit 2 (Month 0) (up to Table 2.46.3)

2.47.1 Previous Diseases (up to Table 2.47.3)

2.48.1 Previous Medication/Non-Drug Therapy (up to Table 2.48.3)

2.49.1 Concomitant Diseases (up to Table 2.49.3)

2.50.1 Concomitant Medication/Non-Drug Therapy(up to Table 2.50.3)

3.1.1.1 One Year Primary Efficacy Parameter: TKV

3.1.1.2 Three Year Primary Efficacy Parameter: TKV

3.3.1 Blood Pressure, Pulse Rate and Weight (up to Table 3.7.3)

3.8.3 Serum Creatinine ((up to Table 3.8.3)

3.9.1 Hematochemistry (up to Table 3.25.3)

3.26.1 Creatinine Clearance (up to Table 3.26.3)

3.27.1 24 Hours Urine Collection (up to Table 3.33.3)

3.34.1 Spot Morning Urine (up to Table 3.38. 3)

3.39.1 TKV and absolute and percent changes in TKV (up to Table 3.39.3)

3.40.1 htTKV and absolute and percent changes in htTKV (up to Table 3.40.3)

3.41.1 Lipid Profile (up to Table 3.44.3)

3.45.1 Hemochrome (up to Table 3.49.3)

3.50.1 Total slope of $eGFR_{mdrd}$ and $Egfr_{ckd-epi}$ (up to Table 3.51.3)

3.52 Chronic slope of $eGFR_{mdrd}$ and $Egfr_{ckd-epi}$ (up to Table 3.53)

3.53.1 Time to ESRD and time to composite endpoint (up to Table 3.54.3)

3.55.1 Overall, Cardiovascular and Non-cardiovascular Mortality (up to Table 3.57.3)

3.58.1 Incidence of Major Cardiovascular Event (up to Table 3.58.2)

4.1 Summary of Adverse Events

4.2 Summary of Serious Adverse Events

4.3 Frequency of Adverse Events by Body System and Preferred Term

4.4 Frequency of Serious Adverse Events by Body System and Preferred Term

4.5 Death

4.6 Laboratory Tests at Baseline (Visit 2 - Month 0)

4.7 Laboratory Test Shift Table – Creatinine (mg/dl)

4.8 Laboratory Test Shift Table – Urea (mg/dl)

4.9 Laboratory Test Shift Table – Calcium (mg/dl)

4.10 Laboratory Test Shift Table – Sodium (mEq/l)

4.11 Laboratory Test Shift Table – Potassium (mEq/l)

4.12 Laboratory Test Shift Table – Phosphorous (mg/dl)

4.13 Summary Statistics for Change from Baseline – Creatinine (mg/dl)

4.14 Summary Statistics for Change from Baseline – Urea (mg/dl)

4.15 Summary Statistics for Change from Baseline – Calcium (mg/dl)

4.16 Summary Statistics for Change from Baseline – Sodium (mEq/l)

4.17 Summary Statistics for Change from Baseline – Potassium (mEq/l)

4.18 Summary Statistics for Change from Baseline – Phosphorous (mg/dl)

4.19 Urine Analysis Shift Table – Leukocytes

4.20 Urine Analysis Shift Table – Nitrates

4.21 Urine Analysis Shift Table – Ketones

4.22 Urine Analysis Shift Table – pH

4.23 Urine Analysis Shift Table – Glucose

4.24 Change From Baseline serum creatinine

4.25 Urine Analysis Shift Table – Hb

4.26 Urine Analysis Shift Table – Proteins

4.27 ECG Shift Table

SECTION 5

PROPOSED FIGURES

1. Kaplan-Meier Curves for ESRD
2. Kaplan-Meier Curves for Doubling of Serum Creatinine or ESRD
3. Kaplan-Meier Curves for Time to Overall Mortality
4. Kaplan-Meier Curves for Time to Cardiovascular Mortality
5. Kaplan-Meier Curves for Time to Non-cardiovascular Mortality
6. Kaplan-Meier Curves for Time to First Major Cardiovascular Event

These graphs will be produced for both the Full Analysis Set and Per Protocol Set and according to Stratum CCC absent and to Stratum CCC present.

SECTION 6

PROPOSED APPENDICES

1. Case Report Form
2. Quality of Life and Health and labour Questionnaire
3. Study Flow Chart
4. Trial-End Summary
5. Protocol Violations
6. Demographic Data
7. Previous Disease
8. Concomitant Disease
9. Previous Medication/Non-drug Therapy
10. Concomitant Medication/Non-drug Therapy
11. Temporary Withdrawal
12. Time to ESRD
13. Time to doubling of serum creatinine or ESRD
14. Time to overall mortality
15. Time to cardiovascular mortality
16. Time to non-cardiovascular mortality
17. Time to First Major cardiovascular event
18. Adverse events
19. Laboratory data
20. Urine analysis
21. Randomization details

ISTITUTO DI RICERCHE FARMACOLOGICHE MARIO NEGRI

Centro di Ricerche Cliniche per le Malattie Rare "Aldo e Cele Daccò"

Villa Camozzi – 24020 Ranica (BG) – Italy, tel +39.035.45351 - fax +39.035.4535.371
villacamozzi@marionegri.it – http://villacamozzi.marionegri.it

A prospective, randomized, double-blind, placebo controlled clinical trial to assess the effects of long-acting somatostatin (octreotide Lar) therapy on disease progression in patients with autosomal dominant polycystic kidney disease and moderate to severe renal insufficiency (ALADIN 2 Study)

Electronic Case Report Form

September 16th, 2011

I CONTRIBUTI PER LA RICERCA VERSATI ALL'ISTITUTO SONO FISCALMENTE DEDUCIBILI DAL REDDITO (Gazzetta Uff. N.179 del 3/8/2011)
FONDAZIONE PER RICERCHE ERETTA IN ENTE MORALE, D.P.R. 361 DEL 5/4/1961 - REGISTRO PERSONE GIURIDICHE PREFETTURA MILANO N.227
CONTO CORRENTE POST. N.58337205 - COD. FISC. E PARTITA IVA 03254210150 - ANAGRAFE NAZIONALE RICERCHE COD.G1690099
RECOGNIZED AS A TAX EXEMPT ORGANIZATION UNDER SECTION 501 (c)(3) OF THE USA INTERNAL REVENUE CODE-TAX I.D. No.: 98-6000957
Sistema di gestione qualità certificato da Certiquality UNI EN ISO 9001:2008,
progettazione ed erogazione di corsi di formazione specialistica nell'ambito della biologia e della medicina
SEDE LEGALE: Via Giuseppe La Masa, 19 - 20156 Milano MI - Italy

BASELINE DATA FORM DATA SUMMARY	
USER: Nadia Rubis	CENTER: -

PATIENT NUMBER: 666-01	VISIT: v01	ROW:	VISIT DATE: No date

FIELD	VALUE
Written Informed Consent	
Date of Informed Consent	
Birth Date	
Gender	
Height(cm)	
Race	
Other Race	
Smoke	
Cigarettes/Day	
Current Smoker Since	
Former Smoker Since	
Former Smoker To	
Alcohol	
Coffee/Tea	
Marital Status	
Education	
Professional Status	
Childbearing Potential	
Diabetes Mellitus	
Arterial Hypertension	
Coronary Heart Disease	
Polycystic Kidney Disease	
Other Renal Disease	
Cerebrovascular Disease	
Other Relevant Diseases	
Other Relevant Diseases Specify	

INSERTED ON: 2011-10-10 14:45:26	BY: Demo Demo	LOCKED: NO
PRINTED ON: 2011-10-10 15:36:42	BY: Nadia Rubis	

PREVIOUS DISEASES FORM DATA SUMMARY	
USER: Nadia Rubis	CENTER: -

PATIENT NUMBER: 666-01	VISIT: v01	ROW: 1	VISIT DATE: No date

FIELD	VALUE
Disease	
Onset Date	
Onset Date Unknown	No
Resolved Date	
Resolved Date Unknown	No
Resolved Date Ongoing	No

INSERTED ON: 2011-10-10 14:45:33	BY: Demo Demo	LOCKED: NO
PRINTED ON: 2011-10-10 15:36:42	BY: Nadia Rubis	

PATIENT NUMBER: 666-01	VISIT: v01	ROW: 2	VISIT DATE: No date

FIELD	VALUE
Disease	
Onset Date	
Onset Date Unknown	No
Resolved Date	
Resolved Date Unknown	No
Resolved Date Ongoing	No

INSERTED ON: 2011-10-10 14:45:38	BY: Demo Demo	LOCKED: NO
PRINTED ON: 2011-10-10 15:36:42	BY: Nadia Rubis	

PATIENT NUMBER: 666-01	VISIT: v01	ROW: 3	VISIT DATE: No date

FIELD	VALUE
Disease	
Onset Date	
Onset Date Unknown	No
Resolved Date	
Resolved Date Unknown	No
Resolved Date Ongoing	No

INSERTED ON: 2011-10-10 14:45:43	BY: Demo Demo	LOCKED: NO
PRINTED ON: 2011-10-10 15:36:42	BY: Nadia Rubis	

PATIENT NUMBER: 666-01	VISIT: v01	ROW: 4	VISIT DATE: No date

FIELD	VALUE
Disease	
Onset Date	
Onset Date Unknown	No
Resolved Date	
Resolved Date Unknown	No
Resolved Date Ongoing	No

INSERTED ON: 2011-10-10 14:45:47	BY: Demo Demo	LOCKED: NO
PRINTED ON: 2011-10-10 15:36:42	BY: Nadia Rubis	

PATIENT NUMBER: 666-01	VISIT: v01	ROW: 5	VISIT DATE: No date

FIELD	VALUE
Disease	
Onset Date	
Onset Date Unknown	No
Resolved Date	
Resolved Date Unknown	No
Resolved Date Ongoing	No

INSERTED ON: 2011-10-10 14:45:51	BY: Demo Demo	LOCKED: NO
PRINTED ON: 2011-10-10 15:36:42	BY: Nadia Rubis	

PREVIOUS TREATMENTS FORM DATA SUMMARY	
USER: Nadia Rubis	CENTER: -

PATIENT NUMBER: 666-01	VISIT: v01	ROW: 1	VISIT DATE: No date

FIELD	VALUE
Treatment	
First Intake Date	
First Intake Date Unknown	No
Last Intake Date	
Last Intake Date Unknown	No
Last Intake Date Ongoing	No
Dose	
Schedule	
Route	
Disease	

INSERTED ON: 2011-10-10 14:46:01	BY: Demo Demo	LOCKED: NO
PRINTED ON: 2011-10-10 15:36:42	BY: Nadia Rubis	

PATIENT NUMBER: 666-01	VISIT: v01	ROW: 2	VISIT DATE: No date

FIELD	VALUE
Treatment	
First Intake Date	
First Intake Date Unknown	No
Last Intake Date	
Last Intake Date Unknown	No
Last Intake Date Ongoing	No
Dose	
Schedule	
Route	
Disease	

INSERTED ON: 2011-10-10 14:46:06	BY: Demo Demo	LOCKED: NO
PRINTED ON: 2011-10-10 15:36:42	BY: Nadia Rubis	

PATIENT NUMBER: 666-01	VISIT: v01	ROW: 3	VISIT DATE: No date

FIELD	VALUE
Treatment	
First Intake Date	
First Intake Date Unknown	No
Last Intake Date	
Last Intake Date Unknown	No
Last Intake Date Ongoing	No
Dose	
Schedule	
Route	
Disease	

INSERTED ON: 2011-10-10 14:46:10	BY: Demo Demo	LOCKED: NO
PRINTED ON: 2011-10-10 15:36:42	BY: Nadia Rubis	

PATIENT NUMBER: 666-01	VISIT: v01	ROW: 4	VISIT DATE: No date

FIELD	VALUE
Treatment	
First Intake Date	
First Intake Date Unknown	No
Last Intake Date	
Last Intake Date Unknown	No
Last Intake Date Ongoing	No
Dose	
Schedule	
Route	
Disease	

INSERTED ON: 2011-10-10 14:46:15	BY: Demo Demo	LOCKED: NO
PRINTED ON: 2011-10-10 15:36:42	BY: Nadia Rubis	

PATIENT NUMBER: 666-01	VISIT: v01	ROW: 5	VISIT DATE: No date

FIELD	VALUE
Treatment	
First Intake Date	
First Intake Date Unknown	No
Last Intake Date	
Last Intake Date Unknown	No
Last Intake Date Ongoing	No
Dose	
Schedule	
Route	
Disease	

INSERTED ON: 2011-10-10 14:46:20	BY: Demo Demo	LOCKED: NO
PRINTED ON: 2011-10-10 15:36:42	BY: Nadia Rubis	

PHYSICAL EXAMINATION FORM DATA SUMMARY	
USER: Nadia Rubis	CENTER: -

PATIENT NUMBER: 666-01	VISIT: v02	ROW:	VISIT DATE: No date

FIELD	VALUE
Visit Date	
Date of Physical Examination	
Were there any pathological results observed within the physical examination?	
Date of Measurement	
Systolic(mmHg)-Value 1	
Diastolic(mmHg)-Value 1	
Pulse Rate(bpm)-Value 1	
Systolic(mmHg)-Value 2	
Diastolic(mmHg)-Value 2	
Pulse Rate(bpm)-Value 2	
Systolic(mmHg)-Value 3	
Diastolic(mmHg)-Value 3	
Pulse Rate(bpm)-Value 3	
Systolic Mean (mmHg)	0
Diastolic Mean (mmHg)	0
Pulse Rate Mean (bpm)	0
Weight (kg)	

INSERTED ON: 2011-10-10 14:46:27	BY: Demo Demo	LOCKED: NO
PRINTED ON: 2011-10-10 15:36:42	BY: Nadia Rubis	

PATIENT NUMBER: 666-01	VISIT: v03	ROW:	VISIT DATE: No date

FIELD	VALUE
Visit Date	
Date of Physical Examination	
Were there any pathological results observed within the physical examination?	
Date of Measurement	
Systolic(mmHg)-Value 1	
Diastolic(mmHg)-Value 1	
Pulse Rate(bpm)-Value 1	
Systolic(mmHg)-Value 2	
Diastolic(mmHg)-Value 2	
Pulse Rate(bpm)-Value 2	
Systolic(mmHg)-Value 3	
Diastolic(mmHg)-Value 3	
Pulse Rate(bpm)-Value 3	
Systolic Mean (mmHg)	0
Diastolic Mean (mmHg)	0
Pulse Rate Mean (bpm)	0
Weight (kg)	

INSERTED ON: 2011-10-10 14:46:32	BY: Demo Demo	LOCKED: NO
PRINTED ON: 2011-10-10 15:36:42	BY: Nadia Rubis	

PATIENT NUMBER: 666-01	VISIT: v04	ROW:	VISIT DATE: No date

FIELD	VALUE
Visit Date	
Date of Physical Examination	
Were there any pathological results observed within the physical examination?	
Date of Measurement	
Systolic(mmHg)-Value 1	
Diastolic(mmHg)-Value 1	
Pulse Rate(bpm)-Value 1	
Systolic(mmHg)-Value 2	
Diastolic(mmHg)-Value 2	
Pulse Rate(bpm)-Value 2	
Systolic(mmHg)-Value 3	
Diastolic(mmHg)-Value 3	
Pulse Rate(bpm)-Value 3	
Systolic Mean (mmHg)	0
Diastolic Mean (mmHg)	0
Pulse Rate Mean (bpm)	0
Weight (kg)	

INSERTED ON: 2011-10-10 14:46:36	BY: Demo Demo	LOCKED: NO
PRINTED ON: 2011-10-10 15:36:42	BY: Nadia Rubis	

PATIENT NUMBER: 666-01	VISIT: v05	ROW:	VISIT DATE: No date

FIELD	VALUE
Visit Date	
Date of Physical Examination	
Were there any pathological results observed within the physical examination?	
Date of Measurement	
Systolic(mmHg)-Value 1	
Diastolic(mmHg)-Value 1	
Pulse Rate(bpm)-Value 1	
Systolic(mmHg)-Value 2	
Diastolic(mmHg)-Value 2	
Pulse Rate(bpm)-Value 2	
Systolic(mmHg)-Value 3	
Diastolic(mmHg)-Value 3	
Pulse Rate(bpm)-Value 3	
Systolic Mean (mmHg)	0
Diastolic Mean (mmHg)	0
Pulse Rate Mean (bpm)	0
Weight (kg)	

INSERTED ON: 2011-10-10 14:46:40	BY: Demo Demo	LOCKED: NO
PRINTED ON: 2011-10-10 15:36:42	BY: Nadia Rubis	

PATIENT NUMBER: 666-01	VISIT: v06	ROW:	VISIT DATE: No date

FIELD	VALUE
Visit Date	
Date of Physical Examination	
Were there any pathological results observed within the physical examination?	
Date of Measurement	
Systolic(mmHg)-Value 1	
Diastolic(mmHg)-Value 1	
Pulse Rate(bpm)-Value 1	
Systolic(mmHg)-Value 2	
Diastolic(mmHg)-Value 2	
Pulse Rate(bpm)-Value 2	
Systolic(mmHg)-Value 3	
Diastolic(mmHg)-Value 3	
Pulse Rate(bpm)-Value 3	
Systolic Mean (mmHg)	0
Diastolic Mean (mmHg)	0
Pulse Rate Mean (bpm)	0
Weight (kg)	

INSERTED ON: 2011-10-10 14:46:44	BY: Demo Demo	LOCKED: NO
PRINTED ON: 2011-10-10 15:36:42	BY: Nadia Rubis	

PATIENT NUMBER: 666-01	VISIT: v07	ROW:	VISIT DATE: No date

FIELD	VALUE
Visit Date	
Date of Physical Examination	
Were there any pathological results observed within the physical examination?	
Date of Measurement	
Systolic(mmHg)-Value 1	
Diastolic(mmHg)-Value 1	
Pulse Rate(bpm)-Value 1	
Systolic(mmHg)-Value 2	
Diastolic(mmHg)-Value 2	
Pulse Rate(bpm)-Value 2	
Systolic(mmHg)-Value 3	
Diastolic(mmHg)-Value 3	
Pulse Rate(bpm)-Value 3	
Systolic Mean (mmHg)	0
Diastolic Mean (mmHg)	0
Pulse Rate Mean (bpm)	0
Weight (kg)	

INSERTED ON: 2011-10-10 14:46:48	BY: Demo Demo	LOCKED: NO
PRINTED ON: 2011-10-10 15:36:42	BY: Nadia Rubis	

PATIENT NUMBER: 666-01	VISIT: v08	ROW:	VISIT DATE: No date

FIELD	VALUE
Visit Date	
Date of Physical Examination	
Were there any pathological results observed within the physical examination?	
Date of Measurement	
Systolic(mmHg)-Value 1	
Diastolic(mmHg)-Value 1	
Pulse Rate(bpm)-Value 1	
Systolic(mmHg)-Value 2	
Diastolic(mmHg)-Value 2	
Pulse Rate(bpm)-Value 2	
Systolic(mmHg)-Value 3	
Diastolic(mmHg)-Value 3	
Pulse Rate(bpm)-Value 3	
Systolic Mean (mmHg)	0
Diastolic Mean (mmHg)	0
Pulse Rate Mean (bpm)	0
Weight (kg)	

INSERTED ON: 2011-10-10 14:46:53	BY: Demo Demo	LOCKED: NO
PRINTED ON: 2011-10-10 15:36:42	BY: Nadia Rubis	

PATIENT NUMBER: 666-01	VISIT: v09	ROW:	VISIT DATE: No date

FIELD	VALUE
Visit Date	
Date of Physical Examination	
Were there any pathological results observed within the physical examination?	
Date of Measurement	
Systolic(mmHg)-Value 1	
Diastolic(mmHg)-Value 1	
Pulse Rate(bpm)-Value 1	
Systolic(mmHg)-Value 2	
Diastolic(mmHg)-Value 2	
Pulse Rate(bpm)-Value 2	
Systolic(mmHg)-Value 3	
Diastolic(mmHg)-Value 3	
Pulse Rate(bpm)-Value 3	
Systolic Mean (mmHg)	0
Diastolic Mean (mmHg)	0
Pulse Rate Mean (bpm)	0
Weight (kg)	

INSERTED ON: 2011-10-10 14:46:57	BY: Demo Demo	LOCKED: NO
PRINTED ON: 2011-10-10 15:36:42	BY: Nadia Rubis	

PATIENT NUMBER: 666-01	VISIT: v10	ROW:	VISIT DATE: No date

FIELD	VALUE
Visit Date	
Date of Physical Examination	
Were there any pathological results observed within the physical examination?	
Date of Measurement	
Systolic(mmHg)-Value 1	
Diastolic(mmHg)-Value 1	
Pulse Rate(bpm)-Value 1	
Systolic(mmHg)-Value 2	
Diastolic(mmHg)-Value 2	
Pulse Rate(bpm)-Value 2	
Systolic(mmHg)-Value 3	
Diastolic(mmHg)-Value 3	
Pulse Rate(bpm)-Value 3	
Systolic Mean (mmHg)	0
Diastolic Mean (mmHg)	0
Pulse Rate Mean (bpm)	0
Weight (kg)	

INSERTED ON: 2011-10-10 14:47:02	BY: Demo Demo	LOCKED: NO
PRINTED ON: 2011-10-10 15:36:42	BY: Nadia Rubis	

PATIENT NUMBER: 666-01	VISIT: v11	ROW:	VISIT DATE: No date

FIELD	VALUE
Visit Date	
Date of Physical Examination	
Were there any pathological results observed within the physical examination?	
Date of Measurement	
Systolic(mmHg)-Value 1	
Diastolic(mmHg)-Value 1	
Pulse Rate(bpm)-Value 1	
Systolic(mmHg)-Value 2	
Diastolic(mmHg)-Value 2	
Pulse Rate(bpm)-Value 2	
Systolic(mmHg)-Value 3	
Diastolic(mmHg)-Value 3	
Pulse Rate(bpm)-Value 3	
Systolic Mean (mmHg)	0
Diastolic Mean (mmHg)	0
Pulse Rate Mean (bpm)	0
Weight (kg)	

INSERTED ON: 2011-10-10 14:47:06	BY: Demo Demo	LOCKED: NO
PRINTED ON: 2011-10-10 15:36:42	BY: Nadia Rubis	

PATIENT NUMBER: 666-01	VISIT: v12	ROW:	VISIT DATE: No date

FIELD	VALUE
Visit Date	
Date of Physical Examination	
Were there any pathological results observed within the physical examination?	
Date of Measurement	
Systolic(mmHg)-Value 1	
Diastolic(mmHg)-Value 1	
Pulse Rate(bpm)-Value 1	
Systolic(mmHg)-Value 2	
Diastolic(mmHg)-Value 2	
Pulse Rate(bpm)-Value 2	
Systolic(mmHg)-Value 3	
Diastolic(mmHg)-Value 3	
Pulse Rate(bpm)-Value 3	
Systolic Mean (mmHg)	0
Diastolic Mean (mmHg)	0
Pulse Rate Mean (bpm)	0
Weight (kg)	

INSERTED ON: 2011-10-10 14:47:10	BY: Demo Demo	LOCKED: NO
PRINTED ON: 2011-10-10 15:36:42	BY: Nadia Rubis	

PATIENT NUMBER: 666-01	VISIT: v13	ROW:	VISIT DATE: No date

FIELD	VALUE
Visit Date	
Date of Physical Examination	
Were there any pathological results observed within the physical examination?	
Date of Measurement	
Systolic(mmHg)-Value 1	
Diastolic(mmHg)-Value 1	
Pulse Rate(bpm)-Value 1	
Systolic(mmHg)-Value 2	
Diastolic(mmHg)-Value 2	
Pulse Rate(bpm)-Value 2	
Systolic(mmHg)-Value 3	
Diastolic(mmHg)-Value 3	
Pulse Rate(bpm)-Value 3	
Systolic Mean (mmHg)	0
Diastolic Mean (mmHg)	0
Pulse Rate Mean (bpm)	0
Weight (kg)	

INSERTED ON: 2011-10-10 14:47:15	BY: Demo Demo	LOCKED: NO
PRINTED ON: 2011-10-10 15:36:42	BY: Nadia Rubis	

PATIENT NUMBER: 666-01	VISIT: v14	ROW:	VISIT DATE: No date

FIELD	VALUE
Visit Date	
Date of Physical Examination	
Were there any pathological results observed within the physical examination?	
Date of Measurement	
Systolic(mmHg)-Value 1	
Diastolic(mmHg)-Value 1	
Pulse Rate(bpm)-Value 1	
Systolic(mmHg)-Value 2	
Diastolic(mmHg)-Value 2	
Pulse Rate(bpm)-Value 2	
Systolic(mmHg)-Value 3	
Diastolic(mmHg)-Value 3	
Pulse Rate(bpm)-Value 3	
Systolic Mean (mmHg)	0
Diastolic Mean (mmHg)	0
Pulse Rate Mean (bpm)	0
Weight (kg)	

INSERTED ON: 2011-10-10 14:47:20	BY: Demo Demo	LOCKED: NO
PRINTED ON: 2011-10-10 15:36:42	BY: Nadia Rubis	

BLOOD LABORATORY EXAMINATIONS FORM DATA SUMMARY	
USER: Nadia Rubis	CENTER: -

PATIENT NUMBER: 666-01	VISIT: v02	ROW:	VISIT DATE: No date

FIELD	VALUE
Date of Sample	
Was the patient fasting or not?	
Creatinine	
Urea	
BUN	
Sodium	
Potassium	
Calcium	
Phosphorus	
GOT(AST)	
GPT(ALT)	
Alkaline Phosphatase	
Gamma GT	
Total Bilirubin	
Direct Bilirubin	
CPK	
Glucose	
HbA1c	
Uric Acid	
Total Cholesterol	
HDL Cholesterol	
LDL Cholesterol	
Triglycerides	
Total Proteins	
Albumin	
IGF-1	
Leukocytes	
Platelets	
Erythrocytes	
Hemoglobin	
Hematocrit	
Creatinine Unit	
Urea Unit	
BUN Unit	

BLOOD LABORATORY EXAMINATIONS FORM DATA SUMMARY			
PATIENT NUMBER: 666-01	VISIT: v02	ROW:	VISIT DATE: No date

FIELD	VALUE
Sodium Unit	
Potassium Unit	
Calcium Unit	
Phosphorus Unit	
GOT(AST) Unit	
GPT(ALT) Unit	
Alkaline Phosphatase Unit	
Gamma GT Unit	
Total Bilirubin Unit	
Direct Bilirubin Unit	
CPK Unit	
Glucose Unit	
HbA1c Unit	
Uric Acid Unit	
Total Cholesterol Unit	
HDL Cholesterol Unit	
LDL Cholesterol Unit	
Triglycerides Unit	
Total Proteins Unit	
Albumin Unit	
IGF-1 Unit	
Leukocytes Unit	
Platelets Unit	
Erythrocytes Unit	
Hemoglobin Unit	
Hematocrit Unit	
Creatinine Assessment	
Urea Assessment	
BUN Assessment	
Sodium Assessment	
Potassium Assessment	
Calcium Assessment	
Phosphorus Assessment	
GOT(AST) Assessment	
GPT(ALT) Assessment	
Alkaline Phosphatase Assessment	
Gamma GT Assessment	
Total Bilirubin Assessment	
Direct Bilirubin Assessment	
CPK Assessment	

BLOOD LABORATORY EXAMINATIONS FORM DATA SUMMARY			
PATIENT NUMBER: 666-01	VISIT: v02	ROW:	VISIT DATE: No date

FIELD	VALUE
Glucose Assessment	
HbA1c Assessment	
Uric Acid Assessment	
Total Cholesterol Assessment	
HDL Cholesterol Assessment	
LDL Cholesterol Assessment	
Triglycerides Assessment	
Total Proteins Assessment	
Albumin Assessment	
IGF-1 Assessment	
Leukocytes Assessment	
Platelets Assessment	
Erythrocytes Assessment	
Hemoglobin Assessment	
Hematocrit Assessment	

INSERTED ON: 2011-10-10 14:47:32	BY: Demo Demo	LOCKED: NO
PRINTED ON: 2011-10-10 15:36:42	BY: Nadia Rubis	

PATIENT NUMBER: 666-01	VISIT: v03	ROW:	VISIT DATE: No date

FIELD	VALUE
Date of Sample	
Was the patient fasting or not?	
Creatinine	
Urea	
BUN	
Sodium	
Potassium	
Calcium	
Phosphorus	
GOT(AST)	
GPT(ALT)	
Alkaline Phosphatase	
Gamma GT	
Total Bilirubin	
Direct Bilirubin	
CPK	
Glucose	
HbA1c	
Uric Acid	
Total Cholesterol	
HDL Cholesterol	
LDL Cholesterol	
Triglycerides	
Total Proteins	
Albumin	
IGF-1	
Leukocytes	
Platelets	
Erythrocytes	
Hemoglobin	
Hematocrit	
Creatinine Unit	
Urea Unit	
BUN Unit	
Sodium Unit	
Potassium Unit	
Calcium Unit	
Phosphorus Unit	
GOT(AST) Unit	
GPT(ALT) Unit	

BLOOD LABORATORY EXAMINATIONS FORM DATA SUMMARY			
PATIENT NUMBER: 666-01	VISIT: v03	ROW:	VISIT DATE: No date

FIELD	VALUE
Alkaline Phosphatase Unit	
Gamma GT Unit	
Total Bilirubin Unit	
Direct Bilirubin Unit	
CPK Unit	
Glucose Unit	
HbA1c Unit	
Uric Acid Unit	
Total Cholesterol Unit	
HDL Cholesterol Unit	
LDL Cholesterol Unit	
Triglycerides Unit	
Total Proteins Unit	
Albumin Unit	
IGF-1 Unit	
Leukocytes Unit	
Platelets Unit	
Erythrocytes Unit	
Hemoglobin Unit	
Hematocrit Unit	
Creatinine Assessment	
Urea Assessment	
BUN Assessment	
Sodium Assessment	
Potassium Assessment	
Calcium Assessment	
Phosphorus Assessment	
GOT(AST) Assessment	
GPT(ALT) Assessment	
Alkaline Phosphatase Assessment	
Gamma GT Assessment	
Total Bilirubin Assessment	
Direct Bilirubin Assessment	
CPK Assessment	
Glucose Assessment	
HbA1c Assessment	
Uric Acid Assessment	
Total Cholesterol Assessment	
HDL Cholesterol Assessment	
LDL Cholesterol Assessment	

BLOOD LABORATORY EXAMINATIONS FORM DATA SUMMARY			
PATIENT NUMBER: 666-01	VISIT: v03	ROW:	VISIT DATE: No date

FIELD	VALUE
Triglycerides Assessment	
Total Proteins Assessment	
Albumin Assessment	
IGF-1 Assessment	
Leukocytes Assessment	
Platelets Assessment	
Erythrocytes Assessment	
Hemoglobin Assessment	
Hematocrit Assessment	

INSERTED ON: 2011-10-10 14:47:37	BY: Demo Demo	LOCKED: NO
PRINTED ON: 2011-10-10 15:36:42	BY: Nadia Rubis	

PATIENT NUMBER: 666-01	VISIT: v04	ROW:	VISIT DATE: No date

FIELD	VALUE
Date of Sample	
Was the patient fasting or not?	
Creatinine	
Urea	
BUN	
Sodium	
Potassium	
Calcium	
Phosphorus	
GOT(AST)	
GPT(ALT)	
Alkaline Phosphatase	
Gamma GT	
Total Bilirubin	
Direct Bilirubin	
CPK	
Glucose	
HbA1c	
Uric Acid	
Total Cholesterol	
HDL Cholesterol	
LDL Cholesterol	
Triglycerides	
Total Proteins	
Albumin	
IGF-1	
Leukocytes	
Platelets	
Erythrocytes	
Hemoglobin	
Hematocrit	
Creatinine Unit	
Urea Unit	
BUN Unit	
Sodium Unit	
Potassium Unit	
Calcium Unit	
Phosphorus Unit	
GOT(AST) Unit	
GPT(ALT) Unit	

BLOOD LABORATORY EXAMINATIONS FORM DATA SUMMARY			
PATIENT NUMBER: 666-01	VISIT: v04	ROW:	VISIT DATE: No date

FIELD	VALUE
Alkaline Phosphatase Unit	
Gamma GT Unit	
Total Bilirubin Unit	
Direct Bilirubin Unit	
CPK Unit	
Glucose Unit	
HbA1c Unit	
Uric Acid Unit	
Total Cholesterol Unit	
HDL Cholesterol Unit	
LDL Cholesterol Unit	
Triglycerides Unit	
Total Proteins Unit	
Albumin Unit	
IGF-1 Unit	
Leukocytes Unit	
Platelets Unit	
Erythrocytes Unit	
Hemoglobin Unit	
Hematocrit Unit	
Creatinine Assessment	
Urea Assessment	
BUN Assessment	
Sodium Assessment	
Potassium Assessment	
Calcium Assessment	
Phosphorus Assessment	
GOT(AST) Assessment	
GPT(ALT) Assessment	
Alkaline Phosphatase Assessment	
Gamma GT Assessment	
Total Bilirubin Assessment	
Direct Bilirubin Assessment	
CPK Assessment	
Glucose Assessment	
HbA1c Assessment	
Uric Acid Assessment	
Total Cholesterol Assessment	
HDL Cholesterol Assessment	
LDL Cholesterol Assessment	

BLOOD LABORATORY EXAMINATIONS FORM DATA SUMMARY			
PATIENT NUMBER: 666-01	VISIT: v04	ROW:	VISIT DATE: No date

FIELD	VALUE
Triglycerides Assessment	
Total Proteins Assessment	
Albumin Assessment	
IGF-1 Assessment	
Leukocytes Assessment	
Platelets Assessment	
Erythrocytes Assessment	
Hemoglobin Assessment	
Hematocrit Assessment	

INSERTED ON: 2011-10-10 14:47:43	BY: Demo Demo	LOCKED: NO
PRINTED ON: 2011-10-10 15:36:42	BY: Nadia Rubis	

PATIENT NUMBER: 666-01	VISIT: v05	ROW:	VISIT DATE: No date

FIELD	VALUE
Date of Sample	
Was the patient fasting or not?	
Creatinine	
Urea	
BUN	
Sodium	
Potassium	
Calcium	
Phosphorus	
GOT(AST)	
GPT(ALT)	
Alkaline Phosphatase	
Gamma GT	
Total Bilirubin	
Direct Bilirubin	
CPK	
Glucose	
HbA1c	
Uric Acid	
Total Cholesterol	
HDL Cholesterol	
LDL Cholesterol	
Triglycerides	
Total Proteins	
Albumin	
IGF-1	
Leukocytes	
Platelets	
Erythrocytes	
Hemoglobin	
Hematocrit	
Creatinine Unit	
Urea Unit	
BUN Unit	
Sodium Unit	
Potassium Unit	
Calcium Unit	
Phosphorus Unit	
GOT(AST) Unit	
GPT(ALT) Unit	

BLOOD LABORATORY EXAMINATIONS FORM DATA SUMMARY			
PATIENT NUMBER: 666-01	VISIT: v05	ROW:	VISIT DATE: No date

FIELD	VALUE
Alkaline Phosphatase Unit	
Gamma GT Unit	
Total Bilirubin Unit	
Direct Bilirubin Unit	
CPK Unit	
Glucose Unit	
HbA1c Unit	
Uric Acid Unit	
Total Cholesterol Unit	
HDL Cholesterol Unit	
LDL Cholesterol Unit	
Triglycerides Unit	
Total Proteins Unit	
Albumin Unit	
IGF-1 Unit	
Leukocytes Unit	
Platelets Unit	
Erythrocytes Unit	
Hemoglobin Unit	
Hematocrit Unit	
Creatinine Assessment	
Urea Assessment	
BUN Assessment	
Sodium Assessment	
Potassium Assessment	
Calcium Assessment	
Phosphorus Assessment	
GOT(AST) Assessment	
GPT(ALT) Assessment	
Alkaline Phosphatase Assessment	
Gamma GT Assessment	
Total Bilirubin Assessment	
Direct Bilirubin Assessment	
CPK Assessment	
Glucose Assessment	
HbA1c Assessment	
Uric Acid Assessment	
Total Cholesterol Assessment	
HDL Cholesterol Assessment	
LDL Cholesterol Assessment	

BLOOD LABORATORY EXAMINATIONS FORM DATA SUMMARY			
PATIENT NUMBER: 666-01	VISIT: v05	ROW:	VISIT DATE: No date

FIELD	VALUE
Triglycerides Assessment	
Total Proteins Assessment	
Albumin Assessment	
IGF-1 Assessment	
Leukocytes Assessment	
Platelets Assessment	
Erythrocytes Assessment	
Hemoglobin Assessment	
Hematocrit Assessment	

INSERTED ON: 2011-10-10 14:47:50	BY: Demo Demo	LOCKED: NO
PRINTED ON: 2011-10-10 15:36:43	BY: Nadia Rubis	

PATIENT NUMBER: 666-01	VISIT: v06	ROW:	VISIT DATE: No date

FIELD	VALUE
Date of Sample	
Was the patient fasting or not?	
Creatinine	
Urea	
BUN	
Sodium	
Potassium	
Calcium	
Phosphorus	
GOT(AST)	
GPT(ALT)	
Alkaline Phosphatase	
Gamma GT	
Total Bilirubin	
Direct Bilirubin	
CPK	
Glucose	
HbA1c	
Uric Acid	
Total Cholesterol	
HDL Cholesterol	
LDL Cholesterol	
Triglycerides	
Total Proteins	
Albumin	
IGF-1	
Leukocytes	
Platelets	
Erythrocytes	
Hemoglobin	
Hematocrit	
Creatinine Unit	
Urea Unit	
BUN Unit	
Sodium Unit	
Potassium Unit	
Calcium Unit	
Phosphorus Unit	
GOT(AST) Unit	
GPT(ALT) Unit	

BLOOD LABORATORY EXAMINATIONS FORM DATA SUMMARY			
PATIENT NUMBER: 666-01	VISIT: v06	ROW:	VISIT DATE: No date

FIELD	VALUE
Alkaline Phosphatase Unit	
Gamma GT Unit	
Total Bilirubin Unit	
Direct Bilirubin Unit	
CPK Unit	
Glucose Unit	
HbA1c Unit	
Uric Acid Unit	
Total Cholesterol Unit	
HDL Cholesterol Unit	
LDL Cholesterol Unit	
Triglycerides Unit	
Total Proteins Unit	
Albumin Unit	
IGF-1 Unit	
Leukocytes Unit	
Platelets Unit	
Erythrocytes Unit	
Hemoglobin Unit	
Hematocrit Unit	
Creatinine Assessment	
Urea Assessment	
BUN Assessment	
Sodium Assessment	
Potassium Assessment	
Calcium Assessment	
Phosphorus Assessment	
GOT(AST) Assessment	
GPT(ALT) Assessment	
Alkaline Phosphatase Assessment	
Gamma GT Assessment	
Total Bilirubin Assessment	
Direct Bilirubin Assessment	
CPK Assessment	
Glucose Assessment	
HbA1c Assessment	
Uric Acid Assessment	
Total Cholesterol Assessment	
HDL Cholesterol Assessment	
LDL Cholesterol Assessment	

BLOOD LABORATORY EXAMINATIONS FORM DATA SUMMARY			
PATIENT NUMBER: 666-01	VISIT: v06	ROW:	VISIT DATE: No date

FIELD	VALUE
Triglycerides Assessment	
Total Proteins Assessment	
Albumin Assessment	
IGF-1 Assessment	
Leukocytes Assessment	
Platelets Assessment	
Erythrocytes Assessment	
Hemoglobin Assessment	
Hematocrit Assessment	

INSERTED ON: 2011-10-10 14:47:56	BY: Demo Demo	LOCKED: NO
PRINTED ON: 2011-10-10 15:36:43	BY: Nadia Rubis	

PATIENT NUMBER: 666-01	VISIT: v07	ROW:	VISIT DATE: No date

FIELD	VALUE
Date of Sample	
Was the patient fasting or not?	
Creatinine	
Urea	
BUN	
Sodium	
Potassium	
Calcium	
Phosphorus	
GOT(AST)	
GPT(ALT)	
Alkaline Phosphatase	
Gamma GT	
Total Bilirubin	
Direct Bilirubin	
CPK	
Glucose	
HbA1c	
Uric Acid	
Total Cholesterol	
HDL Cholesterol	
LDL Cholesterol	
Triglycerides	
Total Proteins	
Albumin	
IGF-1	
Leukocytes	
Platelets	
Erythrocytes	
Hemoglobin	
Hematocrit	
Creatinine Unit	
Urea Unit	
BUN Unit	
Sodium Unit	
Potassium Unit	
Calcium Unit	
Phosphorus Unit	
GOT(AST) Unit	
GPT(ALT) Unit	

BLOOD LABORATORY EXAMINATIONS FORM DATA SUMMARY			
PATIENT NUMBER: 666-01	VISIT: v07	ROW:	VISIT DATE: No date

FIELD	VALUE
Alkaline Phosphatase Unit	
Gamma GT Unit	
Total Bilirubin Unit	
Direct Bilirubin Unit	
CPK Unit	
Glucose Unit	
HbA1c Unit	
Uric Acid Unit	
Total Cholesterol Unit	
HDL Cholesterol Unit	
LDL Cholesterol Unit	
Triglycerides Unit	
Total Proteins Unit	
Albumin Unit	
IGF-1 Unit	
Leukocytes Unit	
Platelets Unit	
Erythrocytes Unit	
Hemoglobin Unit	
Hematocrit Unit	
Creatinine Assessment	
Urea Assessment	
BUN Assessment	
Sodium Assessment	
Potassium Assessment	
Calcium Assessment	
Phosphorus Assessment	
GOT(AST) Assessment	
GPT(ALT) Assessment	
Alkaline Phosphatase Assessment	
Gamma GT Assessment	
Total Bilirubin Assessment	
Direct Bilirubin Assessment	
CPK Assessment	
Glucose Assessment	
HbA1c Assessment	
Uric Acid Assessment	
Total Cholesterol Assessment	
HDL Cholesterol Assessment	
LDL Cholesterol Assessment	

BLOOD LABORATORY EXAMINATIONS FORM DATA SUMMARY			
PATIENT NUMBER: 666-01	VISIT: v07	ROW:	VISIT DATE: No date

FIELD	VALUE
Triglycerides Assessment	
Total Proteins Assessment	
Albumin Assessment	
IGF-1 Assessment	
Leukocytes Assessment	
Platelets Assessment	
Erythrocytes Assessment	
Hemoglobin Assessment	
Hematocrit Assessment	

INSERTED ON: 2011-10-10 14:48:19	BY: Demo Demo	LOCKED: NO
PRINTED ON: 2011-10-10 15:36:43	BY: Nadia Rubis	

PATIENT NUMBER: 666-01	VISIT: v08	ROW:	VISIT DATE: No date

FIELD	VALUE
Date of Sample	
Was the patient fasting or not?	
Creatinine	
Urea	
BUN	
Sodium	
Potassium	
Calcium	
Phosphorus	
GOT(AST)	
GPT(ALT)	
Alkaline Phosphatase	
Gamma GT	
Total Bilirubin	
Direct Bilirubin	
CPK	
Glucose	
HbA1c	
Uric Acid	
Total Cholesterol	
HDL Cholesterol	
LDL Cholesterol	
Triglycerides	
Total Proteins	
Albumin	
IGF-1	
Leukocytes	
Platelets	
Erythrocytes	
Hemoglobin	
Hematocrit	
Creatinine Unit	
Urea Unit	
BUN Unit	
Sodium Unit	
Potassium Unit	
Calcium Unit	
Phosphorus Unit	
GOT(AST) Unit	
GPT(ALT) Unit	

BLOOD LABORATORY EXAMINATIONS FORM DATA SUMMARY			
PATIENT NUMBER: 666-01	VISIT: v08	ROW:	VISIT DATE: No date

FIELD	VALUE
Alkaline Phosphatase Unit	
Gamma GT Unit	
Total Bilirubin Unit	
Direct Bilirubin Unit	
CPK Unit	
Glucose Unit	
HbA1c Unit	
Uric Acid Unit	
Total Cholesterol Unit	
HDL Cholesterol Unit	
LDL Cholesterol Unit	
Triglycerides Unit	
Total Proteins Unit	
Albumin Unit	
IGF-1 Unit	
Leukocytes Unit	
Platelets Unit	
Erythrocytes Unit	
Hemoglobin Unit	
Hematocrit Unit	
Creatinine Assessment	
Urea Assessment	
BUN Assessment	
Sodium Assessment	
Potassium Assessment	
Calcium Assessment	
Phosphorus Assessment	
GOT(AST) Assessment	
GPT(ALT) Assessment	
Alkaline Phosphatase Assessment	
Gamma GT Assessment	
Total Bilirubin Assessment	
Direct Bilirubin Assessment	
CPK Assessment	
Glucose Assessment	
HbA1c Assessment	
Uric Acid Assessment	
Total Cholesterol Assessment	
HDL Cholesterol Assessment	
LDL Cholesterol Assessment	

BLOOD LABORATORY EXAMINATIONS FORM DATA SUMMARY			
PATIENT NUMBER: 666-01	VISIT: v08	ROW:	VISIT DATE: No date

FIELD	VALUE
Triglycerides Assessment	
Total Proteins Assessment	
Albumin Assessment	
IGF-1 Assessment	
Leukocytes Assessment	
Platelets Assessment	
Erythrocytes Assessment	
Hemoglobin Assessment	
Hematocrit Assessment	

INSERTED ON: 2011-10-10 14:48:26	BY: Demo Demo	LOCKED: NO
PRINTED ON: 2011-10-10 15:36:43	BY: Nadia Rubis	

PATIENT NUMBER: 666-01	VISIT: v09	ROW:	VISIT DATE: No date

FIELD	VALUE
Date of Sample	
Was the patient fasting or not?	
Creatinine	
Urea	
BUN	
Sodium	
Potassium	
Calcium	
Phosphorus	
GOT(AST)	
GPT(ALT)	
Alkaline Phosphatase	
Gamma GT	
Total Bilirubin	
Direct Bilirubin	
CPK	
Glucose	
HbA1c	
Uric Acid	
Total Cholesterol	
HDL Cholesterol	
LDL Cholesterol	
Triglycerides	
Total Proteins	
Albumin	
IGF-1	
Leukocytes	
Platelets	
Erythrocytes	
Hemoglobin	
Hematocrit	
Creatinine Unit	
Urea Unit	
BUN Unit	
Sodium Unit	
Potassium Unit	
Calcium Unit	
Phosphorus Unit	
GOT(AST) Unit	
GPT(ALT) Unit	

BLOOD LABORATORY EXAMINATIONS FORM DATA SUMMARY			
PATIENT NUMBER: 666-01	VISIT: v09	ROW:	VISIT DATE: No date

FIELD	VALUE
Alkaline Phosphatase Unit	
Gamma GT Unit	
Total Bilirubin Unit	
Direct Bilirubin Unit	
CPK Unit	
Glucose Unit	
HbA1c Unit	
Uric Acid Unit	
Total Cholesterol Unit	
HDL Cholesterol Unit	
LDL Cholesterol Unit	
Triglycerides Unit	
Total Proteins Unit	
Albumin Unit	
IGF-1 Unit	
Leukocytes Unit	
Platelets Unit	
Erythrocytes Unit	
Hemoglobin Unit	
Hematocrit Unit	
Creatinine Assessment	
Urea Assessment	
BUN Assessment	
Sodium Assessment	
Potassium Assessment	
Calcium Assessment	
Phosphorus Assessment	
GOT(AST) Assessment	
GPT(ALT) Assessment	
Alkaline Phosphatase Assessment	
Gamma GT Assessment	
Total Bilirubin Assessment	
Direct Bilirubin Assessment	
CPK Assessment	
Glucose Assessment	
HbA1c Assessment	
Uric Acid Assessment	
Total Cholesterol Assessment	
HDL Cholesterol Assessment	
LDL Cholesterol Assessment	

BLOOD LABORATORY EXAMINATIONS FORM DATA SUMMARY			
PATIENT NUMBER: 666-01	VISIT: v09	ROW:	VISIT DATE: No date

FIELD	VALUE
Triglycerides Assessment	
Total Proteins Assessment	
Albumin Assessment	
IGF-1 Assessment	
Leukocytes Assessment	
Platelets Assessment	
Erythrocytes Assessment	
Hemoglobin Assessment	
Hematocrit Assessment	

INSERTED ON: 2011-10-10 14:48:32	BY: Demo Demo	LOCKED: NO
PRINTED ON: 2011-10-10 15:36:43	BY: Nadia Rubis	

PATIENT NUMBER: 666-01	VISIT: v10	ROW:	VISIT DATE: No date

FIELD	VALUE
Date of Sample	
Was the patient fasting or not?	
Creatinine	
Urea	
BUN	
Sodium	
Potassium	
Calcium	
Phosphorus	
GOT(AST)	
GPT(ALT)	
Alkaline Phosphatase	
Gamma GT	
Total Bilirubin	
Direct Bilirubin	
CPK	
Glucose	
HbA1c	
Uric Acid	
Total Cholesterol	
HDL Cholesterol	
LDL Cholesterol	
Triglycerides	
Total Proteins	
Albumin	
IGF-1	
Leukocytes	
Platelets	
Erythrocytes	
Hemoglobin	
Hematocrit	
Creatinine Unit	
Urea Unit	
BUN Unit	
Sodium Unit	
Potassium Unit	
Calcium Unit	
Phosphorus Unit	
GOT(AST) Unit	
GPT(ALT) Unit	

BLOOD LABORATORY EXAMINATIONS FORM DATA SUMMARY			
PATIENT NUMBER: 666-01	VISIT: v10	ROW:	VISIT DATE: No date

FIELD	VALUE
Alkaline Phosphatase Unit	
Gamma GT Unit	
Total Bilirubin Unit	
Direct Bilirubin Unit	
CPK Unit	
Glucose Unit	
HbA1c Unit	
Uric Acid Unit	
Total Cholesterol Unit	
HDL Cholesterol Unit	
LDL Cholesterol Unit	
Triglycerides Unit	
Total Proteins Unit	
Albumin Unit	
IGF-1 Unit	
Leukocytes Unit	
Platelets Unit	
Erythrocytes Unit	
Hemoglobin Unit	
Hematocrit Unit	
Creatinine Assessment	
Urea Assessment	
BUN Assessment	
Sodium Assessment	
Potassium Assessment	
Calcium Assessment	
Phosphorus Assessment	
GOT(AST) Assessment	
GPT(ALT) Assessment	
Alkaline Phosphatase Assessment	
Gamma GT Assessment	
Total Bilirubin Assessment	
Direct Bilirubin Assessment	
CPK Assessment	
Glucose Assessment	
HbA1c Assessment	
Uric Acid Assessment	
Total Cholesterol Assessment	
HDL Cholesterol Assessment	
LDL Cholesterol Assessment	

BLOOD LABORATORY EXAMINATIONS FORM DATA SUMMARY			
PATIENT NUMBER: 666-01	VISIT: v10	ROW:	VISIT DATE: No date

FIELD	VALUE
Triglycerides Assessment	
Total Proteins Assessment	
Albumin Assessment	
IGF-1 Assessment	
Leukocytes Assessment	
Platelets Assessment	
Erythrocytes Assessment	
Hemoglobin Assessment	
Hematocrit Assessment	

INSERTED ON: 2011-10-10 14:48:37	BY: Demo Demo	LOCKED: NO
PRINTED ON: 2011-10-10 15:36:43	BY: Nadia Rubis	

PATIENT NUMBER: 666-01	VISIT: v11	ROW:	VISIT DATE: No date

FIELD	VALUE
Date of Sample	
Was the patient fasting or not?	
Creatinine	
Urea	
BUN	
Sodium	
Potassium	
Calcium	
Phosphorus	
GOT(AST)	
GPT(ALT)	
Alkaline Phosphatase	
Gamma GT	
Total Bilirubin	
Direct Bilirubin	
CPK	
Glucose	
HbA1c	
Uric Acid	
Total Cholesterol	
HDL Cholesterol	
LDL Cholesterol	
Triglycerides	
Total Proteins	
Albumin	
IGF-1	
Leukocytes	
Platelets	
Erythrocytes	
Hemoglobin	
Hematocrit	
Creatinine Unit	
Urea Unit	
BUN Unit	
Sodium Unit	
Potassium Unit	
Calcium Unit	
Phosphorus Unit	
GOT(AST) Unit	
GPT(ALT) Unit	

BLOOD LABORATORY EXAMINATIONS FORM DATA SUMMARY			
PATIENT NUMBER: 666-01	VISIT: v11	ROW:	VISIT DATE: No date

FIELD	VALUE
Alkaline Phosphatase Unit	
Gamma GT Unit	
Total Bilirubin Unit	
Direct Bilirubin Unit	
CPK Unit	
Glucose Unit	
HbA1c Unit	
Uric Acid Unit	
Total Cholesterol Unit	
HDL Cholesterol Unit	
LDL Cholesterol Unit	
Triglycerides Unit	
Total Proteins Unit	
Albumin Unit	
IGF-1 Unit	
Leukocytes Unit	
Platelets Unit	
Erythrocytes Unit	
Hemoglobin Unit	
Hematocrit Unit	
Creatinine Assessment	
Urea Assessment	
BUN Assessment	
Sodium Assessment	
Potassium Assessment	
Calcium Assessment	
Phosphorus Assessment	
GOT(AST) Assessment	
GPT(ALT) Assessment	
Alkaline Phosphatase Assessment	
Gamma GT Assessment	
Total Bilirubin Assessment	
Direct Bilirubin Assessment	
CPK Assessment	
Glucose Assessment	
HbA1c Assessment	
Uric Acid Assessment	
Total Cholesterol Assessment	
HDL Cholesterol Assessment	
LDL Cholesterol Assessment	

BLOOD LABORATORY EXAMINATIONS FORM DATA SUMMARY			
PATIENT NUMBER: 666-01	VISIT: v11	ROW:	VISIT DATE: No date

FIELD	VALUE
Triglycerides Assessment	
Total Proteins Assessment	
Albumin Assessment	
IGF-1 Assessment	
Leukocytes Assessment	
Platelets Assessment	
Erythrocytes Assessment	
Hemoglobin Assessment	
Hematocrit Assessment	

INSERTED ON: 2011-10-10 14:48:46	BY: Demo Demo	LOCKED: NO
PRINTED ON: 2011-10-10 15:36:43	BY: Nadia Rubis	

PATIENT NUMBER: 666-01	VISIT: v12	ROW:	VISIT DATE: No date

FIELD	VALUE
Date of Sample	
Was the patient fasting or not?	
Creatinine	
Urea	
BUN	
Sodium	
Potassium	
Calcium	
Phosphorus	
GOT(AST)	
GPT(ALT)	
Alkaline Phosphatase	
Gamma GT	
Total Bilirubin	
Direct Bilirubin	
CPK	
Glucose	
HbA1c	
Uric Acid	
Total Cholesterol	
HDL Cholesterol	
LDL Cholesterol	
Triglycerides	
Total Proteins	
Albumin	
IGF-1	
Leukocytes	
Platelets	
Erythrocytes	
Hemoglobin	
Hematocrit	
Creatinine Unit	
Urea Unit	
BUN Unit	
Sodium Unit	
Potassium Unit	
Calcium Unit	
Phosphorus Unit	
GOT(AST) Unit	
GPT(ALT) Unit	

BLOOD LABORATORY EXAMINATIONS FORM DATA SUMMARY			
PATIENT NUMBER: 666-01	VISIT: v12	ROW:	VISIT DATE: No date

FIELD	VALUE
Alkaline Phosphatase Unit	
Gamma GT Unit	
Total Bilirubin Unit	
Direct Bilirubin Unit	
CPK Unit	
Glucose Unit	
HbA1c Unit	
Uric Acid Unit	
Total Cholesterol Unit	
HDL Cholesterol Unit	
LDL Cholesterol Unit	
Triglycerides Unit	
Total Proteins Unit	
Albumin Unit	
IGF-1 Unit	
Leukocytes Unit	
Platelets Unit	
Erythrocytes Unit	
Hemoglobin Unit	
Hematocrit Unit	
Creatinine Assessment	
Urea Assessment	
BUN Assessment	
Sodium Assessment	
Potassium Assessment	
Calcium Assessment	
Phosphorus Assessment	
GOT(AST) Assessment	
GPT(ALT) Assessment	
Alkaline Phosphatase Assessment	
Gamma GT Assessment	
Total Bilirubin Assessment	
Direct Bilirubin Assessment	
CPK Assessment	
Glucose Assessment	
HbA1c Assessment	
Uric Acid Assessment	
Total Cholesterol Assessment	
HDL Cholesterol Assessment	
LDL Cholesterol Assessment	

BLOOD LABORATORY EXAMINATIONS FORM DATA SUMMARY			
PATIENT NUMBER: 666-01	VISIT: v12	ROW:	VISIT DATE: No date

FIELD	VALUE
Triglycerides Assessment	
Total Proteins Assessment	
Albumin Assessment	
IGF-1 Assessment	
Leukocytes Assessment	
Platelets Assessment	
Erythrocytes Assessment	
Hemoglobin Assessment	
Hematocrit Assessment	

INSERTED ON: 2011-10-10 14:48:53	BY: Demo Demo	LOCKED: NO
PRINTED ON: 2011-10-10 15:36:43	BY: Nadia Rubis	

PATIENT NUMBER: 666-01	VISIT: v13	ROW:	VISIT DATE: No date

FIELD	VALUE
Date of Sample	
Was the patient fasting or not?	
Creatinine	
Urea	
BUN	
Sodium	
Potassium	
Calcium	
Phosphorus	
GOT(AST)	
GPT(ALT)	
Alkaline Phosphatase	
Gamma GT	
Total Bilirubin	
Direct Bilirubin	
CPK	
Glucose	
HbA1c	
Uric Acid	
Total Cholesterol	
HDL Cholesterol	
LDL Cholesterol	
Triglycerides	
Total Proteins	
Albumin	
IGF-1	
Leukocytes	
Platelets	
Erythrocytes	
Hemoglobin	
Hematocrit	
Creatinine Unit	
Urea Unit	
BUN Unit	
Sodium Unit	
Potassium Unit	
Calcium Unit	
Phosphorus Unit	
GOT(AST) Unit	
GPT(ALT) Unit	

BLOOD LABORATORY EXAMINATIONS FORM DATA SUMMARY			
PATIENT NUMBER: 666-01	VISIT: v13	ROW:	VISIT DATE: No date

FIELD	VALUE
Alkaline Phosphatase Unit	
Gamma GT Unit	
Total Bilirubin Unit	
Direct Bilirubin Unit	
CPK Unit	
Glucose Unit	
HbA1c Unit	
Uric Acid Unit	
Total Cholesterol Unit	
HDL Cholesterol Unit	
LDL Cholesterol Unit	
Triglycerides Unit	
Total Proteins Unit	
Albumin Unit	
IGF-1 Unit	
Leukocytes Unit	
Platelets Unit	
Erythrocytes Unit	
Hemoglobin Unit	
Hematocrit Unit	
Creatinine Assessment	
Urea Assessment	
BUN Assessment	
Sodium Assessment	
Potassium Assessment	
Calcium Assessment	
Phosphorus Assessment	
GOT(AST) Assessment	
GPT(ALT) Assessment	
Alkaline Phosphatase Assessment	
Gamma GT Assessment	
Total Bilirubin Assessment	
Direct Bilirubin Assessment	
CPK Assessment	
Glucose Assessment	
HbA1c Assessment	
Uric Acid Assessment	
Total Cholesterol Assessment	
HDL Cholesterol Assessment	
LDL Cholesterol Assessment	

BLOOD LABORATORY EXAMINATIONS FORM DATA SUMMARY			
PATIENT NUMBER: 666-01	VISIT: v13	ROW:	VISIT DATE: No date

FIELD	VALUE
Triglycerides Assessment	
Total Proteins Assessment	
Albumin Assessment	
IGF-1 Assessment	
Leukocytes Assessment	
Platelets Assessment	
Erythrocytes Assessment	
Hemoglobin Assessment	
Hematocrit Assessment	

INSERTED ON: 2011-10-10 14:49:25	BY: Demo Demo	LOCKED: NO
PRINTED ON: 2011-10-10 15:36:43	BY: Nadia Rubis	

PATIENT NUMBER: 666-01	VISIT: v14	ROW:	VISIT DATE: No date

FIELD	VALUE
Date of Sample	
Was the patient fasting or not?	
Creatinine	
Urea	
BUN	
Sodium	
Potassium	
Calcium	
Phosphorus	
GOT(AST)	
GPT(ALT)	
Alkaline Phosphatase	
Gamma GT	
Total Bilirubin	
Direct Bilirubin	
CPK	
Glucose	
HbA1c	
Uric Acid	
Total Cholesterol	
HDL Cholesterol	
LDL Cholesterol	
Triglycerides	
Total Proteins	
Albumin	
IGF-1	
Leukocytes	
Platelets	
Erythrocytes	
Hemoglobin	
Hematocrit	
Creatinine Unit	
Urea Unit	
BUN Unit	
Sodium Unit	
Potassium Unit	
Calcium Unit	
Phosphorus Unit	
GOT(AST) Unit	
GPT(ALT) Unit	

BLOOD LABORATORY EXAMINATIONS FORM DATA SUMMARY			
PATIENT NUMBER: 666-01	VISIT: v14	ROW:	VISIT DATE: No date

FIELD	VALUE
Alkaline Phosphatase Unit	
Gamma GT Unit	
Total Bilirubin Unit	
Direct Bilirubin Unit	
CPK Unit	
Glucose Unit	
HbA1c Unit	
Uric Acid Unit	
Total Cholesterol Unit	
HDL Cholesterol Unit	
LDL Cholesterol Unit	
Triglycerides Unit	
Total Proteins Unit	
Albumin Unit	
IGF-1 Unit	
Leukocytes Unit	
Platelets Unit	
Erythrocytes Unit	
Hemoglobin Unit	
Hematocrit Unit	
Creatinine Assessment	
Urea Assessment	
BUN Assessment	
Sodium Assessment	
Potassium Assessment	
Calcium Assessment	
Phosphorus Assessment	
GOT(AST) Assessment	
GPT(ALT) Assessment	
Alkaline Phosphatase Assessment	
Gamma GT Assessment	
Total Bilirubin Assessment	
Direct Bilirubin Assessment	
CPK Assessment	
Glucose Assessment	
HbA1c Assessment	
Uric Acid Assessment	
Total Cholesterol Assessment	
HDL Cholesterol Assessment	
LDL Cholesterol Assessment	

BLOOD LABORATORY EXAMINATIONS FORM DATA SUMMARY			
PATIENT NUMBER: 666-01	VISIT: v14	ROW:	VISIT DATE: No date

FIELD	VALUE
Triglycerides Assessment	
Total Proteins Assessment	
Albumin Assessment	
IGF-1 Assessment	
Leukocytes Assessment	
Platelets Assessment	
Erythrocytes Assessment	
Hemoglobin Assessment	
Hematocrit Assessment	

INSERTED ON: 2011-10-10 14:49:44	BY: Demo Demo	LOCKED: NO
PRINTED ON: 2011-10-10 15:36:43	BY: Nadia Rubis	

SPIRAL COMPUTED TOMOGRAPHY FORM DATA SUMMARY	
USER: Nadia Rubis	CENTER: -

PATIENT NUMBER: 666-01	VISIT: v02	ROW:	VISIT DATE: No date

FIELD	VALUE
Date of Examination	
Pathological Findings	
Pathological Findings Specify	
Total Kidney Volume	
Total Renal Cyst Volume	
Total Renal Intermediate Volume	
Total Renal Parenchyma Volume	
Total Liver Volume	
Total Liver Cyst Volume	
Total Kidney Volume Unit	
Total Renal Cyst Volume Unit	
Total Renal Intermediate Volume Unit	
Total Renal Parenchyma Volume Unit	
Total Liver Volume Unit	
Total Liver Cyst Volume Unit	
Notes	

INSERTED ON: 2011-10-10 14:49:53	BY: Demo Demo	LOCKED: NO
PRINTED ON: 2011-10-10 15:36:43	BY: Nadia Rubis	

PATIENT NUMBER: 666-01	VISIT: v06	ROW:	VISIT DATE: No date

FIELD	VALUE
Date of Examination	
Pathological Findings	
Pathological Findings Specify	
Total Kidney Volume	
Total Renal Cyst Volume	
Total Renal Intermediate Volume	
Total Renal Parenchyma Volume	
Total Liver Volume	
Total Liver Cyst Volume	
Total Kidney Volume Unit	
Total Renal Cyst Volume Unit	
Total Renal Intermediate Volume Unit	
Total Renal Parenchyma Volume Unit	
Total Liver Volume Unit	
Total Liver Cyst Volume Unit	
Notes	

INSERTED ON: 2011-10-10 14:49:57	BY: Demo Demo	LOCKED: NO
PRINTED ON: 2011-10-10 15:36:43	BY: Nadia Rubis	

PATIENT NUMBER: 666-01	VISIT: v14	ROW:	VISIT DATE: No date

FIELD	VALUE
Date of Examination	
Pathological Findings	
Pathological Findings Specify	
Total Kidney Volume	
Total Renal Cyst Volume	
Total Renal Intermediate Volume	
Total Renal Parenchyma Volume	
Total Liver Volume	
Total Liver Cyst Volume	
Total Kidney Volume Unit	
Total Renal Cyst Volume Unit	
Total Renal Intermediate Volume Unit	
Total Renal Parenchyma Volume Unit	
Total Liver Volume Unit	
Total Liver Cyst Volume Unit	
Notes	

INSERTED ON: 2011-10-10 14:50:01	BY: Demo Demo	LOCKED: NO
PRINTED ON: 2011-10-10 15:36:43	BY: Nadia Rubis	

GALLBLADDER/KIDNEY ULTRASONOGRAPHY FORM DATA SUMMARY	
USER: Nadia Rubis	CENTER: -

PATIENT NUMBER: 666-01	VISIT: v02	ROW:	VISIT DATE: No date

FIELD	VALUE
Date of Gallbladder Ultrasonography	
Gallbladder Sand	
Gallbladder Stones	
Other Gallbladder Pathological Findings	
Other Gallbladder Pathological Findings Specify	
Date of Kidney Ultrasonography	
Renal Stones	
Other Kidney Pathological Findings	
Other Kidney Pathological Findings Specify	

INSERTED ON: 2011-10-10 14:50:09	BY: Demo Demo	LOCKED: NO
PRINTED ON: 2011-10-10 15:36:43	BY: Nadia Rubis	

PATIENT NUMBER: 666-01	VISIT: v03	ROW:	VISIT DATE: No date

FIELD	VALUE
Date of Gallbladder Ultrasonography	
Gallbladder Sand	
Gallbladder Stones	
Other Gallbladder Pathological Findings	
Other Gallbladder Pathological Findings Specify	
Date of Kidney Ultrasonography	
Renal Stones	
Other Kidney Pathological Findings	
Other Kidney Pathological Findings Specify	

INSERTED ON: 2011-10-10 14:50:16	BY: Demo Demo	LOCKED: NO
PRINTED ON: 2011-10-10 15:36:43	BY: Nadia Rubis	

PATIENT NUMBER: 666-01	VISIT: v04	ROW:	VISIT DATE: No date

FIELD	VALUE
Date of Gallbladder Ultrasonography	
Gallbladder Sand	
Gallbladder Stones	
Other Gallbladder Pathological Findings	
Other Gallbladder Pathological Findings Specify	
Date of Kidney Ultrasonography	
Renal Stones	
Other Kidney Pathological Findings	
Other Kidney Pathological Findings Specify	

INSERTED ON: 2011-10-10 14:50:21	BY: Demo Demo	LOCKED: NO
PRINTED ON: 2011-10-10 15:36:43	BY: Nadia Rubis	

PATIENT NUMBER: 666-01	VISIT: v05	ROW:	VISIT DATE: No date

FIELD	VALUE
Date of Gallbladder Ultrasonography	
Gallbladder Sand	
Gallbladder Stones	
Other Gallbladder Pathological Findings	
Other Gallbladder Pathological Findings Specify	
Date of Kidney Ultrasonography	
Renal Stones	
Other Kidney Pathological Findings	
Other Kidney Pathological Findings Specify	

INSERTED ON: 2011-10-10 14:50:28	BY: Demo Demo	LOCKED: NO
PRINTED ON: 2011-10-10 15:36:43	BY: Nadia Rubis	

PATIENT NUMBER: 666-01	VISIT: v06	ROW:	VISIT DATE: No date

FIELD	VALUE
Date of Gallbladder Ultrasonography	
Gallbladder Sand	
Gallbladder Stones	
Other Gallbladder Pathological Findings	
Other Gallbladder Pathological Findings Specify	
Date of Kidney Ultrasonography	
Renal Stones	
Other Kidney Pathological Findings	
Other Kidney Pathological Findings Specify	

INSERTED ON: 2011-10-10 14:50:34	BY: Demo Demo	LOCKED: NO
PRINTED ON: 2011-10-10 15:36:43	BY: Nadia Rubis	

PATIENT NUMBER: 666-01	VISIT: v07	ROW:	VISIT DATE: No date

FIELD	VALUE
Date of Gallbladder Ultrasonography	
Gallbladder Sand	
Gallbladder Stones	
Other Gallbladder Pathological Findings	
Other Gallbladder Pathological Findings Specify	
Date of Kidney Ultrasonography	
Renal Stones	
Other Kidney Pathological Findings	
Other Kidney Pathological Findings Specify	

INSERTED ON: 2011-10-10 14:50:40	BY: Demo Demo	LOCKED: NO
PRINTED ON: 2011-10-10 15:36:43	BY: Nadia Rubis	

PATIENT NUMBER: 666-01	VISIT: v08	ROW:	VISIT DATE: No date

FIELD	VALUE
Date of Gallbladder Ultrasonography	
Gallbladder Sand	
Gallbladder Stones	
Other Gallbladder Pathological Findings	
Other Gallbladder Pathological Findings Specify	
Date of Kidney Ultrasonography	
Renal Stones	
Other Kidney Pathological Findings	
Other Kidney Pathological Findings Specify	

INSERTED ON: 2011-10-10 14:50:46	BY: Demo Demo	LOCKED: NO
PRINTED ON: 2011-10-10 15:36:43	BY: Nadia Rubis	

PATIENT NUMBER: 666-01	VISIT: v09	ROW:	VISIT DATE: No date

FIELD	VALUE
Date of Gallbladder Ultrasonography	
Gallbladder Sand	
Gallbladder Stones	
Other Gallbladder Pathological Findings	
Other Gallbladder Pathological Findings Specify	
Date of Kidney Ultrasonography	
Renal Stones	
Other Kidney Pathological Findings	
Other Kidney Pathological Findings Specify	

INSERTED ON: 2011-10-10 14:50:52	BY: Demo Demo	LOCKED: NO
PRINTED ON: 2011-10-10 15:36:43	BY: Nadia Rubis	

PATIENT NUMBER: 666-01	VISIT: v10	ROW:	VISIT DATE: No date

FIELD	VALUE
Date of Gallbladder Ultrasonography	
Gallbladder Sand	
Gallbladder Stones	
Other Gallbladder Pathological Findings	
Other Gallbladder Pathological Findings Specify	
Date of Kidney Ultrasonography	
Renal Stones	
Other Kidney Pathological Findings	
Other Kidney Pathological Findings Specify	

INSERTED ON: 2011-10-10 14:50:58	BY: Demo Demo	LOCKED: NO
PRINTED ON: 2011-10-10 15:36:43	BY: Nadia Rubis	

PATIENT NUMBER: 666-01	VISIT: v11	ROW:	VISIT DATE: No date

FIELD	VALUE
Date of Gallbladder Ultrasonography	
Gallbladder Sand	
Gallbladder Stones	
Other Gallbladder Pathological Findings	
Other Gallbladder Pathological Findings Specify	
Date of Kidney Ultrasonography	
Renal Stones	
Other Kidney Pathological Findings	
Other Kidney Pathological Findings Specify	

INSERTED ON: 2011-10-10 14:51:06	BY: Demo Demo	LOCKED: NO
PRINTED ON: 2011-10-10 15:36:43	BY: Nadia Rubis	

PATIENT NUMBER: 666-01	VISIT: v12	ROW:	VISIT DATE: No date

FIELD	VALUE
Date of Gallbladder Ultrasonography	
Gallbladder Sand	
Gallbladder Stones	
Other Gallbladder Pathological Findings	
Other Gallbladder Pathological Findings Specify	
Date of Kidney Ultrasonography	
Renal Stones	
Other Kidney Pathological Findings	
Other Kidney Pathological Findings Specify	

INSERTED ON: 2011-10-10 14:51:12	BY: Demo Demo	LOCKED: NO
PRINTED ON: 2011-10-10 15:36:43	BY: Nadia Rubis	

PATIENT NUMBER: 666-01	VISIT: v13	ROW:	VISIT DATE: No date

FIELD	VALUE
Date of Gallbladder Ultrasonography	
Gallbladder Sand	
Gallbladder Stones	
Other Gallbladder Pathological Findings	
Other Gallbladder Pathological Findings Specify	
Date of Kidney Ultrasonography	
Renal Stones	
Other Kidney Pathological Findings	
Other Kidney Pathological Findings Specify	

INSERTED ON: 2011-10-10 14:51:18	BY: Demo Demo	LOCKED: NO
PRINTED ON: 2011-10-10 15:36:43	BY: Nadia Rubis	

PATIENT NUMBER: 666-01	VISIT: v14	ROW:	VISIT DATE: No date

FIELD	VALUE
Date of Gallbladder Ultrasonography	
Gallbladder Sand	
Gallbladder Stones	
Other Gallbladder Pathological Findings	
Other Gallbladder Pathological Findings Specify	
Date of Kidney Ultrasonography	
Renal Stones	
Other Kidney Pathological Findings	
Other Kidney Pathological Findings Specify	

INSERTED ON: 2011-10-10 14:51:23	BY: Demo Demo	LOCKED: NO
PRINTED ON: 2011-10-10 15:36:43	BY: Nadia Rubis	

24H URINE COLLECTIONS - OVERNIGHT URINE COLLECTION - PAH/IOHEXOL PLASMA CLEARANCE FORM DATA SUMMARY	
USER: Nadia Rubis	CENTER: -

PATIENT NUMBER: 666-01	VISIT: v02	ROW:	VISIT DATE: No date

FIELD	VALUE
End Date (24H Urine Collection)	
Duration-HH (24H Urine Collection)	
Duration-MM (24H Urine Collection)	
Volume (24H Urine Collection)	
Proteins	
Sodium	
Urea	
Glucose	
Phosphorus	
Creatinine	
Creatinine Clearance	
End Date (Overnight Urine Collection)	
Duration-HH (Overnight Urine Collection)	
Duration-MM (Overnight Urine Collection)	
Volume (Overnight Urine Collection)	
Endothelin Excretion	
MCP-1 Excretion	
End Date (Pah-Iohexol Plasma Clearance)	
GFR	
RPF	
Proteins Unit	
Sodium Unit	
Urea Unit	
Glucose Unit	
Phosphorus Unit	
Creatinine Unit	
Creatinine Clearance Unit	
Endothelin Excretion Unit	
MCP-1 Excretion Unit	
GFR Unit	
RPF Unit	

INSERTED ON: 2011-10-10 14:51:31	BY: Demo Demo	LOCKED: NO
PRINTED ON: 2011-10-10 15:36:43	BY: Nadia Rubis	

PATIENT NUMBER: 666-01	VISIT: v04	ROW:	VISIT DATE: No date

FIELD	VALUE
End Date (24H Urine Collection)	
Duration-HH (24H Urine Collection)	
Duration-MM (24H Urine Collection)	
Volume (24H Urine Collection)	
Proteins	
Sodium	
Urea	
Glucose	
Phosphorus	
Creatinine	
Creatinine Clearance	
End Date (Overnight Urine Collection)	
Duration-HH (Overnight Urine Collection)	
Duration-MM (Overnight Urine Collection)	
Volume (Overnight Urine Collection)	
Endothelin Excretion	
MCP-1 Excretion	
End Date (Pah-Iohexol Plasma Clearance)	
GFR	
RPF	
Proteins Unit	
Sodium Unit	
Urea Unit	
Glucose Unit	
Phosphorus Unit	
Creatinine Unit	
Creatinine Clearance Unit	
Endothelin Excretion Unit	
MCP-1 Excretion Unit	
GFR Unit	
RPF Unit	

INSERTED ON: 2011-10-10 14:51:36	BY: Demo Demo	LOCKED: NO
PRINTED ON: 2011-10-10 15:36:43	BY: Nadia Rubis	

PATIENT NUMBER: 666-01	VISIT: v06	ROW:	VISIT DATE: No date

FIELD	VALUE
End Date (24H Urine Collection)	
Duration-HH (24H Urine Collection)	
Duration-MM (24H Urine Collection)	
Volume (24H Urine Collection)	
Proteins	
Sodium	
Urea	
Glucose	
Phosphorus	
Creatinine	
Creatinine Clearance	
End Date (Overnight Urine Collection)	
Duration-HH (Overnight Urine Collection)	
Duration-MM (Overnight Urine Collection)	
Volume (Overnight Urine Collection)	
Endothelin Excretion	
MCP-1 Excretion	
End Date (Pah-Iohexol Plasma Clearance)	
GFR	
RPF	
Proteins Unit	
Sodium Unit	
Urea Unit	
Glucose Unit	
Phosphorus Unit	
Creatinine Unit	
Creatinine Clearance Unit	
Endothelin Excretion Unit	
MCP-1 Excretion Unit	
GFR Unit	
RPF Unit	

INSERTED ON: 2011-10-10 14:51:42	BY: Demo Demo	LOCKED: NO
PRINTED ON: 2011-10-10 15:36:44	BY: Nadia Rubis	

PATIENT NUMBER: 666-01	VISIT: v08	ROW:	VISIT DATE: No date

FIELD	VALUE
End Date (24H Urine Collection)	
Duration-HH (24H Urine Collection)	
Duration-MM (24H Urine Collection)	
Volume (24H Urine Collection)	
Proteins	
Sodium	
Urea	
Glucose	
Phosphorus	
Creatinine	
Creatinine Clearance	
End Date (Overnight Urine Collection)	
Duration-HH (Overnight Urine Collection)	
Duration-MM (Overnight Urine Collection)	
Volume (Overnight Urine Collection)	
Endothelin Excretion	
MCP-1 Excretion	
End Date (Pah-Iohexol Plasma Clearance)	
GFR	
RPF	
Proteins Unit	
Sodium Unit	
Urea Unit	
Glucose Unit	
Phosphorus Unit	
Creatinine Unit	
Creatinine Clearance Unit	
Endothelin Excretion Unit	
MCP-1 Excretion Unit	
GFR Unit	
RPF Unit	

INSERTED ON: 2011-10-10 14:51:49	BY: Demo Demo	LOCKED: NO
PRINTED ON: 2011-10-10 15:36:44	BY: Nadia Rubis	

PATIENT NUMBER: 666-01	VISIT: v10	ROW:	VISIT DATE: No date

FIELD	VALUE
End Date (24H Urine Collection)	
Duration-HH (24H Urine Collection)	
Duration-MM (24H Urine Collection)	
Volume (24H Urine Collection)	
Proteins	
Sodium	
Urea	
Glucose	
Phosphorus	
Creatinine	
Creatinine Clearance	
End Date (Overnight Urine Collection)	
Duration-HH (Overnight Urine Collection)	
Duration-MM (Overnight Urine Collection)	
Volume (Overnight Urine Collection)	
Endothelin Excretion	
MCP-1 Excretion	
End Date (Pah-Iohexol Plasma Clearance)	
GFR	
RPF	
Proteins Unit	
Sodium Unit	
Urea Unit	
Glucose Unit	
Phosphorus Unit	
Creatinine Unit	
Creatinine Clearance Unit	
Endothelin Excretion Unit	
MCP-1 Excretion Unit	
GFR Unit	
RPF Unit	

INSERTED ON: 2011-10-10 14:52:01	BY: Demo Demo	LOCKED: NO
PRINTED ON: 2011-10-10 15:36:44	BY: Nadia Rubis	

PATIENT NUMBER: 666-01	VISIT: v12	ROW:	VISIT DATE: No date

FIELD	VALUE
End Date (24H Urine Collection)	
Duration-HH (24H Urine Collection)	
Duration-MM (24H Urine Collection)	
Volume (24H Urine Collection)	
Proteins	
Sodium	
Urea	
Glucose	
Phosphorus	
Creatinine	
Creatinine Clearance	
End Date (Overnight Urine Collection)	
Duration-HH (Overnight Urine Collection)	
Duration-MM (Overnight Urine Collection)	
Volume (Overnight Urine Collection)	
Endothelin Excretion	
MCP-1 Excretion	
End Date (Pah-Iohexol Plasma Clearance)	
GFR	
RPF	
Proteins Unit	
Sodium Unit	
Urea Unit	
Glucose Unit	
Phosphorus Unit	
Creatinine Unit	
Creatinine Clearance Unit	
Endothelin Excretion Unit	
MCP-1 Excretion Unit	
GFR Unit	
RPF Unit	

INSERTED ON: 2011-10-10 14:52:07	BY: Demo Demo	LOCKED: NO
PRINTED ON: 2011-10-10 15:36:44	BY: Nadia Rubis	

PATIENT NUMBER: 666-01	VISIT: v14	ROW:	VISIT DATE: No date

FIELD	VALUE
End Date (24H Urine Collection)	
Duration-HH (24H Urine Collection)	
Duration-MM (24H Urine Collection)	
Volume (24H Urine Collection)	
Proteins	
Sodium	
Urea	
Glucose	
Phosphorus	
Creatinine	
Creatinine Clearance	
End Date (Overnight Urine Collection)	
Duration-HH (Overnight Urine Collection)	
Duration-MM (Overnight Urine Collection)	
Volume (Overnight Urine Collection)	
Endothelin Excretion	
MCP-1 Excretion	
End Date (Pah-Iohexol Plasma Clearance)	
GFR	
RPF	
Proteins Unit	
Sodium Unit	
Urea Unit	
Glucose Unit	
Phosphorus Unit	
Creatinine Unit	
Creatinine Clearance Unit	
Endothelin Excretion Unit	
MCP-1 Excretion Unit	
GFR Unit	
RPF Unit	

INSERTED ON: 2011-10-10 14:52:11	BY: Demo Demo	LOCKED: NO
PRINTED ON: 2011-10-10 15:36:44	BY: Nadia Rubis	

URINE ANALYSIS FORM DATA SUMMARY	
USER: Nadia Rubis	CENTER: -

PATIENT NUMBER: 666-01	VISIT: v02	ROW:	VISIT DATE: No date

FIELD	VALUE
Date of Sample	
Proteins	
Albumin	
Creatinine	
Proteins Unit	
Albumin Unit	
Creatinine Unit	

INSERTED ON: 2011-10-10 14:52:24	BY: Demo Demo	LOCKED: NO
PRINTED ON: 2011-10-10 15:36:44	BY: Nadia Rubis	

PATIENT NUMBER: 666-01	VISIT: v03	ROW:	VISIT DATE: No date

FIELD	VALUE
Date of Sample	
Proteins	
Albumin	
Creatinine	
Proteins Unit	
Albumin Unit	
Creatinine Unit	

INSERTED ON: 2011-10-10 14:52:28	BY: Demo Demo	LOCKED: NO
PRINTED ON: 2011-10-10 15:36:44	BY: Nadia Rubis	

PATIENT NUMBER: 666-01	VISIT: v04	ROW:	VISIT DATE: No date

FIELD	VALUE
Date of Sample	
Proteins	
Albumin	
Creatinine	
Proteins Unit	
Albumin Unit	
Creatinine Unit	

INSERTED ON: 2011-10-10 14:52:31	BY: Demo Demo	LOCKED: NO
PRINTED ON: 2011-10-10 15:36:44	BY: Nadia Rubis	

PATIENT NUMBER: 666-01	VISIT: v05	ROW:	VISIT DATE: No date

FIELD	VALUE
Date of Sample	
Proteins	
Albumin	
Creatinine	
Proteins Unit	
Albumin Unit	
Creatinine Unit	

INSERTED ON: 2011-10-10 14:52:35	BY: Demo Demo	LOCKED: NO
PRINTED ON: 2011-10-10 15:36:44	BY: Nadia Rubis	

PATIENT NUMBER: 666-01	VISIT: v06	ROW:	VISIT DATE: No date

FIELD	VALUE
Date of Sample	
Proteins	
Albumin	
Creatinine	
Proteins Unit	
Albumin Unit	
Creatinine Unit	

INSERTED ON: 2011-10-10 14:52:48	BY: Demo Demo	LOCKED: NO
PRINTED ON: 2011-10-10 15:36:44	BY: Nadia Rubis	

PATIENT NUMBER: 666-01	VISIT: v07	ROW:	VISIT DATE: No date

FIELD	VALUE
Date of Sample	
Proteins	
Albumin	
Creatinine	
Proteins Unit	
Albumin Unit	
Creatinine Unit	

INSERTED ON: 2011-10-10 14:52:52	BY: Demo Demo	LOCKED: NO
PRINTED ON: 2011-10-10 15:36:44	BY: Nadia Rubis	

PATIENT NUMBER: 666-01	VISIT: v08	ROW:	VISIT DATE: No date

FIELD	VALUE
Date of Sample	
Proteins	
Albumin	
Creatinine	
Proteins Unit	
Albumin Unit	
Creatinine Unit	

INSERTED ON: 2011-10-10 14:52:55	BY: Demo Demo	LOCKED: NO
PRINTED ON: 2011-10-10 15:36:44	BY: Nadia Rubis	

PATIENT NUMBER: 666-01	VISIT: v09	ROW:	VISIT DATE: No date

FIELD	VALUE
Date of Sample	
Proteins	
Albumin	
Creatinine	
Proteins Unit	
Albumin Unit	
Creatinine Unit	

INSERTED ON: 2011-10-10 14:52:59	BY: Demo Demo	LOCKED: NO
PRINTED ON: 2011-10-10 15:36:44	BY: Nadia Rubis	

PATIENT NUMBER: 666-01	VISIT: v10	ROW:	VISIT DATE: No date

FIELD	VALUE
Date of Sample	
Proteins	
Albumin	
Creatinine	
Proteins Unit	
Albumin Unit	
Creatinine Unit	

INSERTED ON: 2011-10-10 14:53:03	BY: Demo Demo	LOCKED: NO
PRINTED ON: 2011-10-10 15:36:44	BY: Nadia Rubis	

PATIENT NUMBER: 666-01	VISIT: v11	ROW:	VISIT DATE: No date

FIELD	VALUE
Date of Sample	
Proteins	
Albumin	
Creatinine	
Proteins Unit	
Albumin Unit	
Creatinine Unit	

INSERTED ON: 2011-10-10 14:53:06	BY: Demo Demo	LOCKED: NO
PRINTED ON: 2011-10-10 15:36:44	BY: Nadia Rubis	

PATIENT NUMBER: 666-01	VISIT: v12	ROW:	VISIT DATE: No date

FIELD	VALUE
Date of Sample	
Proteins	
Albumin	
Creatinine	
Proteins Unit	
Albumin Unit	
Creatinine Unit	

INSERTED ON: 2011-10-10 14:53:10	BY: Demo Demo	LOCKED: NO
PRINTED ON: 2011-10-10 15:36:44	BY: Nadia Rubis	

PATIENT NUMBER: 666-01	VISIT: v13	ROW:	VISIT DATE: No date

FIELD	VALUE
Date of Sample	
Proteins	
Albumin	
Creatinine	
Proteins Unit	
Albumin Unit	
Creatinine Unit	

INSERTED ON: 2011-10-10 14:53:14	BY: Demo Demo	LOCKED: NO
PRINTED ON: 2011-10-10 15:36:44	BY: Nadia Rubis	

PATIENT NUMBER: 666-01	VISIT: v14	ROW:	VISIT DATE: No date

FIELD	VALUE
Date of Sample	
Proteins	
Albumin	
Creatinine	
Proteins Unit	
Albumin Unit	
Creatinine Unit	

INSERTED ON: 2011-10-10 14:53:18	BY: Demo Demo	LOCKED: NO
PRINTED ON: 2011-10-10 15:36:44	BY: Nadia Rubis	

ELIGIBILITY TESTS FORM DATA SUMMARY	
USER: Nadia Rubis	CENTER: -

PATIENT NUMBER: 666-01	VISIT: v01	ROW:	VISIT DATE: No date

FIELD	VALUE
Date of Sample (Blood Exam)	
Creatinine	
Glucose	
HbA1c	
Creatinine Unit	
Glucose Unit	
HbA1c Unit	
Date of Sample (24H Urine)	
Proteins	
Proteins Unit	
GFR	0
Date of Examination (Kidney Ultrasonography)	
ADPKD	
Other Pathological Findings (Kidney Ultrasonography)	
Other Pathological Findings Specify	
Date of Examination (Liver Ultrasonography)	
Liver Polycistosis	
Other Pathological Findings (Liver Ultrasonography)	
Other Pathological Findings Specify	

INSERTED ON: 2011-10-10 14:53:28	BY: Demo Demo	LOCKED: NO
PRINTED ON: 2011-10-10 15:36:44	BY: Nadia Rubis	

INCLUSION/EXCLUSION CRITERIA FORM DATA SUMMARY	
USER: Nadia Rubis	CENTER: -

PATIENT NUMBER: 666-01	VISIT: v02	ROW:	VISIT DATE: No date

FIELD	VALUE
Age > 18 years	
Clinical and ultrasound diagnosis of ADPKD	
Estimated GFR between 15 and 40 mL/min/1.73m² (by the MDRD 4 variable equation)	
Written informed consent	
24-h urinary protein excretion rate > 3g (suggestive of a concomitant glomerular disease that could benefit of specific therapy)	
Symptomatic urinary tract lithiasis or obstruction	
Uncontrolled diabetes mellitus (HbA1c > 8%) or hypertension (systolic/diastolic BP > 180/110 mmHg)	
Current urinary tract infection	
Symptomatic biliary tract lithiasis	
Active cancer	
Psychiatric disorders or any condition that might prevent full comprehension of the purposes and risks of the study	
Pregnancy, lactation or child bearing potential and ineffective contraception (estrogen therapy in post menopausal women should not be stopped)	

INSERTED ON: 2011-10-10 14:53:35	BY: Demo Demo	LOCKED: NO
PRINTED ON: 2011-10-10 15:36:44	BY: Nadia Rubis	

RANDOMIZATION FORM DATA SUMMARY	
USER: Nadia Rubis	CENTER: -

PATIENT NUMBER: 666-01	VISIT: v02	ROW:	VISIT DATE: No date

FIELD	VALUE
Has the patient been randomized?	
Randomization Date	
Patient Number	
Protocol Violation	
Inadherence to In/Exclusion Criteria	
Lost to Follow-up	
Withdrawal of Consent	
Concomitant Disease	
Death	

INSERTED ON: 2011-10-10 14:53:40	BY: Demo Demo	LOCKED: NO
PRINTED ON: 2011-10-10 15:36:44	BY: Nadia Rubis	

INVESTIGATIONAL MEDICINAL PRODUCT ADMINISTRATION FORM DATA SUMMARY

USER: Nadia Rubis	CENTER: -

PATIENT NUMBER: 666-01	VISIT:	ROW:	VISIT DATE: No date

FIELD	VALUE
Row 1 Yes/No	
Row 2 Yes/No	
Row 3 Yes/No	
Row 4 Yes/No	
Row 5 Yes/No	
Row 6 Yes/No	
Row 7 Yes/No	
Row 8 Yes/No	
Row 9 Yes/No	
Row 10 Yes/No	
Row 11 Yes/No	
Row 12 Yes/No	
Row 13 Yes/No	
Row 14 Yes/No	
Row 15 Yes/No	
Row 16 Yes/No	
Row 17 Yes/No	
Row 18 Yes/No	
Row 19 Yes/No	
Row 20 Yes/No	
Row 21 Yes/No	
Row 22 Yes/No	
Row 23 Yes/No	
Row 24 Yes/No	
Row 25 Yes/No	
Row 26 Yes/No	
Row 27 Yes/No	
Row 28 Yes/No	
Row 29 Yes/No	
Row 30 Yes/No	
Row 31 Yes/No	
Row 32 Yes/No	
Row 33 Yes/No	
Row 34 Yes/No	

INVESTIGATIONAL MEDICINAL PRODUCT ADMINISTRATION FORM DATA SUMMARY			
PATIENT NUMBER: 666-01	VISIT:	ROW:	VISIT DATE: No date

FIELD	VALUE
Row 35 Yes/No	
Row 36 Yes/No	
Row 37 Yes/No	
Row 38 Yes/No	
Row 39 Yes/No	
Row 40 Yes/No	
Row 1 Date	
Row 2 Date	
Row 3 Date	
Row 4 Date	
Row 5 Date	
Row 6 Date	
Row 7 Date	
Row 8 Date	
Row 9 Date	
Row 10 Date	
Row 11 Date	
Row 12 Date	
Row 13 Date	
Row 14 Date	
Row 15 Date	
Row 16 Date	
Row 17 Date	
Row 18 Date	
Row 19 Date	
Row 20 Date	
Row 21 Date	
Row 22 Date	
Row 23 Date	
Row 24 Date	
Row 25 Date	
Row 26 Date	
Row 27 Date	
Row 28 Date	
Row 29 Date	
Row 30 Date	
Row 31 Date	
Row 32 Date	
Row 33 Date	
Row 34 Date	

INVESTIGATIONAL MEDICINAL PRODUCT ADMINISTRATION FORM DATA SUMMARY			
PATIENT NUMBER: 666-01	VISIT:	ROW:	VISIT DATE: No date

FIELD	VALUE
Row 35 Date	
Row 36 Date	
Row 37 Date	
Row 38 Date	
Row 39 Date	
Row 40 Date	
Row 1 Vial	
Row 2 Vial	
Row 3 Vial	
Row 4 Vial	
Row 5 Vial	
Row 6 Vial	
Row 7 Vial	
Row 8 Vial	
Row 9 Vial	
Row 10 Vial	
Row 11 Vial	
Row 12 Vial	
Row 13 Vial	
Row 14 Vial	
Row 15 Vial	
Row 16 Vial	
Row 17 Vial	
Row 18 Vial	
Row 19 Vial	
Row 20 Vial	
Row 21 Vial	
Row 22 Vial	
Row 23 Vial	
Row 24 Vial	
Row 25 Vial	
Row 26 Vial	
Row 27 Vial	
Row 28 Vial	
Row 29 Vial	
Row 30 Vial	
Row 31 Vial	
Row 32 Vial	
Row 33 Vial	
Row 34 Vial	

INVESTIGATIONAL MEDICINAL PRODUCT ADMINISTRATION FORM DATA SUMMARY			
PATIENT NUMBER: 666-01	VISIT:	ROW:	VISIT DATE: No date

FIELD	VALUE
Row 35 Vial	
Row 36 Vial	
Row 37 Vial	
Row 38 Vial	
Row 39 Vial	
Row 40 Vial	

INSERTED ON: 2011-10-10 14:53:45	BY: Demo Demo	LOCKED: NO
PRINTED ON: 2011-10-10 15:36:44	BY: Nadia Rubis	

CONCOMITANT TREATMENTS FORM DATA SUMMARY	
USER: Nadia Rubis	CENTER: -

PATIENT NUMBER: 666-01	VISIT:	ROW: 1	VISIT DATE: No date

FIELD	VALUE
Medication	
First Intake Date	
Last Intake Date	
First Intake Date Unknown	
Last Intake Date Unknown	
Last Intake Date Ongoing	
Dose	
Schedule	
Route	
Disease/Symptom	

INSERTED ON: 2011-10-10 14:53:54	BY: Demo Demo	LOCKED: NO
PRINTED ON: 2011-10-10 15:36:44	BY: Nadia Rubis	

PATIENT NUMBER: 666-01	VISIT:	ROW: 2	VISIT DATE: No date

FIELD	VALUE
Medication	
First Intake Date	
Last Intake Date	
First Intake Date Unknown	
Last Intake Date Unknown	
Last Intake Date Ongoing	
Dose	
Schedule	
Route	
Disease/Symptom	

INSERTED ON: 2011-10-10 14:53:59	BY: Demo Demo	LOCKED: NO
PRINTED ON: 2011-10-10 15:36:44	BY: Nadia Rubis	

PATIENT NUMBER: 666-01	VISIT:	ROW: 3	VISIT DATE: No date

FIELD	VALUE
Medication	
First Intake Date	
Last Intake Date	
First Intake Date Unknown	
Last Intake Date Unknown	
Last Intake Date Ongoing	
Dose	
Schedule	
Route	
Disease/Symptom	

INSERTED ON: 2011-10-10 14:54:02	BY: Demo Demo	LOCKED: NO
PRINTED ON: 2011-10-10 15:36:44	BY: Nadia Rubis	

PATIENT NUMBER: 666-01	VISIT:	ROW: 4	VISIT DATE: No date

FIELD	VALUE
Medication	
First Intake Date	
Last Intake Date	
First Intake Date Unknown	
Last Intake Date Unknown	
Last Intake Date Ongoing	
Dose	
Schedule	
Route	
Disease/Symptom	

INSERTED ON: 2011-10-10 14:54:06	BY: Demo Demo	LOCKED: NO
PRINTED ON: 2011-10-10 15:36:44	BY: Nadia Rubis	

PATIENT NUMBER: 666-01	VISIT:	ROW: 5	VISIT DATE: No date

FIELD	VALUE
Medication	
First Intake Date	
Last Intake Date	
First Intake Date Unknown	
Last Intake Date Unknown	
Last Intake Date Ongoing	
Dose	
Schedule	
Route	
Disease/Symptom	

INSERTED ON: 2011-10-10 14:54:09	BY: Demo Demo	LOCKED: NO
PRINTED ON: 2011-10-10 15:36:44	BY: Nadia Rubis	

ADVERSE EVENTS FORM DATA SUMMARY	
USER: Nadia Rubis	CENTER: -

PATIENT NUMBER: 666-01	VISIT:	ROW: 1	VISIT DATE: No date

FIELD	VALUE
Event	
Onset Date	
Stopped Date	
Onset Date Unknown	No
Stopped Date Unknown	No
Stopped Date Ongoing	No
Intensity	
Pattern	
Action	
Action	
Action	
Action	
Action	
Action	
Outcome	
Relation	
SAE	

INSERTED ON: 2011-10-10 14:54:17	BY: Demo Demo	LOCKED: NO
PRINTED ON: 2011-10-10 15:36:44	BY: Nadia Rubis	

PATIENT NUMBER: 666-01	VISIT:	ROW: 2	VISIT DATE: No date

FIELD	VALUE
Event	
Onset Date	
Stopped Date	
Onset Date Unknown	No
Stopped Date Unknown	No
Stopped Date Ongoing	No
Intensity	
Pattern	
Action	
Action	
Action	
Action	
Action	
Action	
Outcome	
Relation	
SAE	

INSERTED ON: 2011-10-10 14:54:20	BY: Demo Demo	LOCKED: NO
PRINTED ON: 2011-10-10 15:36:44	BY: Nadia Rubis	

PATIENT NUMBER: 666-01	VISIT:	ROW: 3	VISIT DATE: No date

FIELD	VALUE
Event	
Onset Date	
Stopped Date	
Onset Date Unknown	No
Stopped Date Unknown	No
Stopped Date Ongoing	No
Intensity	
Pattern	
Action	
Action	
Action	
Action	
Action	
Action	
Outcome	
Relation	
SAE	

INSERTED ON: 2011-10-10 14:54:24	BY: Demo Demo	LOCKED: NO
PRINTED ON: 2011-10-10 15:36:44	BY: Nadia Rubis	

PATIENT NUMBER: 666-01	VISIT:	ROW: 4	VISIT DATE: No date

FIELD	VALUE
Event	
Onset Date	
Stopped Date	
Onset Date Unknown	No
Stopped Date Unknown	No
Stopped Date Ongoing	No
Intensity	
Pattern	
Action	
Action	
Action	
Action	
Action	
Action	
Outcome	
Relation	
SAE	

INSERTED ON: 2011-10-10 14:54:29	BY: Demo Demo	LOCKED: NO
PRINTED ON: 2011-10-10 15:36:44	BY: Nadia Rubis	

PATIENT NUMBER: 666-01	VISIT:	ROW: 5	VISIT DATE: No date

FIELD	VALUE
Event	
Onset Date	
Stopped Date	
Onset Date Unknown	No
Stopped Date Unknown	No
Stopped Date Ongoing	No
Intensity	
Pattern	
Action	
Action	
Action	
Action	
Action	
Action	
Outcome	
Relation	
SAE	

INSERTED ON: 2011-10-10 14:54:32	BY: Demo Demo	LOCKED: NO
PRINTED ON: 2011-10-10 15:36:44	BY: Nadia Rubis	

INVESTIGATIONAL MEDICINAL PRODUCT PERMANENTLY DISCONTINUATION FORM DATA SUMMARY	
USER: Nadia Rubis	CENTER: -

PATIENT NUMBER: 666-01	VISIT:	ROW:	VISIT DATE: No date

FIELD	VALUE
Did the patient discontinue the IMP prematurely?	
Date of Last Intake	
Main reason for discontinuation	
Death Date	
Dialysis Date	
Other Reason Specify	
Who did initiate the discontinuation?	
Other Specify	

INSERTED ON: 2011-10-10 14:54:38	BY: Demo Demo	LOCKED: NO
PRINTED ON: 2011-10-10 15:36:44	BY: Nadia Rubis	

TRIAL END SUMMARY FORM DATA SUMMARY	
USER: Nadia Rubis	CENTER: -

PATIENT NUMBER: 666-01	VISIT:	ROW:	VISIT DATE: No date

FIELD	VALUE
Did the patient complete study period per protocol?	
Date of Discontinuation	
Main reason for discontinuation	
Death Date	
Dialysis Date	
Other Reason Specify	
Who did initiate the discontinuation?	
Other Specify	

INSERTED ON: 2011-10-10 14:54:44	BY: Demo Demo	LOCKED: NO
PRINTED ON: 2011-10-10 15:36:44	BY: Nadia Rubis	

Copyright © 2011 Mario Negri Institute. Biomedical Technologies Lab. All Rights Reserved.

CLINICAL INVESTIGATION PROTOCOL

Final Version – 30th May 2013

- CONFIDENTIAL -

PATIENT – REPORTED INFORMATION
ACCORDING TO THE
TYPE OF INSTRUMENT – 02

PARITY – 02

Quality of life and social costs in patients with non diabetic chronic kidney disease

PRINCIPAL INVESTIGATOR
Dr Giuseppe Remuzzi, Scientific Coordinator

IRCCS - Mario Negri Institute for Pharmacological Research
c/o Parco Scientifico Tecnologico Kilometro Rosso
Via Stezzano, 87, Bergamo, 24126, ITALY

PROTOCOL WRITTEN BY
Dr Matteo Conte , Dr Annalisa Perna

IRCCS - Mario Negri Institute for Pharmacological Research
Clinical Research Center for Rare Diseases Aldo e Cele Daccò
Villa Camozzi, Via Camozzi 3, Ranica, 24020, ITALY

ALL INFORMATION CONTAINED HEREIN IS CONFIDENTIAL

1. STUDY TITLE

PATIENT – REPORTED INFORMATION ACCORDING TO THE TYPE OF INSTRUMENT – 02
(PARITY – 02)
Quality of life and social costs in patients with non diabetic chronic kidney disease

2. SYNOPSIS

**TREATMENT**	Not applicable
**TITLE**	PATIENT – REPORTED INFORMATION ACCORDING TO THE TYPE OF INSTRUMENT – 02 (PARITY – 02) - Quality of life and social costs in patients with non diabetic chronic kidney disease
**OBJECTIVE**	To assess quality of life and societal costs in patients with non diabetic chronic kidney disease enrolled in the ongoing clinical trial: ALADIN 2 conducted at the Clinical Research Center *Aldo e Cele Daccò*, Mario IRCCS - Negri Institute, Ranica (BG), Italy
**CONDITION INTERVENTION**	Self-administered questionnaire
**PRIMARY VARIABLE(S)**	Quality of Life evaluation (QoL SF-36 V1^{TM} Questionnaire)<br>Social and labour cost evaluation by Health and Labour Questionnaire (SF-HLQ)
**SECONDARY VARIABLE(S)**	Serious and Non Serious Adverse events.
**STUDY TYPE**	Phase A: observational, cross-sectional study<br>Phase B: observational, prospective cohort study
**NUMBER OF CENTERS**	1<br>The study will be coordinated and monitored by the Clinical Research Center for Rare Diseases Aldo and Cele Daccò of the IRCCS - Mario Negri Institute for Pharmacological Research, Ranica, Bergamo. It will be conducted at Department of Renal Medicine of the Clinical Research Center Aldo and Cele Daccò and at Nephrology Units.
**NUMBER OF PATIENTS TO RECRUIT**	At least 80 patients randomized in the clinical trial ALADIN 2 carried out at the Clinical Research Center *Aldo e Cele Daccò*, Mario Negri Institute, Ranica (BG), Italy
**ENROLMENT PERIOD**	June 2013 – December 2014
**FOLLOW UP TERM**	Yearly for QoL SF-36 V1^{TM} (for the Phase B, prospective cohort study only)
**END OF FOLLOW UP TERM**	Closure of ALADIN 2 trial (for the Phase B, prospective cohort study only)

3. ETHICS

This study will be conducted in accordance with the ethical principles that have their origin in the Declaration of Helsinki and that are consistent with Good Clinical Practice and the applicable laws and regulations.

3.1 Independent ethics committee

This protocol will be submitted for approval to the local ethics committee. This investigation will be implemented in full accordance to the local laws for the protection of patients in biomedical studies and of patient data.

3.2 Data protection

All acting parties declare to keep the patient's data confidential. The patient will be informed about his/her right to consent or to deny the transmission of any study-related concerning his/her state of health, quality of life parameters and health and labour parameters, to his/her treating physician by the investigator before, during and after close-out of this study.

3.3 Patient information and consent

The patient will receive two separate documents: the Patient Information Letter and the Patient Consent Form. The documents will provide a summary of the quality of life (QoL) and socio-economical investigation, including its purpose in easily understandable local language. The investigator will be available to answer any question giving the patient sufficient reflection time. If the patient decides to take part in the investigation, he will date and sign the consent form and receive a copy of the document from the investigator. At any time included in the study will be able to withdraw their consent without any future liabilities.

4. DEFINITION OF RESPONSIBILITIES

4.1 Investigator's responsibilities

Investigator's responsibilities related to ALADIN 2 trial are reported in the final protocol and subsequent amendments of each study. As far as the responsibilities referred to the administration of the two questionnaires are concerned, the clinical investigator will:
a) be well acquainted with the investigation plan;
b) endeavour to ensure an adequate recruitment of subjects;
c) ensure that the subject has an adequate information to obtain informed consent;
d) obtain and document informed consent;
e) carry the primary responsibility for the accuracy, legibility and security of all documentation relevant to the investigation;
f) ensure that the basic data are kept for the appropriate time (as required by national laws and regulation);
g) be responsible for the supervision and assignment of duties to all responsible for the conduct and evaluation of the investigation in his/her department.

4.2 Monitor's responsibilities

The Clinical Monitor shall verify that Good Clinical Practice requirements are implemented. The study will be monitored by means of inspection of the following:
a) patients enrolment in relation to observed inclusion criteria;
b) signed and dated informed consent forms:
c) adherence to he study protocol and respect of privacy for the patient;
d) withdrawal of a patient and documentation of non conformity;
e) all clinical trial requirements and documentation to which this study is linked (ALADIN 2 clinical trial).

5. BACKGROUND

Autosomal Dominant Polycystic Kidney Disease (ADPKD), the most common hereditary cystic renal disease, has an incidence of 1 in 800 live births and account for 7-10% of patients on dialysis in developed countries (1). ADPKD shows genetic heterogeneity, with two main genes implicated, the PKD gene 1 (85%-90% of the cases) and the PKD gene 2 (10-15% of the cases) which encode for proteins policystin 1 and policystin 2 respectively (1,2). Clinically, ADPKD is characterized by renal and extra renal manifestations (1). In the kidneys, multiple cysts grow from distal and collecting tubular epithelial cells producing progressive renal enlargement with relatively initial stable renal functions (3). Thereafter, both tubular and secondary interstitial damage lead to faster renal loss and end-stage renal disease (ESRD) in approximately half of all patients affected in their fifth or sixth decade of life (2,4). More than 50% of the patients display hepatic cysts derived from cholangiocyte proliferation and fluid secretion (5). Pancreatic and intestinal cysts as well as increased risk of aortic aneurysms, heart-valve defects and sudden death due to rupture of intracerebral aneurysms are extra-renal manifestations, too (6). The precise molecular pathogenic mechanisms of ADPKD are not completely understood, but experimental models in renal tubular cells suggest that defect in policystin 1 or policystin 2 carry out to decrease intracellular calcium and increase cAMP levels changing the cell toward a proliferative and secretor phenotype (7,8,9). Cysts growth and enlargement are mainly consequence of epithelial cells proliferation and fluid chloride secretion through apical channels dependent of cAMP (10,11). Likewise, for hepatic cyst development, the proposed mechanisms are linked to abnormalities in epithelial cell proliferation and secretion in response to high intracellular cAMP concentrations secondary to policystin defects (12). Patients with ADPKD, at similar levels of proteinuria and blood pressure control, do not seem to benefit to the same extent of ACE inhibitor therapy and have faster decline in glomerular filtration rate (GFR) compared with other chronic kidney diseases (13). Thus, in ADPKD renoprotective interventions - in addition to achieving maximal reduction of arterial blood pressure and proteinuria, and limiting the effects of other potential disease progression promoters (such as dyslipidemia, chronic hyperglycemia, or smoking) (14,15) - should also be specifically aimed to correct the dysregulation of epithelial cell growth, fluid secretion, and extracellular matrix deposition that is characteristic of this disease. Up to now, no specific therapies for ADPKD are available, but drugs like somatostatin, rapamycin, and tolvaptan targeting to growth and chloride secretions pathways are now being testing worldwide in some clinical trials (16,17). Somatostatin is an endogenous cycled polypeptide presents in two active forms (14 and 18 amino acids) with endocrine, paracrine and autocrine actions (18). The effects of somatostatin are pleiotropic depending on the tissue and the type of receptors it binds to. Five subtypes of somatostatin receptors have been described (sst1-sst5), but on the renal tubular epithelial cells, sst1 and sst2 receptors predominate. Of interest, it has been reported that somatostatin inhibits the increase in intracellular cAMP and chloride secretion induced by arginine-vasopressin hormone (AVH) in rectal glands of sharks

(19,20). Octreotide, a synthetic analog of somatostatin with longer half-life and higher sst2 affinity than naive polypeptides (21), has been used with negligible side effects for long-term treatment of acromegaly (22) and some malignant tumors (23). Considering the inhibitory effect of somatostatin on cell proliferation and chloride secretion, we have performed some years ago a pilot prospective cross-over controlled study with long-acting somatostatin analog in patients with ADPKD and different degree of renal dysfunction (17). We found that in these patients, 6 month treatment with octreotide was safe, well tolerated, and slowed the time-dependent increase in total kidney volume to a significant extent compared to placebo. The net effect in kidney volume resulted from an action of the drug on cyst volume and on parenchyma volume (17). Moreover, more recent post-hoc analysis of the concomitant liver disease progression in the same ADPKD patients demonstrated a significant reduction in the total liver volume during octreotide treatment, not appreciably observed during placebo. The beneficial effect of octreotide has been then confirmed in rodent models of polycystic liver and kidney disease where the drug significantly reduced liver weight, cyst volume, hepatic fibrosis and mitotic indexes by reducing the cholangiocyte and serum cAMP levels (24). Moreover, in the untreated ADPKD patients enrolled in our study, computed tomography evaluation of disease progression showed that the ratio of faintly contrast-enhanced parenchyma volume – referred as intermediate parenchyma – over total parenchyma volume strongly correlated with basal GFR and GFR changes during the observation period (25). The good safety profile of octreotide and the slowing of renal growth demonstrated in our short-term clinical study did suggest the feasibility of a randomized trial in larger series of ADPKD patients with normal renal function or mild renal insufficiency to verify whether long-term somatostatin treatment may eventually provide effective renoprotection. This trial - the ALADIN study - is ongoing and the planned ADPKD patients have been enrolled. So far, no particular side effects have been reported. More important, preliminary interim analysis of data from patients who reached 1 year treatment, confirmed the beneficial effect of octreotide in slowing the growth of total kidney volume compared to placebo. There is urgent need for renoprotection in ADPKD patients, particularly for those with more advanced renal dysfunction, for whom so far no clinical trials have been designed. The findings of the safety and potential benefit of octreotide in few patients with severe renal insufficiency observed in our initial pilot study (17) and the encouraging preliminary long-term effect results of octreotide on kidney growth, make worth investigating the efficacy of a long-acting somatostatin (Octreotide LAR) in slowing or even halting the kidney enlargement and renal function decline in ADPKD patients with moderate/severe renal failure.

6. AIMS OF THE ALADIN 2 STUDY

Primary

The primary objective of this study in the short-term (1-year) is to compare the effect of long acting somatostatin analogue (Octreotide LAR) versus placebo on total kidney volume (TKV) change (delta TKV) as assessed by spiral computed tomography (spiral CT) scan. (see paragraph 6.1.1). While in the Long-term (3-years) the objective will be to compare the effect of long-acting somatostatin analogue (Octreotide LAR) vs. placebo on the rate of GFR decline as assessed by serial measurements of the iohexol plasma clearance.

Secondary
The secondary objectives of this study is to compare, among the two groups, absolute and relative changes in renal and liver volume parameters (at month 0, 12 and 36) as well as in functional and biochemical parameters by quarterly clinical visits during the 36 month follow-up and To assess the safety profile of the somatostatin analogue.

6.1 Description of ALADIN 2 study

ALADIN 2 is a randomized clinical trial coordinated by the IRCCS - Mario Negri Institute for Pharmacological Research at the Clinical Research Center for Rare Diseases, Ranica (Bergamo, Italy). Since October 2011 the above mentioned study has been recruiting patients with non diabetic chronic kidney disease.

7. PARITY 2 - STUDY DESIGN AND PATIENTS

7.1 Enrolment plan

The Phase A of the study has an observational, cross-sectional design. Patients are identified among the subjects referring to the Clinical Research Center for Rare Diseases *Aldo e Cele Daccò* of the IRCCS - Mario Negri Institute for Pharmacological Research fulfilling the inclusion/exclusion criteria. They will be asked to fill in the QoL Questionnaire (QoL SF-36 V1^{TM})[7] and the Health and Labour Questionnaire (SF-HLQ)[9]. Data will be collected in an ad-hoc form and in parallel clinical and demographic data on the above subjects will be collected in the trial Case Report Form (CRF) of the ALADIN 2 study. At least 80 all consecutive patients will be selected after randomization.
The Phase B of the study has an observational, prospective cohort design. Patients will be asked to fill in the questionnaires yearly (SF-36 V1^{TM}, only) and at the final study visit (both, SF-36 V1^{TM} and SF-HLQ). Data will be collected in an ad-hoc form and in parallel clinical and demographic data of the above subjects will be collected on the trial linked CRF of the ALADIN 2 study.
Eligible patients can be identified among the subjects attending the Day Hospital of the Clinical Research Center for Rare Diseases *Aldo e Cele Daccò* of the IRCCS - Mario Negri Institute, who are enrolled in the above mentioned ongoing clinical trial in patients with non diabetic chronic kidney disease.

7.2 Study population

7.2.1 Number of patients

Due to the exploratory nature of the study a formal sample size assessment is not foreseen. However at least 80 patients with non diabetic chronic kidney disease will be adequate to provide a reliable measure of the eight QoL domains (1. physical functioning, 2. role-physical, 3. bodily pain, 4. general health, 5. vitality, 6. social functioning, 7. role-emotional, 8. mental health). The above sample size has been considered adequate also to provide a reliable measure of the societal and labour costs in the above kind of participants. A difference of 5 points or more is considered as clinically significant.

7.2.2 Inclusion criteria

The following inclusion criteria will be considered:
a) patients with non diabetic chronic kidney disease;

b) enrolled in ALADIN 2 clinical trial at the Clinical Research Center for Rare Diseases *Aldo e Cele Daccò* of the IRCCS - Mario Negri Institute for Pharmacological Research;
c) legal capacity to give informed consent;
d) written informed consent;
e) inability to compile the questionnaires for different reasons from those listed in the exclusion criteria (e.g. blindness). In this case the subject will be assisted by accompanying person.
f) Age > 18 years
g) Clinical and ultrasound diagnosis of ADPKD
h) Estimated GFR between 15 and 40 ml/min/1.73m2 (by the MDRD 4 variable equation)

7.2.3 Exclusion criteria

The following exclusion criteria will be considered:
a) patients with diabetic chronic kidney disease;
b) legal incapacity and/or the circumstances rendering the patient unable to understand the nature and the scope of the study.
c) 24-h Urinary protein excretion rate >3g (suggestive of a concomitant glomerular disease that could benefit of specific therapy)
d) Symptomatic urinary tract lithiasis or obstruction
e) Uncontrolled diabetes mellitus (HbA1c >8%) or hypertension (systolic/diastolic BP >180/110 mmHg)
f) Current urinary tract infection
g) Symptomatic biliary tract lithiasis
h) Active cancer
i) Psychiatric disorders or any condition that might prevent full comprehension of the purposes and risks of the study
l) Pregnancy, lactation or child bearing potential and ineffective contraception (estrogen therapy in post menopausal women should not be stopped)

7.3 Setting

Day Hospital of the Clinical Research Center for Rare Diseases *Aldo e Cele Daccò* of the IRCCS - Mario Negri Institute for Pharmacological Research.

7.4 Study Type

The Phase A of the study is an observational cross-sectional study. The phase B has an observational, prospective cohort design.
Patient satisfying the inclusion/exclusion criteria will be provided with two questionnaires. Patient will be asked to fill in the informed consent and then the questionnaires. They will be assisted, if required, by an accompanying persons. Questionnaires will be rendered anonymous for data processing.
The recruitment is expected to start in June 2013 and to end December 2014 for Phase A. Yearly, for phase B patients will be asked to fill in the questionnaire SF-36 V1^{TM} in order to evaluate changes in QoL; at clinical study closure, patients will be asked to fill in both questionnaires SF-36 V1^{TM} and SF-HLQ to assess changes in QoL and societal costs.
In parallel to data-gathering by two previously mentioned questionnaires, demographic and clinical data will be collected in the CRF of the linked clinical trial.

7.5 Quality of life and societal cost evaluation

The primary aim of this study is to asses the QoL and the societal costs as described in the following.

7.5.1 Quality of life

The QoL (including physical functioning, role-physical, bodily pain, general health, vitality, social functioning, role-emotional and mental health) of all patients will be evaluate using the Version 1 of SF-36 (QoL SF-36 V1™ Questionnaire) adjusted and validated in Italy[7] (see Enclosure A). For the Phase B of the study quantity and quality of life can be quantified by the quality-adjusted life year (QALY)[15,16] indicator to be used to assess the impact of healthcare intervention on an individual patient.

7.5.2 Social costs

Social costs include production losses due to the absence from work, reduced productivity, and reduced unpaid labour participation. Production losses of paid and unpaid labour will be assessed by means of the Short-Form-Health and Labour Questionnaire (SF-HLQ), of which existing translations are available for Italy[11] (see Enclosure B). The SF-HLQ Questionnaires allows to estimate indirect non-medical costs of productivity losses caused by absence from work due to health problems. Indirect costs are used in pharmaco-economic research to perform an economic evaluation from societal perspective[9].

7.5.3 Currency

Euro will be considered as currency for the evaluation of social costs.

7.6 Sample size and statistical analysis

7.6.1 Sample size

See paragraph 7.2.1

7.6.2 Statistical analysis

As distributions of QoL scores are skewed[11], the nonparametric Wilcoxon rank sum test will be applied. Scores will be expressed as a median and interquartile range. To compare the costs by clinical category, a multiple linear regression model will be used. Explorative analyses in pre-defined subgroups of patients (e.g. by gender) will be performed. Study results will be reported according to suggested guidelines[17]. A *P value* less than 0.05 (two tailed) will be considered to indicate statistical significance. All analyses will be carried out using SAS 9.1 (SAS Institute Inc., Cary, NC) and Stata 12 (StataCorp LP, College Station, Texas).

8. DATA COLLECTION

The scores of QoL Questionnaire SF36 V1™ and indirect costs (missed days work, etc.) will be quantified by means of the Health and Labour Questionnaire and recorded on a paper data form and coded on a Excel file (Microsoft Office Excel 2003).

9. STUDY START

Expected Phase A and Phase B study are foreseen to start in March 2013.

10. STUDY COMPLETATION

The study will be completed depending on study closure of ALADIN 2 trial.

11. ACKNOWLEDGEMENTS

The authors are grateful to Professor Leona van Roijen (Erasmus University Rotterdam, The Netherlands), Professor Maarten J. Postma (University of Groningen, The Netherlands) and colleagues for permission to us the SF-HLQ instrument.

12. REFERENCES (FROM ALADIN 2'S PROTOCOL)

1. P Wilson. Mechanisms of disease: Polycystic Kidney Disease. N Engl J Med 2004; 350:151-64.
2. M Simons, G Walz. Polycystic kidney disease: Cell division without a c(l)ue?. Kidney Int 2006; 70:854–864.
3. Grantham JJ, Chapman AB, Torres VE. Volume progression in autosomal dominant polycystickidney disease: the major factor determining clinical outcomes. Clin J Am Soc Nephrol 2006; 1:148-157.
4. E Meijer, P Jong, D Peters, R Gansevoort. Better understanding of ADPKD results in potential newtreatment options: ready for the cure. J Nephrol 2008; 21: 133-138.
5. Alvaro D, Onori P, Alpini G, Franchitto A, Jefferson D, Torrice A et al. Morphological and functional features of hepatic cyst epithelium in autosomal dominant polycystic kidney disease. AmJ Pathol. 2008 Feb;172(2):321-32.
6. Ul Haque, Moatasim A. Adult polycystic kidney disease: a disorder of connective tissue? Int J ClinExp Pathol 2008; 1(1):84-90.
7. E Meijer, P Jong, D Peters, R Gansevoort. Better understanding of ADPKD results in potential new treatment options: ready for the cure. J Nephrol 2008; 21: 133-138.
8. T Yamaguchi, D Wallace, B Magenheimer, S Hempson, J Grantham, J Calvet. Calcium restriction allows cAMP activation of the B-Raf/ERK pathway, switching cells to a cAMP-dependent growthstimulated phenotype. J Biol Chem 2004; 279: 40419–40430.
9. T Yamaguchi, S Hempson, G Reif, A Hedge, D Wallace. Calcium restores a normal proliferation phenotype in human polycystic kidney disease epithelial cells. J Am Soc Nephrol 2006; 17: 178–187.
10. M Albaqumi, S Srivastava, Z Li, O Zhdnova, H Wulff. KCa3.1 potassium channels are critical for cAMP-dependent chloride secretion and cyst growth in ADPKD. Kidney Int (advance online publication, June 11, 2008).
11. B Yang, N Sonawane, D Zhao, S Somlo, A. Verkman. Small-molecule CFTR inhibitors slow cyst growth in polycystic kidney disease. J Am Soc Nephrol 2008; 19: 1300–1310.
12. Francis H, Glaser S, Ueno Y, Lesage G, Marucci L, Benedetti A et al. cAMP stimulates the secretory and proliferative capacity of the rat intrahepatic biliary epithelium through changes in the PKA/Src/MEK/ERK1/2 pathway. J Hepatol. 2004 Oct;41(4):528-37.
13. Van Dijk MA, Breuning MH, Duiser R, van Es LA, Westendorp RG. No effect of enalapril on progression in autosomal dominant polycystic kidney disease. Nephrol Dial Transplant 2003; 18:2314-2320.
14. Ecder T et al: Effect of antihypertensive therapy on renal function and urinary albumin excretion in hypertensive patients with autosomal dominant polycystic kidney disease. Am J Kidney Dis 2000; 35: 427-432.
15. Torres VE. Hypertension, proteinuria, and progression of autosomal dominant polycystic kidney disease. Am J Kidney Dis 2000; 35: 547-550.
16. A. Chapman. Autosomal Dominant Polycystic Kidney Disease: Time for a Change? J Am Soc Nephrol 2007; 18: 1399-1407.
17. Ruggenenti P, Remuzzi A, Ondei P, Fasolini G, Antiga L, Ene-Iordache B, Remuzzi G, Epstein F. Safety and efficacy of long-acting somatostatin treatment on autosomal dominant polycystic kidney disease. Kidney Int 2005; 68: 206–216.
18. M. Pawlikowski, G. Melen-Mucha. Perspective of new potential therapeutic applications of somatostatin analogs. Neuroendocrinology Letters 2003; 24: 21-27.

19. P Silva et al: Mode of action of somatostatin to inhibit secretion by shark rectal gland. Am J Physiol 1985 249:R329–R334.
20. P Silva et al: Somatostatin inhibits CNP-induced stimulation of shark rectal gland. Bull Mt Desert Island Biol Lab 1991; 40: 25–29.
21. G Tulipano and S Schulz. Novel insights in somatostatin receptor physiology. Eur J Endocrinol 2007; 156: S3–S11.
22. J Ayuk, SE Stewart, PM Stewart, MC Sheppard. Long-term safety and efficacy of depot long-acting somatostatin analogs for the treatment of acromegaly. J Clin Endocrinol Metab 2002; 87: 4142– 4146.
23. C Susini, L Buscail. Rationale for the use of somatostatin analogs as antitumor agents. Ann Oncol 2006; 17: 1733–1742.
24. Masyuk T, Masyuk A, Torres V, Harris P, Larusso N. Octreotide inhibits hepatic cystogenesis in a rodent model of polycystic liver disease by reducing cholangiocyte adenosine 3',5'- cyclic monophosphate. Gastroenterolgy 2006; 123: 1104-1116.
25. Antiga L, Piccinelli M, Fasolini G, Ene-Iordache B, Ondei P, Bruno S, Remuzzi G, Remuzzi Computed tomography evaluation of ADPKD progression: a progress report. Clin J Am Soc Nephrol 2006; 1: 754-760.
26. Feelders R, Hofland L, van Aken M, Neggers S, Lamberts S, de Herder W and van der Lely AJ. Drugs 2009, 69 (16):2207-26.
27. Caroli A, Antiga L, Cafaro M, Fasolini G, Remuzzi A, Remuzzi G and Ruggenenti P .Clin J Am Soc Nephrol. 2010, 5:783-9.
28. O'Brien P, Fleming J. A multiple testing procedure for clinical trials. Biometrics 1979; 35:549-556.

13. ENCLOSURE A

ALADIN 2 STUDY SYNOPSIS

**TREATMENT**	
**TITLE**	A Prospective, Randomized, Double-Blind, Placebo Controlled Clinical Trial To Assess The Effects Of Long-Acting Somatostatin (Octreotide Lar) Therapy On Disease Progression In Patients With Autosomal Dominant Polycystic Kidney Disease And Moderate To Severe Renal Insufficiency (Aladin 2 Study)
**OBJECTIVE**	The general aim of the trial is to assess the efficacy of one year treatment with long-acting somatostatin analogue (Octreotide LAR) compared with placebo in slowing kidney and liver growth rate in patients with ADPKD and moderate/severe renal insufficiency (estimated GFR by MDRD 4 variables: 15-40 ml/min/1.73m2) and to assess whether and to which extent this translates into slower renal function decline over 3-year follow-up.
**CONDITION INTERVENTION**	After four weeks wash-out period from previous ACE inhibitors and AII receptor antagonist, eligible patients will be randomized to two treatment arms:<br>1. Telmisartan (one week 40 mg daily, followed by fifteen weeks treatment period with 80 mg daily);<br>2. Losartan (one week 50 mg daily, followed by fifteen weeks treatment period with 100 mg daily);<br>At the end of the first treatment period with telmisartan or losartan, each patient will crossover to the other treatment.<br>After the second treatment period, there will be a four week recovery period.
**PRIMARY VARIABLE(S)**	*Primary*<br>Short-term (1-year)<br>To compare the effect of long acting somatostatin analogue (Octreotide LAR) versus placebo on total kidney volume (TKV) change (delta TKV) as assessed by spiral computed tomography (spiral CT) scan.<br>Long-term (3-years)<br>To compare the effect of long-acting somatostatin analogue (Octreotide LAR) vs. placebo on the rate of GFR decline as assessed by serial measurements of the iohexol plasma clearance.
**SECONDARY VARIABLE(S)**	*Secondary*<br>To compare, among the two groups, absolute and relative changes in renal and liver volume parameters (at month 0, 12 and 36) as well as in functional and biochemical parameters by quarterly clinical visits during the 36 month follow-up.
**STUDY TYPE**	A prospective, randomized, open label blinded end point (PROBE).
**NUMBER OF PATIENTS TO RECRUIT**	Due to the exploratory nature of the study a formal sample size assessment is not foreseen. However at least 80 patients with non diabetic chronic kidney disease will be adequate to provide a reliable measure of the eight QoL domains (1. physical functioning, 2. role-physical, 3. bodily pain, 4. general health, 5. vitality, 6. social functioning, 7. role-emotional, 8. mental health). The above sample size has been considered adequate also to provide a reliable measure of the societal and labour costs in non diabetic chronic kidney disease. A difference of 5 points or more is considered as clinically significant.
**STUDY PERIOD**	32 weeks

14. ENCLOSURE B

QUESTIONARIO SULLO STATO DI SALUTE SF-36

Data consegna I_I_I I_I_I I_I_I N° codice paziente I_I_I_I_I_I

ISTRUZIONI: Questo questionario intende valutare cosa Lei pensa della Sua salute. Le informazioni raccolte permetteranno di essere sempre aggiornati su come si sente e su come riesce a svolgere le Sue attività consuete.

Risponda a ciascuna domanda del questionario indicando la Sua risposta come mostrato di volta in volta. Se non si sente certo della risposta, effettui la scelta che comunque Le sembra migliore.

1. In generale, direbbe che la Sua salute è:

(Indichi un numero)

Eccellente .... ……………1
Molto buona..2
Buona..3
Passabile 4
Scadente 5

2. Rispetto ad un anno fa, come giudicherebbe, ora, la Sua salute in generale?

(Indichi un numero)

Decisamente migliore adesso rispetto ad un anno fa 1
Un po' migliore adesso rispetto ad un anno fa 2
Più o meno uguale rispetto ad un anno fa..3
Un po' peggiore adesso rispetto ad un anno fa 4
Decisamente peggiore adesso rispetto ad un anno fa 5

Copyright © New England Medical Center Hospitals, Inc., 1992
All rights reserved.
(IQOLA SF-36 Italian version 1.6)

3. Le seguenti domande riguardano alcune attività che potrebbe svolgere nel corso di una qualsiasi giornata. La Sua salute La limita attualmente nello svolgimento di queste attività?

(Indichi per ogni domanda il numero 1, 2, o 3)

	**SI, mi limita parecchio**	**SI, mi limita parzialmente**	**NO, non mi limita per nulla**
a. **Attività fisicamente impegnative**, come correre, sollevare oggetti pesanti, praticare sport faticosi	1	2	3
b. **Attività di moderato impegno fisico**, come spostare un tavolo, usare l'aspirapolvere, giocare a bocce o fare un giretto in bicicletta	1	2	3
c. Sollevare o portare le borse della spesa	1	2	3
d. Salire **qualche** piano di scale	1	2	3
e. Salire **un** piano di scale	1	2	3
f. Piegarsi, inginocchiarsi o chinarsi	1	2	3
g. Camminare **per un chilometro**	1	2	3
h. Camminare **per qualche centinaia di metri**	1	2	3
i. Camminare **per circa cento metri**	1	2	3
l. Fare il bagno o vestirsi da soli	1	2	3

4. Nelle ultime 4 settimane, ha riscontrato i seguenti problemi sul lavoro o nelle altre attività quotidiane, a causa della Sua salute fisica?

Risponda SI o NO a ciascuna domanda

(Indichi per ogni domanda il numero 1 o 2)

	SI	NO
a. Ha ridotto **il tempo** dedicato al lavoro o ad altre attività	1	2
b. Ha **reso** meno di quanto avrebbe voluto	1	2
c. Ha dovuto limitare alcuni **tipi** di lavoro o di altre attività	1	2
d. Ha avuto **difficoltà** nell'eseguire il lavoro o altre attività (ad esempio, ha fatto più fatica)	1	2

Copyright © New England Medical Center Hospitals, Inc., 1992
All rights reserved.
(IQOLA SF-36 Italian version 1.6)

5. Nelle ultime 4 settimane, ha riscontrato i seguenti problemi sul lavoro o nelle altre attività, a causa del Suo stato emotivo (quale il sentirsi depresso o ansioso)?

Risponda SI o NO a ciascuna domanda

(Indichi per ogni domanda il numero 1 o 2)

	SI	NO
a. Ha ridotto **il tempo** dedicato al lavoro o ad altre attività	1	2
b. Ha **reso** meno di quanto avrebbe voluto	1	2
c. Ha avuto un calo di **concentrazione** sul lavoro o in altre attività	1	2

6. Nelle ultime 4 settimane, in che misura la Sua salute fisica o il Suoi stato emotivo hanno interferito con le normali attività sociali con la famiglia, gli amici, i vicini di casa, i gruppi di cui fa parte?

(Indichi un numero)

Per nulla...1
Leggermente..2
Un po' 3
Molto...4
Moltissimo…..5

7. Quanto dolore fisico ha provato nelle ultime 4 settimane?

(Indichi un numero)

Nessuno 1
Molto lieve.. 2
Lieve 3
Moderato 4
Forte 5
Molto forte 6

Copyright © New England Medical Center Hospitals, Inc., 1992
All rights reserved.
(IQOLA SF-36 Italian version 1.6)

8. Nelle ultime 4 settimane, in che misura il dolore L'ha ostacolata nel lavoro che svolge abitualmente (sia in casa sia fuori casa)?

(Indichi un numero)

Per nulla 1
Molto poco...2
Un po'…..3
Molto…..4
Moltissimo…...5

9. Le seguenti domande si riferiscono a come si è sentito nelle ultime 4 settimane. Risponda a ciascuna domanda scegliendo la risposta che più si avvicina al Suo caso. Per quanto tempo nelle ultime 4 settimane si è sentito...

(Indichi un numero per ogni domanda)

	**Sempre**	**Quasi sempre**	**Molto tempo**	**Una parte del tempo**	**Quasi mai**	**Mai**
a. vivace brillante?	1	2	3	4	5	6
b. molto agitato?	1	2	3	4	5	6
c. così giù di morale che niente avrebbe potuto tirarLa su?	1	2	3	4	5	6
d. calmo e sereno?	1	2	3	4	5	6
e. pieno di energia?	1	2	3	4	5	6
f. scoraggiato e triste?	1	2	3	4	5	6
g. sfinito?	1	2	3	4	5	6
h. felice?	1	2	3	4	5	6
i. stanco?	1	2	3	4	5	6

Copyright © New England Medical Center Hospitals, Inc., 1992
All rights reserved.
(IQOLA SF-36 Italian version 1.6)

10. Nelle ultime 4 settimane, per quanto tempo la Sua salute fisica o il Suo stato emotivo hanno interferito nelle Sue attività sociali, in famiglia, con gli amici?

(Indichi un numero)

Sempre 1
Quasi sempre 2
Una parte del tempo..3
Quasi mai..4
Mai...5

11. Scelga la risposta che meglio descrive quanto siano **VERE** o **FALSE** le seguenti affermazioni.

(Indichi un numero per ogni affermazione)

	**Certamente vero**	**In gran parte vero**	**Non so**	**In gran parte falso**	**Certamente falso**
a. Mi pare di ammalarmi un po' più facilmente degli altri	1	2	3	4	5
b. La mia salute è come quella degli altri	1	2	3	4	5
c. Mi aspetto che la mia salute andrà peggiorando	1	2	3	4	5
d. Godo di ottima salute	1	2	3	4	5

Copyright © New England Medical Center Hospitals, Inc., 1992
All rights reserved.
(IQOLA SF-36 Italian version 1.6)

15. ENCLOSURE C

QUESTIONARIO SF-HLQ

SF-HLQ

Le seguenti domande riguardano gli effetti dei problemi di salute sul lavoro retribuito e su quello non retribuito (ad esempio il lavoro domestico). Tutte le domande fanno riferimento alle **ultime due settimane.**

Con “problemi di salute” ci si riferisce tanto ai problemi fisici quanto a quelli emotivi.

1. Hai attualmente un lavoro retribuito?

   ❑ No, *(continua per favore con la domanda 9)*
   ❑ Si’;
   a. Quante ore alla settimana lavori? .... ore alle settimana.

   b. Suddivide in quanti giorni? .... giorni.

   c. Che lavoro fai? ....

2. a. Nelle ultime due settimane, sei stato/a completamente incapace di svolgere il tuo lavoro retribuito, in seguito a problemi di salute?

   ❑ No
   ❑ Si’, non sono stato in grado di lavorare per ...... giorni.

   b. Sei stato/a completamente incapace di svolgere il tuo lavoro retribuito, in seguito a problemi di salute, per un periodo **superiore** alle ultime due settimane?

   ❑ No
   ❑ Si’, sono malato/a da .... *(continua per favore con la domanda 8)*

A volte i problemi di salute non consentono al lavoratore di presentarsi al lavoro. E’ inoltre possibile che il lavoratore vada al lavoro ma, sempre a causa dei problemi di salute, non sia in grado

di svolgere le sue funzioni con la stessa abituale efficienza. Le domande che vanno dalla n. 3 alla n. 7 si riferiscono a questa seconda situazione.

3. Sei stato/a ostacolato/a da problemi di salute nelle ultime due settimane nello svolgere il tuo lavoro retribuito?

   ❑ No, in nessun modo *(vai alla domanda 8)*
   ❑ Si', mediamente
   ❑ Si', molto

4. Nelle ultime due settimane, quanti giorni sei andato/a al lavoro malgrado avessi problemi di salute?

   .... giorni *(NOTA: **Non** considerare i giorni in cui hai riportato essere stato/a malato/a)*

5. Per cortesia, indica il livello di **efficienza** del tuo lavoro nei giorni in cui hai lavorato benche' avessi problemi di salute.
   (1 indica che a mala pena sei stato/a in grado di lavorare, mentre 10 indica che il tuo lavoro non ne ha subito conseguenze)

1	2	3	4	5	6	7	8	9	10
❑	❑	❑	❑	❑	❑	❑	❑	❑	❑

massima inefficienza — efficiente come al solito

6. Nella seguente domanda troverai una serie di dichiarazioni che possono riferirsi a persone con problemi di salute in relazione alla loro attuale situazione lavorativa. Per cortesia indica, per ogni dichiarazione che e' menzionata, quanto spesso si puo' applicare a te, nelle ultime due settimane.

Sono andato/a al lavoro, ma come conseguenza di problemi di salute ….

	quasi mai	qualche volta	spesso	quasi sempre
.. ho avuto problemi a concentrarmi	❑	❑	❑	❑
.. ho lavorato piu' lentamente	❑	❑	❑	❑
.. ho dovuto isolarmi dagli altri	❑	❑	❑	❑
.. ho avuto piu' difficolta' a prendere decisioni	❑	❑	❑	❑
.. ho dovuto rinviare parte del mio lavoro	❑	❑	❑	❑
.. altri hanno dovuto fare parte del mio lavoro	❑	❑	❑	❑

.. ho avuto altri problemi, (per cortesia indicali) ………………………………………………….

7. Quante ore straordinarie dovrai fare per recuperare il lavoro che non sei stato/a in grado di completare nelle tue normali ore lavorative, in seguito a problemi di salute, nelle ultime due settimane?

   ……………… ore *(NOTA: Non conteggiare i giorni in cui risulti in malattia.)*

8. Vorremmo sapere qual'e' il tuo stipendio netto (quello derivante dal tuo lavoro retribuito). *(NOTA: questa domanda riguarda le tue entrate, non includere quelle del tuo partner - se ne hai uno.)*

   ❑ € ……………………………… alla settimana

   ❑ € ……………………………… per 4 settimane

   ❑ € ……………………………… al mese

   ❑ € ……………………………… all'anno

   ❑ Non so a quanto ammonano le mie entrate, o preferisco non dirlo

   Ora vai alla domanda 10

9. Non hai un lavoro retribuito. Quale delle seguenti situazioni si applica meglio a te stesso/a?

   ❑ Ho il compito quotidiano di svolgere i lavori domestici.
   ❑ Ricevo una pensione, o sono entrato/a in pensione anticipata.
   ❑ Sono ancora uno/a studente/essa.

- ❑ Non sono in grado di svolgere lavoro retribuito in seguito ai problemi di salute da ……/……/…….. Se prima di questa data avevi un lavoro retribuito, quante ore alla settimana lavoravi? ……………………ore alla settimana.
- ❑ Non ho lavoro retribuito per altre ragioni (ad esempio disoccupazione volontaria, volontariato, ecc.)

10. Quanti soldi hai ricevuto, il mese scorso, dai programmi governativi di assistenza sociale/pensione/indennita’ di invalidita’?

- ❑ € 0
- ❑ € 1 - € 200
- ❑ € 201 - € 500
- ❑ € 501 - € 1000
- ❑ > € 1001

11. Quanti soldi hai ricevuto da familiari o amici il mese scorso?

- ❑ € 0
- ❑ € 1 - € 200
- ❑ € 201 - € 500
- ❑ € 501 - € 1000
- ❑ > € 1001

12. Hai svolto le seguenti attivita’ nelle ultime due settimane?

a. Lavori domestici (ad esempio, cucinare, pulire, fare il bucato)

- ❑ Svolti, non sono stato/a ostacolato/a dai problemi di salute
- ❑ Svolti, ma sono stato/a ostacolato/a da problemi di salute
- ❑ Non svolti, a causa dei problemi di salute
- ❑ Non svolti, per altre ragioni

b. Spese (ad esempio, spesa alimentare, altro shopping, andare in banca o all'ufficio postale)

- ❑ Fatte, non sono stato/a ostacolato/a dai problemi di salute
- ❑ Fatte, ma sono stato/a ostacolato/a da problemi di salute
- ❑ Non fatte, a causa dei problemi di salute
- ❑ Non fatte, per altre ragioni

c. Lavori occasionali e altri lavori domestici (ad esempio, riparazioni alla casa, giardinaggio, lavori di manutenzione della macchina)

- ❑ Svolti, non sono stato/a ostacolato/a dai problemi di salute
- ❑ Svolti, ma sono stato/a ostacolato/a da problemi di salute
- ❑ Non svolti, a causa dei problemi di salute
- ❑ Non svolti, per altre ragioni

d. Fatto cose per o con i tuoi figli (ad esempio, prendersi cura di loro, giocare, portali a scuola, aiutarli nei compiti)

- ❑ Svolte, non sono stato/a ostacolato/a dai problemi di salute
- ❑ Svolte, ma sono stato/a ostacolato/a da problemi di salute
- ❑ Non svolte, a causa dei problemi di salute
- ❑ Non svolte, per altre ragioni
- ❑ Non applicabile

13. Altre persone hanno dovuto occuparsi, nelle ultime due settimane, dei lavori di casa che normalmente tu svolgi, a causa dei tuoi problemi di salute?

- ❑ No
- ❑ Si', (puoi segnare anche piu' di una risposta):
  - ❑ Familiari, per ....ore
  - ❑ Altre persone non retribuite, per .... ore
  - ❑ Altro aiuto retribuito, per .... ore

Questa e' la fine del questionario. Grazie molte per la tua collaborazione.

Nello spazio sottostante puoi annotare ogni suggerimento o commento tu voglia fare a proposito del questionario:
....
....
....
....

16. ENCLOSURE D

Lettera per il paziente: descrizione dello studio PARITY-02

Ranica, June 2013

Gentile Signore/a,

Con l'obiettivo di ottenere informazioni sempre più esaurienti riferite al Suo stato di salute, riteniamo di estrema utilità chiederLe di compilare i due questionari che accompagnano questa lettera. Il progetto prende il nome di PARITY-02 ed ha come scopo quello di valutare la qualità della vita e i costi sociali nei pazienti colpiti da malattie renali di origine non diabetica.

Ciò rientra nell'ambito di un'iniziativa della nostra struttura di Day Hospital, che pone l'accento sulla rilevazione di alcune informazioni specificate di seguito.
In particolare, il primo questionario che Le verrà consegnato, denominato Short Form Quality of Life Questionnaire (SF36, Versione 1), pone domande su cosa Lei pensa della Sua salute e su come riesce a svolgere le Sue attività quotidiane, sul dolore, su alcuni aspetti di natura psicologica e relazionale.

Il secondo questionario, Short Form Health and Quality Questionnaire (SF-HLQ), riguarda gli effetti dei problemi di salute sul Suo lavoro, sulle Sue condizioni economiche e sulla capacità a svolgere i lavori domestici.

La compilazione dei due questionari Le verrà richiesta con cadenza annuale, in base alla durata dello studio clinico, a cui sta partecipando. Se sarà necessario, può farsi aiutare nella compilazione da persona che l'accompagna.

E' molto importante sottolineare che i questionari Le saranno sottoposti, solo se Lei lo vorrà e che ciò avverrà in forma del tutto anonima.

Siamo a Sua disposizione per eventuali richieste o chiarimenti e La ringraziamo per l'attenzione dedicata.

Cordiali Saluti.

Dr. --------------- ed il gruppo degli operatori del Day Hospital

20. ENCLOSURE E

Informativa al trattamento dei dati personali e modulo di consenso PARITY-02

Studio PARITY-02 – Informativa al trattamento dei dati personali e modulo di consenso

Studio PARITY-02

Qualità della vita e valutazione dei costi sociali nei pazienti con malattie renali croniche non di origine diabetica

Proposta di partecipazione ad uno studio osservazionale volto a valutare la Qualità della Vita e i Costi Sociali in pazienti affetti da malattie renali di origine non diabetica attraverso la compilazione di due questionari:

- Short Form Quality of Life Questionnaire SF 36 (Versione 1)
- Short Form Health and Labour Questionnaire SF-HLQ.

Informativa sul trattamento dei dati personali

Titolari del trattamento e relative finalità

Il Centro di Ricerche Cliniche per le Malattie Rare Aldo e Cele Daccò dell' IRCCS - Istituto di Ricerche Farmacologiche Mario Negri è promotore dello studio che Le è stato descritto, in accordo alle responsabilità previste dalle norme di Buona Pratica Clinica (d.l. 211/2003). Tratterà i Suoi dati personali (stato di salute, condizione economica e lavorativa) e quelli demografici (data di nascita, sesso, peso, statura) saranno raccolti e trattati esclusivamente in funzione delle finalità dello studio. Il rifiuto di conferirli non consentirà la Sua partecipazione allo studio.

Tutti i dati saranno resi anonimi, registrati, elaborati e conservati dall' IRCCS - Istituto di Ricerche Farmacologiche Mario Negri. Soltanto i soggetti autorizzati potranno collegare il numero di codice, che le sarà attribuito, al Suo nominativo. Anche i dati clinici che La riguardano, contenuti nella Sua documentazione clinica originale potranno essere utilizzati dal personale preposto allo studio, dal Comitato Etico e dalle Autorità Sanitarie, con modalità tali da garantire l'assoluta riservatezza.

Modalità del trattamento

I risultati di analisi statistiche dei dati raccolti, mediante strumenti elettronici, saranno diffusi in forma rigorosamente anonima, ad esempio attraverso pubblicazioni scientifiche e convegni scientifici**.**

Esercizio dei diritti

Lei potrà esercitare in ogni momento i diritti, di cui all'art. 7 del Codice Civile in materia di protezione dei dati personali (es. accedervi, integrarli, aggiornarli, rettificarli, opporsi al loro trattamento per motivi legittimi, ecc.) rivolgendosi direttamente al personale dell' IRCCS - Istituto di Ricerche Farmacologiche

Mario Negri. Lei potrà comunque interrompere in ogni momento, e senza fornire alcuna giustificazione, la Sua partecipazione allo studio.

Consenso

Io sottoscritto/a ____

Nato/a a: ______________________________ (Prov.______), il: ___/___/___

Presa visione dell'informativa, esprimo il consenso, ai sensi del D.Lgs 196/03 in materia di tutela dei dati personali, al trattamento dei miei dati ed alla consultazione della mia cartella clinica per gli scopi della ricerca nei limiti e con le modalità indicate nell'informativa fornitami con il presente documento.
Dichiaro inoltre che la natura dello studio in oggetto mi è stata completamente spiegata.
Ho avuto la possibilità di porre domande, valutare le risposte ricevute e ho compreso pienamente tutte le informazioni fornitami sugli scopi, i metodi, i possibili benefici e i potenziali rischi dello studio. La mia partecipazione è volontaria e posso ritirarmi dallo studio in qualsiasi momento; questo non comprometterà le attenzioni e le cure che riceverò in futuro dai miei medici curanti.

Pertanto volontariamente e liberamente

Acconsento   Non acconsento

A partecipare allo studio e all'uso dei dati personali come indicato nella nota informativa.

Firma del paziente____________________________Data_______________

Firma del Testimone imparziale____________________Data_______________
(se applicabile)

Confermo di aver spiegato la natura di questo studio al paziente.

Firma del medico____________________________Data_______________

Qualora accettasse di partecipare a questo studio, riceverà una copia o un originale di questo modulo di consenso informato, firmato e datato per la Sua documentazione privata.

TABLE TEMPLATES

TABLE 1.1

ANALYSIS POPULATIONS

	Octreotide-LAR	Placebo	Total
Full Analysis Set	xx (xx%)	xx (xx%)	xx (xx%)
Overall	xx (xx%)	xx (xx%)	xx (xx%)
Peripheral Site 1	xx (xx%)	xx (xx%)	xx (xx%)
•	xx (xx%)	xx (xx%)	xx (xx%)
•	xx (xx%)	xx (xx%)	xx (xx%)
•	xx (xx%)	xx (xx%)	xx (xx%)
Peripheral Site n	xx (xx%)	xx (xx%)	xx (xx%)
Per Protocol Set	xx (xx%)	xx (xx%)	xx (xx%)
Overall	xx (xx%)	xx (xx%)	xx (xx%)
Peripheral Site 1	xx (xx%)	xx (xx%)	xx (xx%)
•	xx (xx%)	xx (xx%)	xx (xx%)
•	xx (xx%)	xx (xx%)	xx (xx%)
•	xx (xx%)	xx (xx%)	xx (xx%)
Peripheral Site n	xx (xx%)	xx (xx%)	xx (xx%)

TABLE 1.2

NUMBER OF RANDOMISED PATIENTS AT EACH VISIT

	Octreotide-LAR	Placebo	Total
Visit 2 (Month 0)			
Full Analysis Set	xx	xx	xx
Per Protocol Population	xx	xx	xx
Visit 4 (Month 6)			
Full Analysis Set	xx (xx%)	xx (xx%)	xx (xx%)
Per Protocol Population	xx (xx%)	xx (xx%)	xx (xx%)
Visit 6 (Month 12)			
Full Analysis Set	xx (xx%)	xx (xx%)	xx (xx%)
Per Protocol Population	xx (xx%)	xx (xx%)	xx (xx%)
Visit 8 (Month 18)			
Full Analysis Set	xx (xx%)	xx (xx%)	xx (xx%)
Per Protocol Population	xx (xx%)	xx (xx%)	xx (xx%)
●			
●			
●			
At 6 monthly intervals until final (at least 3 years)			

TABLE 1.3

REASONS FOR WITHDRAWAL

	Octreotide-LAR	Placebo	Total
**Number of Patients**	**xx**	**xx**	**xx**
ESRD	xx (xx%)	xx (xx%)	xx (xx%)
Unsatisfactory efficacy	xx (xx%)	xx (xx%)	xx (xx%)
Unsatisfactory compliance	xx (xx%)	xx (xx%)	xx (xx%)
Protocol violation	xx (xx%)	xx (xx%)	xx (xx%)
Lost to follow-up	xx (xx%)	xx (xx%)	xx (xx%)
Withdrawal of consent	xx (xx%)	xx (xx%)	xx (xx%)
Adverse event	xx (xx%)	xx (xx%)	xx (xx%)
Death	xx (xx%)	xx (xx%)	xx (xx%)

Population: Full Analysis Set

TABLE 1.4.1

MAJOR PROTOCOL VIOLATIONS

	Octreotide-LAR	Placebo	Total
**Number of Patients**	**xx**	**xx**	**xx**
Major protocol violation N. 1	xx (xx%)	xx (xx%)	xx (xx%)
…	xx (xx%)	xx (xx%)	xx (xx%)
…	xx (xx%)	xx (xx%)	xx (xx%)
…	xx (xx%)	xx (xx%)	xx (xx%)
…	xx (xx%)	xx (xx%)	xx (xx%)
…	xx (xx%)	xx (xx%)	xx (xx%)
…	xx (xx%)	xx (xx%)	xx (xx%)
Major protocol violation N. n	xx (xx%)	xx (xx%)	xx (xx%)

Population: Full Analysis Set

TABLE 1.4.2

MINOR PROTOCOL VIOLATIONS

	Octreotide-LAR	Placebo	Total
**Number of Patients**	**xx**	**xx**	**xx**
Minor protocol violation N. 1	xx (xx%)	xx (xx%)	xx (xx%)
…	xx (xx%)	xx (xx%)	xx (xx%)
…	xx (xx%)	xx (xx%)	xx (xx%)
…	xx (xx%)	xx (xx%)	xx (xx%)
…	xx (xx%)	xx (xx%)	xx (xx%)
…	xx (xx%)	xx (xx%)	xx (xx%)
…	xx (xx%)	xx (xx%)	xx (xx%)
Minor protocol violation N. n	xx (xx%)	xx (xx%)	xx (xx%)

Population: Full Analysis Set

TABLE 1.5

ADHERENCE TO STUDY SCHEDULE

Scheduled Time of Visit<br>Actual Time of Visit	Octreotide-LAR	Placebo	Total
Visit 3 (3 Months)			
**Number of Patients**	**xx**	**xx**	**xx**
>4 days before	xx (xx%)	xx (xx%)	xx (xx%)
>2 days before	xx (xx%)	xx (xx%)	xx (xx%)
on-time	xx (xx%)	xx (xx%)	xx (xx%)
>2 days after	xx (xx%)	xx (xx%)	xx (xx%)
>4 days after	xx (xx%)	xx (xx%)	xx (xx%)
Visit 4 (6 Months)			
**Number of Patients**	**xx**	**xx**	**xx**
>2 weeks before	xx (xx%)	xx (xx%)	xx (xx%)
>1 week before	xx (xx%)	xx (xx%)	xx (xx%)
on-time	xx (xx%)	xx (xx%)	xx (xx%)
>1 week after	xx (xx%)	xx (xx%)	xx (xx%)
>2 weeks after	xx (xx%)	xx (xx%)	xx (xx%)
Visit 6 (12Months)			
**Number of Patients**	xx	xx	xx
>2 weeks before			
>1 week before	xx (xx%)	xx (xx%)	xx (xx%)
on-time	xx (xx%)	xx (xx%)	xx (xx%)
>1 week after	xx (xx%)	xx (xx%)	xx (xx%)
>2 weeks after	xx (xx%)	xx (xx%)	xx (xx%)
	xx (xx%)	xx (xx%)	xx (xx%)

•
•
•
At 6 monthly intervals until final (at least 3 years)

Population: Full Analysis Set

TABLE 2.1.1

DEMOGRAPHY

	Octreotide-LAR	Placebo	Total
**Number of Patients**	**xx**	**xx**	**xx**
Age (years) at randomization			
Mean (sd)	xx.x (xx.xx)	xx (x.xx)	xx (xx.xx)
Median [Q1 to Q3]	xx.x [x.x to x.x]	xx.x[x.x to x.x]	xx.x[x.x to x.x]
Minimum	xx	xx	xx
Maximum	xx	xx	xx
n			
<40	xx (xx%)	xx (xx%)	xx (xx%)
40-49	xx (xx%)	xx (xx%)	xx (xx%)
50-59	xx (xx%)	xx (xx%)	xx (xx%)
60-69	xx (xx%)	xx (xx%)	xx (xx%)
70-79	xx (xx%)	xx (xx%)	xx (xx%)
>79	xx (xx%)	xx (xx%)	xx (xx%)
Sex			
Male	xx (xx%)	xx (xx%)	xx (xx%)
Female	xx (xx%)	xx (xx%)	xx (xx%)
Race			
Caucasian	xx (xx%)	xx (xx%)	xx (xx%)
Black	xx (xx%)	xx (xx%)	xx (xx%)
Asian	xx (xx%)	xx (xx%)	xx (xx%)
Other	xx (xx%)	xx (xx%)	xx (xx%)

Population: Full Analysis Set

[Note: All tables in Section 2 will additionally be produced for the Per Protocol Set and according to Stratum CCC absent or Stratum CCC present]

TABLE 2.2.1

VITAL SIGNS AT VISIT 2 (MONTH 0)

	Octreotide-LAR	Placebo	Total
**Number of Patients**	**xx**	**xx**	**xx**
Mean systolic blood pressure (mmHg)			
Mean (sd)	xxx.x (xxx.xx)	xxx.x (xxx.xx)	xxx.x (xxx.xx)
Median	xxx.x	xxx.x	xxx.x
Minimum	xxx	xxx	xxx
Maximum	xxx	xxx	xxx
n	xx	xx	xx
Mean diastolic blood pressure (mmHg)			
Mean (sd)	xxx.x (xxx.xx)	xxx.x (xxx.xx)	xxx.x (xxx.xx)
Median	xxx.x	xxx.x	xxx.x
Minimum	xxx	xxx	xxx
Maximum	xxx	xxx	xxx
n	xx	xx	xx
Mean arterial pressure			
(mmHg)	xxx.x (xxx.xx)	xxx.x (xxx.xx)	xxx.x (xxx.xx)
Mean (sd)	xxx.x	xxx.x	xxx.x
Median	xxx	xxx	xxx
Minimum	xxx	xxx	xxx
Maximum	xx	xx	xx
n			
Mean heart rate (bpm)			
Mean (sd)	xxx.x (xxx.xx)	xxx.x (xxx.xx)	xxx.x (xxx.xx)
Median	xxx.x	xxx.x	xxx.x
Minimum	xxx	xxx	xxx
Maximum	xxx	xxx	xxx
n	xx	xx	xx
Weight (Kg)			
Mean (sd)	xxx.x (xxx.xx)	xxx.x (xxx.xx)	xxx.x (xxx.xx)
Median	xxx.x	xxx.x	xxx.x
Minimum	xxx	xxx	xxx
Maximum	xxx	xxx	xxx
n	xx	xx	xx
ECG result			
Normal	xx (xx%)	xx (xx%)	xx (xx%)
Pathological	xx (xx%)	xx (xx%)	xx (xx%)

Population: Full Analysis Set

TABLE 2.3.1

TKV and height-adjusted TKV AT VISIT 2 (MONTH 0)

	Octreotide-LAR	Placebo	Total
TKV (ml)			
Mean (sd)	xxx.xxx (xxx.xxx)	xxx.xxx (xxx.xxx)	xxx.xxx (xxx.xxx)
Median	xxx.xxx	xxx.xxx	xxx.xxx
Q1	xxx.xx	xxx.xx	xxx.xx
Q3	xxx.xx	xxx.xx	xxx.xx
Minimum	xxx.xx	xxx.xx	xxx.xx
Maximum	xxx.xx	xxx.xx	xxx.xx
N	xx	xx	xx
HtTKV (ml)	xxx.xxx (xxx.xxx)	xxx.xxx (xxx.xxx)	xxx.xxx (xxx.xxx)
Mean (sd)	xxx.xxx	xxx.xxx	xxx.xxx
Median	xxx.xx	xxx.xx	xxx.xx
Q1	xxx.xx	xxx.xx	xxx.xx
Q3	xxx.xx	xxx.xx	xxx.xx
Minimum	xxx.xx	xxx.xx	xxx.xx
Maximum	xx	xx	xx
n			

Population: Full Analysis Set

TABLE 2.4.1

MEASURED GLOMERULAR FILTRATION RATE AT VISIT 2 (MONTH 0)

	Octreotide-LAR	Placebo	Total
mGFR (ml/min x 1.73m²)			
Mean (sd)	xxx.xxx (xxx.xxx)	xxx.xxx (xxx.xxx)	xxx.xxx (xxx.xxx)
Median	xxx.xxx	xxx.xxx	xxx.xxx
Q1	xxx.xx	xxx.xx	xxx.xx
Q3	xxx.xx	xxx.xx	xxx.xx
Minimum	xxx.xx	xxx.xx	xxx.xx
Maximum	xxx.xx	xxx.xx	xxx.xx
n	xx	xx	xx

Population: Full Analysis Set

[Note: Tables 2.5-2.46 will be produced in a similar format]

TABLE 2.47.1

PREVIOUS DISEASES

	Octreotide-LAR	Placebo	Total
**Numer of Patients**	**xx**	**xx**	**xx**
Preferred Term 1	xx (xx%)	xx (xx%)	xx (xx%)
●	●	●	●
●	●	●	●
●	●	●	●
Preferred Term n	xx (xx%)	xx (xx%)	xx (xx%)

Population: Full Analysis Set

TABLE 2.48.1

PREVIOUS MEDICATION/NON-DRUG THERAPY

	Octreotide-LAR	Placebo	Total
**Numer of Patients**	**xx**	**xx**	**xx**
Preferred Term 1	xx (xx%)	xx (xx%)	xx (xx%)
●	●	●	●
●	●	●	●
●	●	●	●
Preferred Term n	xx (xx%)	xx (xx%)	xx (xx%)

Population: Full Analysis Set

TABLE 2.49.1

CONCOMITANT DISEASES

	Octreotide-LAR	Placebo	Total
**Numer of Patients**	**xx**	**xx**	**xx**
Preferred Term 1	xx (xx%)	xx (xx%)	xx (xx%)
●	●	●	●
●	●	●	●
●	●	●	●
Preferred Term n	xx (xx%)	xx (xx%)	xx (xx%)

Population: Full Analysis Set

TABLE 2.50.1

CONCOMITANT MEDICATION/NON-DRUG THERAPY

	Octreotide-LAR	Placebo	Total
**Number of Patients**	**xx**	**xx**	**xx**
Preferred Term 1	xx (xx%)	xx (xx%)	xx (xx%)
●	●	●	●
●	●	●	●
●	●	●	●
Preferred Term n	xx (xx%)	xx (xx%)	xx (xx%)

Population: Full Analysis Set

Table 3.1.1 . TKV and height-adjusted TKV, and absolute changes in kidney and cyst volume in participan
randomized to Octreotide-LAR or placebo (overall: stratum CCC absent+ stratum CCC present)

		**Octreotide-LAR**			**Pla**	
		0	1	3	0	
			Years			Y
**TKV**						
	Mean (SD) (*mL)*<br>Median [IQR]	xx.xx (xx.xx)<br>xx.xx [x.x to x.x]	xx.xx (xx.xx)<br>xx.xx [x.x to x.x]	xx.xx (xx.xx)<br>xx.xx [x.x to x.x]	xx.xx (xx.xx)<br>xx.xx [x.x to x.x]	xx.xx (x<br>xx.xx [x
	p value vs.plac. at 1 y					
	p value vs.plac. at 3 y					
	Absolute change (*mL)*	--	xx.xx (xx.xx)<br>xx.xx [x.x to x.x]	xx.xx (xx.xx)<br>xx.xx [x.x to x.x]	--	xx.xx (x<br>xx.xx [x
	Annual slope (*mL/year)*			xx.xx (xx.xx)<br>xx.xx [x.x to x.x]		
	Chronic slope (*mL/year)*			xx.xx (xx.xx)<br>xx.xx [x.x to x.x]		
**htTKV**						
	Mean (SD) (*mL)*<br>Median [IQR]	xx.xx (xx.xx)<br>xx.xx [x.x to x.x]	xx.xx (xx.xx)<br>xx.xx [x.x to x.x]	xx.xx (xx.xx)<br>xx.xx [x.x to x.x]	xx.xx (xx.xx)<br>xx.xx [x.x to x.x]	xx.xx (x<br>xx.xx [x
	p value vs.plac. at 1 y					
	p value vs.plac. at 3 y					
	Absolute change (*mL)*	--	xx.xx (xx.xx)<br>xx.xx [x.x to x.x]	xx.xx (xx.xx)<br>xx.xx [x.x to x.x]	--	xx.xx (x<br>xx.xx [x
	Annual slope (*mL/year)*			xx.xx (xx.xx)<br>xx.xx [x.x to x.x]		
	Chronic slope (*mL/year)*			xx.xx (xx.xx)<br>xx.xx [x.x to x.x]		

TKV, Total Kidney Volume; HtTKV, Height –adjusted Total Kidney Volume

Table 3.1.2 TKV and height-adjusted TKV, and absolute changes in kidney and cyst volume in participants randomized to Octreotide-LAR or placebo. Stratum: CCC absent

		Octreotide-LAR			Pla	
		0	1	3	0	
			Years			Y
**TKV**						
	Mean (SD) (*mL)*<br>Median [IQR]	xx.xx (xx.xx)<br>xx.xx [x.x to x.x]	xx.xx (xx.xx)<br>xx.xx [x.x to x.x]	xx.xx (xx.xx)<br>xx.xx [x.x to x.x]	xx.xx (xx.xx)<br>xx.xx [x.x to x.x]	xx.xx (x<br>xx.xx [x
	p value vs.plac. at 1 y					
	p value vs.plac. at 3 y					
	Absolute change (*mL)*	--	xx.xx (xx.xx)<br>xx.xx [x.x to x.x]	xx.xx (xx.xx)<br>xx.xx [x.x to x.x]	--	xx.xx (x<br>xx.xx [x
	Annual slope (*mL/year)*			xx.xx (xx.xx)<br>xx.xx [x.x to x.x]		
	Chronic slope (*mL/year)*			xx.xx (xx.xx)<br>xx.xx [x.x to x.x]		
**htTKV**						
	Mean (SD) (*mL)*<br>Median [IQR]	xx.xx (xx.xx)<br>xx.xx [x.x to x.x]	xx.xx (xx.xx)<br>xx.xx [x.x to x.x]	xx.xx (xx.xx)<br>xx.xx [x.x to x.x]	xx.xx (xx.xx)<br>xx.xx [x.x to x.x]	xx.xx (x<br>xx.xx [x
	p value vs.plac. at 1 y					
	p value vs.plac. at 3 y					
	Absolute change (*mL)*	--	xx.xx (xx.xx)<br>xx.xx [x.x to x.x]	xx.xx (xx.xx)<br>xx.xx [x.x to x.x]	--	xx.xx (x<br>xx.xx [x
	Annual slope (*mL/year)*			xx.xx (xx.xx)<br>xx.xx [x.x to x.x]		
	Chronic slope (*mL/year)*			xx.xx (xx.xx)<br>xx.xx [x.x to x.x]		

TKV, Total Kidney Volume; HtTKV, Height –adjusted Total Kidney Volume.

Table 3.1.3 TKV and height-adjusted TKV, and absolute changes in kidney and cyst volume in participant randomized to Octreotide-LAR or placebo. Stratum: CCC present

		**Octreotide-LAR**			**Pla**	
		0	1	3	0	
			Years			Y
**TKV**						
	Mean (SD) (*mL)*<br>Median [IQR]	xx.xx (xx.xx)<br>xx.xx [x.x to x.x]	xx.xx (xx.xx)<br>xx.xx [x.x to x.x]	xx.xx (xx.xx)<br>xx.xx [x.x to x.x]	xx.xx (xx.xx)<br>xx.xx [x.x to x.x]	xx.xx (x<br>xx.xx [x
	p value vs.plac. at 1 y					
	p value vs.plac. at 3 y					
	Absolute change (*mL)*	--	xx.xx (xx.xx)<br>xx.xx [x.x to x.x]	xx.xx (xx.xx)<br>xx.xx [x.x to x.x]	--	xx.xx (x<br>xx.xx [x
	Annual slope (*mL/year)*			xx.xx (xx.xx)<br>xx.xx [x.x to x.x]		
	Chronic slope (*mL/year)*			xx.xx (xx.xx)<br>xx.xx [x.x to x.x]		
**htTKV**						
	Mean (SD) (*mL)*<br>Median [IQR]	xx.xx (xx.xx)<br>xx.xx [x.x to x.x]	xx.xx (xx.xx)<br>xx.xx [x.x to x.x]	xx.xx (xx.xx)<br>xx.xx [x.x to x.x]	xx.xx (xx.xx)<br>xx.xx [x.x to x.x]	xx.xx (x<br>xx.xx [x
	p value vs.plac. at 1 y					
	p value vs.plac. at 3 y					
	Absolute change (*mL)*	--	xx.xx (xx.xx)<br>xx.xx [x.x to x.x]	xx.xx (xx.xx)<br>xx.xx [x.x to x.x]	--	xx.xx (x<br>xx.xx [x
	Annual slope (*mL/year)*			xx.xx (xx.xx)<br>xx.xx [x.x to x.x]		
	Chronic slope (*mL/year)*			xx.xx (xx.xx)<br>xx.xx [x.x to x.x]		

TKV, Total Kidney Volume; HtTKV, Height –adjusted Total Kidney Volume.

Table 3.2. Measured Glomerular Filtration Rate (mGFR) at baseline, 6-month, and 1-year, 2-year and 3-year follow-up in the study group as a whole (Overall), and in the two strata (CCC absent or CCC present) considered separately according to treatment with Octreotide-LAR or placebo.

		OCTREOTIDE-LAR						
		Baseline (n=xx)	6 mo (n=xx)	1 yr (n=xx)	2 yrs (n=xx)	3 yrs (n=xx)	Baseline (n=xx)	6 m (n=x
**Overall**	*Actual value**	xx.x [xx.x to xx.x]	xx.x [xx.x to xx.x]	xx.x [xx.x to xx.x]	xx.x [xx.x to xx.x]	xx.x [xx.x to xx.x]	xx.x [xx.x to xx.x]	xx.x [xx.x to x
	Total Slope° 0-3 yrs					xx.x [xx.x to xx.x]		
	Chronic slope° 6 mo-3 yrs					xx.x [xx.x to xx.x]		
**CCC absent**	*Actual value**	xx.x [xx.x to xx.x]	xx.x [xx.x to xx.x]	xx.x [xx.x to xx.x]	xx.x [xx.x to xx.x]	xx.x [xx.x to xx.x]	xx.x [xx.x to xx.x]	xx.x [xx.x to x
	Total Slope 0-3 yrs					xx.x [xx.x to xx.x]		
	Chronic slope 6 mo-3 yrs					xx.x [xx.x to xx.x]		
**CCC present**	*Actual value**	xx.x [xx.x to xx.x]	xx.x [xx.x to xx.x]	xx.x [xx.x to xx.x]	xx.x [xx.x to xx.x]	xx.x [xx.x to xx.x]	xx.x [xx.x to xx.x]	xx.x [xx.x to x
	Total Slope 0-3 yrs					xx.x [xx.x to xx.x]		
	Chronic slope 6 mo-3 yrs					xx.x [xx.x to xx.x]		

Annual slope value is median [interquartile range, IQR].mGFR=measured glomerular filtration rate, * ml/min/1.73m^2; ° ml/min/1.73m^{2}per year

[Note: All tables in Section 3 will additionally be produced for the Per Protocol Set and according to Stratum CCC absent or Stratum CCC present]

TABLE 3.3.1

SYSTOLIC BLOOD PRESSURE (mmHg) OVER TIME: SUMMARY

	Octreotide-LAR	Placebo
Visit 2 (Month 0)		
Mean (sd)	xxx.x (xxx.xx)	xxx.x (xxx.xx)
Median	xxx.x	xxx.x
Minimum	xxx	xxx
Maximum	xxx	xxx
n	xx	xx
Visit 3 (Month 3)		
Mean (sd)	xxx.x (xxx.xx)	xxx.x (xxx.xx)
Median	xxx.x	xxx.x
Minimum	xxx	xxx
Maximum	xxx	xxx
n	xx	xx
Visit 4 (Month 6)		
Mean (sd)	xxx.x (xxx.xx)	xxx.x (xxx.xx)
Median	xxx.x	xxx.x
Minimum	xxx	xxx
Maximum	xxx	xxx
n	xx	xx
Visit 5 (Month 9)		
Mean (sd)	xxx.x (xxx.xx)	xxx.x (xxx.xx)
Median	xxx.x	xxx.x
Minimum	xxx	xxx
Maximum	xxx	xxx
n	xx	xx
Visit 6 (Month 12)		
Mean (sd)	xxx.x (xxx.xx)	xxx.x (xxx.xx)
Median	xxx.x	xxx.x
Minimum	xxx	xxx
Maximum	xxx	xxx
n	xx	xx
Visit 7 (Month 15)		
Mean (sd)	xxx.x (xxx.xx)	xxx.x (xxx.xx)
Median	xxx.x	xxx.x
Minimum	xxx	xxx
Maximum	xxx	xxx
n	xx	xx
•	•	•
•	•	•
At 3 monthly intervals until final visit (at least 3 years)		

Population: Full Analysis Set

TABLE 3.4.1

DIASTOLIC BLOOD PRESSURE (mmHg) OVER TIME: SUMMARY

	Octreotide-LAR	Placebo
Visit 2 (Month 0)		
Mean (sd)	xxx.x (xxx.xx)	xxx.x (xxx.xx)
Median	xxx.x	xxx.x
Minimum	xxx	xxx
Maximum	xxx	xxx
n	xx	xx
Visit 3 (Month 3)		
Mean (sd)	xxx.x (xxx.xx)	xxx.x (xxx.xx)
Median	xxx.x	xxx.x
Minimum	xxx	xxx
Maximum	xxx	xxx
n	xx	xx
Visit 4 (Month 6)		
Mean (sd)	xxx.x (xxx.xx)	xxx.x (xxx.xx)
Median	xxx.x	xxx.x
Minimum	xxx	xxx
Maximum	xxx	xxx
n	xx	xx
Visit 5 (Month 9)		
Mean (sd)	xxx.x (xxx.xx)	xxx.x (xxx.xx)
Median	xxx.x	xxx.x
Minimum	xxx	xxx
Maximum	xxx	xxx
n	xx	xx
Visit 6 (Month 12)		
Mean (sd)	xxx.x (xxx.xx)	xxx.x (xxx.xx)
Median	xxx.x	xxx.x
Minimum	xxx	xxx
Maximum	xxx	xxx
n	xx	xx
Visit 7 (Month 15)		
Mean (sd)	xxx.x (xxx.xx)	xxx.x (xxx.xx)
Median	xxx.x	xxx.x
Minimum	xxx	xxx
Maximum	xxx	xxx
n	xx	xx
•	•	•
•	•	•

At 3 monthly intervals until final visit (at least 3 years)

Population: Full Analysis Set

TABLE 3.5.1

MEAN ARTERIAL PRESSURE (mmHg) OVER TIME: SUMMARY

	Octreotide-LAR	Placebo
Visit 2 (Month 0)		
Mean (sd)	xxx.x (xxx.xx)	xxx.x (xxx.xx)
Median	xxx.x	xxx.x
Minimum	xxx	xxx
Maximum	xxx	xxx
n	xx	xx
Visit 3 (Month 3)		
Mean (sd)	xxx.x (xxx.xx)	xxx.x (xxx.xx)
Median	xxx.x	xxx.x
Minimum	xxx	xxx
Maximum	xxx	xxx
n	xx	xx
Visit 4 (Month 6)		
Mean (sd)	xxx.x (xxx.xx)	xxx.x (xxx.xx)
Median	xxx.x	xxx.x
Minimum	xxx	xxx
Maximum	xxx	xxx
n	xx	xx
Visit 5 (Month 9)		
Mean (sd)	xxx.x (xxx.xx)	xxx.x (xxx.xx)
Median	xxx.x	xxx.x
Minimum	xxx	xxx
Maximum	xxx	xxx
n	xx	xx
Visit 6 (Month 12)		
Mean (sd)	xxx.x (xxx.xx)	xxx.x (xxx.xx)
Median	xxx.x	xxx.x
Minimum	xxx	xxx
Maximum	xxx	xxx
n	xx	xx
Visit 7 (Month 15)		
Mean (sd)	xxx.x (xxx.xx)	xxx.x (xxx.xx)
Median	xxx.x	xxx.x
Minimum	xxx	xxx
Maximum	xxx	xxx
n	xx	xx
•	•	•
•	•	•
At 3 monthly intervals until final visit (at least 3 years)		

Population: Full Analysis Set

TABLE 3.6.1

Pulse rate (mmHg) OVER TIME: SUMMARY

	Octreotide-LAR	Placebo
Visit 2 (Month 0)		
Mean (sd)	xxx.x (xxx.xx)	xxx.x (xxx.xx)
Median	xxx.x	xxx.x
Minimum	xxx	xxx
Maximum	xxx	xxx
n	xx	xx
Visit 3 (Month 3)		
Mean (sd)	xxx.x (xxx.xx)	xxx.x (xxx.xx)
Median	xxx.x	xxx.x
Minimum	xxx	xxx
Maximum	xxx	xxx
n	xx	xx
Visit 4 (Month 6)		
Mean (sd)	xxx.x (xxx.xx)	xxx.x (xxx.xx)
Median	xxx.x	xxx.x
Minimum	xxx	xxx
Maximum	xxx	xxx
n	xx	xx
Visit 5 (Month 9)		
Mean (sd)	xxx.x (xxx.xx)	xxx.x (xxx.xx)
Median	xxx.x	xxx.x
Minimum	xxx	xxx
Maximum	xxx	xxx
n	xx	xx
Visit 6 (Month 12)		
Mean (sd)	xxx.x (xxx.xx)	xxx.x (xxx.xx)
Median	xxx.x	xxx.x
Minimum	xxx	xxx
Maximum	xxx	xxx
n	xx	xx
Visit 7 (Month 15)		
Mean (sd)	xxx.x (xxx.xx)	xxx.x (xxx.xx)
Median	xxx.x	xxx.x
Minimum	xxx	xxx
Maximum	xxx	xxx
n	xx	xx
•	•	•
•	•	•

At 3 monthly intervals until final visit (at least 3 years)

Population: Full Analysis Set

TABLE 3.7.1

Weight (Kg) OVER TIME: SUMMARY

	Octreotide-LAR	Placebo
Visit 2 (Month 0)		
Mean (sd)	xxx.x (xxx.xx)	xxx.x (xxx.xx)
Median	xxx.x	xxx.x
Minimum	xxx	xxx
Maximum	xxx	xxx
n	xx	xx
Visit 3 (Month 3)		
Mean (sd)	xxx.x (xxx.xx)	xxx.x (xxx.xx)
Median	xxx.x	xxx.x
Minimum	xxx	xxx
Maximum	xxx	xxx
n	xx	xx
Visit 4 (Month 6)		
Mean (sd)	xxx.x (xxx.xx)	xxx.x (xxx.xx)
Median	xxx.x	xxx.x
Minimum	xxx	xxx
Maximum	xxx	xxx
n	xx	xx
Visit 5 (Month 9)		
Mean (sd)	xxx.x (xxx.xx)	xxx.x (xxx.xx)
Median	xxx.x	xxx.x
Minimum	xxx	xxx
Maximum	xxx	xxx
n	xx	xx
Visit 6 (Month 12)		
Mean (sd)	xxx.x (xxx.xx)	xxx.x (xxx.xx)
Median	xxx.x	xxx.x
Minimum	xxx	xxx
Maximum	xxx	xxx
n	xx	xx
Visit 7 (Month 15)		
Mean (sd)	xxx.x (xxx.xx)	xxx.x (xxx.xx)
Median	xxx.x	xxx.x
Minimum	xxx	xxx
Maximum	xxx	xxx
n	xx	xx
•	•	•
•	•	•

At 3 monthly intervals until final visit (at least 3 years)

Population: Full Analysis Set

TABLE 3.8.1

SERUM CREATININE (mg/dl) OVER TIME: SUMMARY

	Octreotide-LAR	Placebo
Visit 2 (Month 0)		
Mean (sd)	xxx.x (xxx.xx)	xxx.x (xxx.xx)
Median	xxx.x	xxx.x
Minimum	xxx	xxx
Maximum	xxx	xxx
n	xx	xx
Visit 3 (Month 3)		
Mean (sd)	xxx.x (xxx.xx)	xxx.x (xxx.xx)
Median	xxx.x	xxx.x
Minimum	xxx	xxx
Maximum	xxx	xxx
n	xx	xx
Visit 4 (Month 6)		
Mean (sd)	xxx.x (xxx.xx)	xxx.x (xxx.xx)
Median	xxx.x	xxx.x
Minimum	xxx	xxx
Maximum	xxx	xxx
n	xx	xx
Visit 5 (Month 9)		
Mean (sd)	xxx.x (xxx.xx)	xxx.x (xxx.xx)
Median	xxx.x	xxx.x
Minimum	xxx	xxx
Maximum	xxx	xxx
n	xx	xx
Visit 6 (Month 12)		
Mean (sd)	xxx.x (xxx.xx)	xxx.x (xxx.xx)
Median	xxx.x	xxx.x
Minimum	xxx	xxx
Maximum	xxx	xxx
n	xx	xx
Visit 7 (Month 15)		
Mean (sd)	xxx.x (xxx.xx)	xxx.x (xxx.xx)
Median	xxx.x	xxx.x
Minimum	xxx	xxx
Maximum	xxx	xxx
n	xx	xx
•	•	•
•	•	•

At 3 monthly intervals until final visit (at least 3 years)

Population: Full Analysis Set

[Note: Tables **3.9.1-3.25.3, 3.26.1-3.26.3, 3.27.1-3.33. 3**, **3.34.1-3.38.3, 3.39.1-3.39.3, 3.40.1-3.40.3, 3.41.1-3.44.3, 3.45.1-3.49.3, 3.50.1-3.51.3,** *will be displayed as Table 3.8.1]*

Table 3.52 Estimated Glomerular Filtration Rate using MDRD formula ($eGFR_{mdrd}$) at baseline, 6-month, and 1-year, 2-year and 3-year follow-up in the study group as a whole (Overall), and in the two strata (CCC absent or CCC present) considered separately according to treatment with Octreotide-LAR or placebo.

		OCTREOTIDE-LAR						
		Baseline (n=xx)	6 mo (n=xx)	1 yr (n=xx)	2 yrs (n=xx)	3 yrs (n=xx)	Baseline (n=xx)	6 m (n=x
**Overall**	*Actual value**	xx.x [xx.x to xx.x]	xx.x [xx.x to xx.x]	xx.x [xx.x to xx.x]	xx.x [xx.x to xx.x]	xx.x [xx.x to xx.x]	xx.x [xx.x to xx.x]	xx.x [xx.x to
	Total Slope° 0-3 yrs					xx.x [xx.x to xx.x]		
	Chronic slope° 6 mo-3 yrs					xx.x [xx.x to xx.x]		
**CCC absent**	*Actual value**	xx.x [xx.x to xx.x]	xx.x [xx.x to xx.x]	xx.x [xx.x to xx.x]	xx.x [xx.x to xx.x]	xx.x [xx.x to xx.x]	xx.x [xx.x to xx.x]	xx.x [xx.x to
	Total Slope 0-3 yrs					xx.x [xx.x to xx.x]		
	Chronic slope 6 mo-3 yrs					xx.x [xx.x to xx.x]		
**CCC present**	*Actual value**	xx.x [xx.x to xx.x]	xx.x [xx.x to xx.x]	xx.x [xx.x to xx.x]	xx.x [xx.x to xx.x]	xx.x [xx.x to xx.x]	xx.x [xx.x to xx.x]	xx.x [xx.x to
	Total Slope 0-3 yrs					xx.x [xx.x to xx.x]		
	Chronic slope 6 mo-3 yrs					xx.x [xx.x to xx.x]		

Annual $eGFR_{mdrd}$ slope value is median [interquartile range, IQR].eGFR=estimated glomerular filtration rate, * ml/min/1.73m^2; ° ml/min/1.73m^{2}per year

Table 3.52 Estimated Glomerular Filtration Rate using CKI-EPI formula ($eGFR_{ckd\text{-}epi}$) at baseline, 6-month, and 1-year, 2-year and 3-year follow-up in the study group as a whole (Overall), and in the two strata (CCC absent or CCC present) considered separately according to treatment with Octreotide-LAR or placebo.

		OCTREOTIDE-LAR						
		Baseline (n=xx)	6 mo (n=xx)	1 yr (n=xx)	2 yrs (n=xx)	3 yrs (n=xx)	Baseline (n=xx)	6 m (n=x
**Overall**	*Actual value**	xx.x [xx.x to xx.x]	xx.x [xx.x to xx.x]	xx.x [xx.x to xx.x]	xx.x [xx.x to xx.x]	xx.x [xx.x to xx.x]	xx.x [xx.x to xx.x]	xx.x [xx.x to
	Total Slope° 0-3 yrs					xx.x [xx.x to xx.x]		
	Chronic slope° 6 mo-3 yrs					xx.x [xx.x to xx.x]		
**CCC absent**	*Actual value**	xx.x [xx.x to xx.x]	xx.x [xx.x to xx.x]	xx.x [xx.x to xx.x]	xx.x [xx.x to xx.x]	xx.x [xx.x to xx.x]	xx.x [xx.x to xx.x]	xx.x [xx.x to
	Total Slope 0-3 yrs					xx.x [xx.x to xx.x]		
	Chronic slope 6 mo-3 yrs					xx.x [xx.x to xx.x]		
**CCC present**	*Actual value**	xx.x [xx.x to xx.x]	xx.x [xx.x to xx.x]	xx.x [xx.x to xx.x]	xx.x [xx.x to xx.x]	xx.x [xx.x to xx.x]	xx.x [xx.x to xx.x]	xx.x [xx.x to
	Total Slope 0-3 yrs					xx.x [xx.x to xx.x]		

Chronic slope<br>6 mo-3 yrs	xx.x<br>[xx.x to xx.x]

Annual $eGFR_{ckd-epi}$ slope value is median [interquartile range, IQR].eGFR=estimated glomerular filtration rate, * ml/min/1.73m^2; ° ml/min/1.73m^{2}per year

TABLE 3.53.1

TIME TO ESRD: SUMMARY

	Octreotide-LAR	Placebo
**Number of Patients**	**xx**	**xx**
Number of patients events-free		
6 months	xx (xx%)	xx (xx%)
12 months	xx (xx%)	xx (xx%)
•	•	•
•	•	•
•	•	•
Median time to event (95% CI[a])	xx.x (xx.x to xx.x)	xx.x (xx.x to xx.x)
Number (%) of censored patients	xx (xx%)	xx (xx%)

Population: Full Analysis Set

[a] Calculated using Greenwood's formula

TABLE 3.53.2

TIME TO ESRD: STATISTICAL ANALYSIS (PH MODEL)

Covariate	Parameter Estimate	Standard Error	p-value
Age	x.xx	x.xx	x.xxx
Sex	x.xx	x.xx	x.xxx
Baseline Serum Creatinine	x.xx	x.xx	x.xxx

Treatment Comparison	Hazard Ratio (SE)	95% CI for the Hazard Ratio	p-value[a]
Octreotide-LAR vs Placebo	xx.x (xx.x)	xx.x to xx.x	x.xxx

Population: Full Analysis Set

[a] A p-value <0.05 will be considered to be evidence of a significant difference between the treatment groups

TABLE 3.54.1

TIME TO DOUBLING OF SERUM CREATININE OR ESRD: SUMMARY

	Octreotide-LAR	Placebo
**Number of Patients**	**xx**	**xx**
Number of patients events-free		
6 months	xx (xx%)	xx (xx%)
12 months	xx (xx%)	xx (xx%)
●	●	●
●	●	●
●	●	●
Median time to event (95% CI[a])	xx.x (xx.x to xx.x)	xx.x (xx.x to xx.x)
Number (%) of censored patients	xx (xx%)	xx (xx%)

Population: Full Analysis Set

[a] Calculated using Greenwood's formula

TABLE 3.54.2

TIME TO DOUBLING OF SERUM CREATININE OR ESRD: STATISTICAL ANALYSIS (PH MODEL)

Covariate	Parameter Estimate	Standard Error	p-value

Age	x.xx	x.xx	x.xxx
Sex	x.xx	x.xx	x.xxx
Baseline Serum Creatinine	x.xx	x.xx	x.xxx

Treatment Comparison	Hazard Ratio (SE)	95% CI for the Hazard Ratio	p-value[a]
Octreotide-LAR vs Placebo	xx.x (xx.x)	xx.x to xx.x	x.xxx

Population: Full Analysis Set

[a] A p-value <0.05 will be considered to be evidence of a significant difference between the treatment groups

TABLE 3.55.1

TIME TO OVERALL MORTALITY: SUMMARY

	Octreotide-LAR	Placebo
**Number of Patients**	**xx**	**xx**
Number of patients events-free		
6 months	xx (xx%)	xx (xx%)
12 months	xx (xx%)	xx (xx%)
•	•	•
•	•	•
•	•	•
Median time to event (95% CI[a])	xx.x (xx.x to xx.x)	xx.x (xx.x to xx.x)
Number (%) of censored patients	xx (xx%)	xx (xx%)

Population: Full Analysis Set

a Calculated using Greenwood's formula

TABLE 3.55.2

TIME TO OVERALL MORTALITY: STATISTICAL ANALYSIS (PH MODEL)

Covariate	Parameter Estimate	Standard Error	p-value
Age	x.xx	x.xx	x.xxx
Sex	x.xx	x.xx	x.xxx
Baseline Serum Creatinine	x.xx	x.xx	x.xxx

Treatment Comparison	Hazard Ratio (SE)	95% CI for the Hazard Ratio	p-value[a]
Octreotide-LAR vs Placebo	xx.x (xx.x)	xx.x to xx.x	x.xxx

Population: Full Analysis Set

[a] A p-value <0.05 will be considered to be evidence of a significant difference between the treatment groups

TABLE 3.56.1

TIME TO CARDIOVASCULAR MORTALITY: SUMMARY

	Octreotide-LAR	Placebo
**Number of Patients**	**xx**	**xx**
Number of patients events-free		
6 months	xx (xx%)	xx (xx%)
12 months	xx (xx%)	xx (xx%)
•	•	•
•	•	•
•	•	•
Median time to event (95% CI[a])	xx.x (xx.x to xx.x)	xx.x (xx.x to xx.x)
Number (%) of censored patients	xx (xx%)	xx (xx%)

Population: Full Analysis Set

a Calculated using Greenwood's formula

TABLE 3.56.2

TIME TO CARDIOVASCULAR MORTALITY: STATISTICAL ANALYSIS (PH MODEL)

Covariate	Parameter Estimate	Standard Error	p-value
Age	x.xx	x.xx	x.xxx
Sex	x.xx	x.xx	x.xxx
Baseline Serum Creatinine	x.xx	x.xx	x.xxx

Treatment Comparison	Hazard Ratio (SE)	95% CI for the Hazard Ratio	p-value[a]
Octreotide-LAR vs Placebo	xx.x (xx.x)	xx.x to xx.x	x.xxx

Population: Full Analysis Set

[a] A p-value <0.05 will be considered to be evidence of a significant difference between the treatment groups

TABLE 3.57.1

TIME TO NON-CARDIOVASCULAR MORTALITY: SUMMARY

	Octreotide-LAR	Placebo

**Number of Patients**	**xx**	**xx**
Number of patients events-free		
6 months	xx (xx%)	xx (xx%)
12 months	xx (xx%)	xx (xx%)
●	●	●
●	●	●
●	●	●
Median time to event (95% CI[a])	xx.x (xx.x to xx.x)	xx.x (xx.x to xx.x)
Number (%) of censored patients	xx (xx%)	xx (xx%)

Population: Full Analysis Set

[a] Calculated using Greenwood's formula

TABLE 3.57.2

TIME TO NON-CARDIOVASCULAR MORTALITY: STATISTICAL ANALYSIS (PH MODEL)

Covariate	Parameter Estimate	Standard Error	p-value
Age	x.xx	x.xx	x.xxx
Sex	x.xx	x.xx	x.xxx
Baseline Serum Creatinine	x.xx	x.xx	x.xxx

Treatment Comparison	Hazard Ratio (SE)	95% CI for the Hazard Ratio	p-value[a]
Octreotide-LAR vs Placebo	xx.x (xx.x)	xx.x to xx.x	x.xxx

Population: Full Analysis Set

[a] A p-value <0.05 will be considered to be evidence of a significant difference between the treatment groups

TABLE 4.1

SUMMARY OF ADVERSE EVENTS

	Octreotide-LAR	Placebo
**Number of Patients**	**xx**	**xx**
No (%) of patients with an adverse event	xx (xx%)	xx (xx%)
No (%) of patients with a serious adverse event	xx (xx%)	xx (xx%)
No (%) of patients with a non -serious adverse event	xx (xx%)	xx (xx%)
No (%) of patients with a severe adverse event	xx (xx%)	xx (xx%)
No (%) of patients with an event probably related to study drug	xx (xx%)	xx (xx%)
No (%) of patients with an adverse event leading to permanent discontinuation	xx (xx%)	xx (xx%)
Average number of events per patient	xx.x	xx.x
Average number of events per patient (with a least 1 event)	xx.x	xx.x
No (%) of deaths	xx (xx%)	xx (xx%)

Population: Full Analysis Set

TABLE 4.2

SUMMARY OF SERIOUS ADVERSE EVENTS

	Octreotide-LAR	Placebo
**Number of Patients**	**xx**	**xx**
No (%) of patients with an adverse event	xx (xx%)	xx (xx%)
No (%) of patients with a severe adverse event	xx (xx%)	xx (xx%)
No (%) of patients with an event probably related to study drug	xx (xx%)	xx (xx%)
Average number of events per patient	xx.x	xx.x
Average number of events per patient (with a least 1 event)	xx.x	xx.x
No (%) of deaths	xx (xx%)	xx (xx%)

Population: Full Analysis Set

TABLE 4.3

FREQUENCY OF ADVERSE EVENTS BY BODY SYSTEM AND PREFERRED TERM

	Octreotide-LAR	Placebo
**Number of Patients**	**xx**	**xx**
Body System 1	xx (xx%)	xx (xx%)
Preferred Term 1	xx (xx%)	xx (xx%)
●	●	●
●	●	●
●	●	●
Preferred Term n	xx (xx%)	xx (xx%)
●	●	●
●	●	●
●	●	●
Body System n	xx (xx%)	xx (xx%)
Preferred Term 1	xx (xx%)	xx (xx%)
●	●	●
●	●	●
●	●	●
Preferred Term n	xx (xx%)	xx (xx%)

Population: Full Analysis Set

TABLE 4.4

FREQUENCY OF SERIOUS ADVERSE EVENTS BY BODY SYSTEM AND PREFERRED TERM

	Octreotide-LAR	Placebo
**Number of Patients**	**xx**	**xx**
Body System 1	xx (xx%)	xx (xx%)
Preferred Term 1	xx (xx%)	xx (xx%)
●	●	●
●	●	●
●	●	●
Preferred Term n	xx (xx%)	xx (xx%)
●	●	●
●	●	●
●	●	●
Body System n	xx (xx%)	xx (xx%)
Preferred Term 1	xx (xx%)	xx (xx%)
●	●	●
●	●	●
●	●	●
Preferred Term n	xx (xx%)	xx (xx%)

Population: Full Analysis Set

[Note: Tables 4.7 to 4.9 will be produced using this template. See Section 4 of Statistical Analysis Plan for table titles]

TABLE 4.5

PATIENT DEATHS

	Octreotide-LAR	Placebo
**Number of Patients**	**xx**	**xx**
Number (%) of Deaths	xx (xx%)	xx (xx%)
Reason for Death		
Reason 1	xx (xx%)	xx (xx%)
●	●	●
●	●	●
●	●	●
Reason n	xx (xx%)	xx (xx%)

Population: Full Analysis Set

TABLE 4.6

LABORATORY TESTS AT BASELINE (VISIT B1-WEEK 0)

Laboratory Parameter	Octreotide-LAR	Placebo
Creatinine (mg/dl)		
Mean (sd)	xxx.x (xxx.xx)	xxx.x (xxx.xx)
Median	xxx.x	xxx.x
Minimum	xxx	xxx
Maximum	xxx	xxx
n	xx	xx
N§	xx (xx%)	xx (xx%)
A§	xx (xx%)	xx (xx%)
●	●	●
●	●	●
●	●	●
●	●	●
(%)		
Mean (sd)	xxx.x (xxx.xx)	xxx.x (xxx.xx)
Median	xxx.x	xxx.x
Minimum	xxx	xxx
Maximum	xxx	xxx
n	xx	xx
Low	xx (xx%)	xx (xx%)
Normal	xx (xx%)	xx (xx%)
High	xx (xx%)	xx (xx%)

Population: Full Analysis Set
§ Note: N=Normal or abnormal, but not clinically relevant; A=Abnormal and clinically relevant

TABLE 4.7

LABORATORY TEST SHIFT TABLE-CREATININE (mg/dl)

	Octreotide-LAR			
	NN§	NA§	AN§	AA§
Visit B2 (Week 1)	xx	xx	xx	xx
Visit B4 (Week 3)	xx	xx	xx	xx
Visit B5 (Week 6)	xx	xx	xx	xx
Visit B6 (Week 9)	xx	xx	xx	xx
●	●	●	●	●
●	●	●	●	●
●	●	●	●	●
Final Visit	xx	xx	xx	xx

	Placebo			
	LL§	LN§	LH§	NL§
Visit B2 (Week 1)	xx	xx	xx	xx
Visit B4 (Week 3)	xx	xx	xx	xx
Visit B5 (Week 6)	xx	xx	xx	xx
Visit B6 (Week 9)	xx	xx	xx	xx
●	●	●	●	●
●	●	●	●	●
●	●	●	●	●
Final Visit	xx	xx	xx	xx

Population: Full Analysis Set
§ Note: N=Normal or abnormal, but not clinically relevant; A=Abnormal and clinically relevant

*[Note: **Tables 4.8 to 4.23** will be produced in a similar format]*

TABLE 4.24

SUMMARY STATISTICS FOR CHANGE FROM BASELINE-CREATININE (mg/dl)

	Octreotide-LAR		Placebo	
	Absolute Value	Change from Baseline	Absolute Value	Change from Baseline
Visit B1 (Week 0)				
Mean (sd)	xx.x (xx.xx)		xx.x (xx.xx)	
Median	xx.x		xx.x	
Minimum	xx		xx	
Maximum	xx		xx	
n	xx		xx	
Visit B2 (Week 1)				
Mean (sd)	xx.x (xx.xx)	xx.x (xx.xx)	xx.x (xx.xx)	xx.x (xx.xx)
Median	xx.x	xx.x	xx.x	xx.x
Minimum	xx	xx	xx	xx
Maximum	xx	xx	xx	xx
n	xx	xx	xx	xx
•				
•				
•				
•				
Final Visit				
Mean (sd)	xx.x (xx.xx)	xx.x (xx.xx)	xx.x (xx.xx)	xx.x (xx.xx)
Median	xx.x	xx.x	xx.x	xx.x
Minimum	xx	xx	xx	xx
Maximum	xx	xx	xx	xx
n	xx	xx	xx	xx

Population: Full Analysis Set

[Note: Tables 4.25 to 4.27 will be produced in a similar format]
